# Riding toward Selectivity: Optimization of Covalent 7‑Azaindole-Based BMX Kinase Inhibitors

**DOI:** 10.1021/acs.jmedchem.6c01366

**Published:** 2026-07-13

**Authors:** Xiaojun Julia Liang, Claudia Albertini, Thales Kronenberger, Ekaterina Shevchenko, Alexander Rasch, Benedict-Tilman Berger, Martin Schwalm, Lena Marie Berger, Andreas Krämer, Ricardo A. M. Serafim, Michael Forster, Apirat Chaikuad, Maria Laura Bolognesi, Susanne Müller, Antti Poso, Stefan Laufer, Stefan Knapp, Matthias Gehringer

**Affiliations:** † Department for Medicinal Chemistry, Institute for Biomedical Engineering, Faculty of Medicine, University of Tübingen, Auf der Morgenstelle 8, 72076 Tübingen, Germany; ‡ Cluster of Excellence iFIT (EXC 2180) ‘Image-Guided & Functionally Instructed Tumor Therapies’, University of Tübingen, 72076 Tübingen, Germany; § Department of Pharmaceutical/Medicinal Chemistry, Institute of Pharmaceutical Sciences, Faculty of Sciences, University of Tübingen, Auf der Morgenstelle 8, 72076 Tübingen, Germany; ∥ Department of Pharmacy and Biotechnology, Alma Mater Studiorum-University of Bologna, Via Belmeloro 6, 40126 Bologna, Italy; ⊥ Tübingen Center for Academic Drug Discovery & Development (TüCAD_2_), 72076 Tübingen, Germany; # School of Pharmacy, Faculty of Health Sciences, University of Eastern Finland, 70211 Kuopio, Finland; ∇ Institute of Medical Microbiology and Hygiene, Interfaculty Institute of Microbiology and Infection Medicine, University of Tübingen, Elfriede-Aulhorn-Str. 6, 72076 Tübingen, Germany; ○ Structural Genomics Consortium, 9173 Goethe University Frankfurt, Buchmann Institute for Molecular Life Sciences, Max-von-Laue-Straße 15, 60438 Frankfurt am Main, Germany; ◆ Institute of Pharmaceutical Chemistry, Goethe University Frankfurt, Buchmann Institute for Molecular Life Sciences, Max-von-Laue-Straße 9, 60438 Frankfurt am Main, Germany; ¶ Frankfurt Cancer Institute (FCI) and German Translational Cancer Network (DKTK) Site Frankfurt/Mainz, 60438 Frankfurt am Main, Germany; ⋈ Department of Organic and Pharmaceutical Chemistry, School of Engineering, Institut Químic de Sarrià (IQS), Universitat Ramon Llull (URL), Vía Augusta 390, 08017 Barcelona, Spain

## Abstract

The tyrosine kinase expressed in hepatocellular carcinoma (TEC) family comprises five nonreceptor tyrosine kinases—BTK, ITK, BMX, TXK, and TEC—with key roles in immune signaling. Although BTK and ITK have been extensively studied, selective inhibitors for TEC, TXK, and BMX remain scarce. Recently, we identified 7-azaindole-based covalent BMX inhibitors with potent inhibitory activity and robust cellular target engagement but limited selectivity across TEC family members, especially BTK. Here, we describe a new generation of BMX inhibitors designed to exploit subtle structural differences between BMX and BTK by incorporating diverse *N*-acylamino substituents at the azaindole 5-position or variations at the linker and warhead. Our compounds display subnanomolar BMX potency and improved selectivity over BTK and other TEC kinases. Key compound **11i** showed strong cellular target engagement, good *in vitro* metabolic stability, a favorable kinome profile, and rapid covalent inactivation kinetics, positioning it among the best BMX chemical probes currently available.

## Graphical abstract

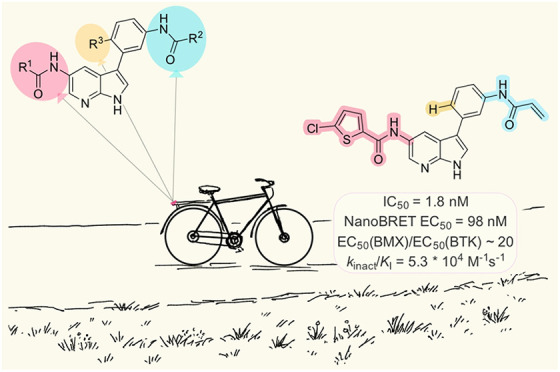

## 1 Introduction

The tyrosine kinase expressed in hepatocellular carcinoma (TEC) family constitutes the second-largest nonreceptor tyrosine kinase family. It includes five members (i) Bruton's tyrosine kinase (BTK), (ii) interleukin-2-inducible T-cell kinase (ITK), (iii) bone marrow tyrosine kinase on chromosome X (BMX), (iv) tyrosine-protein kinase (TXK), and (v) TEC.^1^ BMX was the last of the five TEC kinases to be discovered and it is structurally most closely related to BTK.^1^ Despite the considerable sequence similarity between BMX and BTK, minor sequence differences have been described in the pocket behind the gatekeeper residue (“hydrophobic region I” (HR-I)),^2^ i.e., a phenylalanine (Phe475) in BMX which is a leucine (Leu460) in BTK.^3^ Comparing these two kinases, the steric hindrance of the Phe475 side chain affects not only the conformation of BMX‘s DFG motif but also causes a greater outward tilt of BMX’s αC-helix resulting in a larger HR-I pocket.^3^ Being part of the kinases’ regulatory spine, these differences may also cause differences in protein dynamics. Notably, interactions with the regulatory spine of BTK have recently been described as a key factor in modulating the residence time of inhibitors.^4^ Apart from these differences, the unique phenylalanine present in BMX might serve as a molecular recognition site that mediates BMX-specific interactions such as π–π-stacking or halogen−π-interactions. Additionally, BMX and BTK feature some subtle differences in the front pocket and the hinge region, however, without a direct involvement of nonconserved amino acid side chains in the ATP-binding site.

BMX is mainly expressed in granulocytes, monocytes, epithelial, and endothelial cells, as well as brain, prostate, lung, and heart cells.^5^ However, its physiological and pathological role is still not fully elucidated. It seems to be mainly involved in regulating tumorigenicity,^6^ cell adhesion, motility, angiogenesis, proliferation, and differentiation,^7,8^ possibly playing a role in the therapeutic resistance in acute myeloid leukemia,^9,10^ and in the progression of cardiac hypertrophy.^11^ However, proper validation of BMX as a drug target will require highly specific chemical probes for the exploration of the physiology and pathology of this relatively understudied kinase.^12,13^ Ideally, such probes should also be selective over the closest relatives of BMX, i.e., BTK, as well as the other TEC family members.

TEC family kinases possess a cysteine in the front pocket at the αD-1 position,^14^ which can be used for designing “Targeted Covalent Inhibitors” (TCIs). Such inhibitors contain an electrophilic covalent bond-forming functional group, the so-called “warhead”, which requires a well-balanced reactivity profile to achieve efficient covalent inactivation while maintaining a low degree of promiscuity.^15,16^ TCI binding is a two-step process in which on-target reactivity, described by the rate constant *k*_inact_, can be enhanced by affinity-driven proximity, where the right positioning of the molecule and potential templating effects may cause a significant rate acceleration that enables the specific and efficient targeting of noncatalytic nucleophilic amino acid residues (e.g., cysteine) even with moderately reactive electrophiles (often α,β-unsaturated amides).^15,16^ The overall efficiency of irreversible covalent inhibitors is best described by the ratio *k*_inact_/*K*_I_, a time-independent parameter which has the unit of a second order rate constant (M^–1^ s^–1^). *K*_I_ represents the compound concentration at which the inactivation happens at the half-maximal rate (i.e., at *k*_inact_/2).^17^ In contrast, IC_50_ values of irreversible inhibitors are time-dependent and deliver much less insights compared to kinetic parameters, yet they can remain useful for guiding SAR if properly employed.^18,19^ Unlike cysteine or serine proteases, kinases do not rely on nucleophilic catalysis and do not feature a highly activated active site nucleophile. Many kinases possess noncatalytic cysteines in proximity to the ATP site that hold a lower degree of reactivity and conservation and enable covalent inhibitor design efforts.^14^ However, these cysteines also feature a higher susceptibility to drug resistance mutations.^20,21^

Currently, four irreversible covalent BTK inhibitors, ibrutinib (**1**),^22^ acalabrutinib (**2**),^23^ zanubrutinib (**3**),^24^ and remibrutinib (**4**)^25^ are approved by the US Food and Drug Administration (FDA). While the three first mentioned drugs are mainly used for the treatment of various B-cell lymphoma, remibrutinib is used to treat chronic spontaneous urticaria, an immune-mediated disorder. A fifth covalent BTK inhibitor, rilzabrutinib (**5**),^26^ acts through a reversible covalent mechanism and was recently approved for the treatment of immune thrombocytopenia.^26^ Despite different selectivity profiles, all covalent BTK inhibitors share the same mode-of-action by covalently binding to Cys481, the aforementioned nonconserved αD-1 cysteine in the solvent exposed front pocket of the ATP binding site. Beyond the TEC kinases, this cysteine is present in six other kinases i.e., the human epidermal growth factor receptor (HER)-family kinase EGFR (epidermal growth factor receptor), HER2 and HER4, Janus kinase 3 (JAK3), the mitogen-activated protein kinase kinase 7 (MKK7 or MAP2K7), and the B-lymphoid tyrosine kinase (BLK) belonging to the SRC kinase family.^21^

While providing a possibility to achieve selectivity within the kinome, targeting of this αD-1 cysteine is also a source of possible cross-reactivity among the above-mentioned kinases, which constitutes a considerable challenge. However, it has been shown that covalent inhibitors can achieve certain selectivity windows even between closely related kinases and even if they feature the same nucleophilic amino acid residue.^25,27–29^ Fine-tuning may be achieved by modulating both, noncovalent and covalent binding contributions.

While BTK has been very well explored, the availability of chemical probes to investigate the function of the other members of the TEC family remains limited. Moreover, most current BTK inhibitors possess cross-reactivity within the family. Notably, only very few BMX inhibitors have been developed so far, the first one being BMX-IN-1 (**6**), a benzonaphthyridinone-based compound using an acrylamide warhead to target the αD-1 cysteine Cys496 (Figure 1). Compound **6** shows potent dual activity against BMX and BTK with a half maximal inhibitory potency (IC_50_) in the nanomolar range (BMX IC_50_ = 8 nM and BTK IC_50_ = 10 nM).^30^ The benzonaphthyridinone core was further investigated by Seixas et al., who developed a follow-up series.^31^

**Figure 1:**
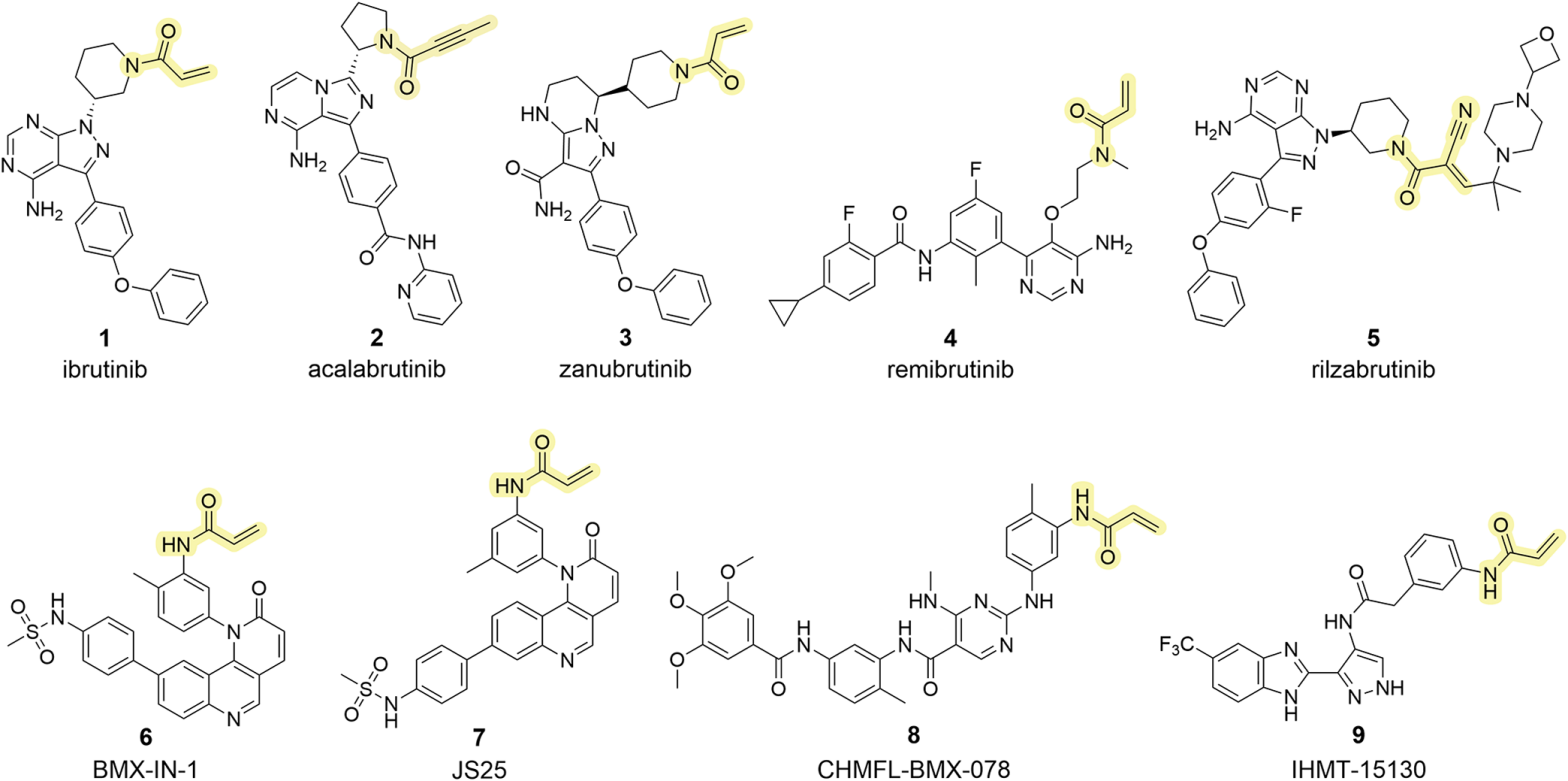
Covalent drugs targeting BTK approved by the FDA: ibrutinib (**1**), acalabrutinib (**2**), zanubrutinib (**3**), remibrutinib (**4**), and reversible covalent inhibitor rilzabrutinib (**5**). Examples of covalent inhibitors addressing BMX as the primary target are represented by BMX-IN-1 (**6**), JS25 (**7**), CHMFL-BMX-078 (**8**), and IHMT-15130 (**9**). The electrophilic covalent warhead moieties are highlighted in yellow.

All compounds from the latter series maintain the strong inhibitory potency of **6** with analogue JS25 (**7**) proving to be the most potent one in a NanoBRET-based intracellular target engagement assessment (**7**: EC_50_ = 45 nM vs **6**: EC_50_ = 495 nM).^31^ A different series of covalent BMX inhibitors was discovered by Liang et al.^32^ These compounds featuring a 2,6-diaminopyrimidine scaffold are exemplified by CHMFL-BMX-078 (**8**) which is an irreversible type II BMX inhibitor (IC_50_ = 11 nM).^32^ While the compound showed promising selectivity for BMX over BTK (IC_50_ = 437 nM) and demonstrated good selectivity in a KINOMEscan assay, it did not significantly bind to BMX in the latter assay, limiting the conclusiveness of its kinome selectivity evaluation. Most recently, IHMT-15130 (**9**) was reported by Qi et al.^33^ as a potent and selective covalent BMX inhibitor, only inhibiting TEC family members and JAK3 significantly. Compound **9** displayed excellent inhibitory potency (*k*_inact_/*K*_I_ = 1.1 × 10^5^ M^–1^ s^–1^) with a strong *K*_I_ contribution (*K*_I_ = 25 nM) and a relatively fast rate of covalent bond formation (*k*_inact_ = 2.8 × 10^–3^ s^–1^), moderate selectivity vs BTK (∼10-fold IC_50_ difference) as well as downstream effects in cells and *in vivo*.^33^

In our previous work, we investigated the structure–activity relationship (SAR) of a new series of 7-azaindole-based BMX inhibitors.^34^ These compounds (exemplified by **10**, Figure 2) were derived from an earlier series of covalent JAK3 inhibitors and achieved selectivity by addressing the “beyond gatekeeper pocket” (and presumably BMX’s threonine gatekeeper, Thr489, itself) by an *N*-linked amide residue at the azaindole 5-position while targeting Cys496 through a *meta*-phenyl-linked acrylamide attached at the azaindole 3-position. While exhibiting low nanomolar potency against BMX and reasonable selectivity over most αD-1 cysteine kinases, these compounds had equipotent inhibitory activity for BTK.^34^ Here, we present the design, synthesis and extensive profiling of two follow-up series, which differ from our previous compounds by modifications at the residue that addresses the back pocket and at the linker/warhead moiety. These modifications led to distinct profiles, including improved enzymatic potency, and significantly enhanced selectivity for BMX over BTK.

**Figure 2:**
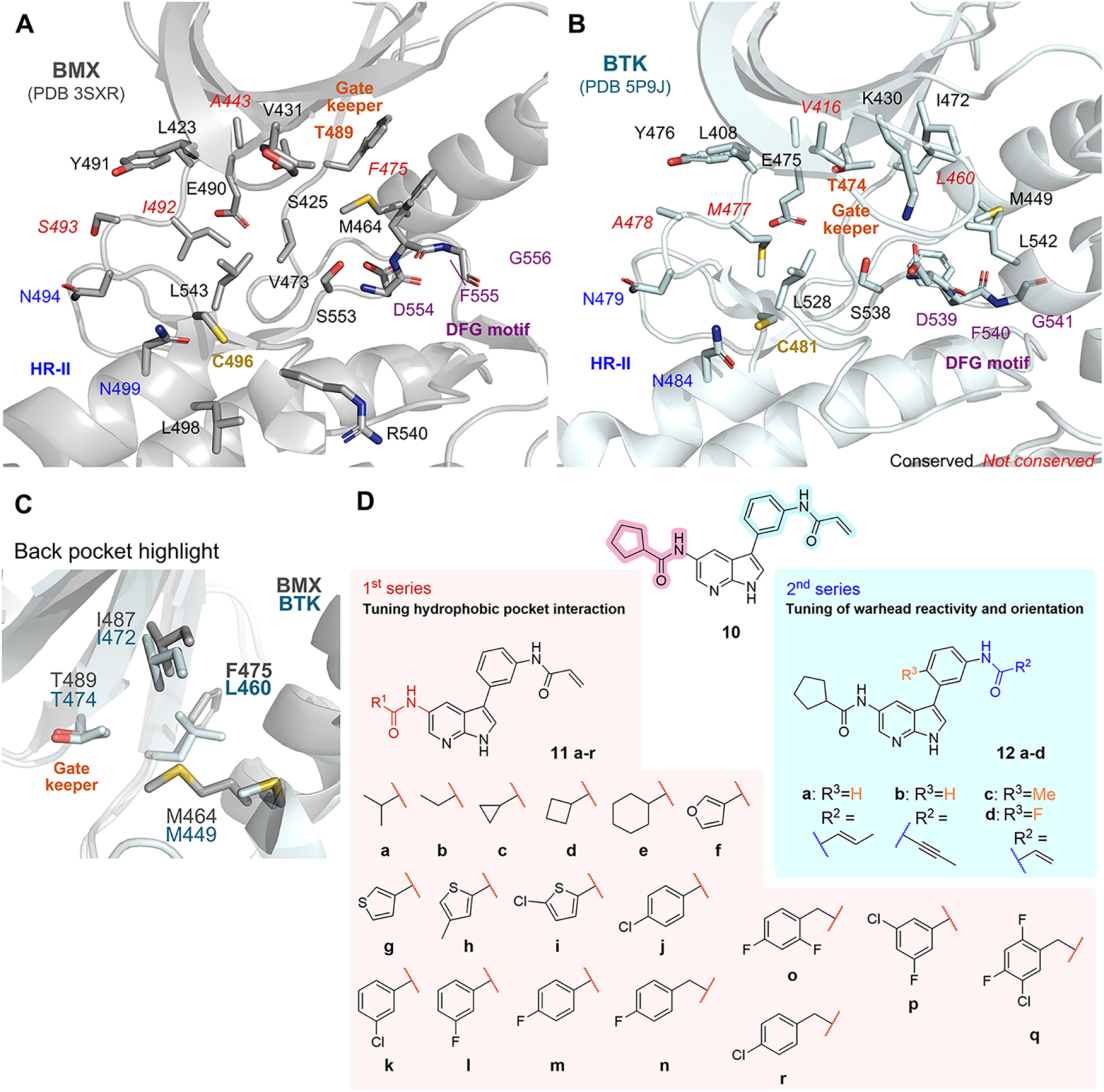
(A, B) Depictions of the kinase domains of BMX (A, PDB: 3SXR) and BTK (B, PDB: 5P9J). The different regions are colored in blue (HR-II), purple (DFG motif), the threonine gate keeper in orange and the αD-1 cysteine in yellow. The amino acids featured in our hypothesis to exploit the structural differences between BMX and BTK are colored in red. (C) Overlay of the backpockets of BMX (black) and BTK (petrol) highlighting Phe475 vs Leu460 in the same position, respectively. (D) The design of optimized 7-azaindole-derived BMX inhibitors (**11a**–**r** and **12a**–**d**).

## 2 Results

### 2.1 Molecular Design

In this study, our goal was to develop selective chemical probes for BMX. Building on the promising results of our previous work,^34^ we chose to explore the covalent inhibitor **10** as a starting point for further optimization. It features an acrylamide covalently targeting Cys496, a 7-azaindole core establishing two H-bonds to isoleucine (Ile) 492 and an inverse amide supposed to interact with Thr489 via an additional hydrogen bond. Two series of compounds with different modifications were designed and synthesized. The first series included compounds **11a**–**r** (Figure 2D) and was developed with the aim of tuning BMX selectivity by modulating the interaction with the “behind gatekeeper pocket (HR-I)” of BMX. To explore whether increased steric constrain or additional interactions including CH−π, π–π or halogen−π interactions with Phe475, which is part of the regulatory spine and replaced by a leucine (Leu460) in BTK (Figure 2A–C) could improve the selectivity against the latter kinase, various *N*-linked alkyl, (hetero)­aryl, and halo­(hetero)­aryl carboxamides were introduced in position 5 of the 7-azaindole system. The aryl rings were connected to the amide carbonyl with or without a methylene spacer, to allow for different orientations within the pocket (Figure 2). In this series, the *meta*-phenylacrylamide moiety of the parent compound was not modified to maintain the previously proven covalent interaction with the target kinase.^34^ The second, smaller series of BMX inhibitors focused on modulating selectivity of BMX vs BTK by exploring the effect of different warhead reactivities, geometry, and preorientation toward the αD-1 cysteine Cys496. Here, the reversible binding element of the previously discovered BMX/BTK dual inhibitor **10** was maintained but the acrylamide moiety was replaced with two related electrophilic warheads i.e., (*E*)-2-butenamide and 2-butynamide to afford compounds **12a** and **12b** (Figure 2). Alternatively, the warhead orientation was modified by introducing small substituents, such as methyl (**12c**) or fluorine (**12d**, both Figure 2), in the *ortho*-position of the linker phenyl ring. Notably, besides the effect of the expected out-of-plane torsion on the orientation of the warhead, these modifications were also expected to modulate warhead reactivity (i.e., a *para*-fluoro substituent would make the warhead slightly more reactive while a methyl group was expected to have the opposite effect) and impact noncovalent interactions with the target protein(s).

### 2.2 Synthesis

Compounds **11a**–**r** (Figure 2) were synthesized following a modified variant of our previous synthetic route.^34^ To this end, acrylamide-substituted aryl boronic ester **15** was synthesized starting from 3-bromoaniline (**13**). First, pinacol boronate **14** was obtained by cross-coupling under Miyaura conditions, using 2-dicyclohexylphosphino-2′,4′,6′-triisopropylbiphenyl (XPhos) and palladium acetate (Pd­(OAc)_2_) as catalyst system. Subsequently, the amino group of aniline **14** was acylated by acryloyl chloride to afford key intermediate **15**. In parallel, 5-nitro-7-azaindole (**16**) was selectively brominated in position 3 by using *N*-bromosuccinimide (NBS) to obtain the intermediate **17** which was protected at the indole nitrogen by introducing a 2-(trimethylsilyl)­ethoxymethyl (SEM) protecting group using SEM chloride (SEM-Cl) to afford intermediate **18**. Finally, the nitro group of **18** was reduced by treatment with zinc and ammonium formate to obtain key intermediate **19** with a free amino group (Scheme 1).

**Scheme 1:**
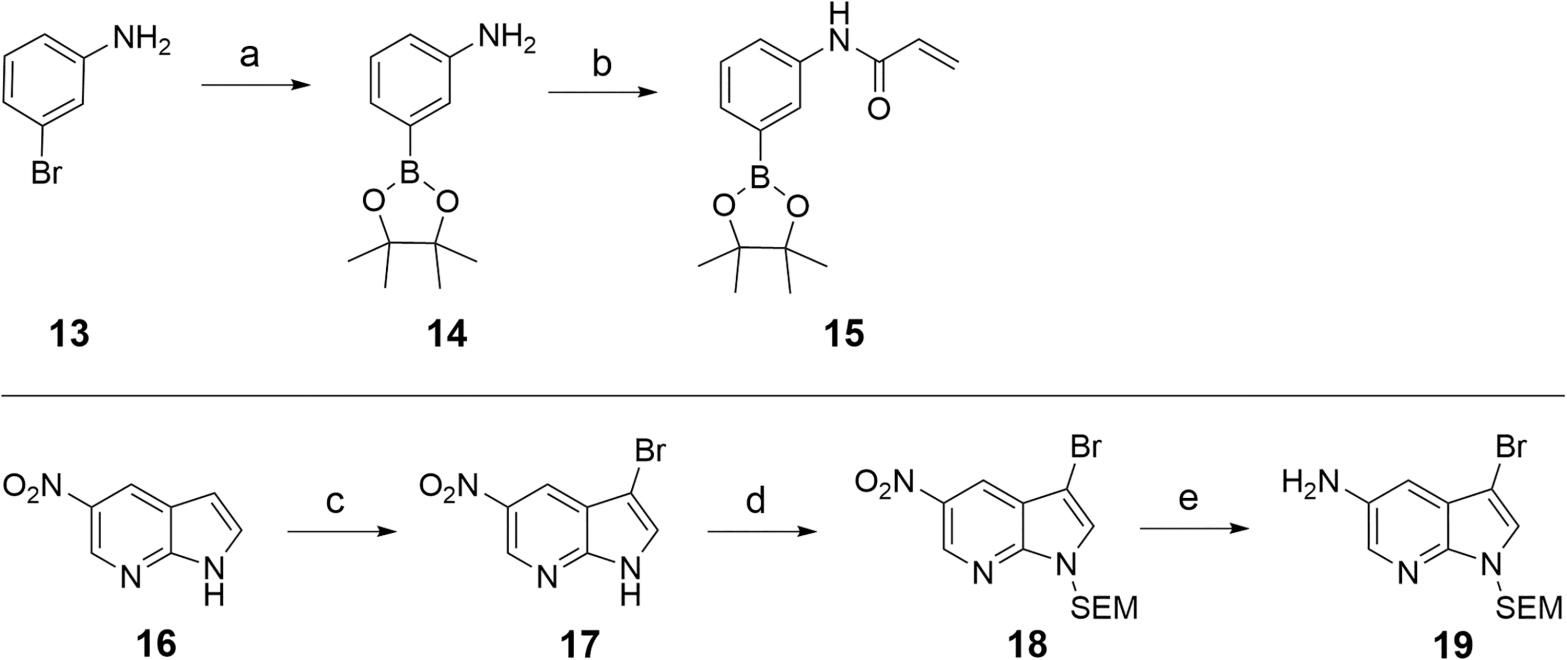
Synthetic Strategy for Obtaining the *N*-Acylated Pinacol Boronate (**15**) and SEM-Protected 3-Bromo-5-amino-7-azaindole (**19**)­^a^

a Reagents and conditions: (a) B_2_pin_2_, XPhos, Pd­(OAc)_2_, KOAc, dioxane, 90 °C, 24 h (55%); (b) acryloyl chloride, Et_3_N, DCM, 0 °C to rt, 1 h (65%); (c) NBS, DMF, rt, 3 h (85%); (d) NaH, SEM-Cl, DMF, 0 °C to rt, 22 h (99%); (e) Zn^0^, ammonium formate, EtOH, 50 °C, 22 h (66%).

In the following step, intermediate **19** (Scheme 2) was acylated on the free amine in position 5 by treatment with the appropriate acyl chloride to afford intermediates **20a**–**r**. Then, the boronate ester **15** was linked to the respective *N*-acylated intermediate (**20a**–**r**) under optimized Suzuki cross-coupling conditions compatible with the presence of the acrylamide, affording SEM-protected prefinal intermediates **21a**–**r**.^34^ Finally, the SEM-group was cleaved under acidic conditions by using trifluoroacetic acid (TFA) and following treatment with ammonia (NH_3_, 25%, aq.) in ethanol to cleave the initially obtained hemiaminal intermediate.

**Scheme 2:**
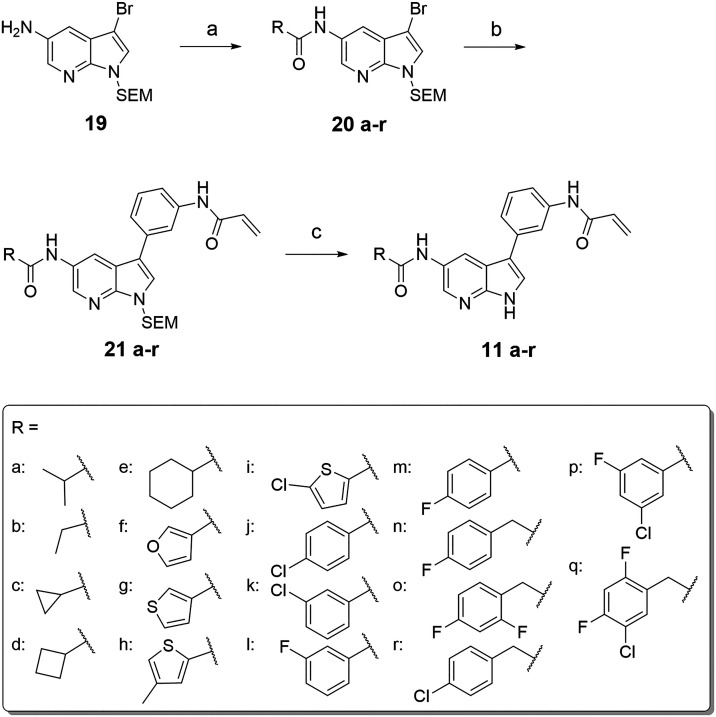
Synthetic Strategy for Obtaining Final Compounds **11a**–**r** ^a^

a Reagents and conditions: (a) corresponding acyl chloride, Et_3_N, DCM, rt, 1 h (51–99%); (b) **15**, *t*Bu_3_P Pd G3, K_2_CO_3_ (0.5 M, aq.), dioxane, rt or 80 °C, 22 h (35–82%); (c) (i) TFA, DCM, rt, 8 h; (ii) NH_3_ (25%, aq.), EtOH, rt, 24 h (36–98%).

The second series of compounds (**12a**–**d**, Figure 2) was obtained by synthesizing boronic esters **22**, **23**, **29**, and **30** as key intermediates (Scheme 3). 3-Bromoaniline (**13**), 3-bromo-4-methylaniline (**24**), and 3-bromo-4-fluoroaniline (**26**) were converted to the corresponding boronates **14** (see step **a** in Scheme 1), **27**, and **28** under Miyaura cross-coupling conditions, respectively. Then, they were acylated to afford **22**, **23**, **29**, and **30**. Intermediates **22** and **23** were synthesized under peptide coupling conditions using HATU/DIPEA or DCC/DMAP to activate (*E*)-2-butenoic acid and 2-butynoic acid, respectively (Scheme 3). In contrast, intermediates **29** and **30** were synthesized by acylating the pinacol boronates **27** and **28** with acryloyl chloride (Scheme 3).

**Scheme 3:**
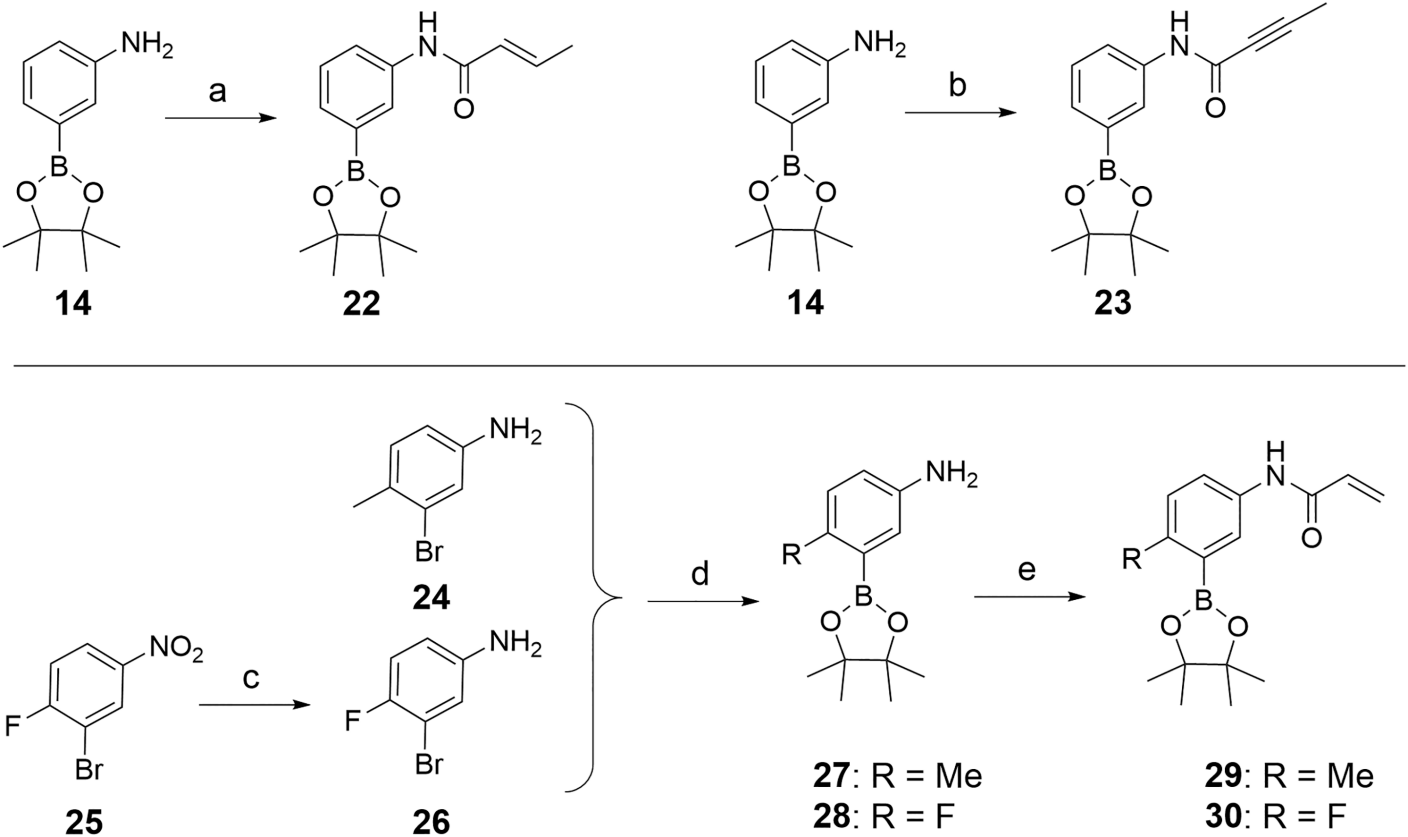
Synthetic Strategy for Obtaining the Acylated Pinacol Boronates **22**, **23**, **29**, **30** ^a^

a Reagents and conditions: (a) (*E*)-2-butenoic acid, HATU, DIPEA, THF, 0 °C to rt, 22 h (60%); (b) 2-butynoic acid, DCC, DMAP, DCM, 0 °C to rt, 22 h (47%); (c) SnCl_2_, HCl (37%, aq.), EtOH, 75 °C, 30 min (99%); (d) B_2_pin_2_, Xphos, Pd­(OAc)_2_, KOAc, dioxane, 90 °C, 24 h (43–44%); (e) acryloyl chloride, Et_3_N, DCM, 0 °C to rt, 1 h (60–91%).

In parallel, the previously synthesized intermediate **19** (Scheme 2) was derivatized in position 5 by acylation with cyclopentanecarbonyl chloride to afford the key intermediate **31** (Scheme 4). In turn, Suzuki cross-coupling reactions between **31** and the corresponding boronic esters **22**, **23**, **29**, **30** in the presence of *t*Bu_3_P Pd G3 and K_2_CO_3_ afforded SEM-protected prefinal compounds **32a**–**d**. Subsequently, the SEM protecting group was cleaved by TFA followed by NH_3_ (25%, aq.) treatment.

**Scheme 4:**
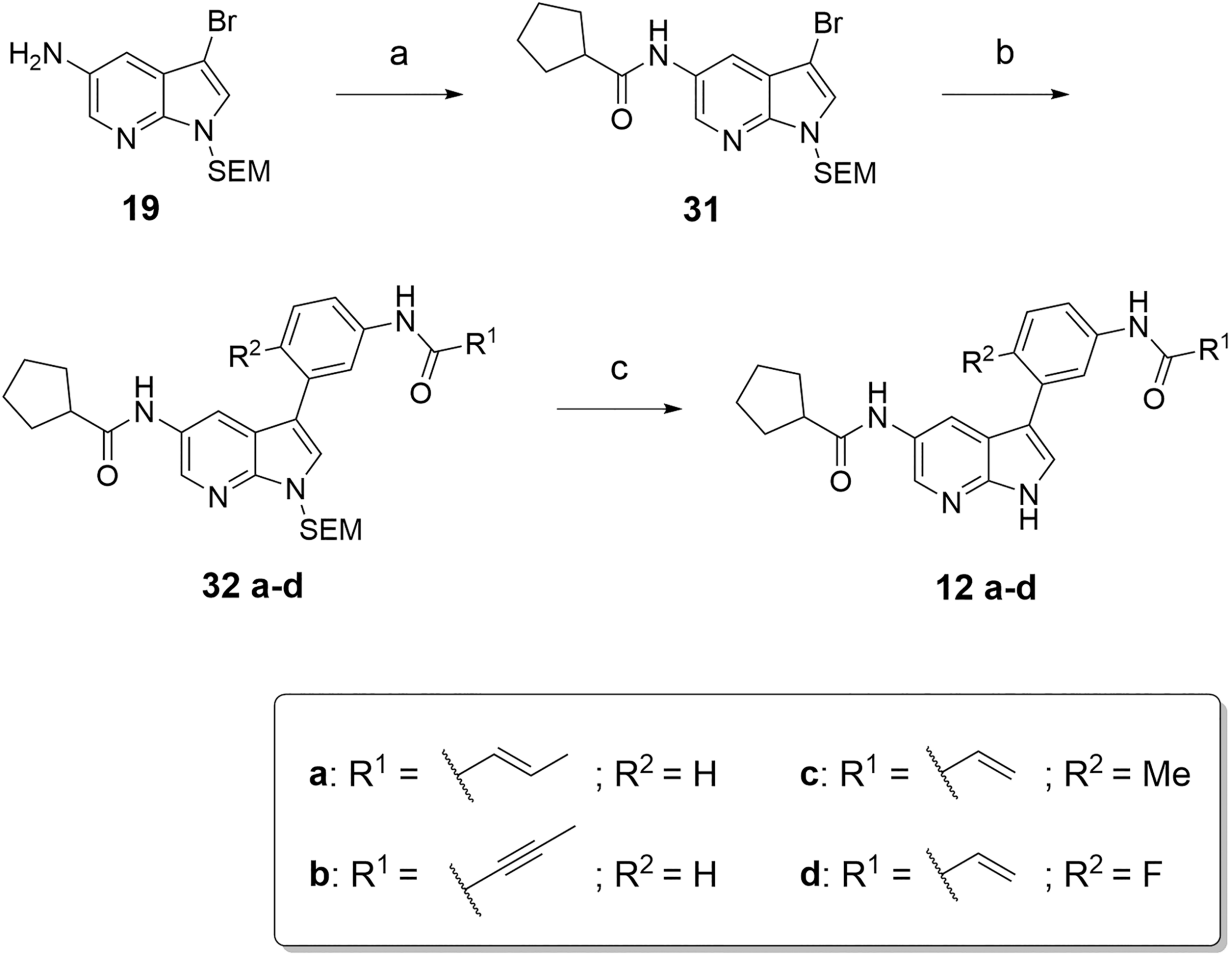
Synthetic Strategy for Obtaining Final Compounds **12a**–**d** ^a^

a Reagents and conditions: (a) cyclopentanecarbonyl chloride, Et_3_N, DCM, rt, 1 h (82%); (b) corresponding boronic ester (**22**, **23**, **29**, and **30**), *t*Bu_3_P Pd G3, K_2_CO_3_ (0.5 M, aq.), dioxane, rt or 80 °C, 22 h (27–56%); (c) (i) TFA, DCM, rt; (ii) NH_3_ (25%, aq.), EtOH, rt, 24 h (59–89%).

To examine the impact of the covalent mode-of-action, which we confirmed for the parent compounds by mass spectrometry,^34^ similar noncovalent compounds **33** and **34** bearing a propionamide instead of an acrylamide moiety were synthesized. Here, **11i** and **12c** were chosen for the synthesis of corresponding nonreactive propionamide analogues as they belong to structurally different series and showed a favorable selectivity for BMX vs BTK in the NanoBRET assay (*vide infra*). Acylation of the appropriate boronic acid or ester (**35**, **27**) using propionyl chloride afforded boronic acid derivatives (**36**, **37**, Scheme 5). They were coupled with 7-azaindole intermediates (**20i**, **31**) in Suzuki cross-coupling reactions to afford **38** and **39** which were SEM-deprotected in a last step to afford **33** and **34**.

**Scheme 5:**
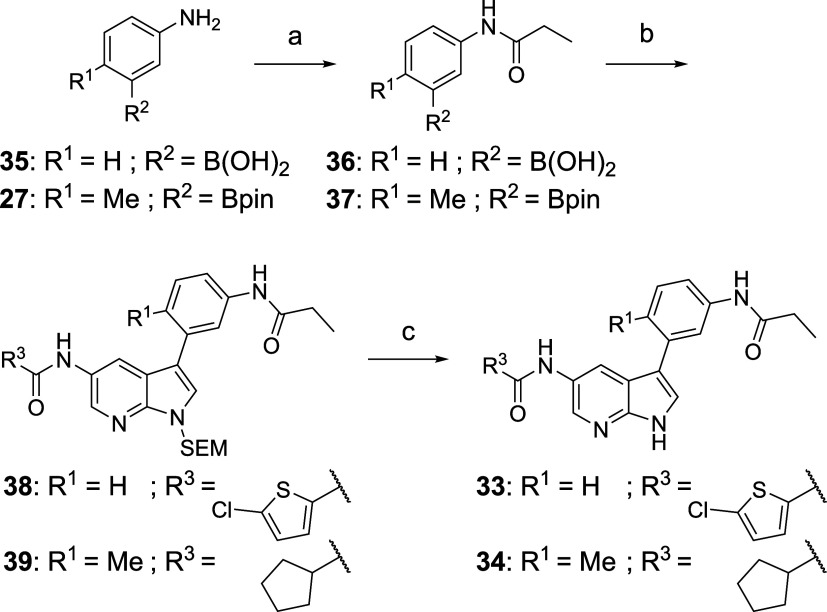
Synthetic Strategy for Obtaining Noncovalent Control Compounds **33** and **34** ^a^

a Reagents and conditions: (a) boronic acid (**35**) or ester (**27**), propionyl chloride, Et_3_N, THF, 0 °C to rt, 1 h (71%, 55%); (b) corresponding boronic acid or ester (**36**, **37**) and azaindole (**20i**, **31**), *t*Bu_3_P Pd G3, K_2_CO_3_ (0.5 M, aq.), dioxane, 60 °C, 22 h (99%, 86%); (c) (i) TFA, DCM, rt; (ii) NH_3_ (25%, aq.), EtOH, rt, 24 h (63%, 85%).

### 2.3 Biochemical Evaluation

For a first evaluation of the binding of compounds **11a**–**r** and **12a**–**d**, a DSF (differential scanning fluorimetry)-based thermal shift assay (Δ*T*_m_) was employed.^35^ The compounds were assessed at 10 μM concentration against BMX and its closest relative BTK as well as MKK7, all featuring an equivalently positioned αD-1 cysteine (Figure 3). Most of the tested compounds displayed stronger thermal stabilization of BMX than BTK and MKK7, suggesting a higher binding affinity for BMX. However, substantial variations in thermal shifts were observed. For instance, compound **11b**, showed similar stabilization for BMX and MKK7 reaching Δ*T*_m_ values in the range of 10–11 °C while several other compounds stabilized BMX in the same Δ*T*_m_ range and exhibited only very weak stabilization of MKK7. Similarly, while all compounds showed stronger stabilization of BMX compared to BTK, some compounds such as **11n** or **11o** showed a pronounced difference while the shifts obtained for other compounds like **12d** suggested a more balanced profile.

**Figure 3:**
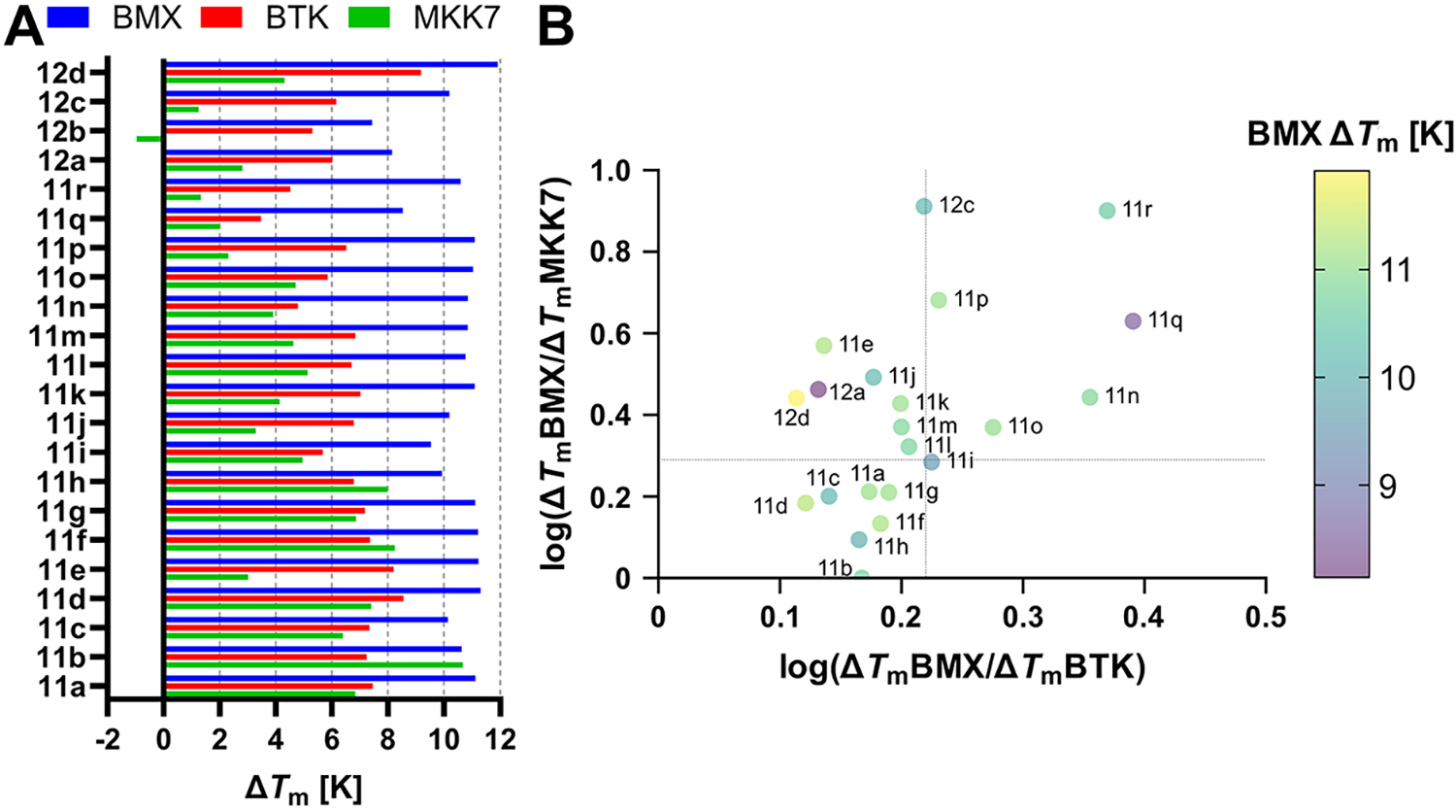
(A) Thermal stabilization (Δ*T*_m_) [K] of BMX (blue), BTK (red) and MKK7’s (green) kinase domains by compounds **11a**–**r** and **12a**–**d** (see the Supporting Information, Table S1 for exact values). (B) Logarithmic depiction of the Δ*T*_m_ ratios. Logarithmic ratios were used to linearize the data and improve visualization of selectivity differences across the compound series. The dashed lines indicate ratio thresholds of log(1.65) (*x*-axis) and log(1.93) (*y*-axis) used for compound preselection along with structural differences.

For validation in a complementary assay, all compounds (**11a**–**r** and **12a**–**d**) were screened in an activity-based assay format at a fixed time point against the recombinant BMX kinase. Additionally, based on Δ*T*_m_ BMX/BTK and Δ*T*_m_ BMX/MKK7 data (Table S1), and using the diversity of structural features as additional selection criteria, compounds **11i**, **11n**, **11p**, **11q**, **11r**, and **12c** were selected to be additionally tested against BTK. The selected compounds showed at least a Δ*T*_m_ ratio BMX/BTK ≥ 1.65 and a Δ*T*_m_ ratio BMX/MKK7 ≥ 1.93. Moreover, with respect to their structural characteristics, all but **12c** possess a bulky halogenated aryl substituent amide-linked at position 5 of the 7-azaindole scaffold that may form additional interactions with the HR-I of BMX. In contrast, compound **12c** features an *ortho*-methyl substituent on the front-pocket phenyl ring linked to the 3-position of the azaindole that facilitates an out-of-plane torsion thereby altering the preorientation of the warhead. Thus, compound **12c** may offer a possibility to discriminate between BMX and BTK through minor differences in the orientation and trajectory of the αD-1 cysteines present in both kinases. The apparent IC_50_ values of **11a**–**r** and **12a**–**d** in the enzymatic assay in comparison to the parent compound **10** (IC_50_ = 2.2 nM) as well as IC_50_’s of nonreactive analogues **33** and **34** are shown in Table 1.^34^

**Table 1:**
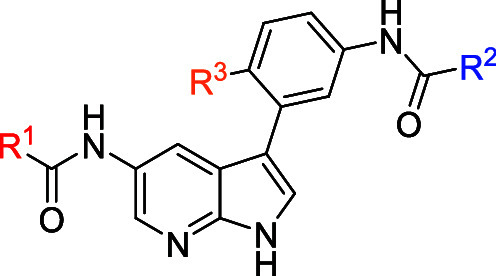

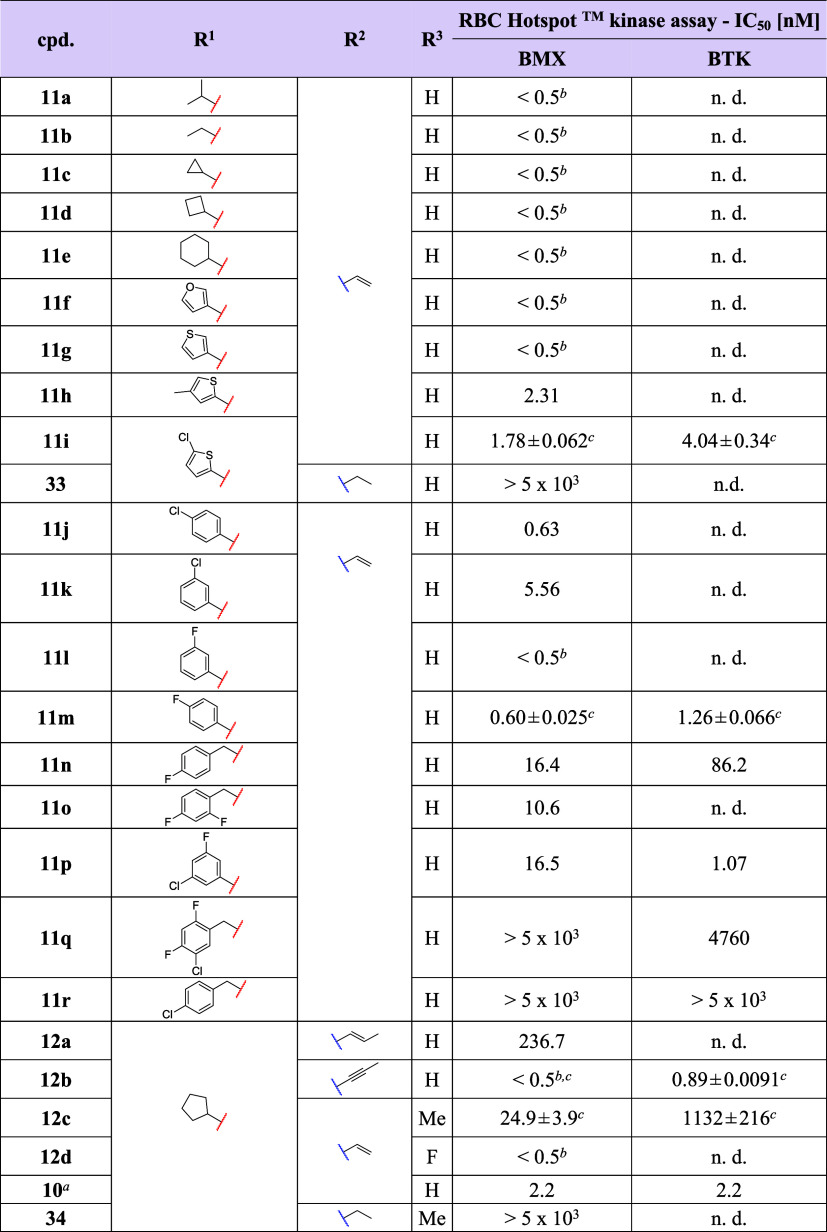
IC_50_ Evaluation of Compounds **11a**–**r** and **12a**–**d** against BMX^d^

a IC_50_ of the hit compound published by Forster et al. determined in the same assay.^34^

b IC_50_ value smaller than the lowest concentration tested (0.5 nM).

c IC_50_ values were redetermined in duplicate after an initial evaluation in singlicate.

d Pre-selected compounds were also tested against BTK. IC_50_ values were determined in a radiometric format (Reaction Biology HotSpot^36^) starting at 5 μM with [ATP] = 10 μM. n.d.: not determined.

Interestingly, all putative irreversible compounds showed at least double-digit nanomolar IC_50_ values for BMX with the exception of compounds **11q** and **11r** (Table 1) featuring a methylene-linked haloaryl moiety at the carboxamide (R^1^) and crotonamide derivative **12a**. To our delight, compounds **11a**–**j**, **11l**, and **11m** with different (cyclo)­alkyl and (halogenated) aryl or heteroaryl residues at R^1^ demonstrated very promising IC_50_ values in the single-digit nanomolar range, some even below the lowest concentration tested (0.5 nM), whereas noncovalent binding control compounds **33** and **34** exhibited IC_50_ values over 5 μM. This strong drop of activity compared to their reactive counterparts is in line with a covalent mode-of-action. Notably, and as mentioned before, covalent interaction has already been confirmed for compound **10** and related compounds from our parent series, where several compounds completely labeled BMX at 1.5 -fold molar excess after 30 min at 4 °C.^34^

In compounds **12a**–**d** (Table 1) the parent cyclopentyl moiety at R^1^ was retained but changes were made at the front pocket residue and covalent warhead. The presence of an (*E*)-but-2-enamide warhead (**12a**) resulted in weaker BMX inhibition with respect to parent acrylamide **10** while the 2-butynamide bearing **12b** showed an improved IC_50_ value (<0.5 nM). Attempts to alter warhead orientation through the introduction of methyl (**12c**) and fluorine (**12d**) substituents at the *ortho*-position on the phenyl ring maintained substantial BMX inhibition. The more potent among these compounds was fluoro analogue **12d** which showed again subnanomolar potency while potency of the methyl analogue **12c** was reduced (IC_50_ ∼ 25 nM). Notably, besides orientation, the fluoro substituent may also increase the reactivity of the acrylamide by reducing electron density in the aryl ring, which may drive a faster inactivation (*k*_inact_) step while the methyl group is expected to have an opposite effect.

Testing of compounds **11i, 11n**, **11p**, **11q**, **11r**, and **12c** against BTK revealed that the 4-fluorobenzyl substituted (R^1^) compound **11n** showed a 5-fold higher potency on BMX while the chlorothiophene analogue **11i** maintained a 2-fold difference. Chlorobenzyl analogue **11r** and trihalogenated analogue **11q** showed no pronounced inhibition of either BMXor BTK. In contrast to the Δ*T*_m_ data (BMX/BTK = 1.7, Table S1), 2-chloro-4-fluorophenyl analogue **11p** displayed significantly higher inhibitory potency for BTK compared to BMX. However, installation of an *ortho*-methyl group at the front pocket aryl linker (**12c**) while maintaining the cyclopentyl back pocket residue of the parent compound **10** shifted selectivity toward BMX. Compound **12c** exhibited nanomolar IC_50_ values against BMX with micromolar inhibition of BTK (Table 1), which is also consistent with the trend observed in the Δ*T*_m_ results.

To corroborate the results obtained on isolated enzymes, all compounds were tested for BMX and BTK target engagement in HEK293T cells using a bioluminescence resonance energy transfer (NanoBRET) assay. Notably, it has recently been highlighted that cellular selectivity profiles may significantly differ from such determined in enzyme assays.^37^ Despite most of the compounds’ IC_50_ values being around the single-digit nanomolar range in the enzymatic assay, only cycloalkyl analogues (R^1^) **11d** and **11e**, furyl analogue **11f**, chlorothiophene **11i**, and fluorophenyl analogue **11m** maintained nanomolar EC_50_ values in the NanoBRET assay (Table 2). This may be due to their more drug-like size and polarity compared to the more lipophilic and larger analogues. Poorly performing compounds **11j**–**l** and **11n**–**r** all possess a bulky halogenated phenyl residue which potentially leads to efflux or poor intracellular distribution. A possible explanation for **11m** being an outlier in this argumentation could be a slight increase in polarity due to the *para*-fluoro substitution compared to unsubstituted phenyl derivatives. Another element may be the physiological concentration of ATP present in the NanoBRET experiment which is much higher than in the biochemical assay (∼1.5 mM vs 10 μM) leading to stronger ATP competition and therefore stronger *K*_I_-dependent discrimination. Among the aforementioned analogues, **11i** and **11m** showed the best selectivity windows for BMX over BTK (∼20-fold and ∼95-fold, respectively). Comparison between the cellular and enzymatic assay also revealed that **11n** and **12c** maintained a good selectivity against BTK (∼10-fold and ∼8-fold, respectively).

**Table 2:**
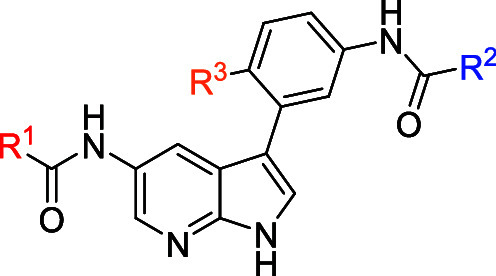

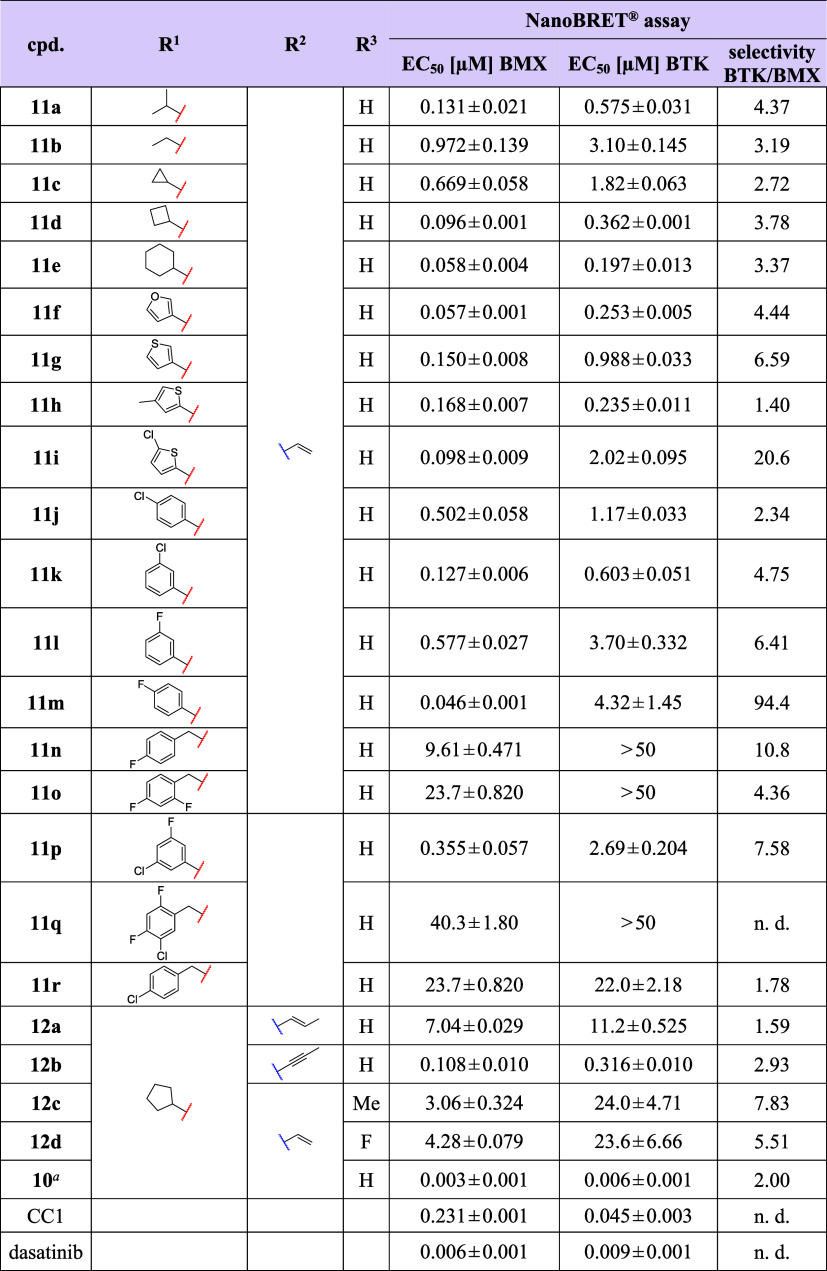
EC_50_ Evaluation of Compounds **11a**–**r** and **12a**–**d** on BMX and BTK by NanoBRET Assays^b^

a IC_50_ of the hit compound published by Forster et al.^34^

b Experiments were performed in HEK293T cells as triplicates and repeated at least two times.

In order to explore broader kinome selectivity, **11i**, **11m**, **11n**, **12b**, and **12c** were selected for a thermal shift screen against approximately 100 kinases (see Table S2 for all Δ*T*_m_ values). Compounds **11i**, **11m**, and **11n** were chosen, since they showed the highest BTK/BMX selectivity in cells. Compounds **12b** and **12c** were additionally selected as they may offer learnings about the influence of the nature or (pre)­orientation of the warhead on selectivity. In our DSF selectivity assay, compound **11m**, which had excellent BMX vs BTK selectivity in the NanoBRET assay (BTK/BMX = 94.4) demonstrated a Δ*T*_m_ > 4 K on 5 kinases (Table S2). A similar trend was observed for compounds **11i** (selectivity BTK/BMX = 20.6, Table 2) that showed a Δ*T*_m_ > 4 K on 2 additional kinases (off-targets and the degree of their thermal stabilization are shown in Figure 4) whereas **11n** (selectivity BTK/BMX = 10.8) showed none. The 2-butynamide compound **12b** (selectivity BTK/BMX = 2.9) demonstrated a Δ*T*_m_ > 4 K on 16 kinases suggesting a significantly decreased overall selectivity (Table 3). Conversely, the modification of the warhead orientation in **12c** (acrylamide with *ortho*-methyl at the phenyl spacer) showed somewhat improved selectivity with a significant thermal stabilization > 4 K observed for 2 additional kinases.

**Table 3:** Fraction of Stabilized Kinases within a Panel Containing 104 Enzymes^a^

cpd.	Fraction of Kinases stabilized > 4 K [%]	Δ*T*_m_ > 4 K	Δ*T*_m_ < 4 K
**11i**	3	3	101
**11m**	5	5	99
**11n**	1	1	103
**12b**	15	16	88
**12c**	3	3	101
**STAU**	77	80	24

a The screen was performed using a differential scanning fluorimetry (DSF) assay format. Thermal shift values were determined as the offsets between curves of inhibitor and negative control (DMSO). The *pan*-kinase inhibitor staurosporine was used as a positive control.

**Table 4:**
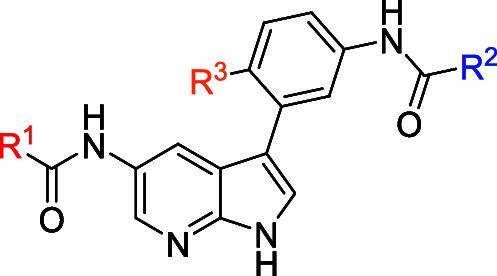

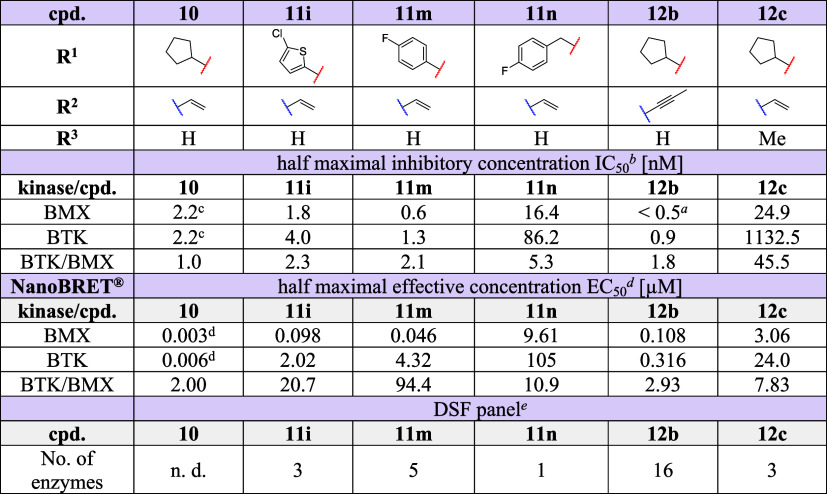
Summary of the BMX and BTK Binding and Inhibition Profiles for a Relevant Set of Compounds

a IC_50_ < than the lowest concentration tested (0.5 nM).

b IC_50_ values measured the RBC Hotspot assay.

c Data obtained in our previous study using the same assay.^34^

d BMX and BTK cellular target engagement determined by a NanoBRET assay in HEK293T cells.

e DSF panel investigating stabilization of ∼100 kinases. Number of enzymes with a Δ*T*_m_ > 4 K are shown.

**Figure 4:**
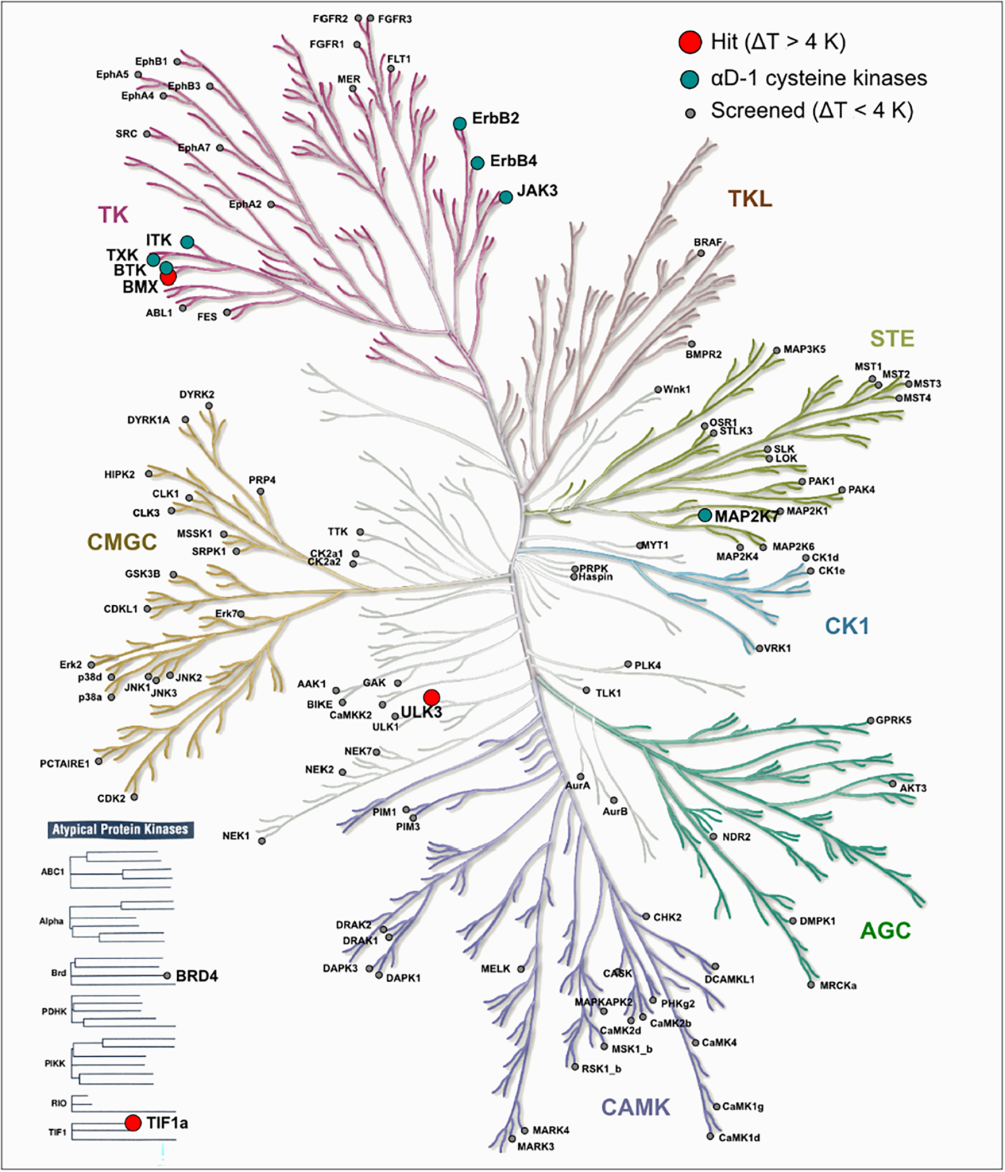
Depiction of the off-targets of compound **11i**. This is exemplary for the differential scanning fluorimetry (DSF) screening assay of compounds **11i**, **11m**, **11n**, **12b**, and **12c** (see the Supporting Information, Table S2). Thermal shift values were determined as the offsets between curves of inhibitor and negative control (DMSO). The *pan*-kinase inhibitor staurosporine was used as a positive control. The red dots represent kinases where **11i** showed a thermal shift Δ*T*_m_ > 4 K. The gray dots represent the tested but not significantly affected kinases (Δ*T*_m_ < 4 K). The depiction also features αD-1 cysteine containing kinases (dark cyan dots) which are possible off-targets of **11i**. The figure was prepared with KinMap^38^ (more details can be found in the Supporting Information). A summary of the profiles determined for key compounds is provided in Table 4.

A complementary activity assay (tested at a fixed concentration of 250 nM, Table 5) was conducted on an additional small kinase panel, comprising all αD-1 cysteine containing kinases except BMX and BTK, for which IC_50_ values had already been obtained in the same assay format. This panel generally showed good selectivity among these kinases. The most selective compounds were *ortho*-methyl compound **12c** and the butynamide **12b**, highlighting the role of warhead orientation. While **12c** only slightly affected BLK and TXK, compound **12b** had significant off-target activity on the TEC family member TXK and to a lesser extent TEC but did not affect other kinases in the set. Chlorothiophene **11i** showed significantly reduced TEC/TXK inhibition, however, at the expense of stronger inhibitory activity on HER4; a trend that was even more pronounced for 4-fluorophenyl analogue **11m**. Notably, none of the compounds had a significant effect on JAK3, MKK7 and ITK, which differ from the other kinases in the set through their gatekeeper residue (methionine in JAK3/MKK7, phenylalanine in ITK, threonine in all others).^34^

**Table 5:**
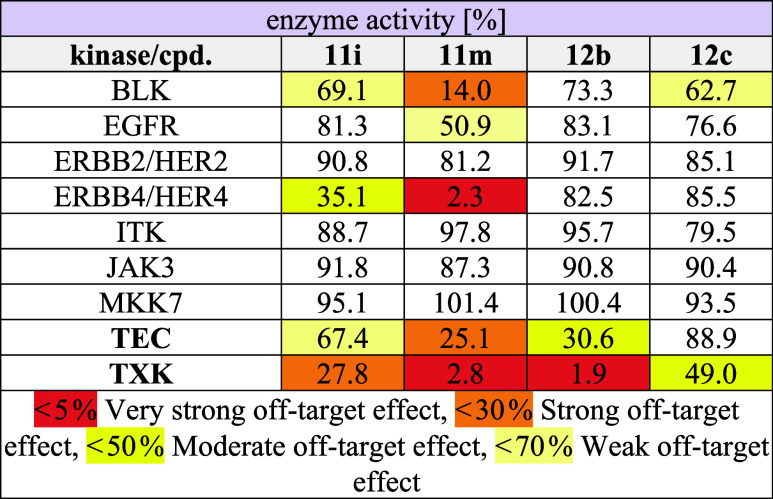
Radiometric Hotspot Assay by RBC Determining the Residual Activity of **11i**, **11m**, **12b**, and **12c** against the Other αD-1 Cysteine Containing Kinases at 250 nM with [ATP] = 10 μM^a^

a The enzyme activity was measured as duplicates and calculated in relation to a DMSO control.

Overall, we found compound **11i** to feature the most compelling profile in the aforementioned assays. Therefore, this compound was selected for more extensive selectivity profiling in a NanoBRET screen against 192 kinases (see SI Table S3). In line with previous results, it was found to solely engage TXK apart from BMX with over 60% (Figure 5) thereby confirming its selectivity.

**Figure 5:**
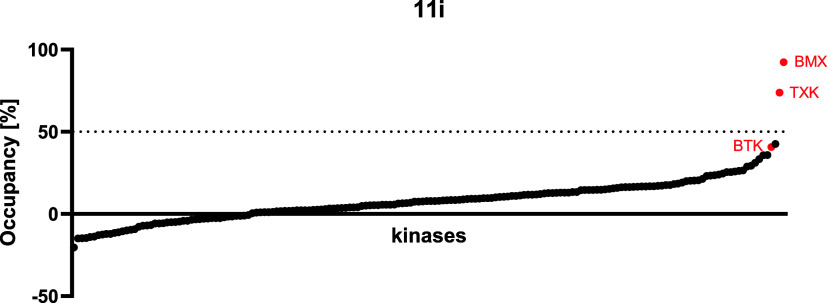
Cellular target occupancy [%] of **11i** at 1 μM in a NanoBRET kinase panel against 192 kinases. Details can be found in Supporting Information (Table S3).

### 2.4 Evaluation of Binding Kinetics and Validation of the Covalent Modification

The formation of a covalent bond between an irreversible inhibitor and a kinase of interest is time-dependent and involves a two-step mechanism: an initial equilibrium binding event, followed by covalent bond formation. As potency shifts over time, covalent inhibitors are best characterized by the time-independent inactivation efficiency rate constant *k*_inact_/*K*_I_ defining the overall efficiency of the covalent binding process.^17^ Here, the constant *K*_I_ describes the concentration at which covalent inactivation occurs at the half-maximal rate, whereas the maximal rate (i.e., at target saturation with the noncovalently bound inhibitor) is defined by the first-order rate constant *k*_inact_.^17,39,40^ Therefore, we evaluated the kinetic parameters of key compounds **11i**, **12b**, and **12c** using the AssayQuant PhosphoSens® platform. Generally, *K*_I_ values in the upper nanomolar to low micromolar range were observed (see Table 6), which recapitulates our previous results obtained with nonreactive analogues in end point enzyme assays. The BMX *k*_inact_/*K*_I_ values of **11i** (5.28 × 10^4^ M^–1^ s^–1^) and **12b** (5.14 × 10^4^ M^–1^ s^–1^) get close to the FDA-approved inhibitor acalabrutinib (**2**, 2.41 × 10^5^ M^–1^ s^–1^ on BTK)^41^ indicating a drug-like range. Acalabrutinib only shows a 4-fold difference in *k*_inact_/*K*_I_ compared to **11i**. Although its *K*_I_ is 20-fold lower than the one determined for **11i** (8.70 × 10^–9^ M vs 1.58 × 10^–7^ M), it is 4-fold slower in covalent bond formation than **11i** (*k*_inact_: 2.1 × 10^–3^ s^–1^ vs 8.3 × 10^–3^ s^–1^). However, it should be considered that acalabrutinib contains a 2-butynamide warhead which is expected to be less reactive than the acrylamides used here. In contrast, zanubrutinib (**3**), a BTK inhibitor which possesses an acrylamide warhead features a *K*_I_ value (1.32 × 10^–7^ M) in the same range as our compounds **11i** and **12b** but it has a ∼10-fold higher *k*_inact_/*K*_I_ (5.80 × 10^5^ M^–1^ s^–1^) due to its faster bond-forming kinetics (*k*_inact_ = 7.66 × 10^–2^ s^–1^).^24^ Interestingly, the 10-fold drop of inactivation efficiency in **12c** (4.36 × 10^3^ M^–1^ s^–1^) compared to **11i** and **12b** is driven by a higher *K*_I_ while *k*_inact_ was fairly constant within the series (∼9 × 10^–3^ s^–1^) indicating that the orientation imposed via the introduction of a methyl substituent in *ortho*-position of the linker aryl ring did not hamper covalent bond formation.

**Table 6:** Kinetic Studies of Key Compounds **11i**, **12b**, and **12c** Determined Using the PhosphoSens CSox-Sensor Platform by AssayQuant^a^

cpd.	**11i**	**12b**	**12c**
*k*_inact_ [s^–1^]	8.3 × 10^–3^ ± 4.0 * 10^–4^	9.5 × 10^–3^ ± 6.7 * 10^–4^	8.5 × 10^–3^ ± 4.8 * 10^–4^
*K*_I_ [M]	1.58 × 10^–7^ ± 8.86 * 10^–9^	1.86 × 10^–7^ ± 1.50 * 10^–8^	1.95 × 10^–6^ ± 0.127 * 10^–7^
*k*_inact_/*K*_I_ [M^–1^ s^–1^]	5.28 × 10^4^ ± 570	5.14 × 10^4^ ± 750	4.36 × 10^3^ ± 52

a The reaction progress curves were measured in a continuous assay format at 24 different inhibitor concentrations.

To further validate the covalent modification of BMX with our compounds, we investigated covalent adduct formation between BMX and **11i** by intact protein MS which showed 100% labeling at a 1:2 molecule-to-compound ratio after 90 min incubation time at 4 °C (Figure S3).

### 2.5 Proposed Binding Mode Generated by Molecular Modeling

To expand our previous model of covalent BMX inhibition and to enable future optimizations, we sought for a more comprehensive model rationalizing BTK/BMX inhibition and selectivity. While our attempts to obtain X-ray crystal structures remained futile, we generated a sophisticated computational model for BMX (based on PDB 6I99, chain B—see methods for more details) and BTK (PDB 5P9J) on the basis of published high-resolution crystal structures with covalently bound ligands, followed by covalent docking (compounds **10**, **11i**, and **11m**) and locally minimizing the structure subsequently. Afterward, it was submitted to all-atom molecular dynamics (MD) simulations (5 × 1 μs per compound). Those MD trajectories were compared with simulations of the hit compounds **11i** and **11m** in their prereactive binding mode (i.e., noncovalently bound), in which one can derive a more accurate binding energy estimation.

The modeled binding mode suggested that the compounds interacted with a single residue from the hinge region via two hydrogen bonds (Met477-BTK and Ile492-BMX, >90% of the analyzed simulation time for all compounds, Figure 6A–F), while being further stabilized by an array of hydrophobic contacts. Nonsurprisingly, the compounds displayed no relevant interactions with the DFG motif.

**Figure 6:**
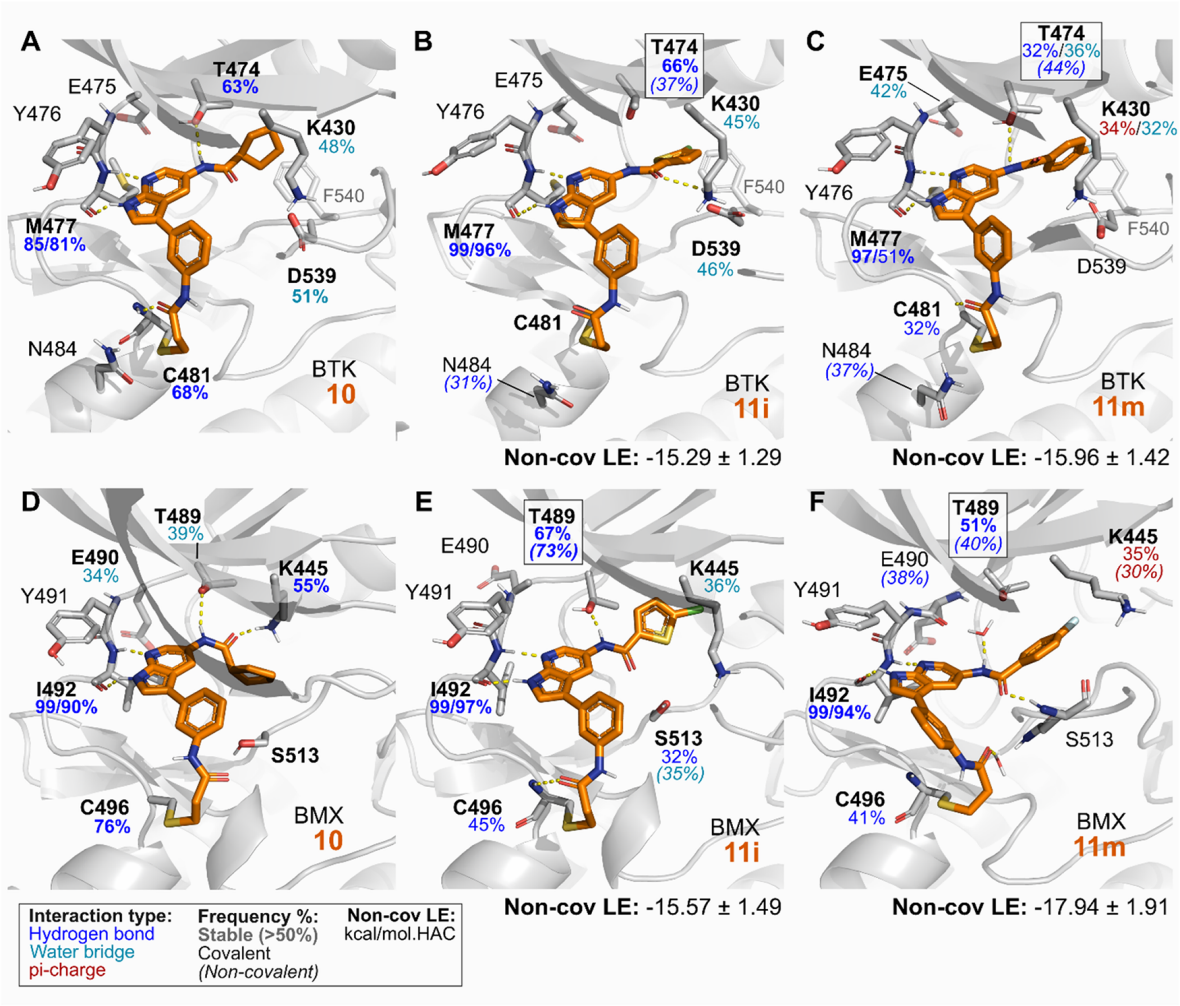
Potential binding mode of compounds **10** (A, D), **11i** (B, E) and **11m** (C, F) within the binding pocket of BTK (A–C) and BMX (D–F). Relevant frames from the MD simulation (derived from cluster analyses), display the potential binding modes of the compounds. For each compound, the interaction frequency of hydrogen bond, water bridge and π-charge interactions, along the analyzed 5 μs (5 × 1 μs) is depicted in blue, turquoise and red, respectively. Bold interactions were stable over more than 50% of the analyzed simulation, and italicized values were derived from noncovalently bound simulations. Free energy binding calculation by MM-GBSA calculated along the trajectory (kcal/mol normalized by the Heavy Atoms Count, HAC) are depicted below the panels.

The presence of either a conformationally more flexible cycloalkyl substituent (**10**) or aromatic rings (**11i** and **11m**) at the carboxamide (R^1^) drive a favorable interaction with the behind gatekeeper pocket (HR-I) of BMX and to a lesser extend to the HR-I of BTK. The amide group establishes stable polar contacts with the gatekeeper threonine’s side-chain (>50% in all our simulated compounds, Table S4) and, specifically for BTK, an intermittent water-mediated interaction with the catalytic lysine (Lys430, ∼30%) was observed. Compound **11i** displays a higher number of defined interactions with BMX than **11m**. However, MM-GBSA predicted binding energy supports a higher affinity between BMX and **11m** (Figure 6A–F and Tables S4, S5).

The initial hypothesis that the different R^1^ substituents would display additional π–π or halogen−π interactions with the HR-I residues, especially Phe475 in BMX was based on previous docking models.^34^ However, this was not supported by our MD calculations. Interestingly, previous shorter simulations of BMX with covalent inhibitor JS24 (500 ns with the original cocrystallized ligand from 6I99^31^) supports a one-hydrogen bond hinge interaction mode (in contrast to what is suggested by the corresponding X-ray structure), along with a stable, but dynamic, interactions with the catalytic Lys445. In contrast, our models display two hydrogen bond interactions of our compounds with the same hinge amino acid.

Compound **12c** retains most of these key interactions, including those with the gatekeeper threonine residue and the hinge region, in both BMX and BTK (Figure 7A,B). However, the torsional profile analysis of compound **12c** and **10** (Figure 7C) enabled an additional observation on the former: *ortho*-methylation of the linker phenyl moiety has been shown to impact the selectivity of the compound toward BMX. Notably, when compound **12c** is bound to BMX, a distinct peak emerges in the 2D bar chart at approximately −45°, in contrast to compound **10**, which exhibits a bimodal distribution with major peaks at −45° and −135° in the 2D representation (Figure 7C). These 2D bar charts effectively capture torsional probabilities across various angles, providing insight into their prevalence throughout the simulation. This finding implies that the *N*-(4-methylphenyl)­acrylamide moiety aligns more favorably within the BMX binding pocket, achieving better shape complementarity compared to its unmethylated counterpart compound **10** (Figure 7A–C) in comparison to BTK. This favorable binding mode likely contributes to the experimentally observed increased selectivity toward BMX. Additionally, different preorientations of the acrylamide of **12c**, calculated using QM-conformational scans, show a stronger preference for conformations where this group points to the direction of the target cysteine residue (namely conformation A, Figure 7D), i.e., 65.5% of the calculated conformations in comparison to ∼50% for compounds **11i** and **11m** (Supporting Information, Figure S1 depicts all clustered conformations and their distributions).

**Figure 7:**
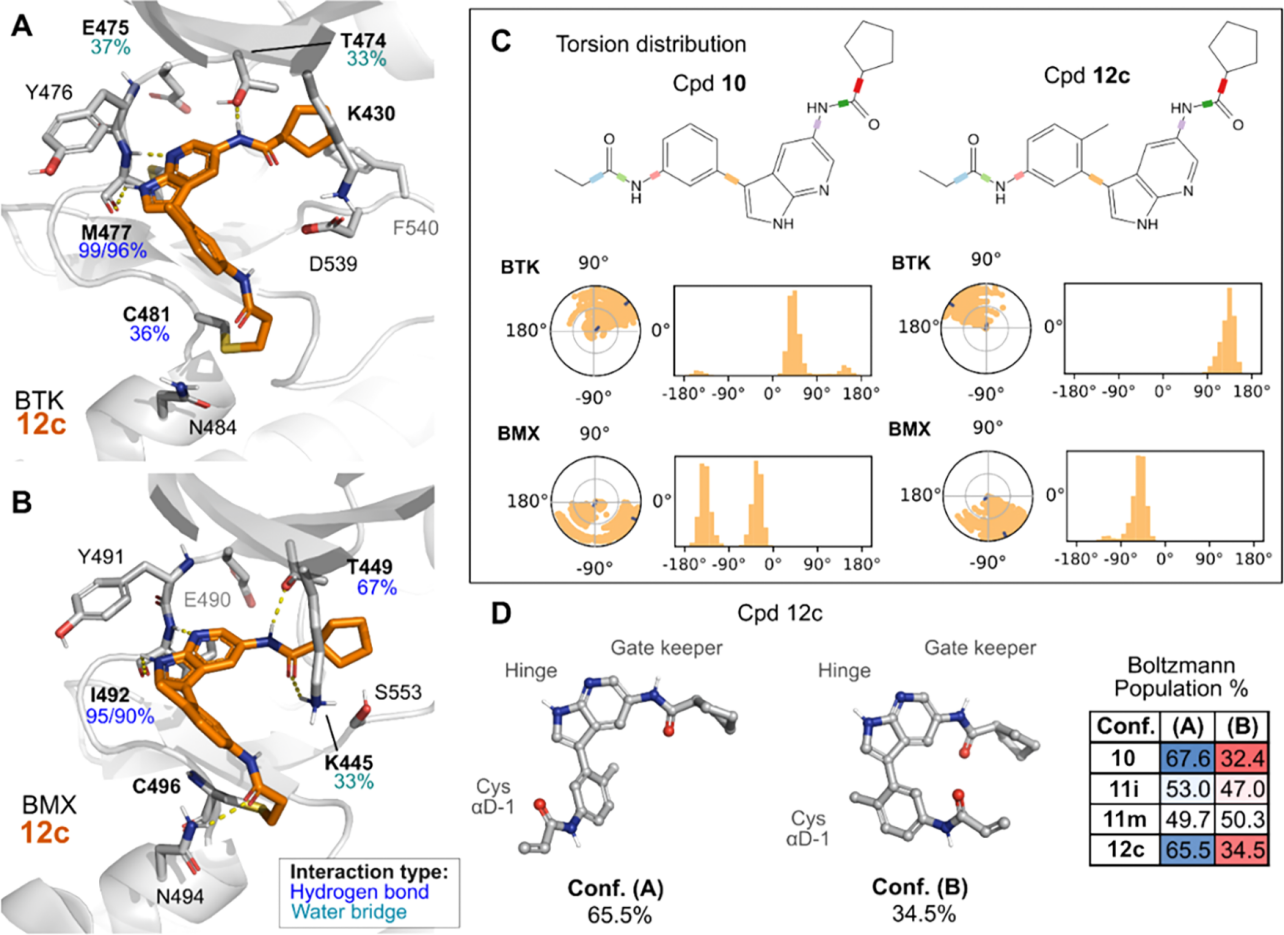
Potential binding mode of compounds **12c** within the binding pocket from BTK (A) and BMX (B). Relevant frames from the MD simulation (derived from cluster analyses), display the potential binding mode of the compound. The interaction frequency of hydrogen bonds and water bridges, along the analyzed 5 μs (5 × 1 μs) is depicted in blue and turquoise, respectively. Bold interactions were stable over more than 30% of the analyzed simulation. (C) Torsion angles distribution along the simulations, highlighting the distribution of torsions between the hinge binding motif and the phenyl linker in orange. (D) quantum mechanics calculated conformer and tautomer predictor output cluster conformations of compound **12c**, grouped as (A)—with the acrylamide group pointing to the direction of the covalently captured cysteine residue and (B)—where the acrylamide group points outward. Inset D table depicts the calculated Boltzmann Population for each compound clustering frequency of their output conformations according to (A) or (B).

### 2.6 Microsomal and Glutathione Stability

The *in vitro* metabolism of key compounds from different structural series (i.e., **11m**, **12b**, and **12c**) was assessed by monitoring stability in human liver microsomes over 2 h. Interestingly, around 30% of **12b** (butynamide) and **12c** (*ortho*-Me-Ph-linker) were metabolized during this time frame whereas **11m**, with a fluorophenyl backpocket residue was >90% unmetabolized suggesting greater metabolic stability (Figure 8A). The most significant modification observed was *N*-deacylation on both, the warhead site and the acylamino residue in position 5 of the azaindole (for details see Figure S2). Moreover, a set of compounds including **11i**, **12b**, and **12c** was assessed in an HPLC-based glutathione (GSH) stability assay for evaluating intrinsic reactivity against a standard, i.e., the FDA-approved kinase inhibitor afatinib. Under conditions used here, afatinib showed a half-life *t*_1/2_ of 6.89 ± 0.15 h and all covalent BMX inhibitors showed a significantly lower reactivity with no significant degradation over the measured time frame of 12 h.

**Figure 8:**
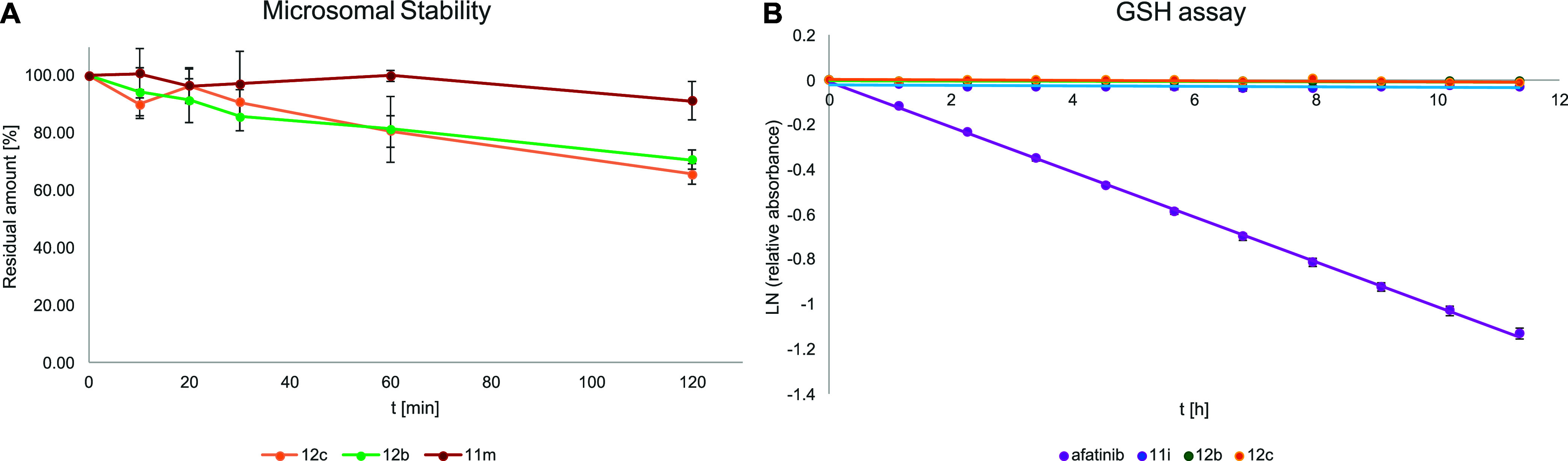
(A) Microsomal stability of compounds **11m**, **12b**, and **12c** (0.1 mM compound, human liver microsomes, TRIS-HCl buffer, pH 7.4, 37 °C, *n* = 2) and (B) GSH stability assessment (5 mM GSH, MeCN/PBS buffer 50:50, pH 7.4, 40 °C, *n* = 2) of **11i**, **12b**, and **12c**.

Overall, these results suggest that BMX inhibitory potency is driven mostly by moderate affinity combined with efficient covalent inactivation (*k*_inact_), the latter being promoted by proper warhead placement rather than high intrinsic reactivity.

## 3 Discussion and Conclusion

In this study, we designed and synthesized two sets of BMX inhibitors derived from parent compound **10** identified in our previous work.^34^ The aim of our study was to improve selectivity over the closely related kinase BTK. The first compound set exploited subtle differences in the “behind gatekeeper pockets” of BMX and BTK through variation of the 5-acylamino substituent. We employed aliphatic and (hetero)­aryl substituents as well as halogenated analogues thereof for adequate pocket filling and building up CH−π, π–π or halogen−π interactions. Our data revealed that the presence of flexible aliphatic substituents (**11a**–**e**) or small heteroaromatic rings (**11f** and **11g**) as well as the introduction of a more bulky chloro- or fluoroaryl substituent (**11i**–**m**) contribute to favorable interactions with the HR-I of BMX, resulting in compounds that inhibit recombinant BMX with an IC_50_ in the single-digit nanomolar range or below. Moreover, in cellular NanoBRET assays **11i** and **11m** emerged as key compounds with nanomolar potencies and the highest selectivity for BMX over BTK, being 21- and 94-fold, respectively. As this assay did solely quantify cellular target engagement in HEK293T cells, other cellular systems may have an effect on inhibitor potency and selectivity which may shift ideal concentrations to be applied in experiments interrogating BMX biology. Although we found promising overall characteristics for our compounds (*vide infra*), further complementary experiments will be needed to determine pharmacokinetic properties, target occupancy and selectivity *in vivo*.

With a second set, we successfully implemented a strategy to fine-tune selectivity by altering the orientation and nature of the reactive warhead moiety exemplified by compounds **12b**, **12c**, and **12d**, all exhibiting IC_50_ values for BMX in the nanomolar range. Although potencies in the cellular target engagement assay were lower compared to the aforementioned compounds, the selectivity of **12b** and **12c** was shifted toward BMX and these compounds were also selected for further profiling. Overall, both sets showed significantly improved selectivity for BMX vs BTK when compared to parent compound **10**, which was, however, also accompanied by a certain loss of cellular potency.

A DSF kinase panel of around 100 kinases used for broader selectivity assessment revealed that **11i**, **11n**, and **12c** possessed the best selectivity profile, hitting two, none, and two additional kinases with a Δ*T*_m_ > 4 K, respectively. Results where corroborated with a NanoBRET-based cellular selectivity screen against 192 kinases where compound **11i** showed a very clean profile binding only the two TEC family kinases BMX and TXK. An assessment of all kinases with an equivalently positioned cysteine (at 250 nM, Hotspot kinase assay^36^) confirmed relatively clean yet slightly varying profiles of compounds **11i**, **11m**, **12b**, and **12c**. Compound **11i** only inhibited TXK by around 70% and HER4 by around 65%, offering the best combination of high potency and selectivity. Interestingly, JS25 (**7**), a compound previously described as BMX inhibitor, revealed in a panel of 36 BMX-related kinases (at a concentration of 1 μM^31,42^ ) that besides several TEC kinases and BLK, JAK3 remained a relevant off-target. BMX inhibitor IHMT-15130 (**9**) was investigated against a panel of 207 kinases revealing 12 off-targets (>70% inhibition @ 1 μM) also including most of the TEC family members^33^ whereas the compounds presented here retain TXK as a relevant off-target, while selectivity over other TEC family members was improved. Similarly, most of our compounds spare JAK3, presumably due to an additional gatekeeper interaction and the increased steric bulk of the HR-I interacting groups.

High potencies of our compounds were further confirmed by kinetic studies using the continuous AssayQuant PhosphoSens® format where compounds **11i** and **12b** showed similar *k*_inact_/*K*_I_ values around 5 × 10^4^ M^–1^ s^–1^ and relatively fast covalent inactivation (*k*_inact_ = 8.3 × 10^–3^ s^–1^ and 9.5 × 10^–3^ s^–1^, respectively) while compound **12c** proved to be 1 order of magnitude less potent. Furthermore, fast covalent modification of BMX by **11i** was experimentally proven through intact protein mass spectrometry. In terms of *in vitro* metabolic stability and intrinsic reactivity, all key compounds showed favorable properties. Results could partly be rationalized through docking and MD simulation studies as well as torsion analysis, which will provide a good starting point for further optimization.

We successfully identified **11i** as a potent BMX inhibitor with excellent inhibitory activity, cellular target engagement, and selectivity, while its nonreactive propionamide analogue **33** loses its BMX inhibitory properties almost completely (IC_50_ > 5 μM) and may thus be used as a negative control. Overall, inhibitor **11i** emerges as one of the best currently available chemical probes for interrogating BMX biology.

## 4 Materials and Methods

### 4.1 Synthetic Procedures

All starting materials and reagents were purchased from Sigma-Aldrich, TCI, Alfa Aesar, and Acros Organic (Italy or Germany) and used without further purification. Thin-layer chromatography (TLC) was carried out on Merck 60 F254 silica plates (Merck KGaA, Darmstadt, Germany). Developed plates were air-dried and visualized by exposure to UV light (λ = 254 and 365 nm) or by an appropriate staining reagent. Preparative column chromatography was carried out with an Interchim PuriFlash 430 or PuriFlash XS420 (Interchim S.A., Montlucon, Allier, France) automated flash chromatography system using normal phase Grace Davison Davisil LC60A 20–45 μm silica (W.R. Grace and Company, Columbia, MD, USA) or Merck Geduran Si60 63–200 μm silica (Merck KGaA, Darmstadt, Germany). Nuclear magnetic resonance (NMR) spectral analysis was performed on Bruker Avance III HD 600/400/200 instruments (Bruker Corporation, Billerica, MA, USA). The samples were dissolved in deuterated solvents and chemical shifts (δ) are given in parts per million (ppm) in relation to tetramethylsilane (TMS). Spectra were calibrated using the residual peaks of the used solvent. ^1^H and ^13^C NMR spectra were acquired at 300 K and coupling constants (J) are reported in hertz (Hz). Spin multiplicity is reported as s = singlet, br s = broad singlet, d = doublet, dd = doublet of doublets, t = triplet, td = triplet of doublets, q = quartet, m = multiplet. Mass spectrometry (MS) was carried out with an Advion TLC-MS interface (Advion, Ithaca, NY, USA) with electrospray ionization (ESI) in positive and/or negative mode. Instrument settings were as follows: ESI voltage 3.50 kV, capillary voltage 187 V, source voltage 44 V, capillary temperature 250 °C, desolvation gas temperature 250 °C, and gas flow 5 L/min nitrogen.

Purities of final compounds were determined via high-performance liquid chromatography (HPLC). Method A: Agilent 1100 Series LC system (Agilent Technologies, Santa Clara, CA, USA) with a Phenomenex Luna C8 column (150 × 4.6 mm, 5 μm) (Phenomenex Inc. Torrance, CA, USA) and detection was performed with a UV DAD at 254 and 230 nm wavelength. Elution was carried out with the following gradient: mobile phase A: [H_2_O/KH_2_PO_4_ (solution 0.01 M), pH 2.30]; mobile phase B: [MeOH], 40% B to 85% B in 8 min, 85% B for 5 min, 85 to 40% B in 1 min, 40% B for 2 min, stop time 16 min, flow 1.5 mL/min. Method B: Agilent 1100 Series LC system (Agilent Technologies, Santa Clara, CA, USA) with a Phenomenex Kinetex C8 100A column (150 × 4.6 mm, 2.6 μm) (Phenomenex Inc. Torrance, CA, USA) and detection was performed with a UV DAD at 254 and 230 nm wavelength. Elution was carried out with the following gradients: mobile phase A: [H_2_O/KH_2_PO_4_ (solution 0.01 M) (v/v), pH 2.30 (solvent A)], mobile phase B: [MeOH]. 40% B to 95% in 9 min, 95% B for 1 min, 95% B to 40% B in 1 min, 40% B for 5 min, flow 0.5 mL/min. Method C: Agilent 1100 Series LC system (Agilent Technologies, Santa Clara, CA, USA) with a Phenomenex Kinetex C8 100A column (150 × 4.6 mm, 2.6 μm) (Phenomenex Inc. Torrance, CA, USA) and detection was performed with a UV DAD at 254 and 230 nm wavelength. Elution was carried out with the following gradients: mobile phase A: [H_2_0 + 0.1% TFA], mobile phase B: [ACN + 0.1% TFA]. Ten % B for 1 min, 10% B to 90% in 10 min, 90% B for 2 min, 90% B to 10% B in 1 min, 10% B for 2 min, flow 0.5 mL/min. All compounds were obtained with a purity level of 95% or higher except for compound **33** (94%).

#### 4.1.1 General Procedure A (Boronic Ester Formation)

In a flame-dried Schlenk flask the corresponding aniline (1.0 equiv), KOAc (3.0 equiv), and bis­(pinacolato)­diboron (1.0 equiv) were suspended in dry dioxane. After three degas cycles vacuum/argon Pd­(OAc)_2_ (5%) and XPhos (10%) were added to the reaction mixture. The degassing procedure was repeated three times and then the reaction was stirred overnight at 90 °C. After cooling to room temperature, the mixture was diluted with EtOAc and filtered over a layer of Celite. The filtrate was washed with water twice before drying over Na_2_SO_4_ and evaporation. The residue was purified via automated flash column chromatography on silica gel using an appropriate solvent system.

#### 4.1.2 General Procedure B (Boronic Ester Acylation)

To a solution of the corresponding boronic ester (1.0 equiv) and Et_3_N (1.1 equiv) in dry DCM, acryloyl chloride (1.1 equiv) was added dropwise at 0 °C using an ice bath. The reaction mixture was stirred for 1 h at room temperature. Then, the solvent was evaporated, and the residue was purified via automated flash column chromatography on silica gel and an appropriate solvent system.

#### 4.1.3 General Procedure C (7-Azaindole Acylation by Commercial Acyl Chloride)

The corresponding acyl chloride (2.0 equiv) was added dropwise to an ice bath-cooled solution of **19** and Et_3_N (3.0 equiv) in dry DCM. Then, the reaction was warmed to room temperature and stirred for 1 h. The solvent was evaporated, and the residue was purified via automated flash column chromatography on silica gel and an appropriate solvent system.

#### 4.1.4 General Procedure D (7-Azaindole Acylation Activating a Carboxylic Acid)

Thionyl chloride (3.0 equiv) was added dropwise to a 0.37 M ice bath-cooled solution of the corresponding carboxylic acid in dry DCM, in the presence of a catalytic amount of DMF (1 or 2 drops). The mixture was warmed to 40 °C and stirred for 3 h. Then, the organic layer was evaporated and a 0.24 M solution of the previously formed acyl chloride in dry DCM was added dropwise to a solution of **19** and Et_3_N (3.6 equiv) in dry DCM. The reaction mixture was stirred at room temperature for 1 h. Afterward, the solvent was evaporated, and the residue was purified via automated flash column chromatography on silica gel and an appropriate solvent system.

#### 4.1.5 General Procedure E (Suzuki Coupling)

The corresponding boronic acid (1.5 equiv) and aryl bromide (1.0 equiv) were solubilized in dioxane in a dry flask to obtain a 0.14 M solution. The mixture was degassed for 7 min. Then, Pd-*t*Bu_3_P G3 (5%) was added, and the mixture was degassed for another 5 min. Finally, a previously degassed aqueous solution of K_2_CO_3_ (0.5 M, 3.0 equiv) was added and the mixture was degassed for another 5 min. All degas cycles were done by sonication under argon. The reaction was stirred overnight at room temperature or at 80 °C under an argon atmosphere. The solvent in the reaction mixture was evaporated, and the residue was purified by automated flash column chromatography on silica gel and an appropriate solvent system.

#### 4.1.6 General Procedure F (SEM Cleavage)

TFA was added to a 0.037 M solution of the SEM-protected compound dissolved in dry DCM, and the solution was stirred until the TLC control showed complete consumption of the starting material (5 h to overnight). Then, the organic layer was removed, and the residue was suspended in EtOH, basified by NH_3_ (25%, aq.), and stirred at room temperature until HPLC indicated complete consumption of the hydroxymethyl intermediate (usually overnight). The volatile layer was evaporated under reduced pressure, and the residue was purified by automated flash column chromatography on silica gel and an appropriate solvent system.

##### 4.1.6.1 *N*-(3-(5-Isobutyramido-1*H*-pyrrolo­[2,3-*b*]­pyridin-3-yl)­phenyl)­acrylamide (**11a**)

The preparation was carried out following *General Procedure F* starting from 36 mg of **21a** (0.075 mmol). Flash purification by eluting with DCM/MeOH (gradient: 2–5% of MeOH) afforded 10 mg (36%) of the title compound as a white solid. ^1^H NMR (400 MHz, DMSO) δ 11.83 (s, 1H), 10.22 (s, 1H), 9.91 (s, 1H), 8.52 (d, *J* = 2.1 Hz, 1H), 8.44 (d, *J* = 2.2 Hz, 1H), 7.93 (s, 1H), 7.75 (d, *J* = 2.6 Hz, 1H), 7.62 (d, *J* = 7.9 Hz, 1H), 7.43–7.34 (m, 2H), 6.49 (dd, *J* = 17.0, 10.1 Hz, 1H), 6.29 (dd, *J* = 17.0, 2.0 Hz, 1H), 5.78 (dd, *J* = 10.1, 2.0 Hz, 1H), 2.71–2.60 (m, 1H), 1.14 (d, *J* = 6.8 Hz, 6H); ^13^C NMR (101 MHz, DMSO) δ 175.20, 163.18, 145.72, 139.49, 136.74, 135.54, 131.94, 129.62, 129.24, 126.84, 124.31, 121.67, 118.25, 117.26, 116.90, 116.65, 114.20, 34.74, 19.54. TLC-MS (ESI) *m*/*z*: 349.4 [M + H]^+^; 371.3 [M + Na]^+^; 347.2 [M – H]^−^. HRMS ESI-TOF [M + H]^+^ *m*/*z* calculated for C_20_H_20_N_4_O_2_: 349.1665, found: 349.1662. HPLC *t*_ret_ = 5.97 min. (Method A); purity: 97.5% (254.4 nm), 98.1% (230.4 nm).

##### 4.1.6.2 *N*-(3-(5-Propionamido-1*H*-pyrrolo­[2,3-*b*]­pyridin-3-yl)­phenyl)­acrylamide (**11b**)

The preparation was carried out following *General Procedure F* starting from 42 mg of **21b** (0.09 mmol). Flash purification by eluting with DCM/MeOH (gradient: 2–5% of MeOH) afforded 19 mg (63%) of the title compound as a white solid. ^1^H NMR (400 MHz, DMSO) δ 11.83 (bs, 1H), 10.22 (s, 1H), 9.95 (s, 1H), 8.49 (d, *J* = 2.0 Hz, 1H), 8.41 (d, *J* = 2.1 Hz, 1H), 7.94 (s, 1H), 7.75 (d, *J* = 2.5 Hz, 1H), 7.61 (d, *J* = 7.8 Hz, 1H), 7.49–7.31 (m, 2H), 6.48 (dd, *J* = 17.0, 10.1 Hz, 1H), 6.28 (dd, *J* = 17.0, 1.9 Hz, 1H), 5.78 (dd, *J* = 10.1, 1.9 Hz, 1H), 2.36 (q, *J* = 7.5 Hz, 2H), 1.12 (t, *J* = 7.5 Hz, 3H); ^13^C NMR (101 MHz, DMSO) δ 172.00, 163.22, 145.75, 139.52, 136.74, 135.56, 131.94, 129.55, 129.28, 126.91, 124.38, 121.68, 118.31, 117.25, 116.91, 116.68, 114.20, 29.25, 9.70. TLC-MS (ESI) *m*/*z*: 357.3 [M + Na]^+^; 333.2 [M – H]^−^. HRMS ESI-TOF [M + H]^+^ *m*/*z* calculated for C_19_H_18_N_4_O_2_: 335.1508, found: 335.1504. HPLC *t*_ret_ = 6.51 min. (Method C); purity: 100.0% (254.4 nm), 100.0% (230.4 nm).

##### 4.1.6.3 *N*-(3-(3-Acrylamidophenyl)-1*H*-pyrrolo­[2,3-*b*]­pyridin-5-yl)­cyclopropanecarboxamide (**11c**)

The preparation was carried out following *General Procedure F* starting from 32 mg of **21c** (0.067 mmol). Flash purification by eluting with DCM/MeOH (gradient: 3–5% of MeOH) afforded 50 mg (85%) of the title compound as a white solid. ^1^H NMR (400 MHz, DMSO) δ 11.85 (s, 1H), 10.30 (s, 1H), 10.23 (s, 1H), 8.49 (d, *J* = 2.3 Hz, 1H), 8.41 (d, *J* = 2.3 Hz, 1H), 7.93 (t, *J* = 1.9 Hz, 1H), 7.76 (d, *J* = 2.7 Hz, 1H), 7.61 (dt, *J* = 8.1, 1.6 Hz, 1H), 7.40 (t, *J* = 7.8 Hz, 1H), 7.35 (dt, *J* = 7.7, 1.5 Hz, 1H), 6.48 (dd, *J* = 17.0, 10.1 Hz, 1H), 6.29 (dd, *J* = 17.0, 2.0 Hz, 1H), 5.78 (dd, *J* = 10.1, 2.0 Hz, 1H), 1.87–1.76 (m, 1H), 0.88–0.77 (m, 4H). ^13^C NMR (101 MHz, DMSO) δ 171.76, 163.28, 145.76, 139.54, 136.72, 135.57, 131.95, 129.62, 129.34, 127.00, 124.46, 121.70, 118.33, 117.24, 116.92, 116.73, 114.23, 14.42, 7.09. TLC-MS (ESI) *m*/*z*: 369.2 [M + Na]^+^; 345.0 [M – H]^−^. HRMS ESI-TOF [M + H]^+^ *m*/*z* calculated for C_20_H_18_N_4_O_2_: 347.1508, found: 347.1506. HPLC *t*_ret_ = 6.81 min. (Method C); purity: 100.0% (254.4 nm), 100.0% (230.4 nm).

##### 4.1.6.4 *N*-(3-(3-Acrylamidophenyl)-1*H*-pyrrolo­[2,3-*b*]­pyridin-5-yl)­cyclobutanecarboxamide (**11d**)

The preparation was carried out following *General Procedure F* starting from 75 mg of **21d** (0.15 mmol). Flash purification by eluting with DCM/MeOH (gradient: 1–6% of MeOH) afforded 38 mg (7 0%) of the title compound as a white solid. ^1^H NMR (400 MHz, DMSO) δ 11.84 (s, 1H), 10.23 (s, 1H), 9.83 (s, 1H), 8.51 (d, *J* = 1.8 Hz, 1H), 8.43 (d, *J* = 2.0 Hz, 1H), 7.93 (s, 1H), 7.76 (d, *J* = 2.4 Hz, 1H), 7.63 (d, *J* = 7.7 Hz, 1H), 7.41 (t, *J* = 7.8 Hz, 1H), 7.36 (d, *J* = 7.7 Hz, 1H), 6.49 (dd, *J* = 17.0, 10.1 Hz, 1H), 6.29 (dd, *J* = 16.9, 1.7 Hz, 1H), 5.79 (dd, *J* = 10.1, 1.8 Hz, 1H), 3.31–3.22 (m, 1H), 2.34–2.22 (m, 2H), 2.19–2.07 (m, 2H), 2.02–1.88 (m, 1H), 1.89–1.77 (m, 1H). ^13^C NMR (101 MHz, DMSO) δ 172.89, 163.17, 145.70, 139.44, 136.78, 135.49, 131.91, 129.47, 129.18, 126.74, 124.25, 121.61, 118.34, 117.24, 116.89, 116.63, 114.17, 24.62, 17.69. TLC-MS (ESI) *m*/*z*: 383.1 [M + Na]^+^; 415.1 [M + Na + MeOH]^+^; 359.1 [M – H]^−^. HRMS ESI-TOF [M + H]^+^ *m*/*z* calculated For C_21_H_20_N_4_O_2_: 361.1665, found: 361.1664. HPLC *t*_ret_ = 6.40 min. (Method A); purity: 97.4% (254.4 nm), 98.5% (230.4 nm).

##### 4.1.6.5 *N*-(3-(3-Acrylamidophenyl)-1*H*-pyrrolo­[2,3-*b*]­pyridin-5-yl)­cyclohexanecarboxamide (**11e**)

The preparation was carried out following *General Procedure F* starting from 69 mg of **21e** (0.13 mmol). Flash purification by eluting with DCM/MeOH (gradient: 2–3% of MeOH) afforded 32 mg (62%) of the title compound as a white solid. ^1^H NMR (400 MHz, DMSO) δ 11.82 (s, 1H), 10.22 (s, 1H), 9.88 (s, 1H), 8.51 (d, *J* = 2.3 Hz, 1H), 8.42 (d, *J* = 2.3 Hz, 1H), 7.92 (s, 1H), 7.74 (d, *J* = 2.6 Hz, 1H), 7.61 (d, *J* = 8.5 Hz, 1H), 7.40 (t, *J* = 7.8 Hz, 1H), 7.36–7.31 (m, 1H), 6.48 (dd, *J* = 17.0, 10.1 Hz, 1H), 6.28 (dd, *J* = 17.0, 2.1 Hz, 1H), 5.77 (dd, *J* = 10.1, 2.1 Hz, 1H), 2.36 (tt, *J* = 11.5, 3.4 Hz, 1H), 1.89–1.72 (m, 4H), 1.66 (d, *J* = 11.8 Hz, 1H), 1.50–1.37 (m, 2H), 1.35–1.16 (m, 3H). ^13^C NMR (101 MHz, DMSO) δ 174.32, 163.21, 145.69, 139.51, 136.69, 135.57, 131.94, 129.71, 129.27, 126.90, 124.32, 121.70, 118.14, 117.28, 116.92, 116.66, 114.21, 44.73, 29.21, 25.44, 25.28. TLC-MS (ESI) *m*/*z*: 411.3 [M + Na]^+^; 387.3 [M – H]^−^. HRMS ESI-TOF [M + H]^+^ *m*/*z* calculated For C_23_H_24_N_4_O_2_: 389.1978, found: 389.1976. HPLC *t*_ret_ = 7.98 min. (Method C); purity: 95.9% (254.4 nm), 95.6% (230.4 nm).

##### 4.1.6.6 *N*-(3-(3-Acrylamidophenyl)-1*H*-pyrrolo­[2,3-*b*]­pyridin-5-yl)­furan-3-carboxamide (**11f**)

The preparation was carried out following *General Procedure F* starting from 47 mg of **21f** (0.093 mmol). Flash purification by eluting with DCM/MeOH (gradient: 1–7% of MeOH) afforded 17 mg (52%) of the title compound as a white solid. ^1^H NMR (400 MHz, DMSO) δ 11.89 (s, 1H), 10.22 (s, 1H), 10.07 (s, 1H), 8.53 (d, *J* = 2.3 Hz, 1H), 8.51 (d, *J* = 2.3 Hz, 1H), 8.38 (s, 1H), 7.95 (s, 1H), 7.83–7.76 (m, 2H), 7.62 (d, *J* = 6.9 Hz, 1H), 7.44–7.35 (m, 2H), 7.03 (s, 1H), 6.47 (dd, *J* = 17.0, 10.1 Hz, 1H), 6.27 (dd, *J* = 17.0, 1.9 Hz, 1H), 5.77 (dd, *J* = 10.1, 1.9 Hz, 1H). ^13^C NMR (101 MHz, DMSO) δ 163.24, 160.61, 146.10, 145.72, 144.18, 139.47, 137.90, 135.42, 131.90, 129.24, 128.70, 126.80, 124.45, 122.85, 121.63, 120.00, 117.24, 116.97, 116.69, 114.26, 109.14. TLC-MS (ESI) *m*/*z*: 395.2 [M + Na]^+^; 371.1 [M – H]^−^. HRMS ESI-TOF [M + H]^+^ *m*/*z* calculated for C_21_H_16_N_4_O_3_: 373.1301, found: 373.1298. HPLC *t*_ret_ = 6.82 min. (Method A); purity: 100.0% (254.4 nm), 100.0% (230.4 nm).

##### 4.1.6.7 *N*-(3-(3-Acrylamidophenyl)-1*H*-pyrrolo­[2,3-*b*]­pyridin-5-yl)­thiophene-3-carboxamide (**11g**)

The preparation was carried out following *General Procedure F* starting from 40 mg of **21g** (0.077 mmol). Flash purification by eluting with DCM/MeOH (gradient: 1–7% of MeOH) afforded 29 mg (94%) of the title compound as a white solid. ^1^H NMR (400 MHz, DMSO) δ 11.92 (s, 1H), 10.23 (s, 1H), 10.21 (s, 1H), 8.58 (d, *J* = 2.3 Hz, 1H), 8.56 (d, *J* = 2.3 Hz, 1H), 8.38 (t, *J* = 2.1 Hz, 1H), 7.97 (s, 1H), 7.81 (d, *J* = 2.7 Hz, 1H), 7.68 (s, 1H), 7.68 (s, 1H), 7.64 (dt, *J* = 7.0, 2.2 Hz, 1H), 7.49–7.35 (m, 2H), 6.48 (dd, *J* = 16.9, 10.1 Hz, 1H), 6.28 (dd, *J* = 17.0, 2.1 Hz, 1H), 5.78 (dd, *J* = 10.1, 2.0 Hz, 1H). ^13^C NMR (101 MHz, DMSO) δ 163.17, 161.01, 146.08, 139.46, 138.00, 137.63, 135.42, 131.89, 129.48, 129.19, 128.86, 127.03, 126.84, 126.74, 124.38, 121.59, 120.03, 117.21, 116.91, 116.65, 114.24. TLC-MS (ESI) *m*/*z* 411.2 [M + Na]^+^; 387.2 [M – H]^−^. HRMS ESI-TOF [M + H]^+^ *m*/*z* calculated for C_21_H_16_N_4_O_2_S: 389.1072, found: 389.1068. HPLC *t*_ret_ = 7.52 min. (Method A); purity: 98.7% (254.4 nm), 96.8% (230.4 nm).

##### 4.1.6.8 *N*-(3-(3-Acrylamidophenyl)-1*H*-pyrrolo­[2,3-*b*]­pyridin-5-yl)-4-methylthiophene-2-carboxamide (**11h**)

The preparation was carried out following *General Procedure F* starting from 100 mg of **21h** (0.21 mmol). Flash purification by eluting with DCM/MeOH (gradient: 2–6% of MeOH) afforded 75 mg (99%) of the title compound as a yellowish solid. ^1^H NMR (400 MHz, DMSO) δ 11.91 (s, 1H), 10.30 (s, 1H), 10.23 (s, 1H), 8.55 (s, 2H), 7.96 (s, 1H), 7.87 (s, 1H), 7.80 (d, *J* = 2.3 Hz, 1H), 7.63 (d, *J* = 6.8 Hz, 1H), 7.48–7.35 (m, 3H), 6.48 (dd, *J* = 16.9, 10.1 Hz, 1H), 6.28 (dd, *J* = 17.0, 2.1 Hz, 1H), 5.80–5.73 (m, 1H), 2.29 (s, 3H). ^13^C NMR (101 MHz, DMSO) δ 163.19, 160.09, 146.08, 139.48, 139.31, 137.89, 137.83, 135.37, 131.91, 130.85, 129.20, 128.74, 126.93, 126.74, 124.45, 121.59, 119.90, 117.20, 116.93, 116.66, 114.26, 15.36. TLC-MS (ESI) *m*/*z*: 425.3 [M + Na]^+^; 401.2 [M – H]^−^. HRMS ESI-TOF [M + H]^+^ *m*/*z* calculated for C_22_H_18_N_4_O_2_S: 403.1229, found: 403.1228. HPLC *t*_ret_ = 8.39 min. (Method B); purity: 95.7% (254.4 nm), 96.1% (230.4 nm).

##### 4.1.6.9 *N*-(3-(3-Acrylamidophenyl)-1*H*-pyrrolo­[2,3-*b*]­pyridin-5-yl)-5-chlorothiophene-2-carboxamide (**11i**)

The preparation was carried out following *General Procedure F* starting from 88 mg of **21i** (0.15 mmol). Flash purification by eluting with DCM/MeOH (gradient: 1–7% of MeOH) afforded 54 mg (81%) of the title compound as a yellowish solid. ^1^H NMR (400 MHz, DMSO) δ 11.95 (s, 1H), 10.47 (s, 1H), 10.23 (s, 1H), 8.51 (s, 2H), 7.96 (s, 1H), 7.92 (d, *J* = 3.9 Hz, 1H), 7.82 (d, *J* = 2.3 Hz, 1H), 7.62 (d, *J* = 7.1 Hz, 1H), 7.48–7.34 (m, 2H), 7.29 (d, *J* = 4.0 Hz, 1H), 6.47 (dd, *J* = 16.9, 10.1 Hz, 1H), 6.27 (dd, *J* = 17.0, 1.4 Hz, 1H), 5.77 (dd, *J* = 10.2, 1.4 Hz, 1H). ^13^C NMR (101 MHz, DMSO) δ 163.32, 159.15, 146.31, 139.57, 139.02, 138.02, 135.43, 133.86, 131.95, 129.41, 129.07, 128.40, 128.33, 127.05, 124.78, 121.74, 120.30, 117.25, 117.02, 116.77, 114.38. TLC-MS (ESI) *m*/*z*: 445.0 [M + Na]^+^; 421.0 [M – H]^−^. HRMS ESI-TOF [M + H]^+^ *m*/*z* calculated for C_21_H_15_ClN_4_O_2_S: 423.0682, found:423.0684. HPLC *t*_ret_ = 11.23 min. (Method B); purity: 95.1% (254.4 nm), 95.0% (230.4 nm).

##### 4.1.6.10 *N*-(3-(3-Acrylamidophenyl)-1*H*-pyrrolo­[2,3-*b*]­pyridin-5-yl)-4-chlorobenzamide (**11j**)

The preparation was carried out following *General Procedure F* starting from 90 mg of **21j** (0.16 mmol). Flash purification by eluting with DCM/MeOH (gradient: 1–6% of MeOH) afforded 28 mg (42%) of the title compound as a white solid. ^1^H NMR (400 MHz, DMSO) δ 11.92 (s, 1H), 10.45 (s, 1H), 10.21 (s, 1H), 8.59 (d, *J* = 1.9 Hz, 1H), 8.57 (d, *J* = 2.1 Hz, 1H), 8.04 (d, *J* = 8.5 Hz, 2H), 7.96 (s, 1H), 7.80 (d, *J* = 2.5 Hz, 1H), 7.69–7.55 (m, 3H), 7.44–7.34 (m, 2H), 6.46 (dd, *J* = 17.0, 10.1 Hz, 1H), 6.27 (dd, *J* = 17.0, 1.9 Hz, 1H), 5.76 (dd, *J* = 10.1, 1.9 Hz, 1H). ^13^C NMR (101 MHz, DMSO) δ 164.51, 163.24, 146.21, 139.54, 138.07, 136.44, 135.48, 133.38, 131.93, 129.59, 129.33, 128.95, 128.53, 126.94, 124.57, 121.70, 120.15, 117.25, 116.97, 116.70, 114.33. TLC-MS (ESI) *m*/*z*: 439.3 [M + Na]^+^; 415.0 [M – H]^−^. HRMS ESI-TOF [M + H]^+^ *m*/*z* calculated for C_23_H_17_ClN_4_O_2_: 417.1118, found: 417.1115. HPLC *t*_ret_ = 10.99 min. (Method B); purity: 96.0% (254.4 nm), 95.5% (230.4 nm).

##### 4.1.6.11 *N*-(3-(3-Acrylamidophenyl)-1*H*-pyrrolo­[2,3-*b*]­pyridin-5-yl)-3-chlorobenzamide (**11k**)

The preparation was carried out following *General Procedure F* starting from 62 mg of **21k** (0.11 mmol). Flash purification by eluting with DCM/MeOH (gradient: 1–7% of MeOH) afforded 45 mg (98%) of the title compound as a white solid. ^1^H NMR (400 MHz, DMSO) δ 11.93 (s, 1H), 10.49 (s, 1H), 10.23 (s, 1H), 8.60 (d, *J* = 2.4 Hz, 1H), 8.58 (d, *J* = 2.3 Hz, 1H), 8.07 (t, *J* = 1.9 Hz, 1H), 7.98 (s, 1H), 7.96 (s, 1H), 7.80 (d, *J* = 2.6 Hz, 1H), 7.74–7.65 (m, 1H), 7.64–7.55 (m, 2H), 7.46–7.35 (m, 2H), 6.46 (dd, *J* = 16.9, 10.1 Hz, 1H), 6.27 (dd, *J* = 17.0, 2.0 Hz, 1H), 5.76 (dd, *J* = 10.1, 2.0 Hz, 1H). ^13^C NMR (101 MHz, DMSO) δ 164.18, 163.30, 146.25, 139.57, 138.05, 136.67, 135.49, 133.32, 131.95, 131.49, 130.53, 129.38, 128.92, 127.43, 127.02, 126.50, 124.65, 121.74, 120.18, 117.29, 117.02, 116.73, 114.38. TLC-MS (ESI) *m*/*z*: 439.0 [M + Na]^+^; 415.1 [M – H]^−^. HRMS ESI-TOF [M + H]^+^ *m*/*z* calculated for C_23_H_17_ClN_4_O_2_: 417.1118, found: 417.1114. HPLC *t*_ret_ = 11.13 min. (Method B); purity: 98.8% (254.4 nm), 99.1% (230.4 nm).

##### 4.1.6.12 *N*-(3-(3-Acrylamidophenyl)-1*H*-pyrrolo­[2,3-*b*]­pyridin-5-yl)-3-fluorobenzamide (**11l**)

The preparation was carried out following *General Procedure F* starting from 90 mg of **21l** (0.17 mmol). Flash purification by eluting with DCM/MeOH (gradient: 1–7% of MeOH) afforded 50 mg (74%) of the title compound as a white solid. ^1^H NMR (400 MHz, DMSO) δ 11.93 (s, 1H), 10.46 (s, 1H), 10.23 (s, 1H), 8.59 (d, *J* = 2.1 Hz, 1H), 8.57 (d, *J* = 2.1 Hz, 1H), 7.96 (s, 1H), 7.86 (d, *J* = 7.8 Hz, 1H), 7.84–7.78 (m, 2H), 7.64–7.56 (m, 2H), 7.45 (td, *J* = 8.4, 2.1 Hz, 1H), 7.42–7.35 (m, 2H), 6.45 (dd, *J* = 17.0, 10.1 Hz, 1H), 6.26 (dd, *J* = 17.0, 1.9 Hz, 1H), 5.76 (dd, *J* = 10.1, 1.9 Hz, 1H). ^13^C NMR (101 MHz, DMSO) δ 164.32 (d, *J* = 2.0 Hz), 163.35, 162.05 (d, *J* = 244.4 Hz), 146.28, 139.59, 138.11, 137.02 (d, *J* = 7.1 Hz), 135.52, 131.95, 130.75 (d, *J* = 7.7 Hz), 129.43, 128.93, 127.08, 124.68, 123.92 (d, *J* = 2.9 Hz), 121.78, 120.26, 118.61 (d, *J* = 21.5 Hz), 117.32, 117.06, 115.69 (d, *J* = 215.7 Hz), 114.40. TLC-MS (ESI) *m*/*z*: 423.0 [M + Na]^+^; 455.0 [M + Na + MeOH]^+^; 399.1 [M – H]^−^. HRMS ESI-TOF [M + H]^+^ *m*/*z* calculated for C_23_H_17_FN_4_O_2_: 401.1414, found: 401.1409. HPLC *t*_ret_ = 10.57 min. (Method B); purity: 100.0% (254.4 nm), 100.0% (230.4 nm).

##### 4.1.6.13 *N*-(3-(3-Acrylamidophenyl)-1*H*-pyrrolo­[2,3-*b*]­pyridin-5-yl)-4-fluorobenzamide (**11m**)

The preparation was carried out following *General Procedure F* starting from 85 mg of **21m** (0.16 mmol). Flash purification by eluting with DCM/MeOH (gradient: 1–7% of MeOH) afforded 37 mg (56%) of the title compound as a yellowish solid. ^1^H NMR (400 MHz, DMSO) δ 11.92 (s, 1H), 10.40 (s, 1H), 10.23 (s, 1H), 8.58 (d, *J* = 6.7 Hz, 2H), 8.09 (dd, *J* = 8.6, 5.6 Hz, 2H), 7.96 (s, 1H), 7.80 (d, *J* = 1.7 Hz, 1H), 7.62 (d, *J* = 6.6 Hz, 1H), 7.45–7.33 (m, 4H), 6.46 (dd, *J* = 17.0, 10.1 Hz, 1H), 6.27 (dd, *J* = 17.0, 1.7 Hz, 1H), 5.77 (dd, *J* = 10.1, 1.7 Hz, 1H). ^13^C NMR (101 MHz, DMSO) δ 164.56, 164.12 (d, *J* = 249.4 Hz), 163.28, 146.20, 139.55, 138.13, 135.51, 131.94, 131.12 (d, *J* = 3.5 Hz), 130.38 (d, *J* = 8.9 Hz), 129.36, 129.06, 126.99, 124.57, 121.72, 120.20, 117.26, 116.98, 116.72, 115.42 (d, *J* = 21.9 Hz), 114.33. TLC-MS (ESI) *m*/*z*: 423.0 [M + Na]^+^; 399.1 [M – H]^−^. HRMS ESI-TOF [M + H]^+^ *m*/*z* calculated for C_23_H_17_FN_4_O_2_: 401.1414, found: 401.1411. HPLC *t*_ret_ = 10.45 min. (Method B); purity: 99.0% (254.4 nm), 98.8% (230.4 nm).

##### 4.1.6.14 *N*-(3-(5-(2-(4-Fluorophenyl)­acetamido)-1*H*-pyrrolo­[2,3-*b*]­pyridin-3-yl)­phenyl)­acrylamide (**11n**)

The preparation was carried out following *General Procedure F* starting from 56 mg of **21n** (0.10 mmol). Flash purification by eluting with DCM/MeOH (gradient: 1–7% of MeOH) afforded 30 mg (73%) of the title compound as a white solid. ^1^H NMR (400 MHz, DMSO) δ 11.86 (s, 1H), 10.28 (s, 1H), 10.22 (s, 1H), 8.49 (d, *J* = 2.2 Hz, 1H), 8.41 (d, *J* = 2.2 Hz, 1H), 7.93 (s, 1H), 7.76 (s, 1H), 7.58 (d, *J* = 7.9 Hz, 1H), 7.43–7.35 (m, 3H), 7.33 (d, *J* = 7.7 Hz, 1H), 7.15 (t, *J* = 8.9 Hz, 2H), 6.46 (dd, *J* = 17.0, 10.1 Hz, 1H), 6.27 (dd, *J* = 17.0, 1.9 Hz, 1H), 5.77 (dd, *J* = 10.1, 1.9 Hz, 1H), 3.67 (s, 2H). ^13^C NMR (101 MHz, DMSO) δ 169.07, 163.24, 161.11 (d, *J* = 242.2 Hz), 145.85, 139.47, 136.67, 135.45, 132.09 (d, *J* = 2.9 Hz), 131.92, 131.01 (d, *J* = 7.9 Hz), 129.34, 129.24, 126.83, 124.44, 121.64, 118.40, 117.29, 116.96, 116.69, 114.95 (d, *J* = 21.1 Hz), 114.25, 42.01. TLC-MS (ESI) *m*/*z*: 437.1 [M + Na]^+^; 469.0 [M + Na + MeOH]^+^; 413.2 [M – H]^−^ . HRMS ESI-TOF [M + H]^+^ *m*/*z* calculated for C_24_H_19_FN_4_O_2_: 415.1570, found: 415.1565. HPLC *t*_ret_ = 10.51 min. (Method B); purity: 96.6% (254.4 nm), 97.3% (230.4 nm).

##### 4.1.6.15 *N*-(3-(5-(2-(2,4-Difluorophenyl)­acetamido)-1*H*-pyrrolo­[2,3-*b*]­pyridin-3-yl)­phenyl)­acrylamide (**11o**)

The preparation was carried out following *General Procedure F* starting from 67 mg of **21o** (0.12 mmol). Flash purification by eluting with DCM/MeOH (gradient: 2–6% of MeOH) afforded 25 mg (48%) of the title compound as a white solid. ^1^H NMR (400 MHz, DMSO) δ 11.86 (s, 1H), 10.30 (s, 1H), 10.20 (s, 1H), 8.49 (s, 1H), 8.42 (s, 1H), 7.93 (s, 1H), 7.76 (s, 1H), 7.59 (d, *J* = 7.4 Hz, 1H), 7.47 (q, *J* = 8.0 Hz, 1H), 7.42–7.28 (m, 2H), 7.22 (t, *J* = 9.0 Hz, 1H), 7.14–7.01 (m, 1H), 6.47 (dd, *J* = 16.8, 10.1 Hz, 1H), 6.27 (d, *J* = 16.8 Hz, 1H), 5.77 (d, *J* = 10.6 Hz, 1H), 3.76 (s, 2H). ^13^C NMR (101 MHz, DMSO) δ 167.86, 163.15, 145.82, 139.44, 136.60, 135.39, 132.87 (dd, *J* = 9.4, 6.1 Hz), 131.90, 129.24, 129.15, 126.69, 124.37, 121.57, 119.21 (dd, *J* = 16.0, 4.0 Hz), 118.29, 117.24, 116.91, 116.63, 114.22, 111.07 (d, *J* = 24.4 Hz), 103.43 (t, *J* = 26.0 Hz), 35.31. Resolution was too low to observe both C–F carbons. TLC-MS (ESI) *m*/*z*: 455.4 [M + Na]^+^; 431.3 [M – H]^−^. HRMS ESI-TOF [M + H]^+^ *m*/*z* calculated for C_24_H_18_F_2_N_4_O_2_: 433.1476, found: 433.1474. HPLC *t*_ret_ = 6.40 min. (Method A); purity: 97.4% (254.4 nm), 98.5% (230.4 nm).

##### 4.1.6.16 *N*-(3-(3-Acrylamidophenyl)-1*H*-pyrrolo­[2,3-*b*]­pyridin-5-yl)-3-chloro-5-fluorobenzamide (**11p**)

The preparation was carried out following *General Procedure F* starting from 74 mg of **21p** (0.13 mmol). Flash purification by eluting with DCM/MeOH (gradient: 1–10% of MeOH) afforded 41 mg (72%) of the title compound as a white solid. ^1^H NMR (400 MHz, DMSO) δ 11.95 (s, 1H), 10.54 (s, 1H), 10.23 (s, 1H), 8.68–8.50 (m, 2H), 7.97 (s, 1H), 7.96–7.92 (m, 1H), 7.87–7.79 (m, 2H), 7.72 (dt, *J* = 8.5, 2.0 Hz, 1H), 7.61 (dt, *J* = 7.3, 2.0 Hz, 1H), 7.45–7.35 (m, 2H), 6.47 (dd, *J* = 17.0, 10.1 Hz, 1H), 6.27 (dd, *J* = 17.0, 1.9 Hz, 1H), 5.77 (dd, *J* = 10.1, 1.9 Hz, 1H). ^13^C NMR (101 MHz, DMSO) δ 163.31, 162.93, 161.87 (d, *J* = 209.6 Hz), 146.33, 139.57, 138.16 (d, *J* = 7.8 Hz), 137.99, 135.45, 134.35 (d, *J* = 10.5 Hz), 131.94, 129.39, 128.67, 127.04, 124.74, 124.00 (d, *J* = 3.0 Hz), 121.76, 120.21, 119.11 (d, *J* = 24.6 Hz), 117.32, 117.06, 116.75, 114.42, 113.82 (d, *J* = 23.1 Hz). TLC-MS (ESI) *m*/*z*: 457.0 [M + Na]^+^; 489.2 [M + Na + MeOH]^+^; 433.0 [M – H]^−^. HRMS ESI-TOF [M + H]^+^ *m*/*z* calculated for C_23_H_16_ClFN_4_O_2_: 435.1024, found: 435.1017. HPLC *t*_ret_ = 11.50 min. (Method B); purity: 96.3% (254.4 nm), 95.3% (230.4 nm).

##### 4.1.6.17 *N*-(3-(5-(2-(5-Chloro-2,4-difluorophenyl)­acetamido)-1*H*-pyrrolo­[2,3-*b*]­pyridin-3-yl) phenyl)­acrylamide (**11q**)

The preparation was carried out following *General Procedure F* starting from 45 mg of **21q** (0.075 mmol). Flash purification by eluting with DCM/MeOH (gradient: 2–5% of MeOH) afforded 19 mg (56%) of the title compound as a white solid. ^1^H NMR (400 MHz, DMSO) δ 11.88 (s, 1H), 10.34 (s, 1H), 10.22 (s, 1H), 8.49 (d, *J* = 2.0 Hz, 1H), 8.42 (d, *J* = 2.1 Hz, 1H), 7.94 (s, 1H), 7.77 (d, *J* = 2.6 Hz, 1H), 7.71 (t, *J* = 8.0 Hz, 1H), 7.59 (d, *J* = 7.9 Hz, 1H), 7.53 (t, *J* = 9.6 Hz, 1H), 7.39 (t, *J* = 7.8 Hz, 1H), 7.34 (d, *J* = 7.7 Hz, 1H), 6.47 (dd, *J* = 17.0, 10.1 Hz, 1H), 6.28 (dd, *J* = 17.0, 1.9 Hz, 1H), 5.77 (dd, *J* = 10.1, 1.8 Hz, 1H), 3.80 (s, 2H). ^13^C NMR (101 MHz, DMSO) δ 167.48, 163.24, 159.27 (dd, *J* = 247.8, 9.5 Hz), 145.90, 139.54, 136.59, 135.48, 132.94, 132.89, 131.94, 129.29 (d, *J* = 5.1 Hz), 126.95, 124.59, 121.68, 121.03 (dd, *J* = 18.0, 4.1 Hz), 118.31, 117.26, 116.96, 116.71, 114.67 (dd, *J* = 17.5, 4.0 Hz), 114.29, 105.30 (dd, *J* = 27.8, 25.3 Hz), 35.14. Resolution was too low to observe the second C–F carbon. TLC-MS (ESI) *m*/*z*: 489.2 [M + Na]^+^; 521.3 [M + Na + MeOH]^+^; 465.3 [M – H]^−^. HRMS ESI-TOF [M + H]^+^ *m*/*z* calculated for C_24_H_17_ClF_2_N_4_O_2_: 467.1086, found: 467.1082. HPLC *t*_ret_ = 11.16 min. (Method B); purity: 95.8% (254.4 nm), 95.8% (230.4 nm).

##### 4.1.6.18 *N*-(3-(5-(2-(4-Chlorophenyl)­acetamido)-1*H*-pyrrolo­[2,3-*b*]­pyridin-3-yl)­phenyl)­acrylamide (**11r**)

The preparation was carried out following *General Procedure F* starting from 32 mg of **21r** (0.057 mmol). Flash purification by eluting with DCM/MeOH (gradient: 2–6% of MeOH) afforded 22 mg (85%) of the title compound as a white solid. ^1^H NMR (400 MHz, DMSO) δ 11.86 (s, 1H), 10.29 (s, 1H), 10.21 (s, 1H), 8.48 (d, *J* = 2.1 Hz, 1H), 8.41 (d, *J* = 2.2 Hz, 1H), 7.93 (s, 1H), 7.75 (d, *J* = 1.9 Hz, 1H), 7.58 (d, *J* = 7.9 Hz, 1H), 7.44–7.29 (m, 6H), 6.47 (dd, *J* = 17.0, 10.1 Hz, 1H), 6.27 (dd, *J* = 17.0, 1.9 Hz, 1H), 5.77 (dd, *J* = 10.1, 1.9 Hz, 1H), 3.69 (s, 2H). ^13^C NMR (101 MHz, DMSO) δ 168.81, 163.24, 145.88, 139.52, 136.66, 135.48, 134.99, 131.95, 131.29, 131.12, 129.34, 129.29, 128.23, 126.91, 124.52, 121.66, 118.39, 117.26, 116.93, 116.69, 114.25, 42.18. TLC-MS (ESI) *m*/*z*: 453.2 [M + Na]^+^; 429.1 [M – H]^−^. HRMS ESI-TOF [M + H]^+^ *m*/*z* calculated for C_24_H_19_ClN_4_O_2_: 431.1275, found: 431.1271. HPLC *t*_ret_ = 11.04 min. (Method B); purity: 99.5% (254.4 nm), 95.9% (230.4 nm).

##### 4.1.6.19 (*E*)-*N*-(3-(3-(But-2-enamido)­phenyl)-1*H*-pyrrolo­[2,3-*b*]­pyridin-5-yl)­cyclopentanecarboxamide (**12a**)

The preparation was carried out following *General Procedure F* starting from 111 mg of **32a** (0.21 mmol). Flash purification by eluting with DCM/MeOH (gradient: 2–5% of MeOH) afforded 72 mg (89%) of the title compound as a white solid. ^1^H NMR (400 MHz, DMSO) δ 11.81 (s, 1H), 10.01 (s, 1H), 9.94 (s, 1H), 8.51 (d, *J* = 2.2 Hz, 1H), 8.43 (d, *J* = 2.2 Hz, 1H), 7.91 (s, 1H), 7.73 (d, *J* = 2.6 Hz, 1H), 7.60 (d, *J* = 8.1 Hz, 1H), 7.43–7.27 (m, 2H), 6.82 (dd, *J* = 15.2, 6.9 Hz, 1H), 6.17 (dd, *J* = 15.2, 1.7 Hz, 1H), 2.82 (p, *J* = 7.9 Hz, 1H), 1.95–1.82 (m, 5H), 1.82–1.65 (m, 4H), 1.64–1.51 (m, 2H). ^13^C NMR (101 MHz, DMSO) δ 174.38, 163.50, 145.69, 139.76, 136.73, 135.46, 129.64, 129.16, 126.08, 124.24, 121.35, 118.25, 117.18, 116.82, 116.66, 114.27, 45.07, 30.09, 25.69, 17.49. TLC-MS (ESI) *m*/*z*: 411.3 [M + Na]^+^; 387.2 [M – H]^−^. HRMS ESI-TOF [M + H]^+^ *m*/*z* calculated for C_23_H_24_N_4_O_2_: 389.1978, found: 389.1973. HPLC *t*_ret_ = 7.66 min. (Method A); purity: 97.1% (254.4 nm), 97.2% (230.4 nm).

##### 4.1.6.20 *N*-(3-(3-(But-2-ynamido)­phenyl)-1*H*-pyrrolo­[2,3-*b*]­pyridin-5-yl)­cyclopentane Carboxamide (**12b**)

The preparation was carried out following *General Procedure F* starting from 44 mg of **32b** (0.085 mmol). Flash purification by eluting with DCM/MeOH (gradient: 2–5% of MeOH) afforded 20 mg (65%) of the title compound as a white solid. ^1^H NMR (400 MHz, DMSO) δ 11.84 (s, 1H), 10.68 (s, 1H), 9.95 (s, 1H), 8.51 (d, *J* = 2.1 Hz, 1H), 8.41 (d, *J* = 2.2 Hz, 1H), 7.86 (t, *J* = 1.7 Hz, 1H), 7.74 (d, *J* = 2.6 Hz, 1H), 7.54 (dt, *J* = 7.5, 1.9 Hz, 1H), 7.42–7.30 (m, 2H), 2.82 (p, *J* = 7.9 Hz, 1H), 2.07 (s, 3H), 1.88 (m, 2H), 1.82–1.64 (m, 4H), 1.58 (m, 2H). ^13^C NMR (101 MHz, DMSO) δ 174.40, 150.56, 145.70, 139.07, 136.78, 135.56, 129.68, 129.25, 124.38, 121.93, 118.28, 117.35, 117.00, 116.62, 114.06, 84.11, 75.99, 45.09, 30.12, 25.72, 3.22. TLC-MS (ESI) *m*/*z*: 409.3 [M + Na]^+^; 385.1 [M – H]^−^. HRMS ESI-TOF [M + H]^+^ *m*/*z* calculated for C_23_H_22_N_4_O_2_: 387.1821, found: 387.1817. HPLC *t*_ret_ = 7.46 min. (Method A); purity: 100.0% (254.4 nm), 100.0% (230.4 nm).

##### 4.1.6.21 *N*-(3-(5-Acrylamido-2-methylphenyl)-1*H*-pyrrolo­[2,3-*b*]­pyridin-5-yl)­cyclopentane Carboxamide (**12c**)

The preparation was carried out following *General Procedure F* starting from 38 mg of **32c** (0.073 mmol). Flash purification by eluting with DCM/MeOH (gradient: 0.2–1.5% of MeOH) afforded 22 mg (81%) of the title compound as a yellowish solid. ^1^H NMR (400 MHz, DMSO) δ 11.75 (s, 1H), 10.11 (s, 1H), 9.89 (s, 1H), 8.38 (d, *J* = 2.2 Hz, 1H), 8.12 (d, *J* = 2.2 Hz, 1H), 7.66–7.56 (m, 2H), 7.52 (d, *J* = 2.5 Hz, 1H), 7.26 (d, *J* = 8.0 Hz, 1H), 6.42 (dd, *J* = 17.0, 10.1 Hz, 1H), 6.22 (dd, *J* = 17.0, 2.0 Hz, 1H), 5.72 (dd, *J* = 10.1, 2.0 Hz, 1H), 2.76 (dt, *J* = 16.1, 8.0 Hz, 1H), 2.22 (s, 3H), 1.83 (tdd, *J* = 8.0, 5.7, 2.8 Hz, 2H), 1.76–1.60 (m, 4H), 1.59–1.46 (m, 2H). ^13^C NMR (101 MHz, DMSO) δ 174.35, 163.00, 144.94, 136.85, 136.13, 134.10, 131.97, 130.96, 130.67, 129.49, 126.63, 125.36, 120.96, 118.09, 117.65, 113.66, 45.09, 30.10, 25.69, 19.95. TLC-MS (ESI) *m*/*z*: 411.3 [M + Na]^+^; 387.3 [M – H]^−^. HRMS ESI-TOF [M + H]^+^ *m*/*z* calculated for C_23_H_24_N_4_O_2_: 389.1978 found: 389.1975. HPLC *t*_ret_ = 7.48 min. (Method A); purity: 97.0% (254.4 nm), 97.6% (230.4 nm).

##### 4.1.6.22 *N*-(3-(5-Acrylamido-2-fluorophenyl)-1*H*-pyrrolo­[2,3-*b*]­pyridin-5-yl)­cyclopentane Carboxamide (**12d**)

The preparation was carried out following *General Procedure F* starting from 76 mg of **32d** (0.15 mmol). Flash purification by eluting with DCM/MeOH (gradient: 2–4% of MeOH) afforded 35 mg (59%) of the title compound as a white solid. ^1^H NMR (400 MHz, DMSO) δ 11.94 (s, 1H), 10.26 (s, 1H), 9.94 (s, 1H), 8.44 (d, *J* = 2.1 Hz, 1H), 8.33 (s, 1H), 7.91 (dd, *J* = 6.8, 2.4 Hz, 1H), 7.70–7.65 (m, 2H), 7.29 (dd, *J* = 10.1, 9.2 Hz, 1H), 6.44 (dd, *J* = 17.0, 10.1 Hz, 1H), 6.27 (dd, *J* = 17.0, 1.9 Hz, 1H), 5.77 (dd, *J* = 10.1, 1.9 Hz, 1H), 2.82–2.78 (m, 1H), 1.88–1.55 (m, 8H). ^13^C NMR (101 MHz, DMSO) δ 174.41, 163.11, 154.99 (d, *J* = 242.1 Hz), 145.21, 136.85, 135.57 (d, *J* = 2.2 Hz), 131.78, 129.72, 126.99, 126.26 (d, *J* = 5.6 Hz), 122.33 (d, *J* = 14.7 Hz), 120.46 (d, *J* = 3.2 Hz), 118.53, 117.21, 116.10 (d, *J* = 23.1 Hz), 107.89, 45.07, 30.11, 25.70. TLC-MS (ESI) *m*/*z*: 415.2 [M + Na]^+^; 391.1 [M – H]^−^. HRMS ESI-TOF [M + H]^+^ *m*/*z* calculated for C_22_H_21_FN_4_O_2_: 393.1727 found: 393.1724. HPLC *t*_ret_ = 7.79 min. (Method A); purity: 100.0% (254.4 nm), 95.0% (230.4 nm).

##### 4.1.6.23 3-(4,4,5,5-Tetramethyl-1,3,2-dioxaborolan-2-yl)­aniline (**14**)

The preparation was carried out following *General Procedure A* starting from 2.0 g of **13** (11.6 mmol) in 60.0 mL dry dioxane. Flash purification by eluting with hexane/EtOAc (gradient: 10–15% of EtOAc) afforded 1.48 g (58%) of the title compound as a brown oil. ^1^H NMR (400 MHz, CDCl_3_) δ 7.24–7.07 (m, 3H), 6.79 (ddd, *J* = 7.6, 2.4, 1.3 Hz, 1H), 3.58 (bs, 2H), 1.34 (s, 12H). ^13^C NMR (101 MHz, CDCl_3_) δ 146.15, 129.17, 125.43, 121.58, 118.48, 84.13, 25.29. TLC-MS (ESI) *m*/*z*: 220.2 [M + H]^+^. HPLC *t*_ret_ = 7.84 min. (Method B).

##### 4.1.6.24 *N*-(3-(4,4,5,5-Tetramethyl-1,3,2-dioxaborolan-2-yl)­phenyl)­acrylamide (**15**)

The reaction was carried out following *General Procedure B* starting from 1.5 g of **14** (6.7 mmol) in 28.0 mL dry DCM. Flash purification by eluting with hexane/EtOAc (gradient: 20–30% of EtOAc) afforded 1.2 g (65%) of the title compound as a yellowish solid. ^1^H NMR (400 MHz, CDCl_3_) δ 7.93 (d, *J* = 7.3 Hz, 1H), 7.76 (s, 1H), 7.65 (bs, 1H), 7.55 (d, *J* = 7.2 Hz, 1H), 7.34 (t, *J* = 7.7 Hz, 1H), 6.41 (m, 1H), 6.25 (dd, *J* = 16.9, 10.2 Hz, 1H), 5.72 (dd, *J* = 10.2, 0.9 Hz, 1H), 1.32 (s, 12H). ^13^C NMR (101 MHz, CDCl_3_) δ 163.79, 137.37, 131.36, 130.93, 128.71, 127.76, 126.19, 123.33, 84.06, 24.98. TLC-MS (ESI) *m*/*z*: 272.1 [M – H]^−^. HPLC *t*_ret_ = 10.66 min. (Method B).

##### 4.1.6.25 3-Bromo-5-nitro-1*H*-pyrrolo­[2,3-*b*]­pyridine (**17**)

2.62 g of *N*-bromosuccinimide (14.7 mmol) were added portion-wise to an ice-cooled solution of 2.0 g of **16** (12.3 mmol) in 48.0 mL dry DMF. After complete addition, the mixture was warmed to room temperature and stirred for 3 h. After complete consumption of the starting material, the mixture was diluted with water until a precipitate formed. The solid was isolated by filtration and dried at 50 °C in a convection oven to afford the title compound as 2.5 g (83%) of a yellow solid. ^1^H NMR (400 MHz, DMSO) 12.92 (bs, 1H), 9.14 (d, *J* = 2.4 Hz, 1H), 8.60 (d, *J* = 2.3 Hz, 1H), 8.04 (d, *J* = 2.4 Hz, 1H). ^13^C NMR (101 MHz, DMSO) δ 149.57, 140.40, 139.51, 130.50, 123.41, 118.39, 90.09. TLC-MS (ESI) *m*/*z*: 240.2 [M – H]^−^. HPLC *t*_ret_ = 10.70 min. (Method B).

##### 4.1.6.26 3-Bromo-5-nitro-1-((2-(trimethylsilyl)­ethoxy)­methyl)-1*H*-pyrrolo­[2,3-*b*]­pyridine (**18**)

302 mg of NaH (12.6 mmol) were added portion-wise to a solution of 3.6 g of **17** (12.6 mmol) in 29.0 mL dry DMF in an inert atmosphere. After 1 h of stirring 2.3 g of SEM-Cl (13.9 mmol) was added dropwise. The reaction was warmed to room temperature and stirred overnight. Afterward, it was diluted with 200 mL of EtOAC and transferred to a separatory funnel. The organic phase was washed four times with water (400 mL x 4). Successively, it was dried over Na_2_SO_4_ and evaporated. The title compound was obtained as 4.60 g (99%) of a brown solid and was used without further purification in the following step. ^1^H NMR (400 MHz, CDCl_3_) δ 9.24 (d, *J* = 2.4 Hz, 1H), 8.72 (d, *J* = 2.4 Hz, 1H), 7.58 (s, 1H), 5.70 (s, 2H), 3.65–3.47 (m, 2H), 0.99–0.89 (m, 2H), −0.05 (s, 9H). ^13^C NMR (101 MHz, CDCl_3_) δ 150.31, 142.18, 141.52, 131.77, 125.77, 120.68, 93.64, 74.86, 68.52, 19.20, 0.00. TLC-MS (ESI) *m*/*z*: 371.1 [M + H]^+^. HPLC *t*_ret_ = 13.58 min. (Method B).

##### 4.1.6.27 3-Bromo-1-((2-(trimethylsilyl)­ethoxy)­methyl)-1*H*-pyrrolo­[2,3-*b*]­pyridin-5-amine (**19**)

4.0 g of zinc powder (61.8 mmol) and 3.9 g of ammonium formate (61.8 mmol) were added to a solution of 4.6 g **18** (12.4 mmol) in 90.0 mL EtOH (abs.). The mixture was heated to 50 °C and stirred overnight. Then, the unreacted zinc was removed by filtration and the filtrate was concentrated under reduced pressure. The residue obtained was purified via automated flash column chromatography on silica gel by eluting with hexane/EtOAc (gradient: 20–50% of EtOAc). The title compound was obtained as 2.78 g (66%) of a brown oil. ^1^H NMR (400 MHz, CDCl_3_) δ 7.98 (d, *J* = 2.2 Hz, 1H), 7.28 (s, 1H), 7.22 (d, *J* = 2.4 Hz, 1H), 5.55 (s, 2H), 4.24 (bs, 2H), 3.57–3.43 (m, 2H), 0.94–0.81 (m, 2H), −0.08 (s, 9H). ^13^C NMR (100 MHz, CDCl_3_) δ 142.9, 135.5, 135.4, 127.7, 120.4, 114.1, 89.0, 73.1, 66.5, 17.9, −1.3. HPLC *t*_ret_ = 11.67 min. (Method B).

##### 4.1.6.28 *N*-(3-Bromo-1-((2-(trimethylsilyl)­ethoxy)­methyl)-1*H*-pyrrolo­[2,3-*b*]­pyridin-5-yl)­isobutyramide (**20a**)

The preparation was carried out following *General Procedure C* starting from 100 mg of **19** (0.29 mmol) in 3.0 mL of dry DCM. Flash purification by eluting with hexane/EtOAc (gradient: 20–30% of EtOAc) afforded 119 mg (99%) of the title compound as a brown oil. ^1^H NMR (400 MHz, CDCl_3_) δ 8.27 (d, *J* = 2.0 Hz, 1H), 8.24 (d, *J* = 2.2 Hz, 1H), 7.64 (bs, 1H), 7.34 (s, 1H), 5.58 (s, 2H), 3.57–3.43 (m, 2H), 2.64–2.50 (m, 1H), 1.27 (d, *J* = 6.9 Hz, 6H), 0.98–0.80 (m, 2H), −0.07 (s, 9H). ^13^C NMR (101 MHz, CDCl_3_) δ 176.02, 144.35, 138.23, 129.32, 127.87, 120.24, 119.86, 90.06, 73.10, 66.56, 36.51, 19.76, 17.88, −1.33. TLC-MS (ESI) *m*/*z*: 434.2 [M + Na]^+^; 466.3 [M + Na + MeOH]^+^; 410.1 [M – H]^−^. HPLC *t*_ret_ = 12.68 min. (Method B).

##### 4.1.6.29 *N*-(3-Bromo-1-((2-(trimethylsilyl)­ethoxy)­methyl)-1*H*-pyrrolo­[2,3-*b*]­pyridin-5-yl)­propionamide (**20b**)

The preparation was carried out following *General Procedure C* starting from 100 mg of **19** (0.29 mmol) in 3.0 mL of dry DCM. Flash purification by eluting with hexane/EtOAc (gradient: 20–40% of EtOAc) afforded 104 mg (90%) of the title compound as a brown oil. ^1^H NMR (400 MHz, CDCl_3_) δ 8.26 (d, *J* = 2.1 Hz, 1H), 8.18 (d, *J* = 2.2 Hz, 1H), 7.79 (bs, 1H), 7.33 (s, 1H), 5.57 (s, 2H), 3.56–3.44 (m, 2H), 2.43 (q, *J* = 7.5 Hz, 2H), 1.25 (t, *J* = 7.5 Hz, 3H), 1.00–0.78 (m, 2H), −0.08 (s, 9H). ^13^C NMR (101 MHz, CDCl_3_) δ 172.85, 144.37, 138.35, 129.25, 127.83, 120.22, 119.80, 90.01, 73.07, 66.56, 30.48, 17.86, 9.79, −1.34. TLC-MS (ESI) *m*/*z*: 420.2 [M + Na]^+^; 452.2 [M + Na + MeOH]^+^; 396.1 [M – H]^−^. HPLC *t*_ret_ = 12.40 min. (Method B)

##### 4.1.6.30 *N*-(3-Bromo-1-((2-(trimethylsilyl)­ethoxy)­methyl)-1*H*-pyrrolo­[2,3-*b*]­pyridin-5-yl)­cyclopropanecarboxamide (**20c**)

The preparation was carried out following *General Procedure C* starting from 100 mg of **19** (0.29 mmol) in 3.0 mL of dry DCM. Flash purification by eluting with hexane/EtOAc (gradient: 0–30% of EtOAc) afforded 120 mg (>99%) of the title compound as a brown oil. ^1^H NMR (400 MHz, CDCl_3_) δ 8.33 (s, 1H), 8.28 (s, 1H), 8.16 (bs, 1H), 7.34 (s, 1H), 5.60 (s, 2H), 3.62–3.45 (m, 2H), 1.68–1.53 (m, 1H), 1.10 (m, 2H), 0.92–0.86 (m, 2H), 0.84 (dd, *J* = 7.5, 2.9 Hz, 2H), −0.07 (s, 9H). ^13^C NMR (101 MHz, CDCl_3_) δ 172.81, 143.54, 137.37, 129.73, 128.05, 120.74, 120.28, 90.27, 73.37, 66.67, 17.90, 15.57, 8.21, −1.33. TLC-MS (ESI) *m*/*z*: 432.2 [M + Na]^+^; 464.3 [M + Na + MeOH]^+^. HPLC *t*_ret_ = 12.53 min. (Method B).

##### 4.1.6.31 *N*-(3-Bromo-1-((2-(trimethylsilyl)­ethoxy)­methyl)-1*H*-pyrrolo­[2,3-*b*]­pyridin-5-yl)­cyclobutanecarboxamide (**20d**)

The preparation was carried out following *General Procedure D* starting from 100 mg of **19** (0.29 mmol) in 5.0 mL of DCM. Flash purification by eluting with hexane/EtOAc (gradient: 20–40% of EtOAc) afforded 108 mg (88%) of the title compound as a yellow oil. ^1^H NMR (400 MHz, CDCl_3_) δ 8.28 (d, *J* = 2.0 Hz, 1H), 8.24 (d, *J* = 2.0 Hz, 1H), 7.40 (bs, 1H), 7.35 (s, 1H), 5.59 (s, 2H), 3.62–3.45 (m, 2H), 3.29–3.14 (m, 1H), 2.53–2.34 (m, 2H), 2.34–2.16 (m, 2H), 2.13–1.82 (m, 2H), 0.98–0.80 (m, 2H), −0.07 (s, 9H). ^13^C NMR (101 MHz, CDCl_3_) δ 173.83, 144.41, 138.15, 129.32, 127.87, 119.99, 119.85, 90.05, 73.08, 66.57, 40.74, 25.49, 18.26, 17.88, −1.33. TLC-MS (ESI) *m*/*z*: 446.3 [M + Na]^+^; 478.6 [M + Na + MeOH]^+^; 422.3 [M – H]^−^. HPLC *t*_ret_ = 10.38 min. (Method A).

##### 4.1.6.32 *N*-(3-Bromo-1-((2-(trimethylsilyl)­ethoxy)­methyl)-1*H*-pyrrolo­[2,3-*b*]­pyridin-5-yl)­cyclohexanecarboxamide (**20e**)

The preparation was carried out following *General Procedure C* starting from 100 mg of **19** (0.29 mmol) in 3.0 mL of DCM. Flash purification by eluting with hexane/EtOAc (gradient: 0–40% of EtOAc) afforded 84 mg (93%) of the title compound as a yellow oil. ^1^H NMR (400 MHz, CDCl_3_) δ 8.41 (s, 1H), 8.38 (s, 1H), 7.86 (bs, 1H), 7.37 (s, 1H), 5.63 (s, 2H), 3.62–3.43 (m, 2H), 2.43–2.27 (m, 1H), 2.00 (d, *J* = 13.0 Hz, 2H), 1.90–1.80 (m, 2H), 1.66–1.51 (m, 2H), 1.39–1.20 (m, 4H), 0.93–0.83 (m, 2H), −0.06 (s, 9H). ^13^C NMR (101 MHz, CDCl_3_) δ 175.29, 142.93, 136.65, 129.77, 128.29, 121.40, 120.70, 90.49, 73.62, 66.72, 46.33, 29.80, 25.82, 25.78, 17.91, −1.31. TLC-MS (ESI) *m*/*z*: 474.1 [M + Na]^+^: 450.2 [M – H]^−^. HPLC *t*_ret_ = 13.29 min. (Method B).

##### 4.1.6.33 *N*-(3-Bromo-1-((2-(trimethylsilyl)­ethoxy)­methyl)-1*H*-pyrrolo­[2,3-*b*]­pyridin-5-yl)­furan-3-carboxamide (**20f**)

The preparation was carried out following *General Procedure D* starting from 100 mg of **19** (0.29 mmol) in 5.0 mL of DCM. Flash purification by eluting with hexane/EtOAc (gradient: 20–40% of EtOAc) afforded 87 mg (69%) of the title compound as a yellow solid. ^1^H NMR (400 MHz, CDCl_3_) δ 8.36 (d, *J* = 2.2 Hz, 1H), 8.21 (d, *J* = 2.1 Hz, 1H), 8.08 (bs, 1H), 7.85 (s, 1H), 7.48 (t, *J* = 1.7 Hz, 1H), 7.37 (s, 1H), 6.77 (s, 1H), 5.60 (s, 2H), 3.60–3.45 (m, 2H), 1.01–0.81 (m, 2H), −0.06 (s, 9H). ^13^C NMR (101 MHz, CDCl_3_) δ 161.40, 145.53, 144.66, 144.25, 138.76, 128.75, 128.05, 122.76, 120.83, 119.90, 108.52, 90.11, 73.15, 66.64, 17.90, −1.31. TLC-MS (ESI) *m*/*z*: 458.3 [M + Na]^+^; 503.6 [M + Na + MeOH]^+^; 434.2 [M – H]^−^. HPLC *t*_ret_ = 9.92 min. (Method A).

##### 4.1.6.34 *N*-(3-Bromo-1-((2-(trimethylsilyl)­ethoxy)­methyl)-1*H*-pyrrolo­[2,3-*b*]­pyridin-5-yl)­thiophene-3-carboxamide (**20g**)

The preparation was carried out following *General Procedure D* starting from 100 mg of **19** (0.29 mmol) in 5.0 mL of dry DCM. Flash purification by eluting with hexane/EtOAc (gradient: 20–40% of EtOAc) afforded 67 mg (51%) of the title compound as a yellowish solid. ^1^H NMR (400 MHz, CDCl_3_) δ 8.37 (d, *J* = 2.3 Hz, 1H), 8.27 (bs, 1H), 8.20 (d, *J* = 2.3 Hz, 1H), 8.04 (dd, *J* = 2.8, 1.1 Hz, 1H), 7.53 (dd, *J* = 5.1, 1.2 Hz, 1H), 7.36–7.30 (m, 2H), 5.58 (s, 2H), 3.70–3.39 (m, 2H), 0.93–0.83 (m, 2H), −0.07 (s, 9H). ^13^C NMR (101 MHz, CDCl_3_) δ 161.91, 144.54, 138.87, 137.35, 129.21, 128.98, 127.93, 126.92, 126.40, 120.80, 119.83, 90.08, 73.13, 66.61, 17.88, −1.31. TLC-MS (ESI) *m*/*z*: 474.2 [M + Na]^+^; 450.1 [M – H]^−^. HPLC *t*_ret_ = 10.23 min. (Method A).

##### 4.1.6.35 *N*-(3-Bromo-1-((2-(trimethylsilyl)­ethoxy)­methyl)-1*H*-pyrrolo­[2,3-*b*]­pyridin-5-yl)-4-methylthiophene-2-carboxamide (**20h**)

The preparation was carried out following *General Procedure D* starting from 100 mg of **19** (0.29 mmol) in 5.0 mL of DCM. Flash purification by eluting with hexane/EtOAc (gradient: 20–40% of EtOAc) afforded 114 mg (84%) of the title compound as a yellowish oil. ^1^H NMR (400 MHz, CDCl_3_) δ 8.37 (d, *J* = 2.3 Hz, 1H), 8.23 (d, *J* = 2.3 Hz, 1H), 8.07 (bs, 1H), 7.50 (d, *J* = 1.1 Hz, 1H), 7.35 (s, 1H), 7.19–7.08 (m, 1H), 5.59 (s, 2H), 3.58–3.47 (m, 2H), 2.27 (s, 3H), 0.94–0.82 (m, 2H), −0.07 (s, 9H). ^13^C NMR (101 MHz, CDCl_3_) δ 160.76, 144.56, 138.77, 138.69, 138.27, 131.08, 128.92, 127.96, 126.66, 120.66, 119.85, 90.11, 73.11, 66.61, 17.88, 15.78, −1.32. TLC-MS (ESI) *m*/*z*: 488.3 [M + Na]^+^; 520.3 [M + Na + MeOH]^+^; 464.2 [M – H]^−^. HPLC *t*_ret_ = 10.57 min. (Method A).

##### 4.1.6.36 *N*-(3-Bromo-1-((2-(trimethylsilyl)­ethoxy)­methyl)-1*H*-pyrrolo­[2,3-*b*]­pyridin-5-yl)-5-chlorothiophene-2-carboxamide (**20i**)

The preparation was carried out following *General Procedure C* starting from 69 mg of **19** (0.20 mmol) in 2.0 mL of DCM. Flash purification by eluting with hexane/EtOAc (gradient: 0–40% of EtOAc) afforded 84 mg (93%) of the title compound as a yellow oil. ^1^H NMR (400 MHz, CDCl_3_) δ 8.65 (bs, 1H), 8.36 (d, *J* = 2.2 Hz, 1H), 8.15 (d, *J* = 2.2 Hz, 1H), 7.52 (d, *J* = 4.0 Hz, 1H), 7.32 (s, 1H), 6.85 (d, *J* = 4.0 Hz, 1H), 5.56 (s, 2H), 3.56–3.43 (m, 2H), 0.93–0.84 (m, 2H), −0.08 (s, 9H). ^13^C NMR (101 MHz, CDCl_3_) δ 160.05, 143.83, 138.23, 137.45, 136.56, 128.67, 128.34, 128.19, 127.35, 121.48, 120.11, 90.21, 73.38, 66.71, 17.86, −1.33. TLC-MS (ESI) *m*/*z*: 508.2 [M + Na]^+^; 540.9 [M + Na + MeOH]^+^; 484.2 [M – H]^−^. HPLC *t*_ret_ = 13.43 min. (Method B).

##### 4.1.6.37 *N*-(3-Bromo-1-((2-(trimethylsilyl)­ethoxy)­methyl)-1*H*-pyrrolo­[2,3-*b*]­pyridin-5-yl)-4-chlorobenzamide (**20j**)

The preparation was carried out following *General Procedure D* starting from 80 mg of **19** (0.23 mmol) in 4.0 mL of DCM. Flash purification by eluting with hexane/EtOAc (gradient: 5–30% of EtOAc) afforded 111 mg (>99%) of the title compound as a brownish oil. ^1^H NMR (400 MHz, CDCl_3_) δ 8.43 (d, *J* = 1.9 Hz, 1H), 8.34 (bs, 1H), 8.30 (d, *J* = 1.9 Hz, 1H), 7.88 (s, 1H), 7.86 (s, 1H), 7.45–7.41 (m, 2H), 7.38 (s, 1H), 5.62 (s, 2H), 3.59–3.47 (m, 2H), 0.94–0.86 (m, 2H), −0.07 (s, 9H). ^13^C NMR (101 MHz, CDCl_3_) δ 165.37, 144.12, 138.48, 138.23, 132.75, 129.15, 129.05, 128.80, 128.23, 121.28, 120.16, 90.28, 73.33, 66.71, 17.89, −1.30. TLC-MS (ESI) *m*/*z*: 502.0 [M + Na]^+^; 534.1 [M + Na + MeOH]^+^; 478.0 [M – H]^−^. HPLC *t*_ret_ = 11.17 min. (Method A).

##### 4.1.6.38 *N*-(3-Bromo-1-((2-(trimethylsilyl)­ethoxy)­methyl)-1*H*-pyrrolo­[2,3-*b*]­pyridin-5-yl)-3-chlorobenzamide (**20k**)

The preparation was carried out following *General Procedure C* starting from 100 mg of **19** (0.29 mmol) in 3.0 mL of DCM. Flash purification by eluting with hexane/EtOAc (gradient: 0–10% of EtOAc) afforded 119 mg (86%) of the title compound as a yellow oil. ^1^H NMR (400 MHz, CDCl_3_) δ 8.43 (d, *J* = 2.1 Hz, 1H), 8.34 (bs, 1H), 8.29 (d, *J* = 2.0 Hz, 1H), 7.91 (d, *J* = 1.6 Hz, 1H), 7.80 (d, *J* = 7.7 Hz, 1H), 7.50 (dd, *J* = 8.0, 1.1 Hz, 1H), 7.41 (d, *J* = 7.8 Hz, 1H), 7.38 (s, 1H), 5.62 (s, 2H), 3.78–3.37 (m, 2H), 1.04–0.76 (m, 2H), −0.06 (s, 9H). ^13^C NMR (101 MHz, CDCl_3_) δ 165.07, 144.18, 138.27, 136.21, 135.08, 132.15, 130.20, 128.97, 128.22, 127.68, 125.47, 121.23, 120.12, 90.29, 73.32, 66.71, 17.90, −1.30. TLC-MS (ESI) *m*/*z*: 502.2 [M + Na]^+^; 534.3 [M + Na + MeOH]^+^; 478.2 [M – H]^−^. HPLC *t*_ret_ = 13.34 min. (Method B).

##### 4.1.6.39 *N*-(3-Bromo-1-((2-(trimethylsilyl)­ethoxy)­methyl)-1*H*-pyrrolo­[2,3-*b*]­pyridin-5-yl)-3-fluorobenzamide (**20l**)

The preparation was carried out following *General Procedure C* starting from 100 mg of **19** (0.29 mmol) in 3.0 mL of DCM. Flash purification by eluting with hexane/EtOAc (gradient: 10–30% of EtOAc) afforded 135 mg (>99%) of the title compound as a yellow oil. ^1^H NMR (400 MHz, CDCl_3_) δ 8.53–8.35 (m, 2H), 8.27 (s, 1H), 7.69 (d, *J* = 7.7 Hz, 1H), 7.64 (d, *J* = 9.0 Hz, 1H), 7.42 (dt, *J* = 13.2, 6.7 Hz, 1H), 7.36 (s, 1H), 7.22 (t, *J* = 8.2 Hz, 1H), 5.60 (s, 2H), 3.58–3.45 (m, 2H), 0.93–0.85 (m, 2H), −0.07 (s, 9H). ^13^C NMR (101 MHz, CDCl_3_) δ 165.14, 162.89 (d, *J* = 248.1 Hz), 144.18, 138.35, 136.66 (d, *J* = 6.8 Hz), 130.53 (d, *J* = 7.7 Hz), 128.99, 128.15, 122.85, 121.17 (d, *J* = 3.5 Hz), 120.05, 119.13 (d, *J* = 21.2 Hz), 114.79 (d, *J* = 23.1 Hz), 90.22, 73.26, 66.67, 17.87, −1.33. TLC-MS (ESI) *m*/*z*: 486.2 [M + Na]^+^; 518.2 [M + Na + MeOH]^+^; 462.2 [M – H]^−^. HPLC *t*_ret_ = 13.00 min. (Method B).

##### 4.1.6.40 *N*-(3-Bromo-1-((2-(trimethylsilyl)­ethoxy)­methyl)-1*H*-pyrrolo­[2,3-*b*]­pyridin-5-yl)-4-fluorobenzamide (**20m**)

The preparation was carried out following *General Procedure D* starting from 100 mg of **19** (0.29 mmol) in 5.0 mL of DCM. Flash purification by eluting with hexane/EtOAc (gradient: 10–20% of EtOAc) afforded 127 mg (94%) of the title compound as a yellow oil. ^1^H NMR (400 MHz, CDCl_3_) δ 8.53 (s, 1H), 8.45 (s, 1H), 8.32 (bs, 1H), 8.00 (dd, *J* = 8.7, 5.2 Hz, 2H), 7.41 (s, 1H), 7.18 (t, *J* = 8.6 Hz, 2H), 5.67 (s, 2H), 3.61–3.45 (m, 2H), 0.99–0.81 (m, 2H), −0.05 (s, 9H). ^13^C NMR (101 MHz, CDCl_3_) δ 165.31, 165.24 (d, *J* = 253.1 Hz), 143.37, 137.39, 130.54 (d, *J* = 3.6 Hz), 129.87 (d, *J* = 8.9 Hz), 129.39, 128.47, 121.95, 120.67, 116.06 (d, *J* = 22.0 Hz), 90.55, 73.62, 66.80, 17.92, −1.29. TLC-MS (ESI) *m*/*z*: 486.4 [M + Na]^+^; 518.2 [M + Na + MeOH]^+^; 462.4 [M – H]^−^. HPLC *t*_ret_ = 12.94 min. (Method B).

##### 4.1.6.41 *N*-(3-Bromo-1-((2-(trimethylsilyl)­ethoxy)­methyl)-1*H*-pyrrolo­[2,3-*b*]­pyridin-5-yl)-2-(4-fluorophenyl)­acetamide (**20n**)

The preparation was carried out following *General Procedure D* starting from 100 mg of **19** (0.29 mmol) in 5.0 mL of DCM. Flash purification by eluting with hexane/EtOAc (gradient: 10–30% of EtOAc) afforded 100 mg (72%) of the title compound as a yellow oil. ^1^H NMR (400 MHz, CDCl_3_) δ 8.15 (d, *J* = 1.9 Hz, 1H), 8.08 (d, *J* = 2.0 Hz, 1H), 7.62 (bs, 1H), 7.30–7.19 (m, 3H), 6.97 (t, *J* = 8.6 Hz, 2H), 5.49 (s, 2H), 3.64 (s, 2H), 3.53–3.30 (m, 2H), 0.89–0.67 (m, 2H), −0.17 (s, 9H). ^13^C NMR (101 MHz, CDCl_3_) δ 169.73, 162.36 (d, *J* = 246.5 Hz), 143.92, 137.63, 131.22 (d, *J* = 8.1 Hz), 130.18 (d, *J* = 3.4 Hz), 128.98, 128.18, 120.85, 120.12, 116.14 (d, *J* = 21.5 Hz), 90.20, 73.30, 66.66, 43.54, 17.86, −1.33. TLC-MS (ESI) *m*/*z*: 500.2 [M + Na]^+^; 532.2 [M + Na + MeOH]^+^; 476.3 [M – H]^−^. HPLC *t*_ret_ = 12.95 min. (Method B).

##### 4.1.6.42 *N*-(3-Bromo-1-((2-(trimethylsilyl)­ethoxy)­methyl)-1*H*-pyrrolo­[2,3-*b*]­pyridin-5-yl)-2-(2,4-difluorophenyl)­acetamide (**20o**)

The preparation was carried out following *General Procedure D* starting from 100 mg of **19** (0.29 mmol) in 5.0 mL of DCM. Flash purification by eluting with hexane/EtOAc (gradient: 20–40% of EtOAc) afforded 117 mg (81%) of the title compound as a yellow oil. ^1^H NMR (400 MHz, CDCl_3_) δ 8.23 (d, *J* = 2.3 Hz, 1H), 8.14 (d, *J* = 2.3 Hz, 1H), 7.63 (bs, 1H), 7.41–7.30 (m, 2H), 6.95–6.81 (m, 2H), 5.58 (s, 2H), 3.73 (s, 2H), 3.55–3.46 (m, 2H), 0.92–0.83 (m, 2H), −0.07 (s, 9H).^13^C NMR (101 MHz, CDCl_3_) δ 168.45, 163.11 (dd, *J* = 154.5, 12.0 Hz), 160.64 (dd, *J* = 153.2, 12.0 Hz), 144.62, 138.35, 132.50 (dd, *J* = 9.6, 5.5 Hz), 128.82, 128.02, 120.39, 119.80, 117.68 (dd, *J* = 16.1, 3.9 Hz), 111.98 (dd, *J* = 21.2, 3.7 Hz), 104.31 (t, *J* = 25.7 Hz), 90.06, 73.10, 66.61, 36.97 (d, *J* = 2.3 Hz), 17.89, −1.32. TLC-MS (ESI) *m*/*z*: 518.3 [M + Na]^+^; 550.3 [M + Na + MeOH]^+^. HPLC *t*_ret_ = 10.52 min. (Method B).

##### 4.1.6.43 *N*-(3-Bromo-1-((2-(trimethylsilyl)­ethoxy)­methyl)-1*H*-pyrrolo­[2,3-*b*]­pyridin-5-yl)-3-chloro-5-fluorobenzamide (**20p**)

104 mg of thionyl chloride (0.87 mmol) were added dropwise to an ice bath-cooled solution of 61 mg 3-chloro-5-fluorobenzoic acid in dry DCM, in the presence of a catalytic amount of DMF (1 drop). The mixture was warmed to 40 °C and stirred for 3 h. Then, the organic layer was evaporated and a solution of 3-chloro-5-fluorobenzoyl chloride in 2.0 mL of dry DCM was added dropwise to a solution of 100 mg of **19** (0.29 mmol) and 65 mg of Et_3_N (0.64 mmol) in 1.0 mL of dry DCM. The reaction was stirred at room temperature for 1 h. Afterward, the solvent was evaporated, and the residue was purified via automated flash column chromatography on silica gel by eluting with hexane/EtOAc (gradient: 10–20% of EtOAc) to afford 127 mg (88%) of the title compound as a yellow oil. ^1^H NMR (400 MHz, CDCl_3_) δ 8.44 (bs, 1H), 8.41 (d, *J* = 1.9 Hz, 1H), 8.24 (d, *J* = 2.0 Hz, 1H), 7.70 (s, 1H), 7.55 (d, *J* = 8.6 Hz, 1H), 7.37 (s, 1H), 7.27–7.22 (m, 1H), 5.61 (s, 2H), 3.59–3.46 (m, 2H), 0.93–0.85 (m, 2H), −0.06 (s, 9H). ^13^C NMR (101 MHz, CDCl_3_) δ 163.93, 162.73 (d, *J* = 252.0 Hz), 144.36, 138.42, 137.60 (d, *J* = 7.5 Hz), 135.88 (d, *J* = 10.0 Hz), 128.64, 128.27, 123.50 (d, *J* = 3.3 Hz), 121.21, 120.02, 119.73 (d, *J* = 24.8 Hz), 113.39 (d, *J* = 23.1 Hz), 90.25, 73.29, 66.73, 17.89, −1.32. TLC-MS (ESI) *m*/*z*: 520.0 [M + Na]^+^; 552.1 [M + Na + MeOH]^+^; 496.2 [M – H]^−^. HPLC *t*_ret_ = 13.62 min. (Method B).

##### 4.1.6.44 *N*-(3-Bromo-1-((2-(trimethylsilyl)­ethoxy)­methyl)-1*H*-pyrrolo­[2,3-*b*]­pyridin-5-yl)-2-(5-chloro-2,4-difluorophenyl)­acetamide (**20q**)

The preparation was carried out following *General Procedure D* starting from 69 mg of **19** (0.20 mmol) in 2.0 mL of DCM. Flash purification by eluting with hexane/EtOAc (gradient: 0–40% of EtOAc) afforded 84 mg (93%) of the title compound as a yellow oil. ^1^H NMR (400 MHz, CDCl_3_) δ 8.29 (s, 1H), 8.24 (s, 1H), 7.87 (bs, 1H), 7.45 (t, *J* = 7.8 Hz, 1H), 7.37 (s, 1H), 6.96 (t, *J* = 9.0 Hz, 1H), 5.61 (s, 2H), 3.72 (s, 2H), 3.61–3.40 (m, 2H), 0.98–0.83 (m, 2H), −0.07 (s, 9H). ^13^C NMR (101 MHz, CDCl_3_) δ 167.85, 159.76 (dd, *J* = 164.0, 10.1 Hz), 157.27 (dd, *J* = 166.6, 10.1 Hz), 143.89, 137.45, 132.71 (d, *J* = 5.2 Hz), 128.90, 128.32, 121.05, 120.25, 118.92 (dd, *J* = 17.2, 4.2 Hz), 116.99 (dd, *J* = 17.1, 4.5 Hz), 105.37 (dd, *J* = 27.3, 24.9 Hz), 90.31, 73.39, 66.72, 36.63, 17.90, −1.31. TLC-MS (ESI) *m*/*z*: 552.1 [M + Na]^+^; 584.0 [M + Na + MeOH]^+^; 528.0 [M – H]^−^. HPLC *t*_ret_ = 11.14 min. (Method A).

##### 4.1.6.45 *N*-(3-Bromo-1-((2-(trimethylsilyl)­ethoxy)­methyl)-1*H*-pyrrolo­[2,3-*b*]­pyridin-5-yl)-2-(4-chlorophenyl)­acetamide (**20r**)

The preparation was carried out following *General Procedure D* starting from 80 mg of **19** (0.23 mmol) in 4.0 mL of DCM. Flash purification by eluting with hexane/EtOAc (gradient: 10–40% of EtOAc) afforded 67 mg (59%) of the title compound as a yellow oil. ^1^H NMR (400 MHz, CDCl_3_) δ 8.30 (s, 1H), 8.21 (d, *J* = 1.8 Hz, 1H), 7.94 (bs, 1H), 7.44–7.31 (m, 5H), 5.65 (s, 2H), 3.77 (s, 2H), 3.60–3.53 (m, 2H), 0.99–0.92 (m, 2H), −0.00 (s, 9H). ^13^C NMR (101 MHz, CDCl_3_) δ 169.47, 144.02, 137.82, 133.64, 132.93, 130.90, 129.29, 128.98, 128.12, 120.74, 120.02, 90.14, 73.26, 66.65, 43.62, 17.87, −1.33. TLC-MS (ESI) *m*/*z*: 516.3 [M + Na]^+^; 547.8 [M + Na + MeOH]^+^; 492.0 [M – H]^−^. HPLC *t*_ret_ = 11.10 min. (Method A).

##### 4.1.6.46 *N*-(3-(5-Isobutyramido-1-((2-(trimethylsilyl)­ethoxy)­methyl)-1*H*-pyrrolo­[2,3-*b*]­pyridin-3-yl)­phenyl)­acrylamide (**21a**)

The preparation was carried out following *General Procedure E* starting from 105 mg of **20a** (0.25 mmol), **15** (0.38 mmol) and using 0.6 mL of K_2_CO_3_ (0.5 M, aq.). The reaction was carried out at 80 °C. Flash purification by eluting with hexane/EtOAc (gradient: 30–50% of EtOAc) afforded 61 mg (49%) of the title compound as a yellowish solid. ^1^H NMR (400 MHz, CDCl_3_) δ 8.50 (d, *J* = 2.3 Hz, 1H), 8.34 (d, *J* = 2.3 Hz, 1H), 8.10 (s, 1H), 7.81 (s, 1H), 7.73 (s, 1H), 7.58–7.51 (m, 1H), 7.48 (s, 1H), 7.35–7.28 (m, 2H), 6.44 (dd, *J* = 16.9, 1.7 Hz, 1H), 6.33 (dd, *J* = 16.8, 10.0 Hz, 1H), 5.74 (dd, *J* = 10.0, 1.6 Hz, 1H), 5.62 (s, 2H), 3.59–3.52 (m, 2H), 2.59 (hept, *J* = 6.9 Hz, 1H), 1.28 (s, 3H), 1.27 (s, 3H), 0.96–0.89 (m, 2H), −0.07 (s, 9H). ^13^C NMR (101 MHz, CDCl_3_) δ 176.25, 164.06, 146.04, 138.55, 137.61, 135.22, 131.45, 129.71, 129.02, 127.85, 126.34, 123.04, 121.07, 118.44, 118.19, 116.06, 73.25, 66.54, 36.51, 19.80, 17.95, −1.28. TLC-MS (ESI) *m*/*z*: 501.6 [M + Na]^+^; 477.5 [M – H]^−^. HPLC *t*_ret_ = 12.34 min. (Method B).

##### 4.1.6.47 *N*-(3-(5-Propionamido-1-((2-(trimethylsilyl)­ethoxy)­methyl)-1*H*-pyrrolo­[2,3-*b*]­pyridin-3-yl)­phenyl)­acrylamide (**21b**)

The preparation was carried out following *General Procedure E* starting from 104 mg of **20b** (0.26 mmol), **15** (0.39 mmol) and using 0.5 mL of K_2_CO_3_ (0.5 M, aq.). The reaction was carried out at 80 °C. Flash purification by eluting with hexane/EtOAc (gradient: 30–40% of EtOAc) afforded 42 mg (35%) of the title compound as a yellowish solid. ^1^H NMR (400 MHz, CDCl_3_) δ 8.51 (d, *J* = 2.1 Hz, 1H), 8.35 (d, *J* = 2.1 Hz, 1H), 7.88 (s, 1H), 7.75 (s, 1H), 7.57–7.46 (m, 3H), 7.39–7.30 (m, 2H), 6.46 (dd, *J* = 16.8, 1.2 Hz, 1H), 6.31 (dd, *J* = 16.8, 10.1 Hz, 1H), 5.78 (dd, *J* = 10.1, 1.2 Hz, 1H), 5.66 (s, 2H), 3.65–3.46 (m, 2H), 2.45 (q, *J* = 7.5 Hz, 2H), 1.28 (t, *J* = 8.5 Hz, 3H), 0.97–0.89 (m, 2H), −0.06 (s, 9H). ^13^C NMR (101 MHz, CDCl_3_) δ 176.25, 164.06, 146.04, 138.55, 137.61, 135.22, 131.45, 129.71, 129.02, 127.85, 126.34, 123.04, 121.07, 118.44, 118.19, 116.06, 73.25, 66.54, 36.51, 19.80, 17.95, −1.28. TLC-MS (ESI) *m*/*z*: 487.3­[M + Na]^+^; 463.3 [M – H]^−^. HPLC *t*_ret_ = 12.07 min. (Method B).

##### 4.1.6.48 *N*-(3-(3-Acrylamidophenyl)-1-((2-(trimethylsilyl)­ethoxy)­methyl)-1*H*-pyrrolo­[2,3-*b*]­pyridin-5-yl)­cyclopropanecarboxamide (**21c**)

The preparation was carried out following *General Procedure E* starting from 120 mg of **20c** (0.29 mmol), **15** (0.43 mmol) and using 0.6 mL of K_2_CO_3_ (0.5 M, aq.). The reaction was carried out at room temperature. Flash purification by eluting with DCM/MeOH (gradient: 0.1–0.2% of MeOH) afforded 95 mg (69%) of the title compound as a white solid. ^1^H NMR (400 MHz, CDCl_3_) δ 8.90 (s, 1H), 8.63 (s, 1H), 8.51 (s, 1H), 8.48 (s, 1H), 7.75 (s, 1H), 7.56 (d, *J* = 7.5 Hz, 1H), 7.40 (s, 1H), 7.24 (d, *J* = 7.9 Hz, 1H), 7.16 (d, *J* = 7.7 Hz, 1H), 6.47–6.40 (m, 2H), 5.74 (dd, *J* = 7.6, 3.9 Hz, 1H), 5.57 (s, 2H), 3.60–3.46 (m, 2H), 1.69 (t, *J* = 4.3 Hz, 1H), 1.07–1.00 (m, 2H), 0.94–0.87 (m, 2H), 0.83 (dd, *J* = 7.4, 2.9 Hz, 2H), −0.07 (s, 9H). ^13^C NMR (101 MHz, CDCl_3_) δ 173.69, 164.47, 140.34, 138.85, 133.18, 131.54, 130.32, 129.71, 127.91, 127.49, 124.89, 122.57, 121.11, 119.08, 118.70, 117.07, 74.74, 67.07, 17.98, 15.46, 8.55, −1.28. TLC-MS (ESI) *m*/*z*: 499.5 [M + Na]^+^; 475.5 [M – H]^−^. HPLC *t*_ret_ = 12.20 min. (Method B).

##### 4.1.6.49 *N*-(3-(3-Acrylamidophenyl)-1-((2-(trimethylsilyl)­ethoxy)­methyl)-1*H*-pyrrolo­[2,3-*b*]­pyridin-5-yl)­cyclobutanecarboxamide (**21d**)

The preparation was carried out following *General Procedure E* starting from 39 mg of **20d** (0.09 mmol), **15** (0.14 mmol) and using 0.2 mL of K_2_CO_3_ (0.5 M, aq.). The reaction was carried out at 80 °C. Flash purification by eluting with hexane/EtOAc (gradient: 30–60% of EtOAc) afforded 30 mg (68%) of the title compound as a yellowish solid. ^1^H NMR (400 MHz, CDCl_3_) δ 8.57 (d, *J* = 2.2 Hz, 1H), 8.33 (d, *J* = 2.2 Hz, 1H), 7.86 (s, 1H), 7.79 (s, 1H), 7.55 (s, 1H), 7.54 (s, 1H), 7.41–7.34 (m, 3H), 6.46 (dd, *J* = 16.8, 1.4 Hz, 1H), 6.32 (dd, *J* = 16.8, 10.1 Hz, 1H), 5.78 (dd, *J* = 10.1, 1.4 Hz, 1H), 5.66 (s, 2H), 3.62–3.53 (m, 2H), 3.23 (p, *J* = 8.1 Hz, 1H), 2.55–2.34 (m, 2H), 2.32–2.19 (m, 2H), 2.11–1.90 (m, 2H), 0.99–0.87 (m, 2H), −0.06 (s, 9H). ^13^C NMR (101 MHz, CDCl_3_) δ 173.94, 163.88, 146.06, 138.52, 137.40, 135.34, 131.44, 129.79, 129.02, 127.94, 126.41, 123.14, 120.88, 118.49, 118.12, 116.11, 73.27, 66.56, 40.80, 25.53, 25.01, 18.26, 17.96, −1.27. TLC-MS (ESI) *m*/*z*: 513.2 [M + Na]^+^; 489.2 [M – H]^−^. HPLC *t*_ret_ = 7.31 min. (Method A).

##### 4.1.6.50 *N*-(3-(3-Acrylamidophenyl)-1-((2-(trimethylsilyl)­ethoxy)­methyl)-1*H*-pyrrolo­[2,3-*b*]­pyridin-5-yl)­cyclohexanecarboxamide (**21e**)

The preparation was carried out following *General Procedure E* starting from 143 mg of **20e** (0.22 mmol), **15** (0.33 mmol) and using 0.7 mL of K_2_CO_3_ (0.5 M, aq.). The reaction was carried out at room temperature. Flash purification by eluting with hexane/EtOAc (gradient: 30–60% of EtOAc) afforded 70 mg (56%) of the title compound as a white solid. ^1^H NMR (400 MHz, CDCl_3_) δ 8.75 (m, 2H), 8.58 (s, 1H), 7.78 (s, 1H), 7.58 (d, *J* = 7.3 Hz, 1H), 7.44 (s, 1H), 7.31–7.23 (m, 2H), 7.16 (d, *J* = 6.6 Hz, 1H), 6.50–6.45 (m, 2H), 5.76 (dd, *J* = 7.3, 3.9 Hz, 1H), 5.64 (s, 2H), 3.61–3.52 (m, 2H), 2.39 (t, *J* = 11.4 Hz, 1H), 2.02–1.92 (m, 2H), 1.88–1.77 (m, 2H), 1.77–1.64 (m, 2H), 1.65–1.45 (m, 4H), 0.93–0.87 (m, 2H), −0.06 (s, 9H). ^13^C NMR (101 MHz, CDCl_3_) δ 176.12, 164.33, 141.04, 138.87, 138.08, 131.58, 130.02, 129.74, 127.87, 127.35, 124.87, 122.75, 119.13, 118.89, 118.77, 117.11, 67.00, 60.53, 46.11, 29.76, 25.77, 17.99, 14.34, −1.27. TLC-MS (ESI) *m*/*z*: 541.4 [M + Na]^+^; 517.4 [M – H]^−^. HPLC *t*_ret_ = 12.94 min. (Method B).

##### 4.1.6.51 *N*-(3-(3-Acrylamidophenyl)-1-((2-(trimethylsilyl)­ethoxy)­methyl)-1*H*-pyrrolo­[2,3-*b*]­pyridin-5-yl)­furan-3-carboxamide (**21f**)

The preparation was carried out following *General Procedure E* starting from 57 mg of **20f** (0.12 mmol), **15** (0.18 mmol) and using 0.25 mL of K_2_CO_3_ (0.5 M, aq.). The reaction was carried out at 80 °C. Flash purification by eluting with hexane/EtOAc (gradient: 30–60% of EtOAc) afforded 25 mg (38%) of the title compound as a yellowish solid. ^1^H NMR (400 MHz, DMSO) δ 10.25 (s, 1H), 10.15 (s, 1H), 8.60 (s, 2H), 8.41 (d, *J* = 0.5 Hz, 1H), 8.02 (s, 1H), 7.99 (s, 1H), 7.83 (t, *J* = 1.7 Hz, 1H), 7.64 (d, *J* = 8.1 Hz, 1H), 7.44 (t, *J* = 7.8 Hz, 1H), 7.39 (dt, *J* = 7.7, 1.3 Hz, 1H), 7.04 (d, *J* = 1.1 Hz, 1H), 6.48 (dd, *J* = 17.0, 10.1 Hz, 1H), 6.29 (dd, *J* = 17.0, 2.0 Hz, 1H), 5.79 (dd, *J* = 10.1, 2.0 Hz, 1H), 5.68 (s, 2H), 3.65–3.53 (m, 2H), 0.92–0.81 (m, 2H), −0.08 (s, 9H). ^13^C NMR (101 MHz, DMSO) δ 163.29, 160.69, 145.94, 145.16, 144.33, 139.65, 137.91, 134.74, 131.90, 129.66, 129.48, 127.52, 127.03, 122.81, 121.73, 120.31, 117.35, 114.53, 109.18, 72.62, 65.61, 17.21, −1.36. TLC-MS (ESI) *m*/*z*: 503.4 [M + H]^+^; 525.3 [M + Na]^+^; 501.2 [M – H]^−^. HPLC *t*_ret_ = 9.71 min. (Method A).

##### 4.1.6.52 *N*-(3-(3-Acrylamidophenyl)-1-((2-(trimethylsilyl)­ethoxy)­methyl)-1*H*-pyrrolo­[2,3-*b*]­pyridin-5-yl)­thiophene-3-carboxamide (**21g**)

The preparation was carried out following *General Procedure E* starting from 67 mg of **20g** (0.15 mmol), **15** (0.23 mmol) and using 0.3 mL of K_2_CO_3_ (0.5 M, aq.). The reaction was carried out at 80 °C. Flash purification by eluting with hexane/EtOAc (gradient: 30–60% of EtOAc) afforded 44 mg (57%) of the title compound as a white solid. ^1^H NMR (400 MHz, DMSO) δ 10.28 (s, 1H), 10.27 (s, 1H), 8.62 (s, 2H), 8.38 (t, *J* = 2.1 Hz, 1H), 8.02 (t, *J* = 1.9 Hz, 1H), 7.98 (s, 1H), 7.68 (s, 1H), 7.67 (s, 1H), 7.62 (dt, *J* = 8.2, 1.5 Hz, 1H), 7.45 (t, *J* = 7.8 Hz, 1H), 7.39 (dt, *J* = 7.8, 1.5 Hz, 1H), 6.47 (dd, *J* = 17.0, 10.1 Hz, 1H), 6.28 (dd, *J* = 17.0, 1.9 Hz, 1H), 5.78 (dd, *J* = 10.1, 1.9 Hz, 1H), 5.68 (s, 2H), 3.63–3.55 (m, 2H), 0.90–0.80 (m, 2H), −0.08 (s, 9H). ^13^C NMR (101 MHz, DMSO) δ 163.42, 161.27, 145.23, 139.69, 138.12, 137.58, 134.83, 131.92, 129.88, 129.58, 127.57, 127.17, 121.84, 120.50, 117.44, 114.62, 72.70, 65.70, 17.28, −1.28. TLC-MS (ESI) *m*/*z*: 541.4 [M + Na]^+^; 517.4 [M – H]^−^. HPLC *t*_ret_ = 9.78 min. (Method A).

##### 4.1.6.53 *N*-(3-(3-Acrylamidophenyl)-1-((2-(trimethylsilyl)­ethoxy)­methyl)-1*H*-pyrrolo­[2,3-*b*]­pyridin-5-yl)-4-methylthiophene-2-carboxamide (**21h**)

The preparation was carried out following *General Procedure E* starting from 114 mg of **20h** (0.25 mmol), **15** (0.38 mmol), and using 0.5 mL of K_2_CO_3_ (0.5 M, aq.). The reaction was carried out at 80 °C. Flash purification by eluting with hexane/EtOAc (gradient: 30–80% of EtOAc) afforded 109 mg (82%) of the title compound as a white solid. ^1^H NMR (400 MHz, DMSO) δ 10.35 (s, 1H), 10.24 (s, 1H), 8.61 (d, *J* = 2.2 Hz, 1H), 8.60 (d, *J* = 2.2 Hz, 1H), 8.00 (t, *J* = 1.9 Hz, 1H), 7.97 (s, 1H), 7.86 (d, *J* = 1.1 Hz, 1H), 7.62 (d, *J* = 8.0 Hz, 1H), 7.45 (dd, *J* = 2.2, 1.1 Hz, 1H), 7.42 (d, *J* = 7.9 Hz, 1H), 7.37 (d, *J* = 7.7 Hz, 1H), 6.47 (dd, *J* = 17.0, 10.1 Hz, 1H), 6.27 (dd, *J* = 17.0, 2.0 Hz, 1H), 5.77 (dd, *J* = 10.1, 2.0 Hz, 1H), 5.67 (s, 2H), 3.64–3.49 (m, 2H), 2.28 (s, 3H), 0.93–0.78 (m, 2H), −0.09 (s, 9H). ^13^C NMR (101 MHz, DMSO) δ 163.24, 160.16, 145.12, 139.63, 139.21, 137.98, 137.83, 134.71, 131.89, 131.00, 129.70, 129.44, 127.50, 127.21, 126.97, 121.70, 120.19, 117.32, 114.54, 72.59, 65.58, 17.18, 15.44, −1.38. TLC-MS (ESI) *m*/*z*: 555.4 [M + Na]^+^; 531.3 [M – H]^−^. HPLC *t*_ret_ = 10.07 min. (Method A).

##### 4.1.6.54 *N*-(3-(3-Acrylamidophenyl)-1-((2-(trimethylsilyl)­ethoxy)­methyl)-1*H*-pyrrolo­[2,3-*b*]­pyridin-5-yl)-5-chlorothiophene-2-carboxamide (**21i**)

The preparation was carried out following *General Procedure E* starting from 119 mg of **20i** (0.19 mmol), **15** (0.29 mmol) and using 0.5 mL of K_2_CO_3_ (0.5 M, aq.). The reaction was carried out at 80 °C. Flash purification by eluting with hexane/EtOAc (gradient: 10–70% of EtOAc) afforded 93 mg (60%) of the title compound as a white solid. ^1^H NMR (400 MHz, DMSO) δ 10.53 (s, 1H), 10.25 (s, 1H), 8.59 (d, *J* = 2.2 Hz, 1H), 8.57 (d, *J* = 2.2 Hz, 1H), 8.02 (s, 1H), 8.00 (s, 1H), 7.94 (d, *J* = 4.1 Hz, 1H), 7.63 (dt, *J* = 8.2, 1.5 Hz, 1H), 7.44 (t, *J* = 7.8 Hz, 1H), 7.38 (dt, *J* = 7.7, 1.4 Hz, 1H), 7.30 (d, *J* = 4.1 Hz, 1H), 6.48 (dd, *J* = 17.0, 10.1 Hz, 1H), 6.28 (dd, *J* = 17.0, 1.9 Hz, 1H), 5.78 (dd, *J* = 10.1, 1.9 Hz, 1H), 5.68 (s, 2H), 3.67–3.52 (m, 2H), 0.91–0.80 (m, 2H), −0.08 (s, 9H). ^13^C NMR (101 MHz, DMSO) δ 163.30, 159.16, 145.31, 139.66, 138.87, 137.99, 134.67, 133.95, 131.90, 129.52, 129.23, 129.17, 128.40, 127.69, 127.06, 121.75, 120.56, 117.38, 117.35, 114.59, 72.64, 65.64, 17.21, −1.34. TLC-MS (ESI) *m*/*z*: 575.6 [M + Na]^+^; 551.6 [M – H]^−^. HPLC *t*_ret_ = 13.05 min. (Method B).

##### 4.1.6.55 *N*-(3-(3-Acrylamidophenyl)-1-((2-(trimethylsilyl)­ethoxy)­methyl)-1*H*-pyrrolo­[2,3-*b*]­pyridin-5-yl)-4-chlorobenzamide (**21j**)

The preparation was carried out following *General Procedure E* starting from 111 mg of **20j** (0.23 mmol), **15** (0.35 mmol), and using 0.4 mL of K_2_CO_3_ (0.5 M, aq.). The reaction was carried out at 80 °C. Flash purification by eluting with hexane/EtOAc (gradient: 10–80% of EtOAc) afforded 95 mg (75%) of the title compound as a yellowish solid. ^1^H NMR (400 MHz, DMSO) δ 10.51 (s, 1H), 10.24 (s, 1H), 8.67–8.60 (m, 2H), 8.10–7.93 (m, 4H), 7.69–7.57 (m, 3H), 7.49–7.34 (m, 2H), 6.46 (dd, *J* = 17.0, 10.1 Hz, 1H), 6.26 (dd, *J* = 16.9, 2.0 Hz, 1H), 5.76 (dd, *J* = 10.1, 2.0 Hz, 1H), 5.67 (s, 2H), 3.64–3.52 (m, 2H), 0.89–0.77 (m, 2H), −0.10 (s, 9H). ^13^C NMR (101 MHz, DMSO) δ 164.65, 163.32, 145.23, 139.64, 138.14, 136.61, 134.74, 133.31, 131.90, 129.92, 129.65, 129.49, 128.59, 127.60, 127.12, 121.80, 120.49, 117.40, 117.36, 114.64, 72.52, 65.67, 17.16, −1.32. TLC-MS (ESI) *m*/*z*: 569.5 [M + Na]^+^; 601.4 [M + Na + MeOH]^+^. HPLC *t*_ret_ = 12.93 min. (Method B).

##### 4.1.6.56 *N*-(3-(3-Acrylamidophenyl)-1-((2-(trimethylsilyl)­ethoxy)­methyl)-1*H*-pyrrolo­[2,3-*b*]­pyridin-5-yl)-3-chlorobenzamide (**21k**)

The preparation was carried out following *General Procedure E* starting from 119 mg of **20k** (0.25 mmol), **15** (0.38 mmol) and using 0.5 mL of K_2_CO_3_ (0.5 M, aq.). The reaction was carried out at room temperature. Flash purification by eluting with hexane/EtOAc (gradient: 10–70% of EtOAc) afforded 67 mg (49%) of the title compound as a yellow solid. ^1^H NMR (400 MHz, DMSO) δ 10.56 (s, 1H), 10.25 (s, 1H), 8.67 (d, *J* = 2.2 Hz, 1H), 8.66 (d, *J* = 2.2 Hz, 1H), 8.09 (t, *J* = 1.8 Hz, 1H), 8.03 (t, *J* = 1.9 Hz, 1H), 8.00 (s, 1H), 7.98 (t, *J* = 1.4 Hz, 1H), 7.70 (ddd, *J* = 8.0, 2.1, 1.0 Hz, 1H), 7.67–7.56 (m, 2H), 7.45 (t, *J* = 7.8 Hz, 1H), 7.39 (dt, *J* = 7.7, 1.4 Hz, 1H), 6.48 (dd, *J* = 17.0, 10.1 Hz, 1H), 6.29 (dd, *J* = 17.0, 2.0 Hz, 1H), 5.78 (dd, *J* = 10.1, 2.0 Hz, 1H), 5.69 (s, 2H), 3.69–3.51 (m, 2H), 0.93–0.76 (m, 2H), −0.07 (s, 9H). ^13^C NMR (101 MHz, DMSO) δ 164.21, 163.30, 145.29, 139.67, 138.11, 136.55, 134.75, 133.34, 131.90, 131.58, 130.59, 129.87, 129.56, 127.59, 127.44, 127.07, 126.52, 121.77, 120.45, 117.39, 117.34, 114.58, 72.77, 65.50, 17.22, −1.33. TLC-MS (ESI) *m*/*z*: 569.5 [M + Na]^+^; 545.6 [M – H]^−^. HPLC *t*_ret_ = 12.96 min. (Method B).

##### 4.1.6.57 *N*-(3-(3-Acrylamidophenyl)-1-((2-(trimethylsilyl)­ethoxy)­methyl)-1*H*-pyrrolo­[2,3-*b*]­pyridin-5-yl)-3-fluorobenzamide (**21l**)

The preparation was carried out following *General Procedure E* starting from 135 mg of **20l** (0.29 mmol), **15** (0.44 mmol) and using 0.5 mL of K_2_CO_3_ (0.5 M, aq.). The reaction was carried out at 80 °C. Flash purification by eluting with hexane/EtOAc (gradient: 30–70% of EtOAc) afforded 95 mg (58%) of the title compound as a white solid. ^1^H NMR (400 MHz, DMSO) δ 10.53 (s, 1H), 10.27 (s, 1H), 8.66 (s, 2H), 8.03 (s, 1H), 8.00 (s, 1H), 7.88 (d, *J* = 7.9 Hz, 1H), 7.84 (d, *J* = 9.4 Hz, 1H), 7.73–7.57 (m, 2H), 7.57–7.33 (m, 3H), 6.47 (dd, *J* = 17.0, 10.1 Hz, 1H), 6.28 (dd, *J* = 17.0, 1.9 Hz, 1H), 5.78 (dd, *J* = 10.1, 1.9 Hz, 1H), 5.69 (s, 2H), 3.68–3.50 (m, 2H), 0.94–0.80 (m, 2H), −0.08 (s, 9H). ^13^C NMR (101 MHz, DMSO) δ 164.29 (d, *J* = 2.2 Hz), 163.29, 162.00 (d, *J* = 244.7 Hz), 145.27, 139.67, 138.08, 136.88 (d, *J* = 7.0 Hz), 134.74, 131.90, 130.70 (d, *J* = 8.0 Hz), 129.81, 129.50, 127.58, 127.06, 123.90 (d, *J* = 2.7 Hz), 121.76, 120.47, 118.63 (d, *J* = 20.4 Hz), 117.38, 117.34, 114.61, 114.39, 72.64, 65.63, 17.21, −1.33. TLC-MS (ESI) *m*/*z*: 553.1 [M + Na]^+^; 585.1 [M + Na + MeOH]^+^; 529.3 [M – H]^−^. HPLC *t*_ret_ = 12.62 min. (Method B).

##### 4.1.6.58 *N*-(3-(3-Acrylamidophenyl)-1-((2-(trimethylsilyl)­ethoxy)­methyl)-1*H*-pyrrolo­[2,3-*b*]­pyridin-5-yl)-4-fluorobenzamide (**21m**)

The preparation was carried out following *General Procedure E* starting from 127 mg of **20m** (0.27 mmol), **15** (0.41 mmol) and using 0.5 mL of K_2_CO_3_ (0.5 M, aq.). The reaction was carried out at 80 °C. Flash purification by eluting with hexane/EtOAc (gradient: 10–70% of EtOAc) afforded 106 mg (63%) of the title compound as a yellowish solid. ^1^H NMR (400 MHz, DMSO) δ 10.45 (s, 1H), 10.24 (s, 1H), 8.64 (s, 2H), 8.09 (dd, *J* = 8.8, 5.5 Hz, 2H), 8.01 (t, *J* = 1.9 Hz, 1H), 7.98 (s, 1H), 7.61 (d, *J* = 8.0 Hz, 1H), 7.47–7.34 (m, 4H), 6.46 (dd, *J* = 17.0, 10.1 Hz, 1H), 6.27 (dd, *J* = 16.9, 1.9 Hz, 1H), 5.76 (dd, *J* = 10.1, 2.0 Hz, 1H), 5.67 (s, 2H), 3.68–3.49 (m, 2H), 0.90–0.77 (m, 2H), −0.09 (s, 9H). ^13^C NMR (101 MHz, DMSO) δ 164.59, 163.75 (d, *J* = 166.2 Hz), 163.29, 145.21, 139.66, 138.12, 134.77, 131.90, 131.01 (d, *J* = 3.0 Hz), 130.41 (d, *J* = 9.1 Hz), 129.97, 129.49, 127.52, 127.05, 121.75, 120.46, 117.36, 117.33, 115.44 (d, *J* = 21.8 Hz), 114.58, 72.63, 65.62, 24.98, 17.22, −1.33. TLC-MS (ESI) *m*/*z*: 553.7 [M + Na]^+^; 585.5 [M + Na + MeOH]^+^. HPLC *t*_ret_ = 12.57 min. (Method B).

##### 4.1.6.59 *N*-(3-(5-(2-(4-Fluorophenyl)­acetamido)-1-((2-(trimethylsilyl)­ethoxy)­methyl)-1*H*-pyrrolo­[2,3-*b*]­pyridin-3-yl)­phenyl)­acrylamide (**21n**)

The preparation was carried out following *General Procedure E* starting from 100 mg of **20n** (0.21 mmol), **15** (0.32 mmol) and using 0.4 mL of K_2_CO_3_ (0.5 M, aq.). The reaction was carried out at room temperature. Flash purification by eluting with hexane/EtOAc (gradient: 10–70% of EtOAc) afforded 61 mg (54%) of the title compound as a yellowish solid. ^1^H NMR (400 MHz, DMSO) δ 10.37 (s, 1H), 10.26 (s, 1H), 8.56 (d, *J* = 2.2 Hz, 1H), 8.49 (d, *J* = 2.2 Hz, 1H), 8.00 (s, 1H), 7.95 (s, 1H), 7.60 (d, *J* = 8.6 Hz, 1H), 7.48–7.37 (m, 3H), 7.33 (d, *J* = 7.8 Hz, 1H), 7.23–7.11 (m, 2H), 6.48 (dd, *J* = 16.9, 10.1 Hz, 1H), 6.29 (dd, *J* = 17.0, 2.0 Hz, 1H), 5.79 (dd, *J* = 10.1, 2.0 Hz, 1H), 5.65 (s, 2H), 3.70 (s, 2H), 3.61–3.52 (m, 2H), 0.88–0.79 (m, 2H), −0.09 (s, 9H). ^13^C NMR (101 MHz, DMSO) δ 169.22, 163.28, 161.16 (d, *J* = 241.9 Hz), 144.89, 139.64, 136.63, 134.76, 132.04 (d, *J* = 3.0 Hz), 131.91, 131.13 (d, *J* = 7.9 Hz), 130.29, 129.46, 127.50, 127.02, 121.73, 118.63, 117.35, 115.03 (d, *J* = 21.1 Hz), 114.53, 72.58, 65.57, 42.03, 17.19, −1.36. TLC-MS (ESI) *m*/*z*: 567.0 [M + Na]^+^; 599.0 [M + Na + MeOH]^+^. HPLC *t*_ret_ = 12.59 min. (Method B).

##### 4.1.6.60 *N*-(3-(5-(2-(2,4-Difluorophenyl)­acetamido)-1-((2-(trimethylsilyl)­ethoxy)­methyl)-1*H*-pyrrolo­[2,3-*b*]­pyridin-3-yl)­phenyl)­acrylamide (**21o**)

The preparation was carried out following *General Procedure E* starting from 117 mg of **20o** (0.24 mmol), **15** (0.36 mmol) and using 0.5 mL of K_2_CO_3_ (0.5 M, aq.). The reaction was carried out at room temperature. Flash purification by eluting with hexane/EtOAc (gradient: 30–60% of EtOAc) afforded 66 mg (49%) of the title compound as a white solid. ^1^H NMR (400 MHz, DMSO) δ 10.40 (s, 1H), 10.24 (s, 1H), 8.55 (d, *J* = 2.2 Hz, 1H), 8.50 (d, *J* = 2.2 Hz, 1H), 8.00 (s, 1H), 7.95 (s, 1H), 7.60 (d, *J* = 8.8 Hz, 1H), 7.52–7.44 (m, 1H), 7.42 (d, *J* = 7.9 Hz, 1H), 7.34 (d, *J* = 7.8 Hz, 1H), 7.29–7.18 (m, 1H), 7.12–7.03 (m, 1H), 6.48 (dd, *J* = 17.0, 10.1 Hz, 1H), 6.28 (dd, *J* = 17.0, 2.0 Hz, 1H), 5.79 (dd, *J* = 10.1, 2.0 Hz, 1H), 5.66 (s, 2H), 3.78 (s, 2H), 3.65–3.52 (m, 2H), 0.89–0.81 (m, 2H), −0.09 (s, 9H). ^13^C NMR (101 MHz, DMSO) δ 168.09, 163.27, 161.81, 159.48, 144.89, 139.63, 136.58, 134.74, 133.10 (d, *J* = 6.2 Hz), 133.00 (d, *J* = 6.1 Hz), 131.91, 130.24, 129.44, 127.50, 126.99, 121.71, 119.22 (dd, *J* = 15.9, 3.5 Hz), 118.57, 117.35, 114.53, 111.20 (dd, *J* = 20.9, 3.4 Hz), 103.57 (t, *J* = 26.0 Hz), 72.57, 65.57, 35.43, 17.18, −1.37. Resolution was too low to observe direct C–F bond coupling. TLC-MS (ESI) *m*/*z*: 585.3 [M + Na]^+^; 561.2 [M – H]^−^. HPLC *t*_ret_ = 10.22 min. (Method A).

##### 4.1.6.61 *N*-(3-(3-Acrylamidophenyl)-1-((2-(trimethylsilyl)­ethoxy)­methyl)-1*H*-pyrrolo­[2,3-*b*]­pyridin-5-yl)-3-chloro-5-fluorobenzamide (**21p**)

The preparation was carried out following *General Procedure E* starting from 127 mg of **20p** (0.25 mmol), **15** (0.38 mmol) and using 0.5 mL of K_2_CO_3_ (0.5 M, aq.). The reaction was carried out at 80 °C. Flash purification by eluting with hexane/EtOAc (gradient: 10–70% of EtOAc) afforded 81 mg (57%) of the title compound as a white solid. ^1^H NMR (400 MHz, DMSO) δ 10.60 (s, 1H), 10.25 (s, 1H), 8.65 (d, *J* = 2.0 Hz, 1H), 8.64 (d, *J* = 2.1 Hz, 1H), 8.02 (t, *J* = 1.9 Hz, 1H), 8.00 (s, 1H), 7.96 (t, *J* = 1.7 Hz, 1H), 7.83 (ddd, *J* = 9.3, 2.4, 1.4 Hz, 1H), 7.73 (dt, *J* = 8.5, 2.0 Hz, 1H), 7.61 (dt, *J* = 8.3, 1.5 Hz, 1H), 7.44 (t, *J* = 7.8 Hz, 1H), 7.38 (dt, *J* = 7.7, 1.4 Hz, 1H), 6.47 (dd, *J* = 17.0, 10.1 Hz, 1H), 6.28 (dd, *J* = 17.0, 1.9 Hz, 1H), 5.77 (dd, *J* = 10.1, 1.9 Hz, 1H), 5.68 (s, 2H), 3.63–3.53 (m, 2H), 0.90–0.80 (m, 2H), −0.09 (s, 9H). ^13^C NMR (101 MHz, DMSO) δ 163.32, 162.96 (d, *J* = 2.9 Hz), 162.05 (d, *J* = 248.2 Hz), 145.35, 139.67, 138.05 (d, *J* = 4.6 Hz), 138.00, 134.70, 134.34 (d, *J* = 10.7 Hz), 131.90, 129.56, 129.51, 127.66, 127.08, 124.01 (d, *J* = 3.4 Hz), 121.78, 120.51, 119.16 (d, *J* = 25.3 Hz), 117.45, 117.42, 117.36, 114.65, 113.84 (d, *J* = 23.1 Hz), 72.65, 65.65, 17.22, −1.33. TLC-MS (ESI) *m*/*z*: 586.9 [M + Na]^+^; 563.0 [M – H]^−^. HPLC *t*_ret_ = 13.15 min. (Method B).

##### 4.1.6.62 *N*-(3-(5-(2-(5-Chloro-2,4-difluorophenyl)­acetamido)-1-((2-(trimethylsilyl)­ethoxy)­methyl)-1*H*-pyrrolo­[2,3-*b*]­pyridin-3-yl) phenyl)­acrylamide (**21q**)

The preparation was carried out following *General Procedure E* starting from 102 mg of **20q** (0.19 mmol), **15** (0.29 mmol) and using 0.35 mL of K_2_CO_3_ (0.5 M, aq.). The reaction was carried out at 80 °C. Flash purification by eluting with hexane/EtOAc (gradient: 10–80% of EtOAc) afforded 45 mg (47%) of the title compound as a white solid. ^1^H NMR (400 MHz, DMSO) δ 10.42 (s, 1H), 10.24 (s, 1H), 8.53 (d, *J* = 2.2 Hz, 1H), 8.47 (d, *J* = 2.2 Hz, 1H), 7.99 (s, 1H), 7.94 (s, 1H), 7.69 (t, *J* = 8.0 Hz, 1H), 7.57 (d, *J* = 8.1 Hz, 1H), 7.51 (t, *J* = 9.6 Hz, 1H), 7.41 (t, *J* = 7.9 Hz, 1H), 7.32 (d, *J* = 7.7 Hz, 1H), 6.46 (dd, *J* = 17.0, 10.1 Hz, 1H), 6.27 (dd, *J* = 17.0, 2.0 Hz, 1H), 5.77 (dd, *J* = 10.1, 2.0 Hz, 1H), 5.64 (s, 2H), 3.79 (s, 2H), 3.65–3.50 (m, 2H), 0.91–0.74 (m, 2H), −0.11 (s, 9H). ^13^C NMR (101 MHz, DMSO) δ 167.64, 163.32, 158.03, 154.95, 148.14, 144.94, 139.66, 136.60, 134.74, 132.96 (d, *J* = 5.9 Hz), 131.92, 130.17, 129.48, 127.57, 127.05, 121.75, 120.96 (dd, *J* = 14.0, 3.7 Hz), 118.66, 117.40, 114.79 (d, *J* = 3.0 Hz), 114.57, 105.32 (dd, *J* = 27.9, 25.2 Hz), 72.61, 65.60, 35.17, 17.21, −1.35. Resolution was too low to observe direct C–F bond coupling. TLC-MS (ESI) *m*/*z*: 651.4 [M + Na + MeOH]^+^. HPLC *t*_ret_ = 12.98 min. (Method B).

##### 4.1.6.63 *N*-(3-(5-(2-(4-Chlorophenyl)­acetamido)-1-((2-(trimethylsilyl)­ethoxy)­methyl)-1*H*-pyrrolo­[2,3-*b*]­pyridin-3-yl)­phenyl)­acrylamide (**21r**)

The preparation was carried out following *General Procedure E* starting from 67 mg of **20r** (0.14 mmol), **15** (0.21 mmol) and using 0.3 mL of K_2_CO_3_ (0.5 M, aq.). The reaction was carried out at room temperature. Flash purification by eluting with hexane/EtOAc (gradient: 10–80% of EtOAc) afforded 37 mg (47%) of the title compound as a white solid. ^1^H NMR (400 MHz, DMSO) δ 10.38 (s, 1H), 10.24 (s, 1H), 8.53 (d, *J* = 2.2 Hz, 1H), 8.47 (d, *J* = 2.2 Hz, 1H), 7.98 (s, 1H), 7.93 (s, 1H), 7.58 (d, *J* = 8.1 Hz, 1H), 7.43–7.36 (m, 5H), 7.32 (d, *J* = 7.8 Hz, 1H), 6.46 (dd, *J* = 17.0, 10.1 Hz, 1H), 6.27 (dd, *J* = 17.0, 2.0 Hz, 1H), 5.77 (dd, *J* = 10.1, 2.0 Hz, 1H), 5.63 (s, 2H), 3.69 (s, 2H), 3.61–3.49 (m, 2H), 0.91–0.76 (m, 2H), −0.11 (s, 9H). ^13^C NMR (101 MHz, DMSO) δ 168.99, 163.32, 144.92, 139.66, 136.67, 134.91, 134.76, 131.93, 131.35, 131.19, 130.25, 129.48, 128.27, 127.53, 127.05, 121.75, 118.72, 117.39, 114.55, 72.60, 65.60, 42.20, 17.21, −1.34. TLC-MS (ESI) *m*/*z*: 615.4 [M + Na + MeOH]^+^. HPLC *t*_ret_ = 12.97 min. (Method B).

##### 4.1.6.64 (*E*)-*N*-(3-(4,4,5,5-Tetramethyl-1,3,2-dioxaborolan-2-yl)­phenyl)­but-2-enamide (**22**)

87 mg of HATU (0.23 mmol) were added to an ice bath-cooled solution of 22 mg of (*E*)-2-butenoic acid (0.26 mmol), and 44 mg of DIPEA (0.35 mmol) in 5.6 mL of dry THF. After 15 min 50 mg of **14** (0.23 mmol) were added, the reaction was warmed to room temperature and stirred overnight. The solvent was evaporated and the crude material was purified by automated flash column chromatography on silica gel by eluting with hexane/EtOAc (gradient: 10–20% of EtOAc) to afford 39 mg (60%) of the title product as yellow oil. ^1^H NMR (400 MHz, CDCl_3_) δ 7.94 (d, *J* = 5.4 Hz, 1H), 7.71 (bs, 1H), 7.53 (d, *J* = 7.3 Hz, 1H), 7.33 (t, *J* = 7.7 Hz, 2H), 6.96 (dd, *J* = 15.1, 6.9 Hz, 1H), 5.92 (dd, *J* = 15.1, 1.7 Hz, 1H), 1.88 (dd, *J* = 6.9, 1.6 Hz, 3H), 1.33 (s, 12H). ^13^C NMR (101 MHz, CDCl_3_) δ 164.15, 141.57, 137.65, 130.66, 128.70, 126.00, 125.55, 123.24, 84.04, 24.99, 17.96. TLC-MS (ESI) *m*/*z*: 288.2 [M + H]^+^. HPLC *t*_ret_ = 10.95 min. (Method B).

##### 4.1.6.65 *N*-(3-(4,4,5,5-Tetramethyl-1,3,2-dioxaborolan-2-yl)­phenyl)­but-2-ynamide (**23**)

A solution of 21 mg 2-butynoic acid (0.25 mmol) in 3.0 mL of DCM was ice bath-cooled. Then, 52 mg of DCC (0.25 mmol), 50 mg of **14** (0.23 mmol) and a catalytic amount of DMAP were added. The mixture was stirred at room temperature overnight. Afterward, the solvent was evaporated, and the crude material was purified via flash column chromatography on silica gel by eluting with hexane/EtOAc (gradient: 10–20% of EtOAc) to afford 31 mg (47%) of the title product as yellowish oil. ^1^H NMR (400 MHz, CDCl_3_) δ 7.88 (dd, *J* = 8.1, 1.1 Hz, 1H), 7.63 (d, *J* = 1.4 Hz, 1H), 7.55 (d, *J* = 7.3 Hz, 1H), 7.47 (bs, 1H), 7.34 (t, *J* = 7.7 Hz, 1H), 1.99 (s, 3H), 1.33 (s, 12H). ^13^C NMR (101 MHz, CDCl_3_) δ 151.13, 136.99, 131.15, 128.78, 125.76, 123.24, 84.44, 84.11, 75.51, 24.99, 3.86. TLC-MS (ESI) *m*/*z*: 308.2 [M + Na]^+^; 340.3 [M + Na + MeOH]^+^; 283.9 [M – H]^−^. HPLC *t*_ret_ = 10.74 min. (Method B).

##### 4.1.6.66 3-Bromo-4-fluoroaniline (**26**)

A solution of 100 mg (0.45 mmol) of **25** in 8.0 mL of EtOH was heated to 75 °C. Then a solution of 382 mg of SnCl_2_ (2.02 mmol) in 2.5 mL of HCl (37%, aq.) was added dropwise to the mixture. After 30 min, a reaction control by TLC showed complete consumption of **25**. The volatiles were evaporated giving 85 mg (>99%) of the title compound as a yellowish solid that was used without further purification in the following step. ^1^H NMR (400 MHz, CDCl_3_) δ 6.93–6.81 (m, 2H), 6.59–6.50 (m, 1H), 3.58 (bs, 2H). ^13^C NMR (101 MHz, CDCl_3_) δ 152.75 (d, *J* = 237.1 Hz), 143.54 (d, *J* = 2.5 Hz), 119.27, 116.79 (d, *J* = 23.4 Hz), 115.20 (d, *J* = 6.4 Hz), 109.16 (d, *J* = 21.9 Hz). TLC-MS (ESI) *m*/*z*: 211.4 [M + Na]^+^; 188.6 [M – H]^−^. HPLC *t*_ret_ = 8.06 min. (Method B).

##### 4.1.6.67 4-Methyl-3-(4,4,5,5-tetramethyl-1,3,2-dioxaborolan-2-yl)­aniline (**27**)

The preparation was carried out following *General Procedure A* starting from 100 mg of **24** (0.54 mmol) in 2.0 mL of dry dioxane. Flash purification by eluting with hexane/EtOAc (gradient: 10–20% of EtOAc) afforded 55 mg (44%) of the title compound as a brown oil. ^1^H NMR (400 MHz, CDCl_3_) δ 7.13 (d, *J* = 2.7 Hz, 1H), 6.97 (d, *J* = 8.1 Hz, 1H), 6.69 (dd, *J* = 8.1, 2.7 Hz, 1H), 3.51 (bs, 2H), 2.43 (s, 3H), 1.34 (s, 12H). ^13^C NMR (101 MHz, CDCl_3_) δ 143.27, 134.90, 130.77, 122.60, 118.02, 83.47, 24.99, 21.25. TLC-MS (ESI) *m*/*z*: 234.2 [M + H]^+^. HPLC *t*_ret_ = 7.66 min. (Method B).

##### 4.1.6.68 4-Fluoro-3-(4,4,5,5-tetramethyl-1,3,2-dioxaborolan-2-yl)­aniline (**28**)

The preparation was carried out following *General Procedure A* starting from 500 mg of **26** (2.65 mmol) in 10.0 mL of dry dioxane. Flash purification by eluting with hexane/EtOAc (gradient: 10–25% of EtOAc) afforded 270 mg (43%) of the title compound as a brown oil. ^1^H NMR (400 MHz, CDCl_3_) δ 7.15 (dd, *J* = 3.4, 1.9 Hz, 1H), 6.91–6.86 (m, 2H), 4.48 (s, 2H), 1.35 (s, 12H). ^13^C NMR (101 MHz, CDCl_3_) δ 162.04 (d, *J* = 244.7 Hz), 139.14, 123.92 (d, *J* = 8.0 Hz), 121.34 (d, *J* = 8.2 Hz), 116.14 (d, *J* = 26.4 Hz), 84.10, 24.94. TLC-MS (ESI) *m*/*z*: 260.7 [M + Na]^+^. HPLC *t*_ret_ = 7.78 min. (Method B).

##### 4.1.6.69 *N*-(4-Methyl-3-(4,4,5,5-tetramethyl-1,3,2-dioxaborolan-2-yl)­phenyl)­acrylamide (**29**)

The reaction was carried out following *General Procedure B* starting from 60 mg of **27** (0.26 mmol) in 1.5 mL of dry DCM. Flash purification by eluting with hexane/EtOAc + 20% MeOH (gradient: 6–9% of EtOAc + 20% MeOH) afforded 68 mg (91%) of the title compound as a yellowish solid. ^1^H NMR (400 MHz, CDCl_3_) δ 7.87 (d, *J* = 6.7 Hz, 1H), 7.65–7.59 (m, 1H), 7.36 (s, 1H), 7.15 (d, *J* = 8.3 Hz, 1H), 6.40 (dd, *J* = 16.8, 1.3 Hz, 1H), 6.21 (dd, *J* = 16.9, 10.2 Hz, 1H), 5.72 (dd, *J* = 10.2, 1.2 Hz, 1H), 2.49 (s, 3H), 1.33 (s, 12H). ^13^C NMR (101 MHz, CDCl_3_) δ 163.50, 141.29, 134.79, 131.46, 130.70, 127.46, 127.13, 122.95, 83.74, 25.03, 21.71. TLC-MS (ESI) *m*/*z*: 288.2 [M + H]^+^; 310.2 [M + Na]^+^. HPLC *t*_ret_ = 11.49 min. (Method B).

##### 4.1.6.70 *N*-(4-Fluoro-3-(4,4,5,5-tetramethyl-1,3,2-dioxaborolan-2-yl)­phenyl)­acrylamide (**30**)

The reaction was carried out following *General Procedure B* starting from 270 mg of **28** (1.14 mmol) in 7.0 mL of DCM. Flash purification by eluting with hexane/EtOAc (gradient: 20–30% of EtOAc) afforded 199 mg (60%) of the title compound as a yellowish solid. ^1^H NMR (400 MHz, CDCl_3_) δ 7.97–7.79 (m, 1H), 7.74–7.63 (m, 1H), 7.49 (s, 1H), 7.01 (t, *J* = 8.8 Hz, 1H), 6.41 (dd, *J* = 16.9, 1.3 Hz, 1H), 6.22 (dd, *J* = 16.9, 10.2 Hz, 1H), 5.75 (dd, *J* = 10.2, 1.1 Hz, 1H), 1.34 (s, 12H). ^13^C NMR (101 MHz, CDCl_3_) δ 163.95 (d, *J* = 249.4 Hz), 163.63, 133.49, 131.11, 128.17 (d, *J* = 8.4 Hz), 127.97, 125.62 (d, *J* = 8.8 Hz), 115.99 (d, *J* = 25.5 Hz), 84.25, 24.95. TLC-MS (ESI) *m*/*z*: 314.0 [M + Na]^+^; 289.8 [M – H]^−^. HPLC *t*_ret_ = 10.29 min. (Method B).

##### 4.1.6.71 *N*-(3-Bromo-1-((2-(trimethylsilyl)­ethoxy)­methyl)-1*H*-pyrrolo­[2,3-*b*]­pyridin-5-yl)­cyclopentanecarboxamide (**31**)

The reaction was carried out following *General Procedure D* using 500 mg of **19** (1.46 mmol) in 9.0 mL of dry DCM. Flash purification by eluting with hexane/EtOAc (gradient: 20–40% of EtOAc) afforded 568 mg (88%) of the title compound as a yellow oil. ^1^H NMR (400 MHz, CDCl_3_) δ 8.33 (d, *J* = 2.2 Hz, 1H), 8.31 (s, 1H), 7.58 (s, 1H), 7.36 (s, 1H), 3.56–3.49 (m, 2H), 2.76 (p, *J* = 8.1 Hz, 1H), 2.05–1.87 (m, 4H), 1.80 (qt, *J* = 10.9, 4.8 Hz, 2H), 1.65 (ddt, *J* = 12.3, 9.5, 4.0 Hz, 2H), 1.25 (s, 2H), 0.95–0.85 (m, 2H), −0.06 (s, 9H). ^13^C NMR (101 MHz, CDCl_3_) δ 175.46, 143.11, 136.68, 129.87, 128.23, 121.12, 120.61, 90.47, 73.57, 66.71, 46.69, 30.75, 26.23, 17.92, −1.30. TLC-MS (ESI) *m*/*z*: 460.3 [M + Na]^+^; 436.1 [M – H]^−^. HPLC *t*_ret_ = 13.07 min. (Method B).

##### 4.1.6.72 (*E*)-*N*-(3-(3-(But-2-enamido)­phenyl)-1-((2-(trimethylsilyl)­ethoxy)­methyl)-1*H*-pyrrolo­[2,3-*b*]­pyridin-5-yl)­cyclopentanecarboxamide (**32a**)

The preparation was carried out following *General Procedure E* starting from 118 mg of **31** (0.26 mmol), **22** (0.39 mmol) and 0.6 mL of K_2_CO_3_ (0.5 M, aq.). The reaction was carried out at room temperature. Flash purification by eluting with hexane/EtOAc (gradient: 30–40% of EtOAc) afforded 79 mg (56%) of the title compound as a yellow solid. ^1^H NMR (400 MHz, CDCl_3_) δ 8.26 (d, *J* = 2.2 Hz, 1H), 8.21 (d, *J* = 2.2 Hz, 1H), 8.05 (s, 1H), 7.91 (s, 1H), 7.63 (s, 1H), 7.31 (d, *J* = 6.4 Hz, 1H), 7.13–7.05 (m, 2H), 6.87–6.72 (m, 1H), 5.86 (dd, *J* = 15.1, 1.6 Hz, 1H), 5.42 (s, 2H), 3.45–3.34 (m, 2H), 2.64–2.51 (m, 1H), 1.85–1.70 (m, 4H), 1.67 (dd, *J* = 6.9, 1.5 Hz, 3H), 1.64–1.50 (m, 3H), 1.51–1.34 (m, 2H), 0.79–0.70 (m, 2H), −0.23 (s, 9H). ^13^C NMR (101 MHz, CDCl_3_) δ 175.73, 164.69, 145.86, 141.50, 138.80, 137.68, 135.09, 129.52, 129.25, 126.14, 125.71, 122.65, 120.94, 118.49, 118.36, 118.16, 116.02, 73.20, 66.49, 46.54, 30.74, 26.19, 24.96, 17.92, −1.31. TLC-MS (ESI) *m*/*z*: 519.6 [M + H]^+^; 541.5 [M + Na]^+^; 517.6 [M – H]^−^. HPLC *t*_ret_ = 10.37 min. (Method A).

##### 4.1.6.73 *N*-(3-(3-(But-2-ynamido)­phenyl)-1-((2-(trimethylsilyl)­ethoxy)­methyl)-1*H*-pyrrolo­[2,3-*b*]­pyridin-5-yl)­cyclopentanecarboxamide (**32b**)

The preparation was carried out following *General Procedure E* starting from 102 mg of **31** (0.23 mmol), **23** (0.35 mmol) and 0.5 mL of K_2_CO_3_ (0.5 M, aq.). The reaction was carried out at room temperature. Flash purification by eluting with hexane/EtOAc (gradient: 30–40% of EtOAc) afforded 32 mg (27%) of the title compound as a yellow solid. ^1^H NMR (400 MHz, CDCl_3_) δ 8.55 (s, 1H), 8.35 (s, 1H), 7.85 (s, 1H), 7.77 (s, 1H), 7.59–7.51 (m, 2H), 7.47 (s, 1H), 7.37 (d, *J* = 3.9 Hz, 2H), 5.66 (s, 2H), 3.61–3.52 (m, 2H), 2.81–2.68 (m, 1H), 2.00 (s, 3H), 1.98–1.90 (m, 4H), 1.78 (d, *J* = 16.9 Hz, 2H), 1.69–1.50 (m, 2H), 0.93 (d, *J* = 8.3 Hz, 2H), −0.06 (s, 9H). ^13^C NMR (101 MHz, CDCl_3_) δ 175.30, 151.36, 146.01, 138.12, 137.46, 135.44, 129.82, 129.28, 126.36, 123.38, 120.73, 118.42, 118.29, 118.01, 116.00, 84.73, 75.62, 73.26, 66.55, 46.79, 30.75, 26.21, 14.25, 3.93, −1.28. TLC-MS (ESI) *m*/*z*: 517.5 [M + H]^+^; 539.5 [M + Na]^+^; 515.4 [M – H]^−^. HPLC *t*_ret_ = 10.11 min. (Method A).

##### 4.1.6.74 *N*-(3-(5-Acrylamido-2-methylphenyl)-1-((2-(trimethylsilyl)­ethoxy)­methyl)-1*H*-pyrrolo­[2,3-*b*]­pyridin-5-yl)­cyclopentanecarboxamide (**32c**)

The preparation was carried out following *General Procedure E* starting from 51 mg of **31** (0.12 mmol), **29** (0.18 mmol) and using 0.25 mL of K_2_CO_3_ (0.5 M, aq.). The reaction was carried out at room temperature. Flash purification by eluting with DCM/MeOH (gradient: 0.5–1.5% of MeOH) afforded 79 mg (56%) of the title compound as a yellow solid. ^1^H NMR (400 MHz, CDCl_3_) δ 8.35–8.27 (m, 1H), 8.13 (s, 1H), 7.66 (s, 1H), 7.56 (d, *J* = 7.9 Hz, 1H), 7.46 (d, *J* = 7.2 Hz, 2H), 7.32 (s, 1H), 7.24 (d, *J* = 8.3 Hz, 1H), 6.40 (dd, *J* = 16.9, 1.5 Hz, 1H), 6.26 (dd, *J* = 16.7, 10.2 Hz, 1H), 5.75–5.69 (m, 2H), 5.68 (s, 1H), 3.64–3.52 (m, 2H), 2.79–2.63 (m, 1H), 2.27 (s, 3H), 1.91 (dq, *J* = 13.3, 6.7 Hz, 4H), 1.82–1.66 (m, 2H), 1.66–1.54 (m, 2H), 0.97–0.87 (m, 2H), −0.06 (s, 9H). ^13^C NMR (101 MHz, CDCl_3_) δ 175.32, 163.79, 145.50, 137.52, 135.75, 133.83, 132.91, 131.45, 131.27, 128.89, 127.60, 127.51, 122.10, 121.19, 119.87, 119.19, 115.41, 73.28, 66.49, 46.69, 30.72, 26.18, 20.37, 17.98, −1.28. TLC-MS (ESI) *m*/*z*: 541.5 [M + Na]^+^; 517.4 [M – H]^−^. HPLC *t*_ret_ = 12.77 min. (Method B).

##### 4.1.6.75 *N*-(3-(5-Acrylamido-2-fluorophenyl)-1-((2-(trimethylsilyl)­ethoxy)­methyl)-1*H*-pyrrolo­[2,3-*b*]­pyridin-5-yl)­cyclopentanecarboxamide (**32d**)

The preparation was carried out following *General Procedure E* starting from 100 mg of **31** (0.23 mmol), **30** (0.35 mmol) and 0.5 mL of K_2_CO_3_ (0.5 M, aq.). The reaction was carried out at room temperature. Flash purification by eluting with hexane/EtOAc (gradient: 20–50% of EtOAc) yielded 58 mg (48%) of the title compound as a yellow solid. ^1^H NMR (400 MHz, CDCl_3_) δ 8.47 (s, 1H), 8.35 (s, 1H), 8.35 (s, 1H), 7.89–7.74 (m, 2H), 7.65 (d, *J* = 2.2 Hz, 1H), 7.62–7.53 (m, 1H), 7.06 (t, *J* = 9.6 Hz, 1H), 6.42 (dd, *J* = 16.9, 1.7 Hz, 1H), 6.31 (dd, *J* = 16.8, 10.0 Hz, 1H), 5.72 (dd, *J* = 10.0, 1.7 Hz, 1H), 5.65 (s, 2H), 3.63–3.52 (m, 2H), 2.75 (p, *J* = 8.1 Hz, 1H), 1.93 (dq, *J* = 13.2, 6.7 Hz, 4H), 1.84–1.69 (m, 2H), 1.69–1.55 (m, 2H), 0.97–0.87 (m, 2H), −0.06 (s, 9H). ^13^C NMR (101 MHz, CDCl_3_) δ 175.65, 164.03, 156.31 (d, *J* = 245.0 Hz), 145.52, 137.52, 134.48, 131.32, 129.20, 129.12 (d, *J* = 8.3 Hz), 127.75, 122.29 (d, *J* = 14.4 Hz), 121.08, 120.85 (d, *J* = 3.9 Hz), 119.48 (d, *J* = 5.7 Hz), 118.72, 116.44 (d, *J* = 24.2 Hz), 109.07, 73.35, 66.60, 46.69, 30.77, 26.21, 17.93, −1.30. TLC-MS (ESI) *m*/*z*: 545.4 [M + Na]^+^; 521.4 [M – H]^−^. HPLC *t*_ret_ = 10.47 min. (Method A).

##### 4.1.6.76 5-Chloro-*N*-(3-(3-propionamidophenyl)-1*H*-pyrrolo­[2,3-*b*]­pyridin-5-yl)­thiophene-2-carboxamide (**33**)

The preparation was carried out following *General Procedure F* starting from 60 mg of **37** (0.11 mmol). Flash purification by eluting with DCM/MeOH (gradient: 1–5% of MeOH) afforded 29 mg (63%) of the title compound as a white solid. ^1^H NMR (400 MHz, DMSO) δ 11.93 (s, 1H), 10.46 (s, 1H), 9.93 (s, 1H), 8.50 (s, 2H), 7.93 (d, *J* = 4.1 Hz, 1H), 7.87 (s, 1H), 7.79 (d, *J* = 2.5 Hz, 1H), 7.56 (d, *J* = 7.5 Hz, 1H), 7.41–7.25 (m, 3H), 2.35 (q, *J* = 7.5 Hz, 2H), 1.10 (t, *J* = 7.5 Hz, 3H). ^13^C NMR (101 MHz, DMSO) δ 172.51, 159.55, 146.77, 140.39, 139.50, 138.47, 135.73, 134.25, 129.68, 129.47, 128.82, 128.70, 125.09, 121.56, 120.82, 117.34, 117.21, 117.09, 114.93, 30.05, 10.16. TLC-MS (ESI) *m*/*z*: 447.1 [M + Na]^+^; 423.3 [M – H]^−^. HRMS ESI-TOF [M + H]^+^ *m*/*z* calculated for C_21_H_17_ClN_4_O_2_S: 425.0833 found: 425.0829. HPLC *t*_ret_ = 8.41 min. (Method C); purity: 95.3% (254.4 nm), 93.9% (230.4 nm).

##### 4.1.6.77 *N*-(3-(2-Methyl-5-propionamidophenyl)-1*H*-pyrrolo­[2,3-*b*]­pyridin-5-yl)­cyclopentanecarboxamide (**34**)

The preparation was carried out following *General Procedure F* starting from 50 mg of **38** (0.096 mmol). Flash purification by eluting with DCM/MeOH (gradient: 1–5% of MeOH) afforded 32 mg (85%) of the title compound as a white solid. ^1^H NMR (400 MHz, DMSO) δ 11.73 (s, 1H), 9.90 (s, 1H), 9.82 (s, 1H), 8.38 (d, *J* = 2.3 Hz, 1H), 8.11 (d, *J* = 2.3 Hz, 1H), 7.57–7.52 (m, 2H), 7.50 (d, *J* = 2.4 Hz, 1H), 7.23 (d, *J* = 8.9 Hz, 1H), 2.77 (p, *J* = 7.9 Hz, 1H), 2.30 (q, *J* = 7.5 Hz, 2H), 2.20 (s, 3H), 1.85 (ddt, *J* = 11.8, 6.2, 2.8 Hz, 2H), 1.77–1.62 (m, 4H), 1.56 (ddd, *J* = 12.4, 9.8, 4.8 Hz, 2H), 1.07 (t, *J* = 7.5 Hz, 3H). ^13^C NMR (101 MHz, DMSO) δ 174.10, 171.55, 144.82, 137.01, 136.01, 133.77, 130.28, 130.01, 129.22, 124.99, 120.47, 117.81, 117.14, 113.57, 44.86, 29.88, 29.27, 25.47, 19.67, 9.46. TLC-MS (ESI) *m*/*z*: 413.3 [M + Na]^+^; 389.3 [M – H]^−^. HRMS ESI-TOF [M + H]^+^ *m*/*z* calculated for C_23_H_26_N_4_O_2_: 391.2129 found: 391.2121. HPLC *t*_ret_ = 7.69 min. (Method C); purity: 97.8% (254.4 nm), 97.2% (230.4 nm).

##### 4.1.6.78 (3-Propionamidophenyl)­boronic Acid (**36**)

The reaction was carried out following *General Procedure B* starting from 200 mg of 3-aminophenyl boronic acid **35** (1.5 mmol) and propionyl chloride (0.15 mg, 1.6 mmol, 1.1 equiv) in 10.0 mL of dry DCM. Upon complete consumption of the starting material, the reaction mixture was concentrated under reduced pressure and the remaining residue was taken into ethyl acetate and washed with saturated aqueous sodium chloride solution. Afterward, the organic phase was dried over sodium sulfate and the solvent was evaporated to afford 155 mg (55%) of the title compound as a white solid, which was used without further purification in the following step. ^1^H NMR (400 MHz, CDCl_3_) δ 7.76 (s, 1H), 7.61 (s, 1H), 7.50–7.33 (m, 2H), 7.27–7.14 (m, 1H), 2.95 (s, 2H), 2.32 (q, *J* = 7.6 Hz, 2H), 1.17 (t, *J* = 7.6 Hz, 3H). ^13^C NMR (101 MHz, CDCl_3_) δ 173.62, 137.51, 130.06, 128.73, 125.56, 122.44, 30.76, 10.05. HPLC *t*_ret_ = 6.28 min. (Method B).

##### 4.1.6.79 *N*-(4-Methyl-3-(4,4,5,5-tetramethyl-1,3,2-dioxaborolan-2-yl)­phenyl)­propionamide (**37**)

The reaction was carried out following *General Procedure B* starting from 300 mg of **27** (1.3 mmol) and propionyl chloride (0.13 mg, 1.4 mmol, 1.1 equiv) in 5.0 mL of dry DCM. Flash purification by eluting with hexane/EtOAc (gradient: 0–30% of EtOAc) yielded 224 mg (60%) of the title compound as a yellowish solid. ^1^H NMR (400 MHz, CDCl_3_) δ 7.78 (dd, *J* = 8.3, 2.5 Hz, 1H), 7.54 (d, *J* = 2.5 Hz, 1H), 7.13 (m, 2H), 2.48 (s, 3H), 2.35 (q, *J* = 7.6 Hz, 2H), 1.33 (s, 12H), 1.23 (t, *J* = 7.6 Hz, 3H). ^13^C NMR (101 MHz, CDCl_3_) δ 171.78, 140.77, 134.89, 130.50, 126.82, 122.79, 83.58, 30.68, 24.90, 21.52, 9.70. TLC-MS (ESI) *m*/*z*: 312.3 [M + Na]^+^; 292.2 [M – H]^−^. HPLC *t*_ret_ = 11.25 min. (Method B).

##### 4.1.6.80 5-Chloro-*N*-(3-(3-propionamidophenyl)-1-((2-(trimethylsilyl)­ethoxy)­methyl)-1*H*-pyrrolo­[2,3-*b*]­pyridin-5-yl)­thiophene-2-carboxamide (**38**)

The preparation was carried out following *General Procedure E* starting from 60 mg of **20i** (0.12 mmol), **36** (0.18 mmol) and using 0.10 mL of K_2_CO_3_ (0.5 M, aq.). The reaction was carried out at 60 °C. Flash purification by eluting with hexane/EtOAc (gradient: 50–100% of EtOAc) afforded 67 mg (99%) of the title compound as a white solid. ^1^H NMR (400 MHz, DMSO) δ 10.52 (s, 1H), 9.95 (s, 1H), 8.58 (d, *J* = 2.2 Hz, 1H), 8.55 (d, *J* = 2.3 Hz, 1H), 7.97 (s, 1H), 7.94 (s, 1H), 7.93 (s, 1H), 7.56 (dt, *J* = 8.3, 1.6 Hz, 1H), 7.39 (s, 1H), 7.35–7.29 (m, 2H), 5.67 (s, 2H), 3.62–3.55 (m, 2H), 2.35 (q, *J* = 7.5 Hz, 2H), 1.10 (t, *J* = 7.6 Hz, 3H), 0.85 (m, 2H), −0.09 (s, 9H). ^13^C NMR (101 MHz, CDCl3) δ 173.38, 160.05, 145.36, 138.54, 137.79, 137.27, 135.89, 134.39, 129.19, 128.76, 128.03, 127.00, 125.79, 121.71, 120.96, 118.24, 117.87, 117.42, 115.72, 72.90, 66.15, 30.02, 17.47, 9.34, −1.87. TLC-MS (ESI) *m*/*z*: 576.8 [M + Na]^+^; 554.5 [M – H]^−^. HPLC *t*_ret_ = 13.49 min. (Method B).

##### 4.1.6.81 *N*-(3-(2-Methyl-5-propionamidophenyl)-1-((2-(trimethylsilyl)­ethoxy)­methyl)-1*H*-pyrrolo­[2,3-*b*]­pyridin-5-yl)­cyclopentanecarboxamide (**39**)

The preparation was carried out following *General Procedure E* starting from 40 mg of **31** (0.082 mmol), **37** (0.14 mmol) and using 0.07 mL of K_2_CO_3_ (0.5 M, aq.). The reaction was carried out at 60 °C. Flash purification by eluting with hexane/EtOAc (gradient: 10–30% of EtOAc) afforded 62 mg (86%) of the title compound as a yellow solid. ^1^H NMR (400 MHz, DMSO) δ 9.96 (s, 1H), 9.83 (s, 1H), 8.44 (d, *J* = 2.3 Hz, 1H), 8.15 (d, *J* = 2.3 Hz, 1H), 7.69 (s, 1H), 7.57 (d, *J* = 2.3 Hz, 1H), 7.54 (dd, *J* = 8.2, 2.3 Hz, 1H), 7.25 (d, *J* = 8.2 Hz, 1H), 5.64 (s, 2H), 3.56 (dd, *J* = 8.4, 7.5 Hz, 2H), 2.78 (p, *J* = 8.0 Hz, 1H), 2.31 (q, *J* = 7.6 Hz, 2H), 2.20 (s, 3H), 1.85 (dt, *J* = 8.3, 5.5 Hz, 2H), 1.75–1.63 (m, 4H), 1.57–1.51 (m, 2H), 1.07 (t, *J* = 7.5 Hz, 3H), 0.83 (dd, *J* = 8.5, 7.4 Hz, 2H), −0.11 (s, 9H). TLC-MS (ESI) *m*/*z*: 543.3 [M + Na]^+^; 519.4 [M – H]^−^. HPLC *t*_ret_ = 13.63 min. (Method B).

### 4.2 Molecular Modeling

#### 4.2.1 Model Generation and Structure Preparation

The cocrystallized structure of BTK in the DFG-in conformation with ibrutinib (PDB 5P9J^43^) was selected for modeling because of its high resolution (1.08 Å). However, for BMX, no example of a DFG-in conformation was available in the PDB. Therefore, the structure with PDB 6I99 ^31^ was chosen due to its high resolution (2 Å) and the presence of a covalently bound ligand. This structure displays a DFG-intermediate conformation (DFG-inter), representing a state between the classical DFG-in and -out conformation), while maintaining Type I inhibition. In the DFG-inter, the phenylalanine side chain is out of the αC-helix pocket but has not moved completely to a DFG-out conformation.^44^ Other available structures, though with worse resolution, such as a dasatinib-bound BMX structure (PDB 3SXR ^3^) also displays this DFG-inter, with a Type I inhibition configuration. Considering the broader range of kinase conformations based on the Ramachandran regions (A, α; B, β; L, left) of the XDF motif and the Phe rotamer (minus, plus, trans), it becomes evident that the noncovalent inhibitor dasatinib binds to different conformations of the same kinase across various structural entries.^44^ For instance, dasatinib interacts with ABL1 in BLA-minus (active conformation) and BAB-trans states (DFG-inter), while engaging BTK in BLA-plus (active conformation) and BAB-trans conformations. This observation justifies our choice of structures to generate this more diverse model followed by MD simulations.

We modeled the systems with Maestro (Schrödinger Release 2023.2 Maestro, Schrödinger, LLC, New York, NY, 2023), and OPLS4 force-field.^45^ BTK (UniProt Q06187, residues: Gln380-Ser529) and BMX (UniProt P51813, residues: Glu411-His675) ligand binding domain models were generated using their ligand-bound structures (PDB ID: 5P9J,^43^ and 6I99 chain B,^31^ respectively) as templates utilizing the Advanced homology modeling tool in Schrödinger suite (v2023.2). Missing amino acid’s side chains were placed using Prime, followed by loop refinement using the same software.^46^ Proteins were prepared using Protein Preparation Wizard (Schrödinger LLC, New York, NY, 2023). Missing hydrogen atoms were added, bond orders were assigned using CCD database, and protonation states of amino acids were optimized with PROPKA (Schrödinger, LLC, New York, NY, 2023) at pH 7.4. Given the truncated nature of our models, both N and C-termini were capped. Final structures were optimized in terms of hydrogen positions, utilizing the H-bond assignment algorithm PROPKA in the Protein Preparation Wizard tool of Maestro, to select the most likely protonation states and tautomer for the Histidine residues. We agreed with the software suggestions, followed by optimizing the generated H-bonding species. Finally, all structures underwent global energy minimization using the steep descent method.

#### 4.2.2 Molecular Docking and Pose Selection

Prior to docking, ligands were prepared using LigPrep (Schrödinger, LLC, New York, NY, 2023) to assign the protonation state (Epik; at pH 7.0 ± 1.0) and the partial charges. The starting conformation of noncovalently bound ligands were generated using docking (Glide v7.7) using extra precision (XP).^47,48^ Docking was calculated within a pocket defined residues with 13 Å around cocrystallized ligands of the original templates for the binding site, generating up to 10 poses per ligand. Representative docking poses were selected not only based on the glide docking score, but also in terms of visual inspection of the warhead position in regard of the covalently prone cysteine, also aiming to similar poses for congeners ligands. Covalent bound was generated by manually editing the Michael acceptor warhead and locally minimizing the structure. Final conformations were submitted to molecular dynamics (MD) simulations.

#### 4.2.3 Molecular Dynamics Simulations

We simulated monomeric BMX and BTK models with ligands using the Desmond MD simulation engine^49^ and the OPLS4 force-field.^45^ The prepared systems were solvated in a cubic box with the size of the box set as 13 Å minimum distance from the box edges to any atom of the protein. TIP3P water model^50^ was used to describe the solvent and the net charge was neutralized using Na^+^ ion. RESPA integrator timesteps of 2 fs for bonded and near and 6 fs for far were applied. The short-range Coulombic interactions were treated using a cutoff value of 9.0 Å, whereas long-range Coulombic interactions were estimated using the Smooth Particle Mesh Ewald (PME) method.^51^ Before the production simulations, systems were relaxed using the default Desmond relaxation protocol. Simulations were run in NPT ensemble, with a temperature of 310 K (using the Nosé-Hoover thermostat^52,53^ ) and pressure of 1.01325 bar (Martyna-Tobias-Klein barostat^54^). For each system, five independent simulations of at least 1 μs were carried out, resulting in 5 μs simulation data for each system. All raw trajectories, calculated energy, distances and protein–ligand interactions are publicly available in the repository Zenodo under the following DOIs: 10.5281/zenodo.8238075.

#### 4.2.4 Analysis of MD Simulation Trajectories

##### 4.2.4.1 Protein–Ligand Interactions and Protein Properties

Maestro simulation interaction analysis tool (Schrödinger, LLC) was used for the analysis of RMSD, RMSF, interaction and torsional profile analyses. We used default values for interaction which are H-bonds: cutoff of 2.5 Å for donor and acceptor atoms, donor angle of 120° and acceptor angle of 90°. Hydrophobic interactions: cutoff of 3.6 Å between ligand’s aromatic or aliphatic carbons and a hydrophobic side chain, π–π interaction was defined as two aromatic groups stacked face-to-face or face-to-edge. Water bridge interactions: default cutoff of 2.8 Å for donor and acceptor atoms, donor angle of 110° and acceptor angle of 90°. For angle and distance calculations, the Maestro event analysis tool (Schrödinger, LLC) was used.

##### 4.2.4.2 Distances and Angle Calculations

Distances between specific secondary structure elements were calculated using their centers of mass with the Maestro script trj_asl_distance.py (Schrödinger LLC). trajectories.

##### 4.2.4.3 MM-GBSA Binding Energy Calculations

Molecular mechanics with generalized Born and surface area (MM-GBSA) predicts the binding free energy of protein–ligand complexes.^55^ The ligands’ ranking based on the free energy could be correlated to the experimental binding affinities, especially in a congeneric series. Every 10th frame from the simulations was considered for the calculations. These were used as input files for the MM-GBSA calculations with thermal_mmgbsa.py script from Schrödinger package. Calculated free-binding energies (kcal/mol) are represented by the MM-GBSA and normalized by the number of heavy atoms (HAC), according to the following formula: Ligand Efficiency = (Binding energy)/(1 + Ln­(HAC)).

#### 4.2.5 QM Conformation Calculations

We used the QM Conformer & Tautomer Predictor tool of Maestro (2023.2; Schrödinger LLC, New York, NY, USA) to generate ten conformers for each of the relevant in water solvent. This tool uses a stepwise minimization, applying Jaguar^56^ in the final stages in the conformation generation.

### 4.3 Biochemical Evaluation

The assessment of IC_50_ values was conducted by Reaction Biology Corp. in their facilities in Malvern, PA, USA. In the HotSpot assay,^36^ all IC_50_ values were first determined by singlicate measurements starting at a concentration of 5 μM with a 10-fold serial dilution (5-point measurement) and duplicates of key compounds were subsequently determined against BMX and BTK. The residual activity was measured at 250 nM in a single dose duplicate mode with [ATP] = 10 μM and staurosporine as control.

### 4.4 Expression and Purification of Recombinant BMX Kinase Domain (K264–H675)

Transformed BL21­(DE3) cells were grown in Terrific Broth supplemented with 50 μg/mL kanamycin. Protein expression was induced at an OD600 of approximately 2 by the addition of 0.5 mM IPTG, followed by incubation at 18 °C for 12 h. Cells expressing C-terminally His_6_-tagged BMX were harvested and lysed by sonication in lysis buffer containing 50 mM HEPES (pH 7.5), 500 mM NaCl, 25 mM imidazole, 5% glycerol, and 0.5 mM TCEP. After centrifugation, the clarified lysate was loaded onto a Ni-Sepharose column equilibrated with 30 mL of lysis buffer. The column was washed with 60 mL of the same buffer, and bound protein was eluted using a stepwise imidazole gradient (50, 100, 200, and 300 mM). Fractions containing BMX (assessed by SDS PAGE) were pooled and concentrated to approximately 59 mg/mL.

### 4.5 Differential Scanning Fluorimetry (DSF) Assay

The assessment of the thermal shift (Δ*T*_m_) was conducted as described by Fedorov et al.^57,58^ Purified proteins were buffered in 25 mM HEPES (pH 7.5) and 500 mM NaCl and assessed in a 384-well plate with a final protein concentration of 2 μM in a 10 μL assay volume. Inhibitors were added to a final concentration of 10 μM, using an ECHO 550 acoustic dispenser (Labcyte). SYPRO-Orange (Fluorescence Probes) was added in a 1:5000 dilution. Excitation and emission filters were set to 465 and 590 nm, respectively. Slow temperature increase was performed from 25 °C at a rate of 3 °C/min to a final temperature of 95 °C with simultaneous scanning, using QuantStudio5 (Applied Biosystems). Data was analyzed via the Boltzmann equation in the Protein Thermal Shift software (Applied Biosystems). Samples were measured in technical duplicates.

### 4.6 Cellular NanoBRET Assay

Full length BMX and BTK was cloned into pFC32K (Promega, Madison, WI, USA) for expression of a C-terminal NanoLuc fusion. The plasmids were transfected into HEK293T cells cultured in DMEM (Gibco, Waltham, MA, USA) supplemented with 10% fetal bovine serum (Gibco, Waltham, MA, USA) and Penicillin/Streptamycin (Gibco,Waltham, MA, USA). NanoBRET assays were performed using the protocol published previously.^59^ Briefly, after transfection and 20 h incubation cells were harvested and subsequently resuspended in OptiMEM (Gibco,Waltham, MA, USA). Cells were aliquoted onto 1536-well plates (Greiner, Kremsmünster, Austria), and inhibitors as well as 0.5 μM Tracer K4 (Promega) for BMX or 0.5 μM Tracer K5 (Promega, Madison, WI, USA) for BTK were added using an ECHO acoustic dispenser. The plates were incubated at 37 °C with 5% CO_2_ for 2 h prior to the addition of both NanoBRET NanoGlo substrate (Promega, Madison, WI, USA) and extracellular NanoLuc inhibitor. BRET luminescence (450 nm for donor emission and 610 nm for BRET signal) was measured using PHERAstar FSX plate reader (BMG Labtech, Ortenberg, Germany). Milli-BRET units (mBU) were calculated as a ratio between BRET signal and the overall measured luminescence. A dose–response fitting was applied and IC_50_ values were calculated using the Prism software (GraphPad Prism 11 software using a normalized 3-parameter curve fit with the following equation: *Y* = 100/(1 + 10­(*X*–log IC_50_)). The affinity of both tracer molecules toward BMX and BTK was tested and showed similar values (EC_50_ (Tracer 4, BMX) = 0.240 μM, EC_50_ (Tracer 5, BTK) = 0.231 μM) indicating no bias of the assay due to the higher affinity of one kinase. Experiments were performed as triplicates and repeated at least three times.

### 4.7 Assessment of Binding Kinetics

Compounds **11i**, **12b**, and **12c** were submitted to *k*_inact_/*K*_I_ value determination in a PhosphoSens CSox-based continuous assay format at AssayQuant Technologies, Inc. All inhibitors in this set were able to be fit to a two-step global model, allowing for independent *K*_I_ and *k*_inact_ determination. Reaction conditions: 50 mM HEPES, pH 7.5, [ATP] at *K*_m_, 1.0 mM DTT, 0.01% Brij-35, 0.5 mM EGTA, 1% glycerol (from EDB), 10 mM MgCl_2_, 0.2 mg/mL BSA (from EDB), 15 μM AQT sensor substrate (AQT0101), 1% DMSO. Two nM BMX, full-length (1-675), N-term GST fusion, Carna (cat. # 08-179/Lot: 13CBS-0583 M). Reaction setup: 0.3 μL compound dilution, 3.0 μL 10 × Sox-based Substrate, 3.0 μL 10 × ATP, 30 min incubation at RT (while reaction mix is made), 23.7 μL reaction mix with 1.4 × enzyme or EDB, and all other components (enzyme/EDB added just before use of reaction mix), 30 μL final reaction volume. The reaction was run at 30 °C for 120 min and performed starting from 0 to 0.05, 0.5, or 5 μM (24-point, 1.5-fold dilution). Reads were taken in sweep mode within the runtime.

### 4.8 Mass Spectrometry

BMX protein at a concentration of 59 mg/mL was diluted in MS buffer (20 mM HEPES, 150 mM NaCl, 0.5 mM TCEP) to a final concentration of 20 μM and incubated with 40 μM **11i** from 10 mM DMSO stock (1:2 protein-to-compound ratio) for 90 min at 4 °C. Samples were analyzed by liquid chromatography-coupled mass spectrometry (LC–MS) using a Waters BioAccord system operated with UNIFI 3.1.0 software. Native BMX protein was analyzed as a control. Protein samples were separated on an Acquity BEH C4 column (1.7 μm, 2.1 × 50 mm) using a 6.5 min linear gradient from 5 to 80% acetonitrile in Milli-Q water. Both mobile phases contained 0.1% formic acid. Mass spectra were acquired in positive ion mcode using a cone voltage of 45 V and a capillary voltage of 0.8 kV.

### 4.9 Metabolic Stability in Human Liver Microsomes (HLM)

Pooled liver microsomes from humans (male) were purchased from Sekisui XenoTech, LLC, Kansas City, KS, USA. Metabolic stability assays were performed in the presence of an NADPH-regenerating system consisting of 5 mM glucose-6-phosphate, 5 U/mL glucose-6-phosphate dehydrogenase, and 1 mM NADP^+^. Liver microsomes (20 mg/mL), NADPH-regenerating system, and 4 mM MgCl2·6 H_2_O in 0.1 M TRIS-HCl-buffer (pH 7.4) were preincubated for 5 min at 37 °C and 750 rpm on a shaker. The reaction was started by adding the preheated compound at 10 mM resulting in a final concentration of 0.1 mM. The reaction was quenched at selected time points (0, 10, 20, 30, 60, and 120 min) by pipetting 100 μL of internal standard (ketoprofen) in acetonitrile at concentrations of 500 μM for compounds **11m** and **12b** and 300 μM for compound **12c**. The samples were vortexed for 30 s and centrifuged (21910 relative centrifugal force, 4 °C, 20 min). The supernatant was used directly for LC-MS analysis.

All compound incubations were conducted at least in triplicates. Additionally, a negative control containing BSA (20 mg/mL) instead of liver microsomes and a positive control using Verapamil instead of the compound was performed. A limit of 1% organic solvent during incubation was not exceeded. Sample separation and detection were performed on an Alliance 2695 Separations Module HPLC system (Waters Corporation, Milford, MA, USA) equipped with a Phenomenex Kinetex 2.6 μm XB-C18 100 Å 50 × 3 mm column (Phenomenex Inc., Torrance, CA, USA) coupled to an Alliance 2996 Photodiode Array Detector and a MICROMASS QUATTRO micro API mass spectrometer (both Waters Corporation, Milford, MA, USA) using electrospray ionization in positive mode.

Mobile phase A: 90% water, 10% acetonitrile and additionally 0.1% formic acid (v/v), mobile phase B: 100% acetonitrile with additional 0.1% formic acid (v/v). The gradient was set to 0–2.5 min 0% B, 2.5–10 min from 0 to 50% B, 10–12 min 50% B, 12–12.01 min from 50 to 0% B, 12.01–17 min 0% B at a flow rate of 0.7 mL/min. Samples were maintained at 10 °C, the column temperature was set to 20 °C with an injection volume of 5 μL. Spray, cone, extractor, and RF lens voltages were at 4 kV, 30 V, 8 and 2 V, respectively. The source and desolvation temperatures were set to 120 and 350 °C, respectively, and the desolvation gas flow was set to 750 L/h. Data analysis was conducted using MassLynx 4.1 software (Waters Corporation, Milford, MA, USA).

### 4.10 Glutathione (GSH) Stability Assay

The GSH stability assay for compounds **11i**, **12b**, **12c** and afatinib was based on the GSH assay established by Keeley et al.^60^ The following amendments from the original protocol have been implemented: MeCN/PBS-buffer was used as reaction medium, the reaction temperature was chosen to be 40 °C. GSH-adduct formation was monitored by measuring the decreasing area under the curve (AUC) of the compound relative to the internal standard indoprofen in an HPLC-system.

### 4.11 Statistical Analyses and Image Generations

Unless stated otherwise in the figure legends, data are presented as the mean ± standard deviation (SD). GraphPad Prism 10.1 Software (GraphPad Software, Inc., San Diego, CA, USA) was used to perform statistical analysis, where p-values <0.05 were statistically significant. Structural data visualization was conducted with PyMOL v.2.5.2 (Schrödinger LLC, New York, NY, USA). Data visualization was also completed by Python 3.7, seaborn (v0.12.2), matplotlib^61^ and GraphPad Prism (v. 10.1 for Windows, GraphPad Software, San Diego, CA, USA).

## Supplementary Material





















## Data Availability

All molecular dynamics trajectories and raw data related to the protein–ligand interactions within the simulations were uploaded and are available in the Zenodo repository under the DOI: https://zenodo.org/records/8238075.

## Notes

Molecular formula strings (CSV)
Docking of compound **10** binding to BMK for MD simulations (PDB)
Docking of compound **10** binding to BTX for MD simulations (PDB)
Docking of compound **11i** binding to BMK for MD simulations (PDB)
Docking of compound **11i** binding to BTX for MD simulations (PDB)
Docking of compound **11m** binding to BMK for MD simulations (PDB)
Docking of compound **11m** binding to BTX for MD simulations (PDB)
Docking of compound **12c** binding to BMK for MD simulations (PDB)
Docking of compound **12c** binding to BTX for MD simulations (PDB)
Supplementary figures, data collection, and further information, including tables with data of the thermal shift assessments, NanoBRET assays, and MD simulations; depictions of HLM stability, intact protein mass experiment, and HPLC traces of selected compounds; and NMR spectra of all synthesized compounds (PDF)

## Abbreviations Used

ATP: adenosine triphosphate
AUC: area under the curve
aq.: aqueous
B_2_pin_2_: Bis­(pinacolato)­diboron
BLK: B-lymphoid tyrosine kinase
BMX/ETK: bone marrow tyrosine kinase on chromosome X
br s: broad singlet
BRET: bioluminescence resonance energy transfer
BTK: Bruton’s tyrosine kinase
CDI: 1,10-carbonyldiimidazole
DAD: diode array detector
DCM: dichloromethane
DMEM: Dulbecco’s modified Eagle’s medium
DMF: *N*,*N*-dimethylformamide
DMSO: dimethyl sulfoxide
EC_50_: half maximal effective concentration
EGFR: epidermal growth factor receptor
ESI: electron spray ionization
Et_3_N: triethylamine
EtOAc: ethyl acetate
EtOH: ethanol
GSH: glutathione
HER: human epidermal growth factor receptor
HPLC: high-performance liquid chromatography
IC_50_: half-maximal inhibitory concentration
ITK: interleukin-2-inducible T-cell kinase
JAK: Janus kinase
KOAc: potassium acetate
LC: liquid chromatography
MAP2K7/MKK7: mitogen activated protein kinase kinase 7
MD: molecular dynamics
MeOH: methanol
MES: 2-(*N*-morpholino)­ethanesulfonic acid
MS: mass spectrometry
NaH: sodium hydride
n-BuLi: *n*-butyllithium
NBS: *N*-bromosuccinimide
NMR: nuclear magnetic resonance spectroscopy
Pd/C: palladium on activated carbon
Pd­(OAc)_2_: palladium­(II)­acetate
PEG: polyethylene glycol
PH: pleckstrin homology
rt: room temperature
SAR: structure–activity relationship
sat.: saturated
SEM: 2-(trimethylsilyl)­ethoxymethyl
tBuOH: *tert*-butanol
tBu_3_P Pd G3: mesyl­[(tri-*t*-butylphosphine)-2-(2-aminobiphenyl)]­palladium­(II)
TCEP: tris­(2-carboxyethyl)­phosphine
Δ*T*_m_: thermal shift
*t*_ret_: retention time
TEC: tyrosine kinase expressed in hepatocellular carcinoma
TFA: trifluoroacetic acid
TH: TEC homology
THF: tetrahydrofurane
TLC: thin-layer chromatography
TMS: tetramethylsilane
TOF: time-of-flight
Tris: tris­(hydroxymethyl)­aminomethan
TSK/EMT: interleukin-2-inducible T-cell kinase
TXK/RLK: tyrosine-protein kinase
UV: ultraviolet
XLA: X-linked agammaglobulinemia
XPhos: 2-dicyclohexylphosphino-20,40,60-triisopropyl-1,10-biphenyl
XPhos Pd G3: (2-dicyclohexylphosphino-20,40,60-triisopropyl-1,10-biphenyl)­[2-(20-amino-1,10-biphenyl)]­palladium­(II) methanesulfonate
XPhos Pd G4: (2-dicyclohexylphosphino-20,40,60-triisopropyl-1,10-biphenyl)­[2-(20-*N*-methylamino-1,10-biphenyl)]­palladium­(II) methanesulfonate
