# Supplementary material for: Riding toward Selectivity: Optimization of Covalent 7‑Azaindole-Based BMX Kinase Inhibitors
Source: J Med Chem. 2026 Jul 13;69(14):17416–47. doi: 10.1021/acs.jmedchem.6c01366 (PMC13403314; doi:10.1021/acs.jmedchem.6c01366)
Supplement: Supplementary file 10 [file jm6c01366_si_010.pdf]

Supporting Information

Riding toward Selectivity: Optimization of Covalent 7-Azaindole-Based BMX Kinase Inhibitors

Xiaojun Julia Liang [a,b,c#], Claudia Albertini [c,d#], Thales Kronenberger [c,e,f,g], Ekaterina Shevchenko [c,e,f], Alexander Rasch [c], Benedict-Tilman Berger [h,i], Martin Schwalm [h,i,j], Lena Marie Berger [h,i], Andreas Krämer [h,i,j], Ricardo A. M. Serafim [a,c,k], Michael Forster [b,c], Apirat Chaikuad [h,i], Maria Laura Bolognesi [d], Susanne Müller [h,i,j], Antti Poso [b,c,e,f], Stefan Laufer [b,c,e], Stefan Knapp [h,i,j], and Matthias Gehringer [a,b,c*]

[a] Department for Medicinal Chemistry, Institute for Biomedical Engineering, Faculty of Medicine, University of Tübingen, Auf der Morgenstelle 8, 72076 Tübingen, Germany

[b] Cluster of Excellence iFIT (EXC 2180) 'Image-Guided & Functionally Instructed Tumor Therapies', University of Tübingen, 72076 Tübingen, Germany.

[c] Department of Pharmaceutical/Medicinal Chemistry, Institute of Pharmaceutical Sciences, Faculty of Sciences, University of Tübingen, Auf der Morgenstelle 8, 72076 Tübingen, Germany

[d] Department of Pharmacy and Biotechnology, Alma Mater Studiorum-University of Bologna, Via Belmeloro 6, 40126 Bologna, Italy

[e] Tübingen Center for Academic Drug Discovery & Development (TüCAD$_2$), 72076 Tübingen, Germany.

[f] School of Pharmacy, Faculty of Health Sciences, University of Eastern Finland, 70211, Kuopio, Finland.

[g] Institute of Medical Microbiology and Hygiene, Interfaculty Institute of Microbiology and Infection Medicine, University of Tübingen, Elfriede-Aulhorn-Str. 6, 72076 Tübingen, Germany.

[h] Structural Genomics Consortium, Goethe University Frankfurt, Buchmann Institute for Molecular Life Sciences, Max-von-Laue-Straße 15, 60438 Frankfurt am Main, Germany

[i] Institute of Pharmaceutical Chemistry, Goethe University Frankfurt, Buchmann Institute for Molecular Life Sciences, Max-von-Laue-Straße 9, 60438 Frankfurt am Main, Germany

[j] Frankfurt Cancer Institute (FCI) and German Translational Cancer Network (DKTK) Site Frankfurt/Mainz, 60438 Frankfurt am Main, Germany

[k] Department of Organic and Pharmaceutical Chemistry, School of Engineering, Institut Químic de Sarrià (IQS), Universitat Ramon Llull (URL), Vía Augusta 390, 08017 Barcelona, Spain

[*] Correspondence: matthias.gehringer@uni-tuebingen.de; [#] Equal contribution

Table of contents

Table S 1. BMX, BTK and MKK7 thermal shift assay 4
Table S 2. Protein kinase thermal shift assay with ΔT_m [K] for title compounds **11i**, **11m**, **11n**, **12b** and **12c**.4
Table S 3. Target occupancy [%] of **11i** measured in a NanoBRET® assay against 192 kinases. 7
Table S 4. Polar interaction frequency [%] along the MD simulation trajectory. 8
Table S 5. MM-GBSA derived ΔG binding free energy. 9
Figure S 1. QM calculated conformations and their Boltzmann population frequency 10
Figure S 2. Human liver microsomal stability of compounds **11m**, **12b** and **12c**. 11
Figure S 3. Intact protein MS experiment of BMX with **11i**. 12
Figure S 4. HPLC spectrum traces of **11i**. 12
Figure S 5. HPLC spectrum traces of **11m**. 13
Figure S 6. HPLC spectrum traces of **12b** 13
Figure S 7. HPLC spectrum traces of **12c** 14
Figure S 8. ^{1}H-NMR spectrum of **11a** in DMSO- d_6. 15
Figure S 9. ^{13}C-NMR spectrum of **11a** in DMSO- d_6. 15
Figure S 10. ^{1}H-NMR spectrum of **11b** in DMSO- d_6. 16
Figure S 11. ^{13}C-NMR spectrum of **11b** in DMSO- d_6. 16
Figure S 12. ^{1}H-NMR spectrum of **11c** in DMSO- d_6. 17
Figure S 13. ^{13}C-NMR spectrum of **11c** in DMSO- d_6. 17
Figure S 14. ^{1}H-NMR spectrum of **11d** in DMSO- d_6. 18
Figure S 15. ^{13}C-NMR spectrum of **11d** in DMSO- d_6. 18
Figure S 16. ^{1}H-NMR spectrum of **11e** in DMSO- d_6. 19
Figure S 17. ^{13}C-NMR spectrum of **11e** in DMSO- d_6. 19
Figure S 18. ^{1}H-NMR spectrum of **11f** in DMSO- d_6. 20
Figure S 19. ^{13}C-NMR spectrum of **11f** in DMSO- d_6. 20
Figure S 20. ^{1}H-NMR spectrum of **11g** in DMSO- d_6. 21
Figure S 21. ^{13}C-NMR spectrum of **11g** in DMSO- d_6. 21
Figure S 22. ^{1}H-NMR spectrum of **11h** in DMSO- d_6. 22
Figure S 23. ^{13}C-NMR spectrum of **11h** in DMSO- d_6. 22
Figure S 24. ^{1}H-NMR spectrum of **11i** in DMSO- d_6. 23
Figure S 25. ^{13}C-NMR spectrum of **11i** in DMSO- d_6. 23
Figure S 26. ^{1}H-NMR spectrum of **11j** in DMSO- d_6. 24
Figure S 27. ^{13}C-NMR spectrum of **11j** in DMSO- d_6. 24
Figure S 28. ^{1}H-NMR spectrum of **11k** in DMSO- d_6. 25
Figure S 29. ^{13}C-NMR spectrum of **11k** in DMSO- d_6. 25
Figure S 30. ^{1}H-NMR spectrum of **11l** in DMSO- d_6. 26
Figure S 31. ^{13}C-NMR spectrum of **11l** in DMSO- d_6. 26
Figure S 32. ^{1}H-NMR spectrum of **11m** in DMSO- d_6. 27
Figure S 33. ^{13}C-NMR spectrum of **11m** in DMSO- d_6. 27
Figure S 34. ^{1}H-NMR spectrum of **11n** in DMSO- d_6. 28
Figure S 35. ^{13}C-NMR spectrum of **11n** in DMSO- d_6. 28
Figure S 36. ^{1}H-NMR spectrum of **11o** in DMSO- d_6. 29
Figure S 37. ^{13}C-NMR spectrum of **11o** in DMSO- d_6. 29

Figure S 38. ^{1}H-NMR spectrum of **11p** in DMSO- d_6. 30
Figure S 39. ^{13}C-NMR spectrum of **11p** in DMSO- d_6. 30
Figure S 40. ^{1}H-NMR spectrum of **11q** in DMSO- d_6. 31
Figure S 41. ^{13}C-NMR spectrum of **11q** in DMSO- d_6. 31
Figure S 42. ^{1}H-NMR spectrum of **11r** in DMSO- d_6. 32
Figure S 43. ^{13}C-NMR spectrum of **11r** in DMSO- d_6. 32
Figure S 44. ^{1}H-NMR spectrum of **12a** in DMSO- d_6. 33
Figure S 45. ^{13}C-NMR spectrum of **12a** in DMSO- d_6. 33
Figure S 46. ^{1}H-NMR spectrum of **12b** in DMSO- d_6. 34
Figure S 47. ^{13}C-NMR spectrum of **12b** in DMSO- d_6. 34
Figure S 48. ^{1}H-NMR spectrum of **12c** in DMSO- d_6. 35
Figure S 49. ^{13}C-NMR spectrum of **12c** in DMSO- d_6. 35
Figure S 50. ^{1}H-NMR spectrum of **12d** in DMSO- d_6. 36
Figure S 51. ^{13}C-NMR spectrum of **12d** in DMSO- d_6. 36
Figure S 52. ^{1}H-NMR spectrum of **33** in DMSO- d_6. 37
Figure S 53. ^{13}C-NMR spectrum of **33** in DMSO- d_6. 37
Figure S 54. ^{1}H-NMR spectrum of **34** in DMSO- d_6. 38
Figure S 55. ^{13}C-NMR spectrum of **34** in DMSO- d_6. 38

Table S 1. BMX, BTK and MKK7 thermal shift assay with ΔT_m for title compounds **11 a-r** – **12 a-d** and the selectivity ratio among BMX/BTK and BMX/MKK7.

compound	BMX ΔTm [K][1]	BTK ΔTm [K][1]	MKK7 ΔTm [K][1]	BMX/BTK	BMX/MKK7
**11a**	11.12	7.46	6.83	1.49	1.63
**11b**	10.63	7.23	10.67	1.47	1.00
**11c**	10.14	7.34	6.39	1.38	1.59
**11d**	11.30	8.55	7.40	1.32	1.53
**11e**	11.22	8.20	3.02	1.37	3.71
**11f**	11.21	7.36	8.24	1.52	1.36
**11g**	11.11	7.18	6.85	1.55	1.62
**11h**	9.93	6.79	7.99	1.46	1.24
**11i**	9.53	5.68	4.95	1.68	1.93
**11j**	10.19	6.78	3.28	1.50	3.11
**11k**	11.09	7.01	4.14	1.58	2.68
**11l**	10.77	6.70	5.13	1.61	2.10
**11m**	10.84	6.84	4.62	1.58	2.35
**11n**	10.85	4.79	3.91	2.27	2.78
**11o**	11.03	5.85	4.71	1.88	2.34
**11p**	11.09	6.52	2.31	1.70	4.81
**11q**	8.53	3.47	2.00	2.46	4.27
**11r**	10.58	4.52	1.33	2.34	7.96
**12a**	8.15	6.02	2.81	1.35	2.90
**12b**	7.43	5.31	-0.95	1.40	-7.79
**12c**	10.19	6.16	1.25	1.65	8.13
**12d**	11.91	9.17	4.31	1.30	2.76

[1] Mean of at least three independent measurements.

Table S 2. Protein kinase thermal shift assay with ΔT_m [K] for title compounds **11i**, **11m**, **11n**, **12b** and **12c**.

kinase/compound	11i [ΔT_m]	11m [ΔT_m]	11n [ΔT_m]	12b [ΔT_m]	12c [ΔT_m]
ABL1	0.3	1.1	0.0	4.5	1.5
BMX	5.9	6.7	7.2	4.1	6.5
CAMKK2	2.0	2.0	0.5	2.9	2.9
CASK	0.4	0.7	0.5	1.1	1.9
CSNK1D	0.1	0.3	0.1	1.8	0.1
CSNK2A1	0.0	0.3	-0.4	2.0	0.6
BMPR2	0.4	0.5	0.2	1.1	0.5
CDK2	1.4	1.2	1.0	1.6	0.5
BRAF	0.5	0.8	0.4	2.1	0.6
AAK1	0.1	1.7	-0.3	2.8	-0.2
CAMK2B	0.5	0.8	0.7	2.2	0.8
CAMK2D	0.9	0.6	0.7	1.6	0.6
CAMK4	1.5	0.9	0.7	1.1	0.7
CDC42BPA	-0.5	-0.1	0.2	-1.5	-1.1

CLK3	0.7	0.7	0.8	1.1	0.4
DYRK2	1.1	2.2	0.7	7.0	2.0
CDKL1	-0.4	0.0	-0.4	0.2	0.2
CHEK2	1.0	1.9	0.2	0.7	-0.2
CLK1	3.1	2.7	2.2	4.7	1.0
DAPK1	-0.9	-0.2	-0.3	0.2	-0.3
CSNK2A2	1.1	2.2	0.4	2.9	0.4
DAPK3	0.6	0.7	0.7	1.9	0.9
FES	-0.5	-0.4	-0.2	-0.3	-1.0
GSG2	0.7	3.3	1.1	4.0	1.6
TTK	1.0	2.1	0.7	3.3	0.1
EPHA2	0.0	0.4	0.0	1.9	0.6
MAPK1	-0.2	-0.1	-0.4	0.1	0.1
MERTK	1.0	0.7	0.5	2.4	0.4
MST3	-1.7	3.0	0.8	-2.7	2.3
DCAMKL1	3.0	1.9	1.6	1.9	2.4
DMPK1	1.4	1.5	1.3	2.8	1.1
EPHA5	0.5	0.9	0.9	1.9	1.0
EPHA7	2.6	1.5	1.5	2.3	1.1
EPHB3	-0.7	-1.0	-2.3	1.2	-0.7
FGFR1	-0.2	-0.2	-0.2	0.0	0.1
FGFR2	0.6	1.1	0.6	1.4	2.2
FGFR3	0.8	1.5	1.0	1.8	1.9
GAK	0.4	1.2	-0.3	2.5	0.8
GPRK5	0.0	1.5	0.0	-0.4	2.3
GSK3B	1.1	2.7	0.9	5.0	0.6
MAP2K1	0.1	0.6	-0.1	0.1	0.3
MAP2K4	1.1	1.3	0.5	2.5	2.3
MAP2K6	0.4	0.4	0.5	1.6	0.9
MAP3K5	0.6	1.3	0.6	1.3	1.1
MAPK10	1.9	2.8	1.0	3.5	1.5
MAPK13	1.8	2.4	1.0	10.6	2.1
MAPK14	0.6	-0.1	0.3	1.0	1.1
MAPK8	2.0	2.2	1.3	4.4	2.5
MAPK9	0.3	0.8	1.3	2.5	2.1
MARK3	2.9	3.5	2.4	5.7	3.6
MARK4	1.7	1.1	0.9	5.5	2.6
MELK	1.6	3.1	2.2	5.8	6.1
PIM3	1.6	1.9	0.4	4.4	1.5
FLT1	0.3	0.2	1.7	0.8	2.3
NEK1	-1.1	-0.3	-0.3	-1.1	-0.4
NEK2	1.7	5.0	2.6	4.9	7.5
NEK7	0.8	0.6	0.7	0.7	0.1
OSR1	0.2	1.4	0.0	1.0	1.5
PAK1	0.7	0.3	0.8	1.8	0.3
MST4	0.0	1.2	-0.2	-0.7	-1.4
PAK4	1.4	1.1	1.1	2.9	1.3

PHKG2	0.8	0.5	0.6	1.5	0.9
PIM1	1.3	1.0	0.7	3.5	0.9
RPS6KA1	0.5	0.8	0.0	0.4	0.2
STK10	1.5	0.8	0.4	0.9	0.0
AKT3	0.1	0.2	0.1	-0.3	-0.3
BMP2K	3.8	4.3	2.6	10.1	2.2
CAMK1D	1.4	1.3	1.0	2.2	1.4
CAMK1G	2.5	2.2	2.1	3.4	2.7
PCTK1	-1.2	-1.0	-2.3	-0.1	0.4
PKMYT1	-0.4	-0.2	-0.5	0.5	-0.5
PLK4	1.5	1.7	-0.3	3.6	1.3
RPS6KA5	3.3	4.1	1.8	8.0	1.6
SLK	1.2	1.9	0.2	0.3	0.2
SRC	-0.2	-0.2	-0.5	0.1	1.8
SRPK1	0.8	1.4	-0.8	0.7	-0.8
STK3	0.7	3.0	0.0	3.4	1.4
STK4	0.3	0.7	0.0	2.7	1.3
STK6	0.4	1.1	0.7	2.9	2.4
STK17A	2.0	2.9	0.6	2.6	0.1
STK17B	0.0	2.7	0.3	3.5	1.6
STK38L	2.1	1.9	0.1	3.3	0.0
STK39	1.2	0.7	0.3	0.0	0.0
BRD4	0.7	1.2	0.4	1.5	0.3
BRPF1	0.4	0.1	0.3	1.1	0.7
TAF1	-6.2	-0.3	-4.5	0.1	0.3
TIF1	4.0	3.0	2.5	3.9	2.5
ULK1	0.6	2.3	0.8	2.6	0.4
ULK3	4.1	6.4	2.2	7.6	1.3
VRK1	0.5	0.2	0.3	0.6	0.5
WNK1	-0.1	-0.2	0.3	0.1	0.8
CSNK1E	0.7	1.8	-0.2	4.5	1.0
EPHA4	0.7	0.9	0.2	1.7	0.7
FECHHS	-2.0	0.5	-1.7	0.9	0.3
HIPK2HS	0.6	0.4	-0.3	0.4	0.6
MAP2K7	1.6	2.6	0.6	-2.6	2.1
EPHB1	-1.2	0.2	-0.5	2.6	-0.4
AURKB	-1.1	0.4	-1.7	1.5	0.9
DYRK1A	0.1	0.6	0.2	2.9	0.5
MAPK15	0.0	1.1	0.0	2.5	0.1
MAPKAPK2	0.2	0.6	-0.1	-0.3	0.0
MSSK1	0.0	0.0	-0.2	0.0	-0.2
NQO2	-0.7	-0.7	-1.0	-0.4	-0.5
TLK1	1.1	1.2	0.8	1.3	0.8

Table S 3. Target occupancy [%] of **11i** measured in a NanoBRET® assay against 192 kinases.

kinase	occupancy [%]	kinase	occupancy [%]	kinase	occupancy [%]
LRRK2	-6.8	PRKAA1	16,6	CDKL3	-3.3
MAPK6	-2.0	PRKAA2	8.1	CDKL5	-14.8
IRAK3	10.3	RPS6KA1	5.7	JNK3	7.8
TEK	35.7	RPS6KA2	15.8	MAPK11	2.4
TNK1	9.1	RPS6KA4	-10.5	MAPK14	-4.2
GAK	20.1	RPS6KA6	2.9	NLK	1.1
MAPK4	15.2	SIK1	12.6	NEK11	-5.2
AAK1	29.3	CLK4	0.9	NEK1	24.0
AURKA	1.6	MAPK8	16.3	NEK2	-
AURKC	21.3	MAPK9	8.6	NEK4	-1.0
AURKB	12.9	IKBKE	26.4	PAK4	-
NUAK1	12.7	LATS1	4.8	MAP4K3	-
LATS2	10.6	PRKX	10.5	STK11	19.0
RPS6KA3	28.8	CSNK2A2	10.9	SLK	5.6
SNF1LK2	12.4	HIPK4	23.6	DAPK2	23.1
MYLK2	11.2	STK10	-9.3	MAP3K2	19.9
AXL	2.22	FGFR4	2.00	PLK2	1.1
FGFR3	9.3	MAP4K1	2.2	PLK3	-
FLT3	11.6	MERTK	-5.7	PLK4	-
IGF1R	7.5	MET	-3.2	STK35	-45.4
INSR	-2.8	RON	-4.2	STK17B	25.5
LIMK2	20.3	TYRO3	17.2	TLK2	14.6
TEC	16.8	LCK	17.5	BRSK2	-4.7
TIE1	3.3	LIMK1	-32.7	MARK2	-10.0
CLK1	10.9	EPHA1	3.9	MELK	10.1
SBK3	-1.6	EPHA4	-14.7	CSNK1A1L	-
NEK9	6.6	EPHA6	12.9	CSNK1D	24.6
NEK3	23.3	EPHA7	2.2	CSNK1G2	-36.0
NIM1K	16.6	EPHB1	-5.8	SIK3 FL	4.1
STK36	8.3	EPHB4	-33.6	SNRK	14.8
ULK2	16.3	FYN	14.5	CAMK1	-2.5
ULK3	14.7	ABL2	7.8	CAMK2A	-1.7
BRSK1	9.1	BMX	92.3	CAMK2D	13.3
MAP3K10	6.3	BTK	40.6	CHEK2	-
MAP3K9	7.9	FER	17.6	DCLK3	18.5
MYLK3	16.8	FES	-20.4	MKNK2	7.5
PHKG1	13.3	JAK3	-5.1	PHKG2	16.2
STK33	7.4	SRMS	7.8	MAP3K3	3.9
STK4	8.5	TXK	73.8	RIOK2	-2.6
TLK1	14.4	CLK2	12.8	MAP4K5	12.1
FGFR1	-13.0	DYRK1A	5.1	MAST3	-
FGFR2	25.8	DYRK1B	11.7	MAST4	-1.2
MUSK	15.6	ERN1	5.3	STK32B	14.9

NTRK1	35.8	ERN2	-7.1	STK3	-12.2
RET	-4.9	HIPK2	-2.5	STK38	0.8
NTRK2	16.4	HIPK3	1.7	STK38L	-14.0
TNK2(iso1)	-59.5	ICK	-0.7	PAK6	3.7
LTK	-12.6	CDK1	5.2	AKT2	-11.6
BRAF(V600E)	6.8	CDK2	2.2	PKMYT1	-9.7
IRAK4	11.7	CDK3	2.6	PRKACA	5.6
ITK	31.1	CDK4	3.4	PRKACB	8.9
JAK2 (V617F)	18.2	CDK5	-7.0	PRKCE	11.81
MAP3K11	RBC	CDK6	5.3	SGK1	9.5
PTK2	16.7	CDK7	9.5	WEE2	-11.2
PTK6	9.7	CDK9	5.5	RIPK1	3.3
PTK2B	1.9	CDK10	-14.9	RIPK2	12.9
BMP2K	-3.6	CDK14	8.5	TNNI3K	-13.9
NEK5	33.2	CDK15	-7.8	MLTK	-24.2
STK16	-3.00	CDK16	6.4	MAP3K12	1.9
TBK1	14.6	CDK17	0.5	MAP3K19	-
ULK1	26.2	CDK18	-1.2	MAP3K21	-5.8
MAP4K2	9.2	CDKL2	3.6	MAP3K4	25.5
WEE1	-79.9	CDK20	-12.2		
MYLK4	17.0	CDKL1	1.2		
MARK4	20.5	CSNK2A1	2.7		

Table S 4. Polar interaction frequency [%] along the MD simulation trajectory (5 x 1 μs per ligand/kinase combination) separated by residue, where (w) denotes water-mediated hydrogen bonds, (pi) pi-stacking interactions and (pc) pi-mediated charge interactions. Double numbers in the kinase interaction residues denote double binding from multiple ligand's atoms.

**COV - BTK**							**NONCOV - BTK**			
**Motif**	**Residues**	**Control**	**10**	**12c**	**11i**	**11m**	**Motif**	**Residues**	**11i**	**11m**
HINGE	Glu475	99		37(w)		42(w)	HINGE	Glu475		
HINGE	Met477	99	81/85	59/49	99/96	97/51	HINGE	Met477	97/73	93/76
GATEKEEP	Thr474		63	33(w)	66	32<br>36(w)	GATEKEEP	Thr474	37	44
CATALYTIC	Lys430	61(w)	48(w)		45(w)	34(pc)<br>32(w)	CATALYTIC	Lys430		
DFG	Asp539		51(w)		46(w)		DFG	Asp539		
DFG	Phe540	70(pi)					DFG	Phe540		
COVsite	Asn484	-	-	-	-	-	COVsite	Asn484	31	37
COVsite	Cys481	95	68	36	72	32	COVsite	Cys481		
**COV - BMX**							**NONCOV - BMX**			
Motif	**Residues**	**Control**	**10**	**12c**	**11i**	**11m**	**Motif**	**Residues**	**11i**	**11m**
HINGE	Ile492	83	99/90	95/90	99/97	99/94	HINGE	Ile492	99/97	99/93
HINGE	Glu490		34(w)				HINGE	Glu490		38(w)
GATEKEEP	Thr449		39(w)	52	67	51	GATEKEEP	Thr449	73	40/37(w)
CATALYTIC	Lys553		55	33	36(w)	35(pc)	CATALYTIC	Lys553		30(pc)
	Ser553				32			Ser553	35(w)	
COVsite	Cys496		76		45	41	COVsite	Cys496		

Table S 5. MM-GBSA derived ΔG binding free energy components is calculated from 5 μs (5 x 1 μs) MD simulations per ligand-protein systems. H-bond – hydrogen bond energy contribution, Lipo – lipophilic interaction energy contribution, and Bind Packing – π–π packing interaction energy contribution to ΔG binding free energy. L.E. – Ligand efficacy. Heavy atom count (HAC) is used for ligand efficacy evaluation. HAC is a measure of the number of non-hydrogen atoms in the ligand molecule.

		H-bond		Lipo		Bind Packing		L.E.	
Kinase	CPD	AVG	SD	AVG	SD	AVG	SD	AVG	SD
BTK	11m	-1.50	0.34	-20.05	1.40	-0.82	0.64	-15.26	1.29
BMX	11m	-1.67	0.36	-20.50	1.90	-1.12	0.76	-15.57	1.49
BTK	11i	-1.51	0.37	-20.77	1.57	-0.57	0.67	-15.96	1.42
BMX	11i	-2.03	0.40	-22.15	2.35	-1.06	0.74	-17.94	1.91

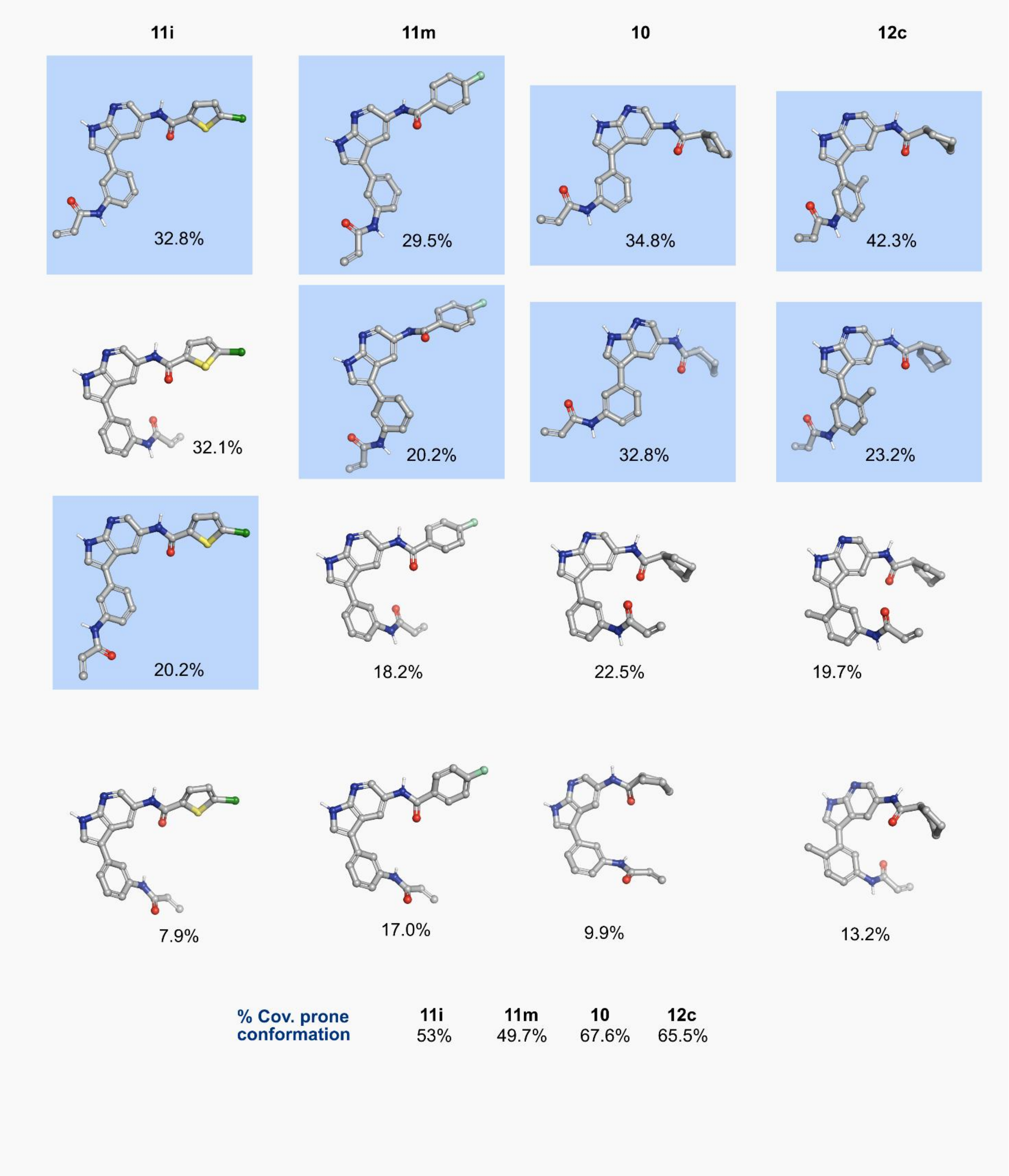

Figure S 1. QM calculated conformations and their Boltzmann population frequency generated based their energy in solution. The frequency of observed conformations prone to covalent bond (Cov. Prone conformations), meaning where the warhead is pointing towards the relevant cysteine (see Figure 7) are quantified below the structures.

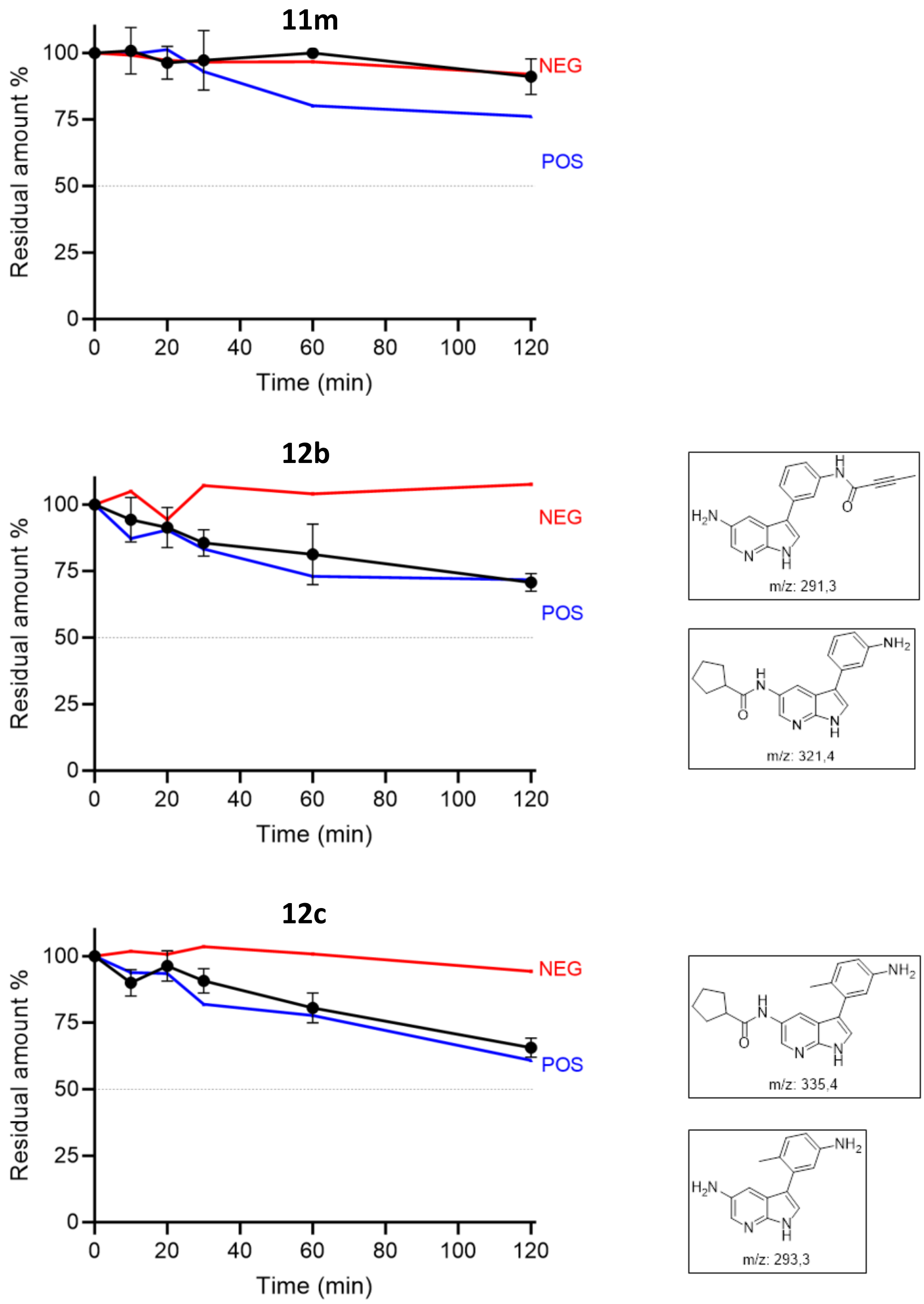

Figure S 2. Human liver microsomal stability of compounds **11m**, **12b** and **12c** and presumed structure of found metabolites.

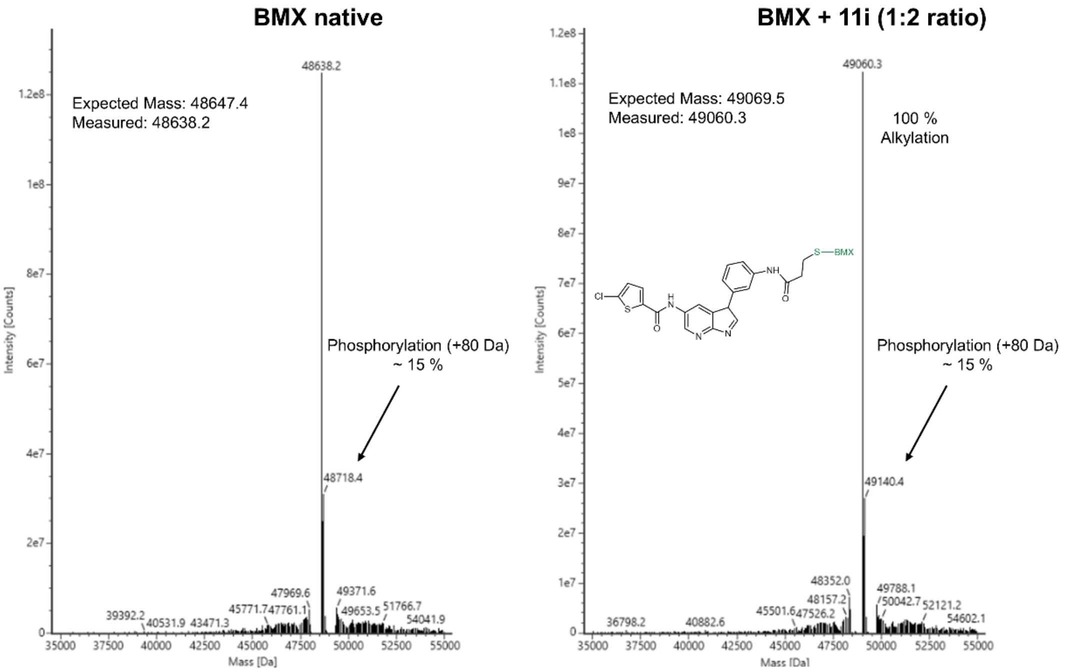

Figure S 3. Intact protein MS experiment of BMX with **11i**. 100% labelling was observed with 2-fold compound excess after 90°C min incubation time at 4 °C.

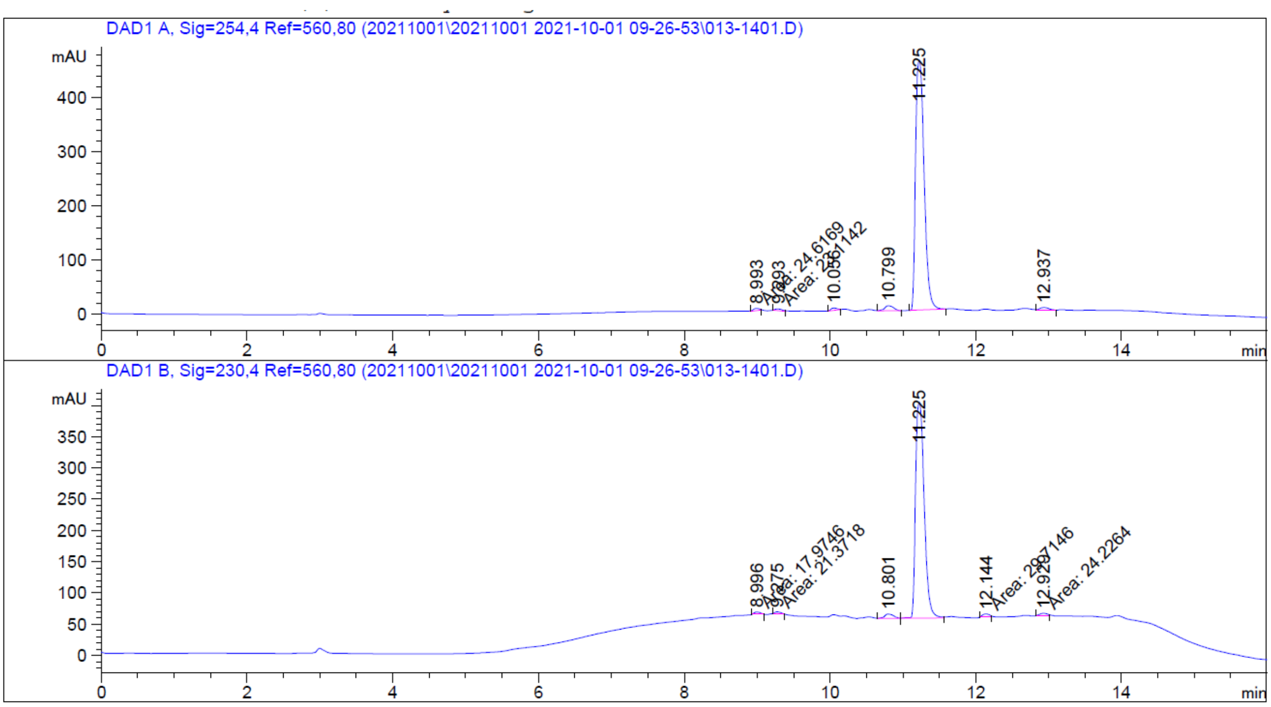

Figure S 4. HPLC spectrum traces of **11i**. t_{ret} = 11.23 min. (Method B). Purity: 95.1 % (254.4 nm), 95.0 % (230.4 nm).

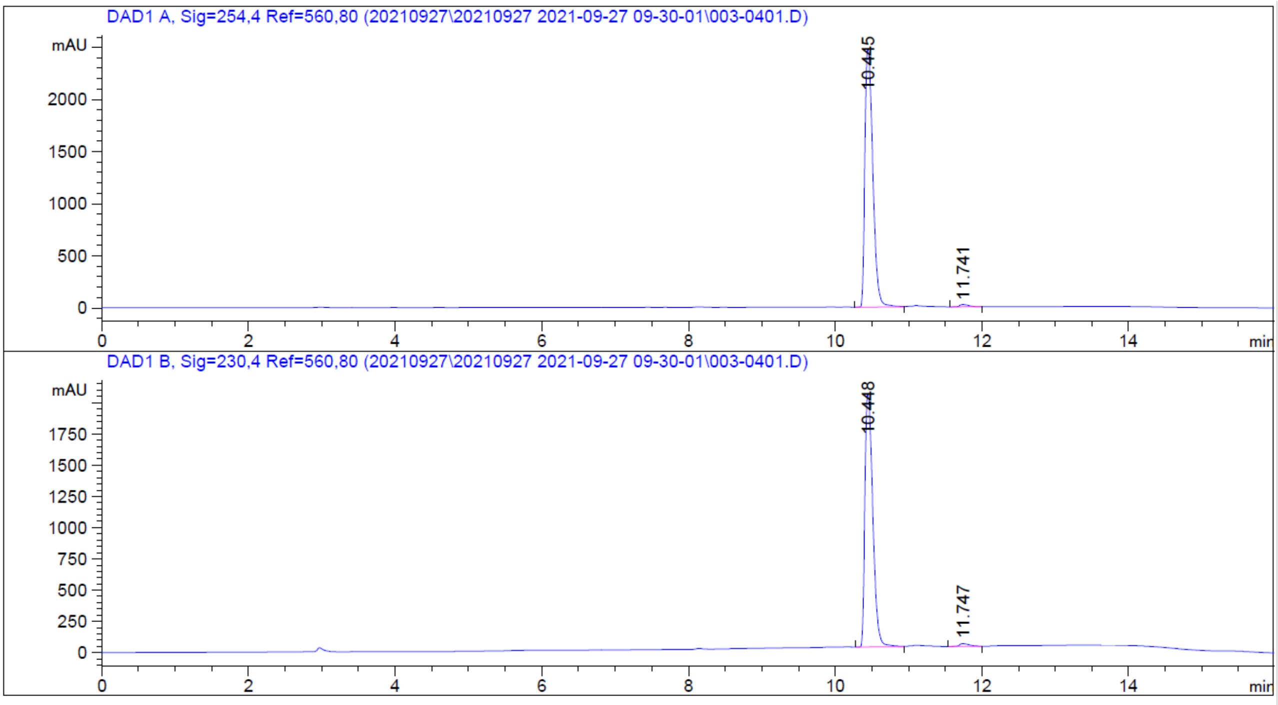

Figure S 5. HPLC spectrum traces of **11m**. t_{ret} = 10.45 min. (Method B). Purity: 99.0 % (254.4 nm), 98.8 % (230.4 nm).

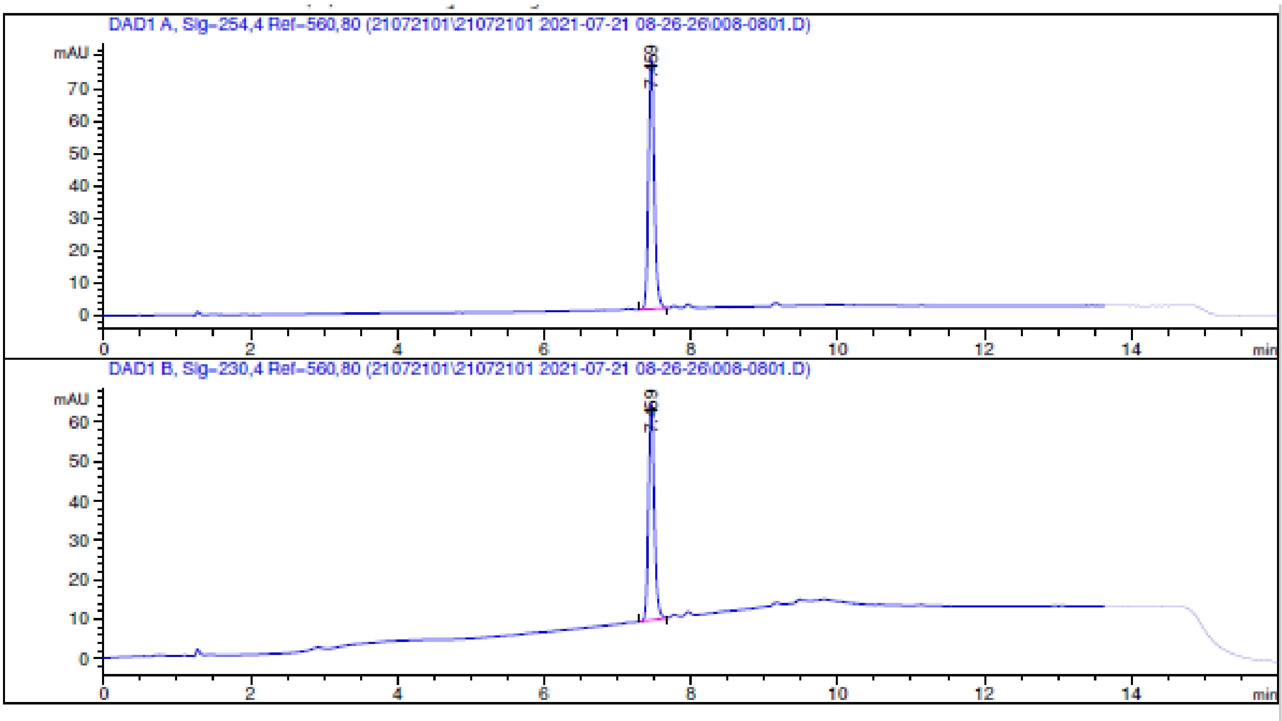

Figure S 6. HPLC spectrum traces of **12b**. t_{ret} = 7.46 min. (Method A). Purity: 100.0 % (254.4 nm), 100.0 % (230.4nm).

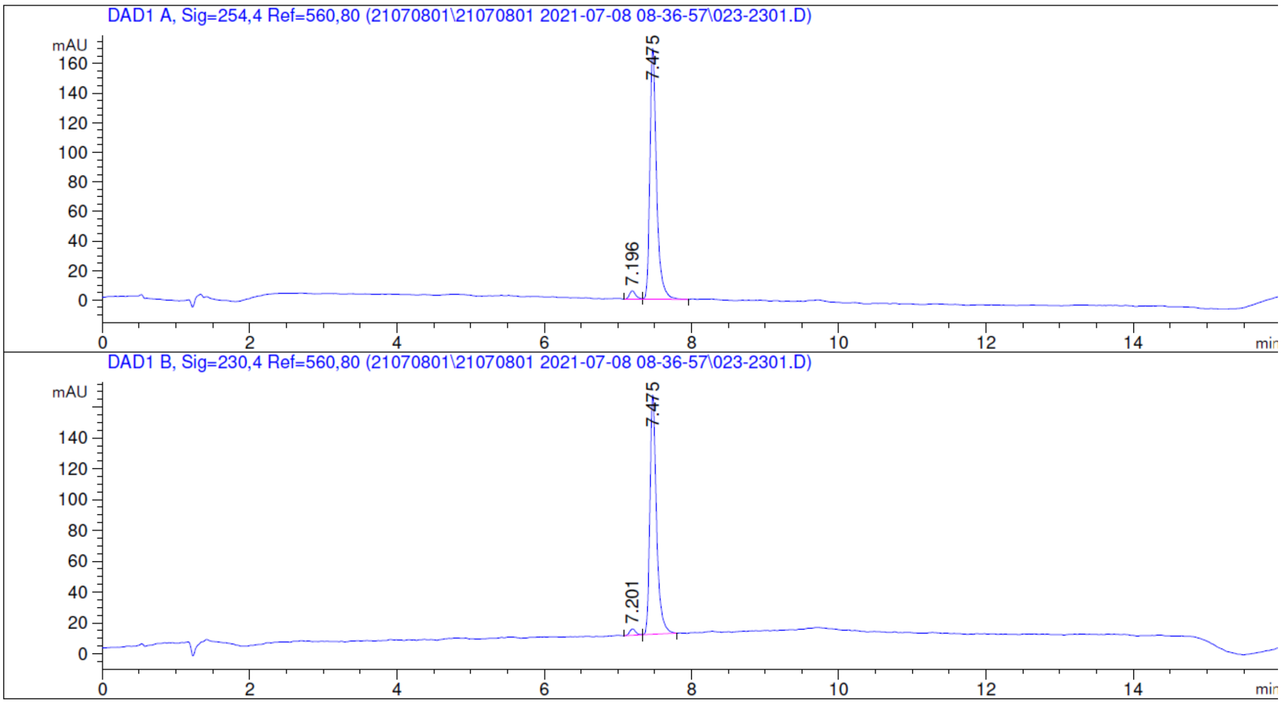

Figure S 7. HPLC spectrum traces of **12c**. t_{ret} = 7.48 min. (Method A). Purity: 97.0 % (254.4 nm), 97.6 % (230.4 nm).

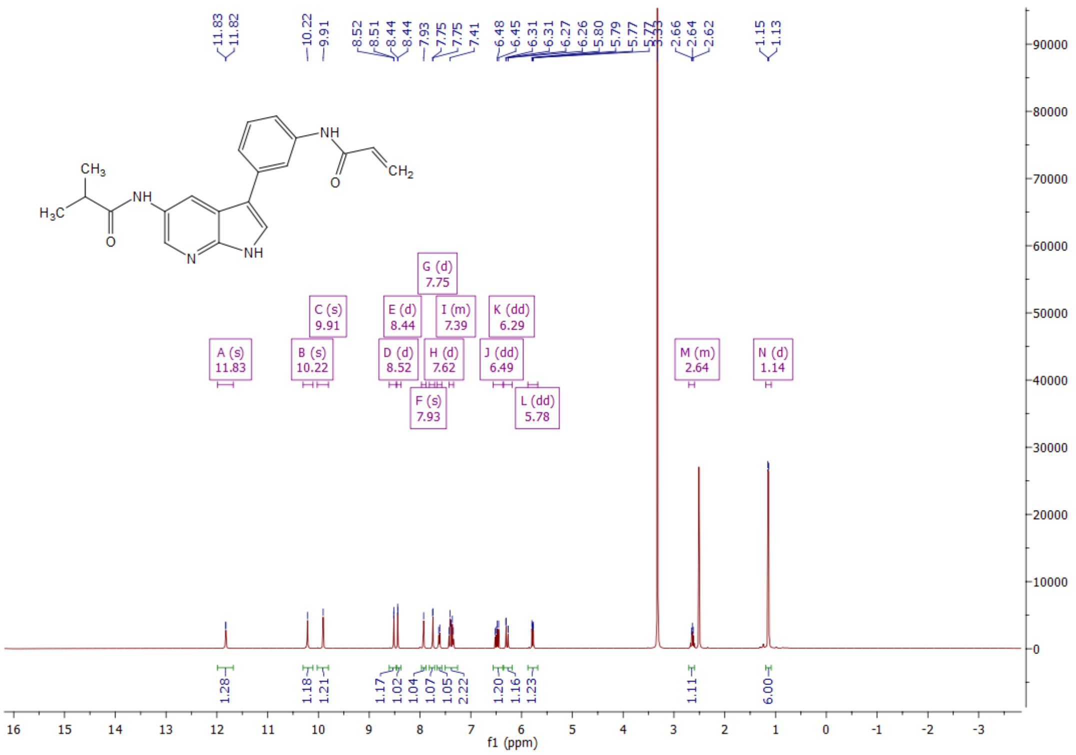

Figure S 8. ^{1}H-NMR spectrum of **11a** in DMSO- d_6.

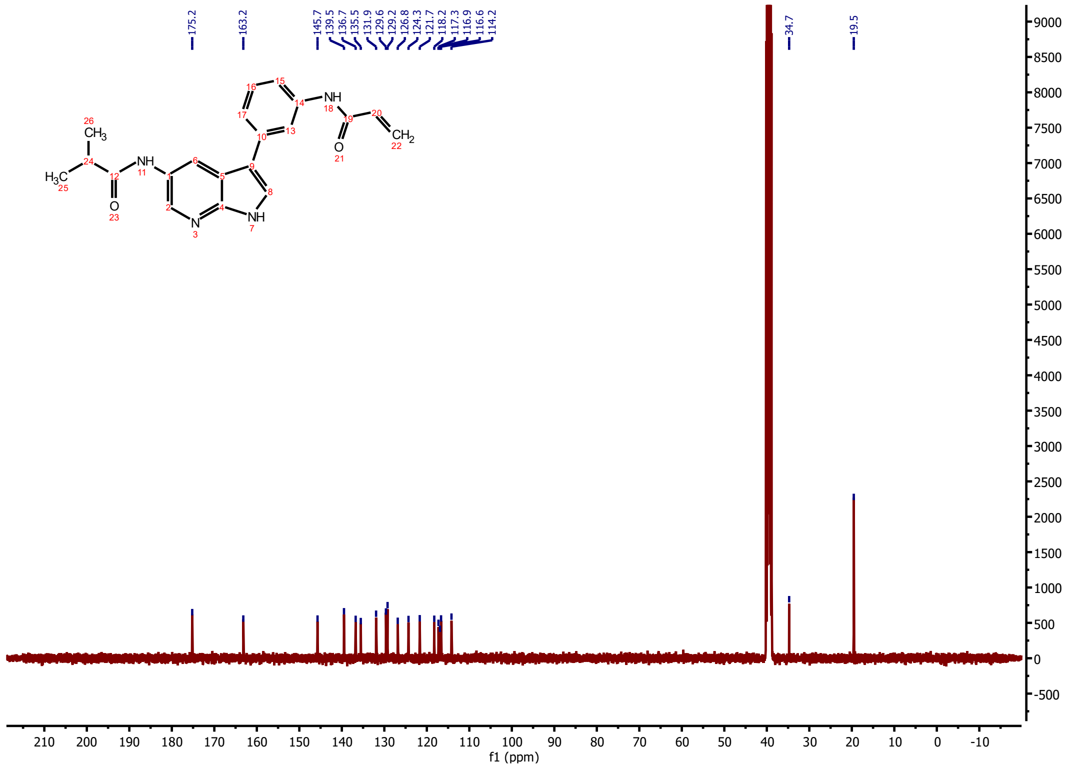

Figure S 9. ^{13}C-NMR spectrum of **11a** in DMSO- d_6.

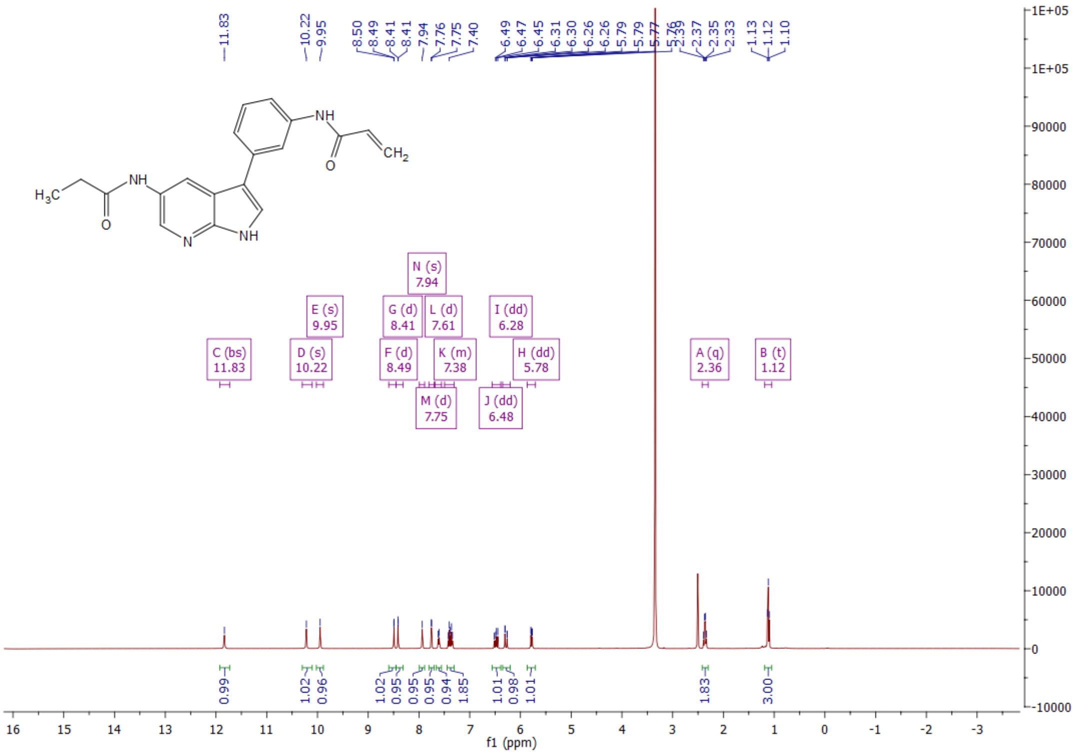

Figure S 10. ^{1}H-NMR spectrum of **11b** in DMSO- d_6.

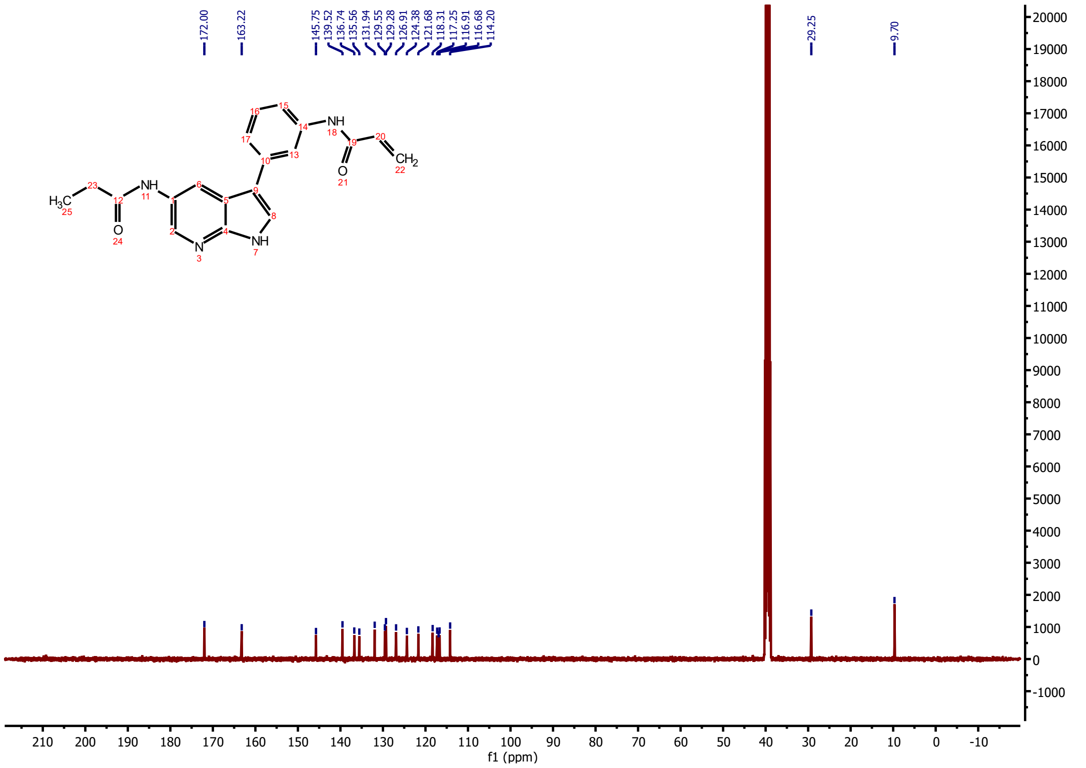

Figure S 11. ^{13}C-NMR spectrum of **11b** in DMSO- d_6.

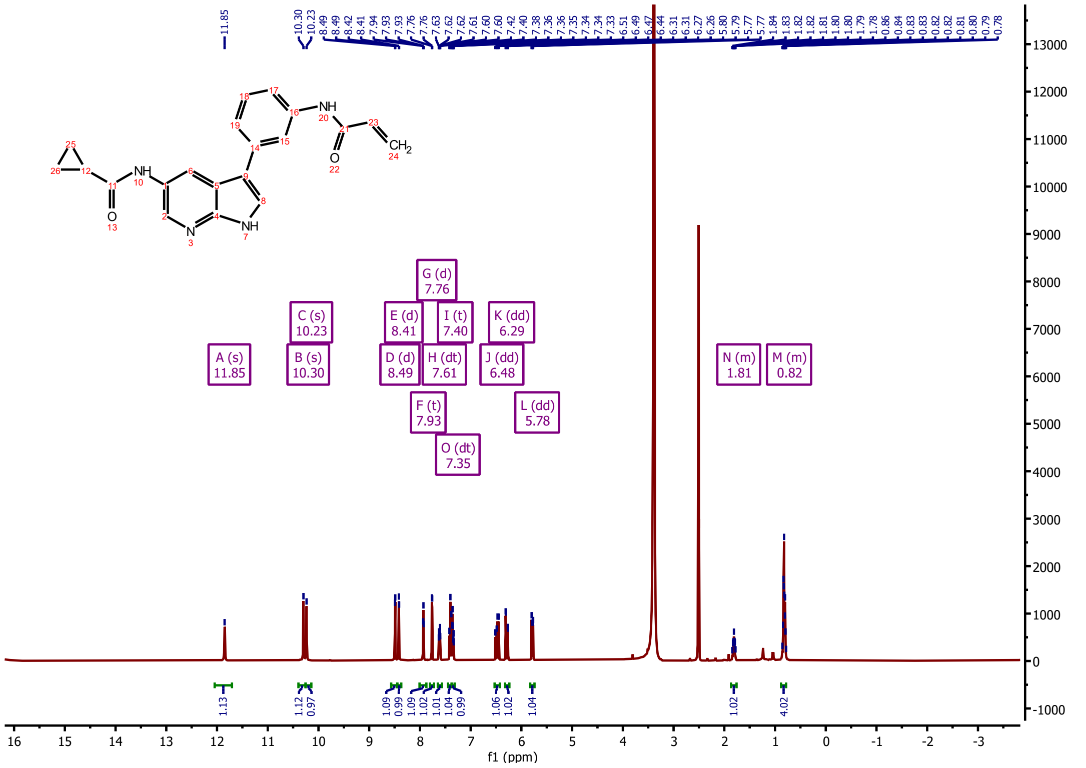

Figure S 12. ^{1}H-NMR spectrum of **11c** in DMSO- d_6.

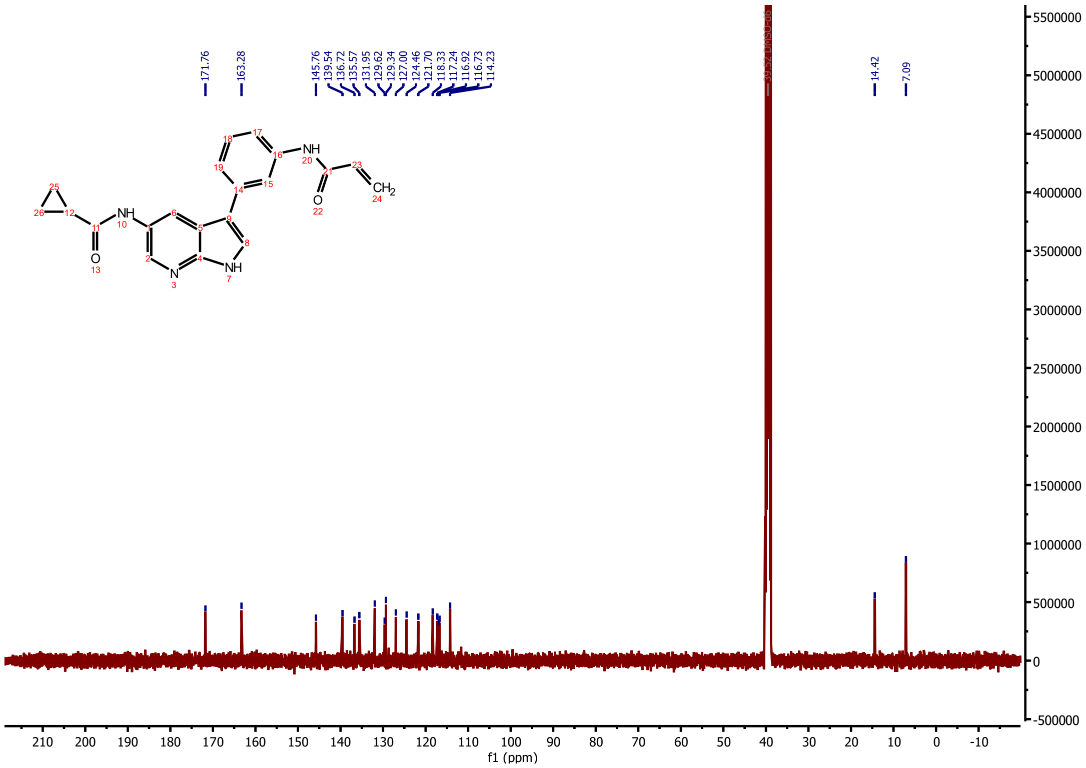

Figure S 13. ^{13}C-NMR spectrum of **11c** in DMSO- d_6.

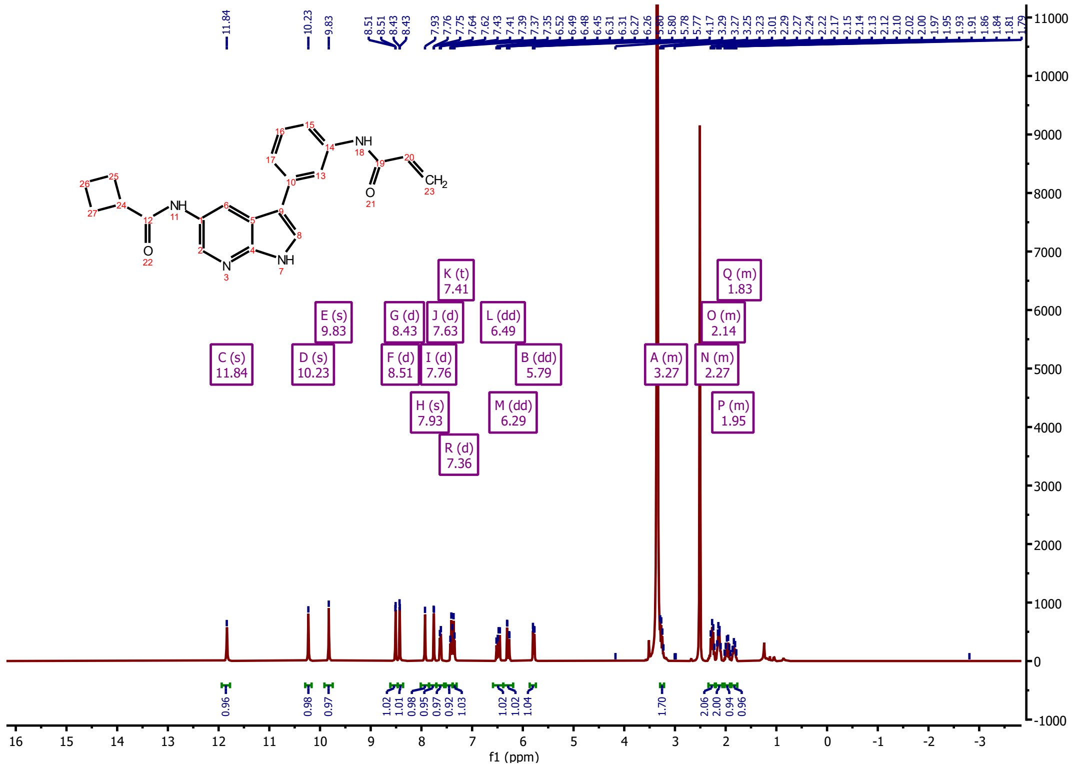

Figure S 14. ^{1}H-NMR spectrum of **11d** in DMSO- d_6.

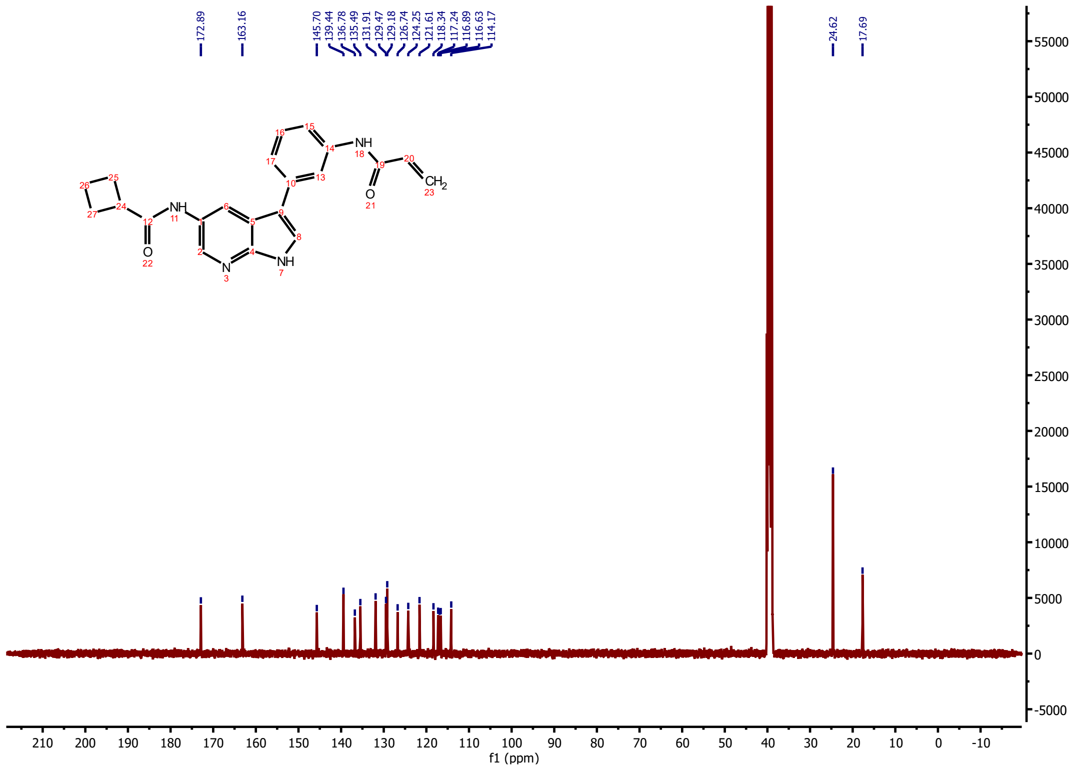

Figure S 15. ^{13}C-NMR spectrum of **11d** in DMSO- d_6.

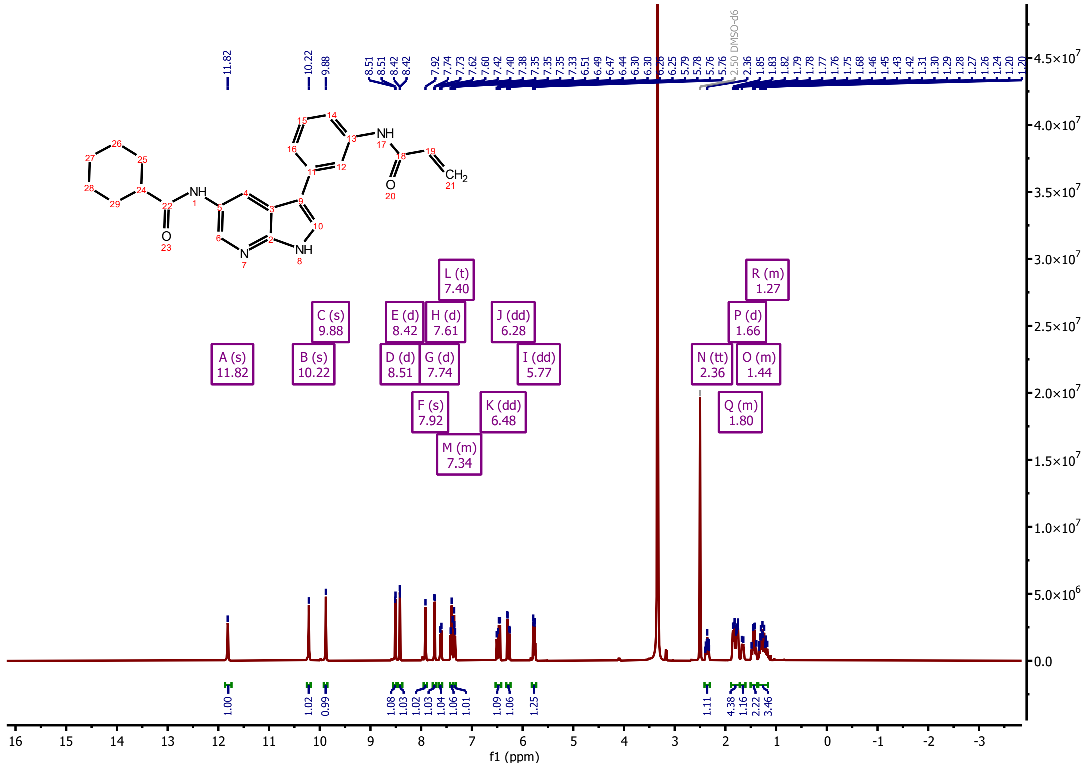

Figure S 16. ^{1}H-NMR spectrum of **11e** in DMSO- d_6.

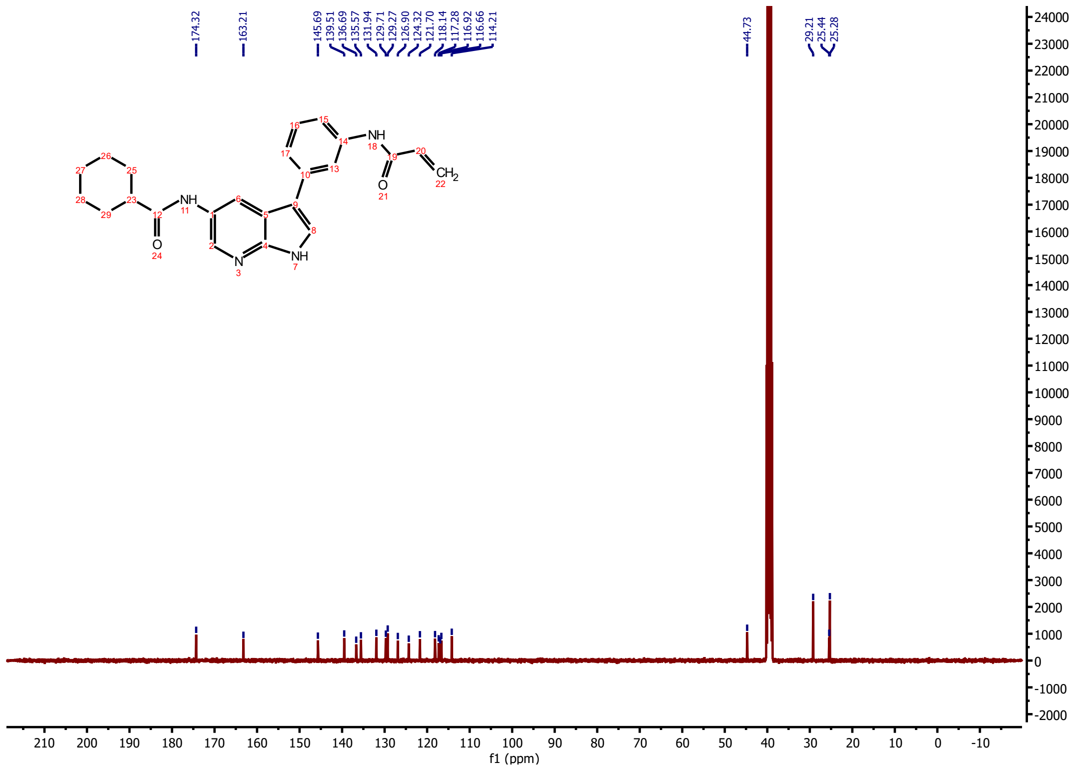

Figure S 17. ^{13}C-NMR spectrum of **11e** in DMSO- d_6.

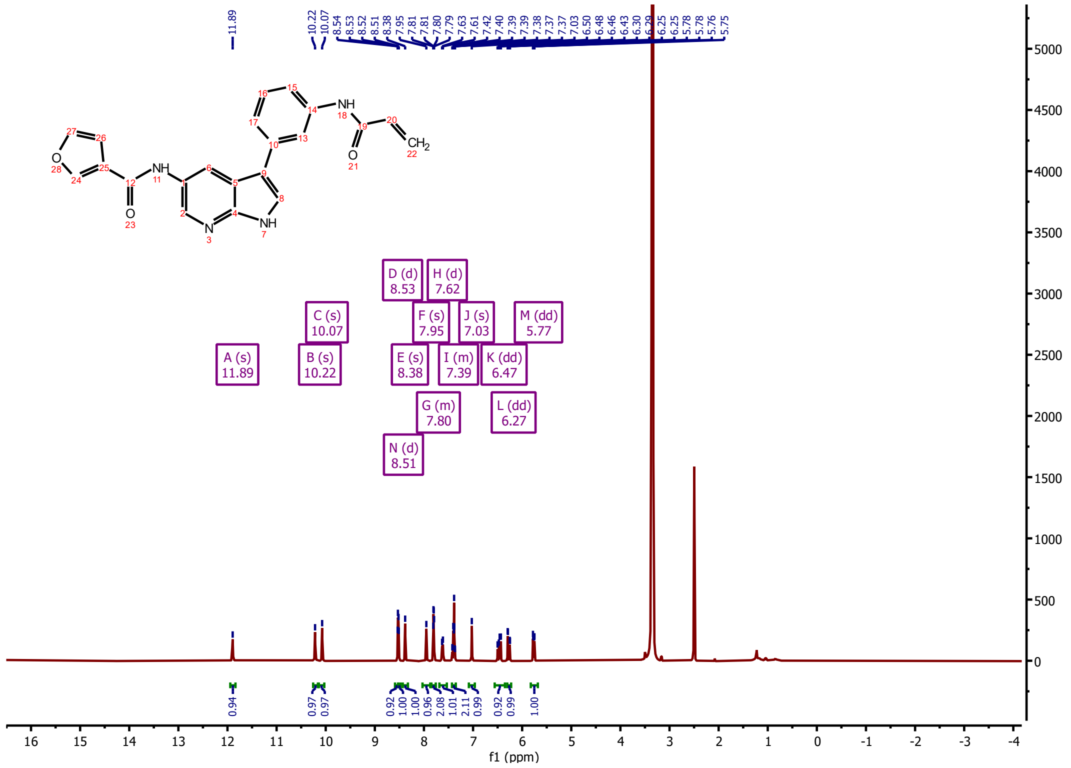

Figure S 18. ^{1}H-NMR spectrum of **11f** in DMSO- d_6.

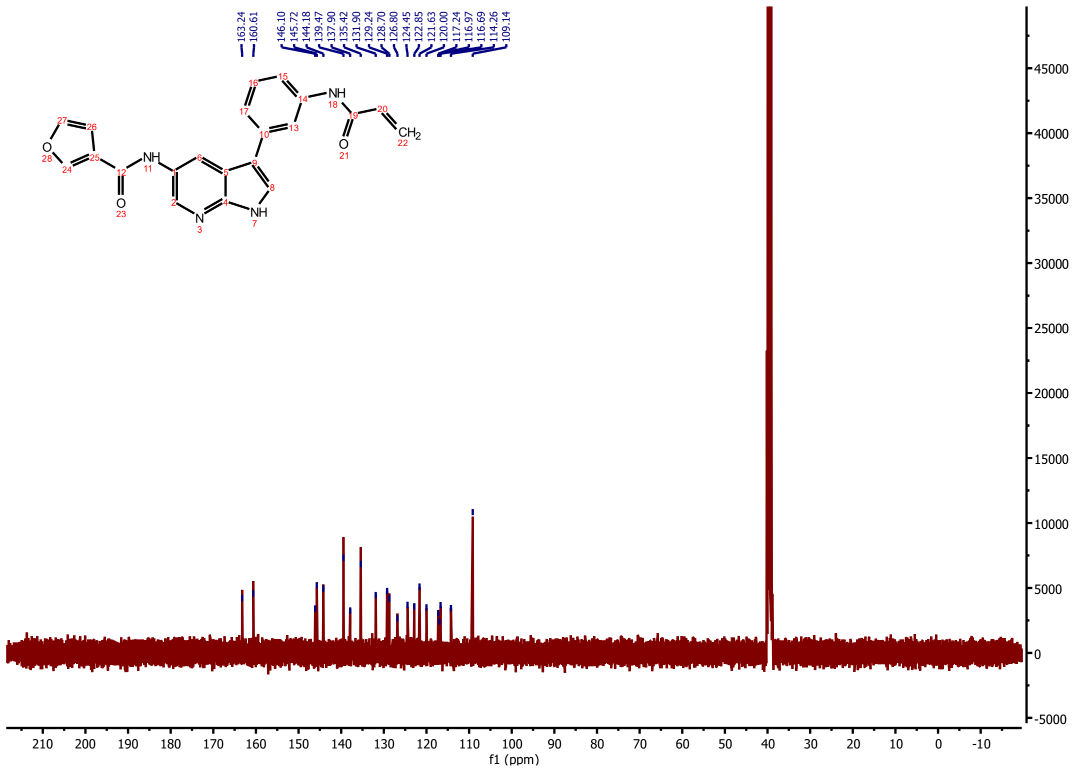

Figure S 19. ^{13}C-NMR spectrum of **11f** in DMSO- d_6.

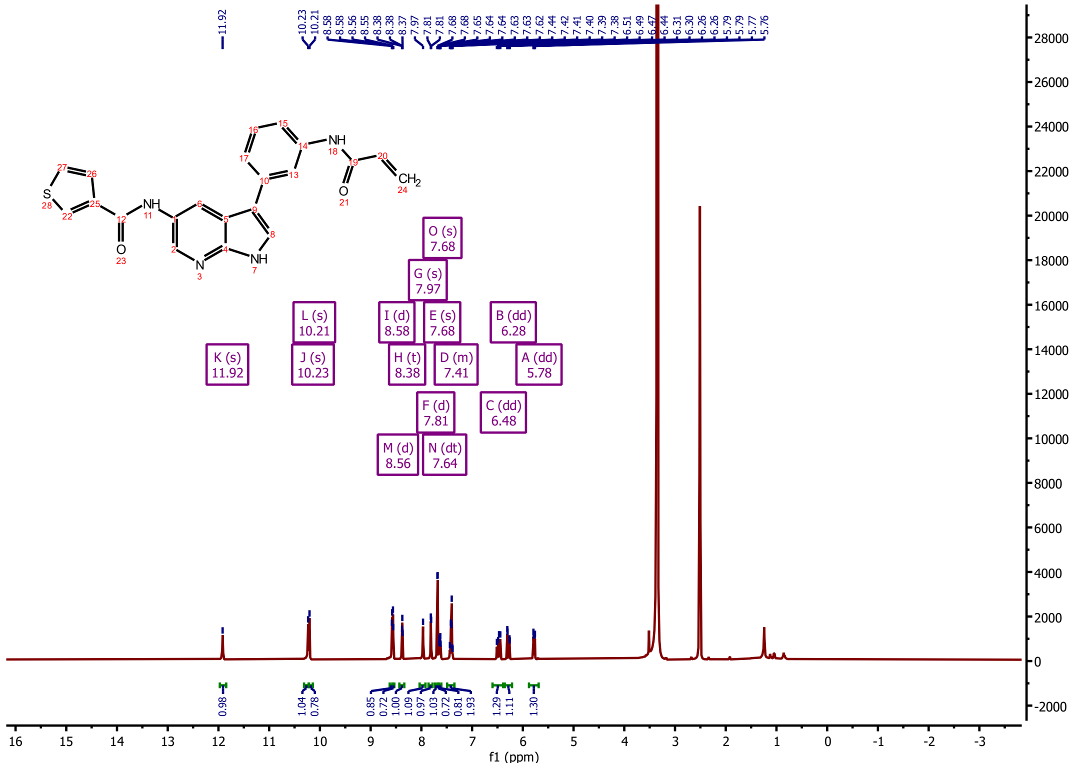

Figure S 20. ^{1}H-NMR spectrum of **11g** in DMSO- d_6.

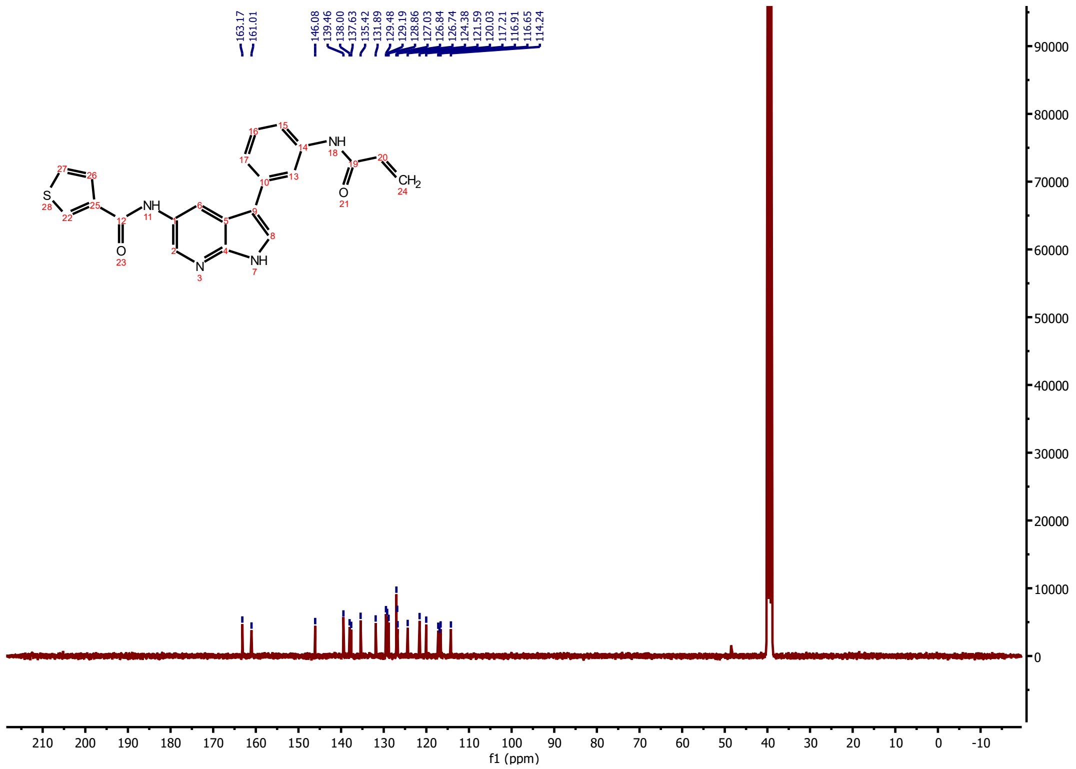

Figure S 21. ^{13}C-NMR spectrum of **11g** in DMSO- d_6.

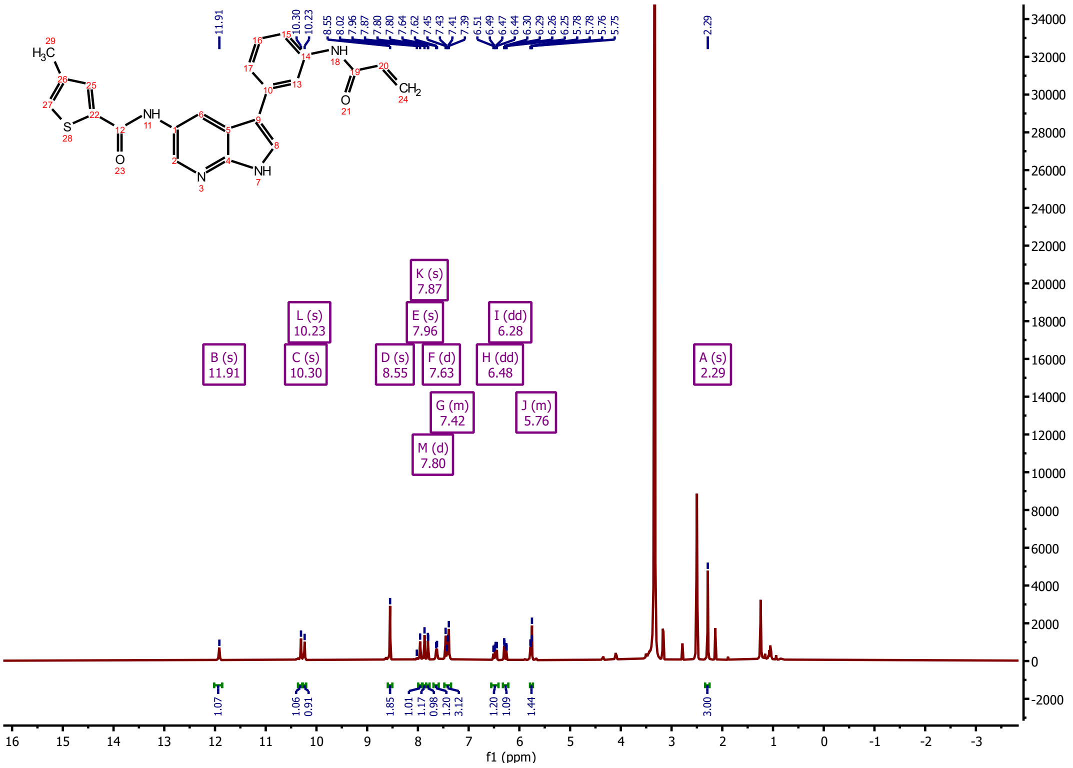

Figure S 22. ^{1}H-NMR spectrum of **11h** in DMSO- d_6.

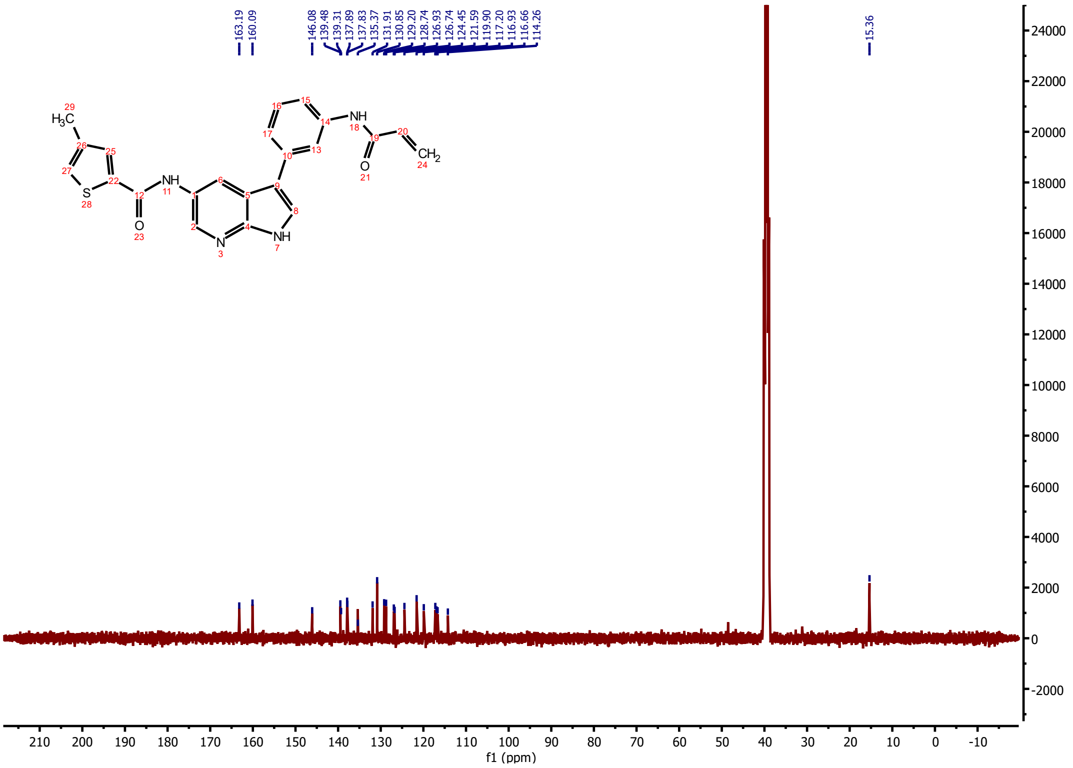

Figure S 23. ^{13}C-NMR spectrum of **11h** in DMSO- d_6.

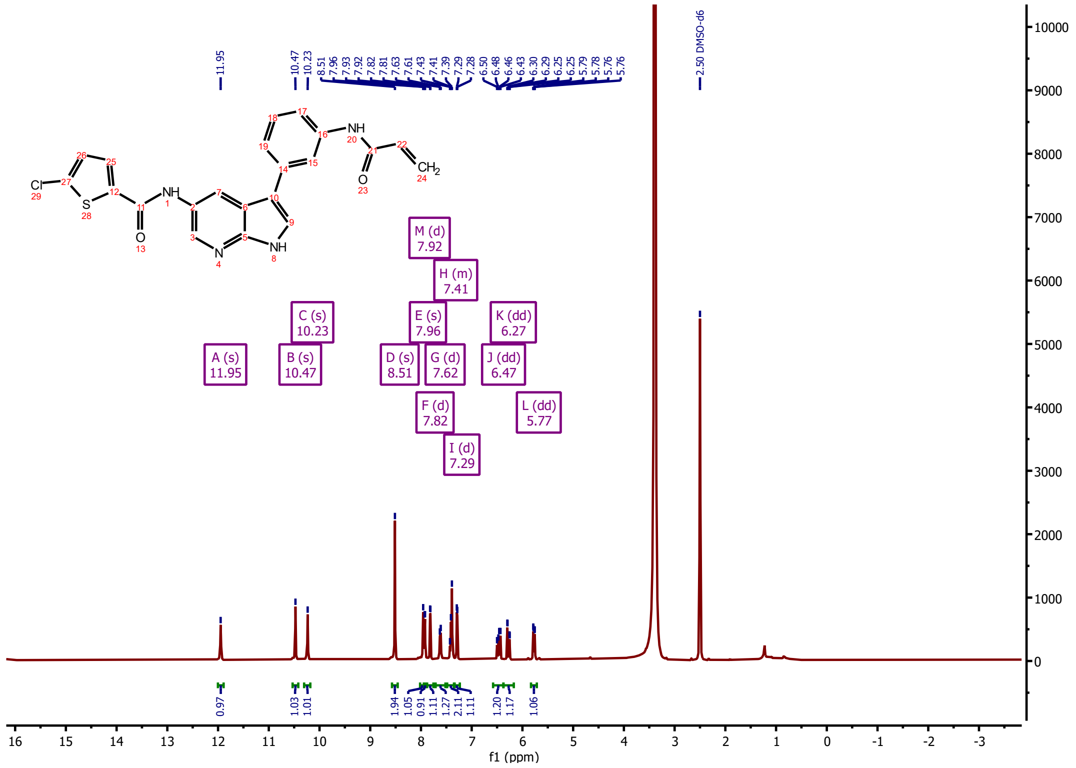

Figure S 24. ^{1}H-NMR spectrum of **11i** in DMSO- d_6.

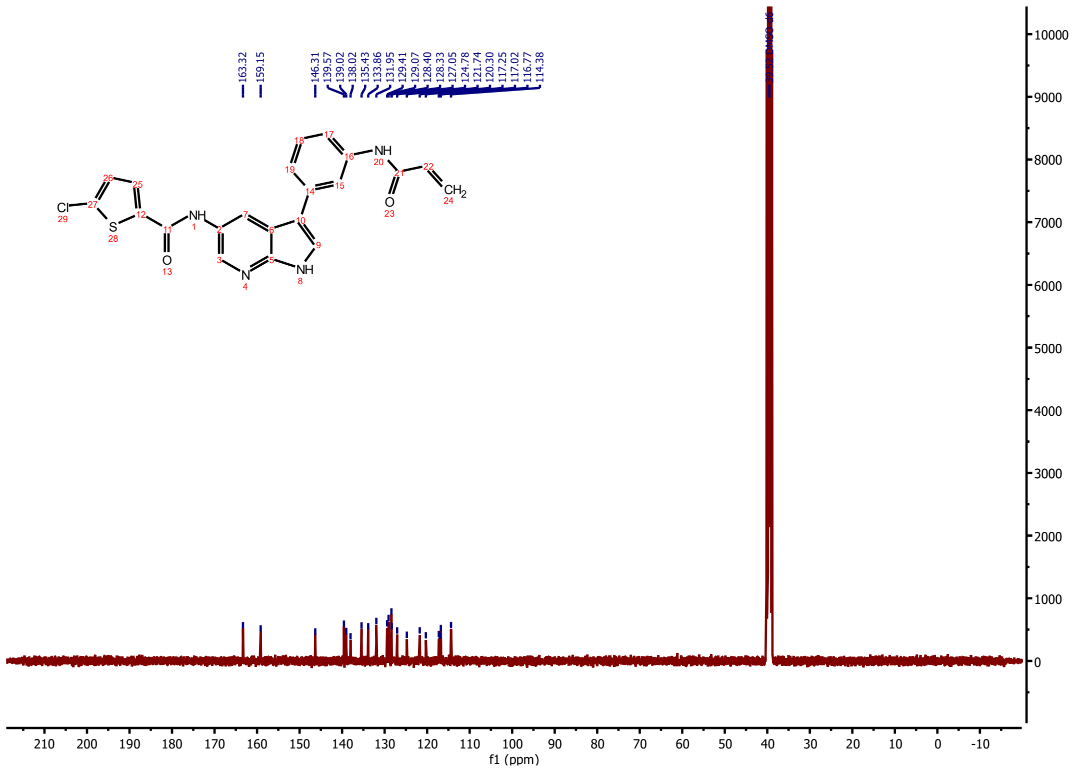

Figure S 25. ^{13}C-NMR spectrum of **11i** in DMSO- d_6.

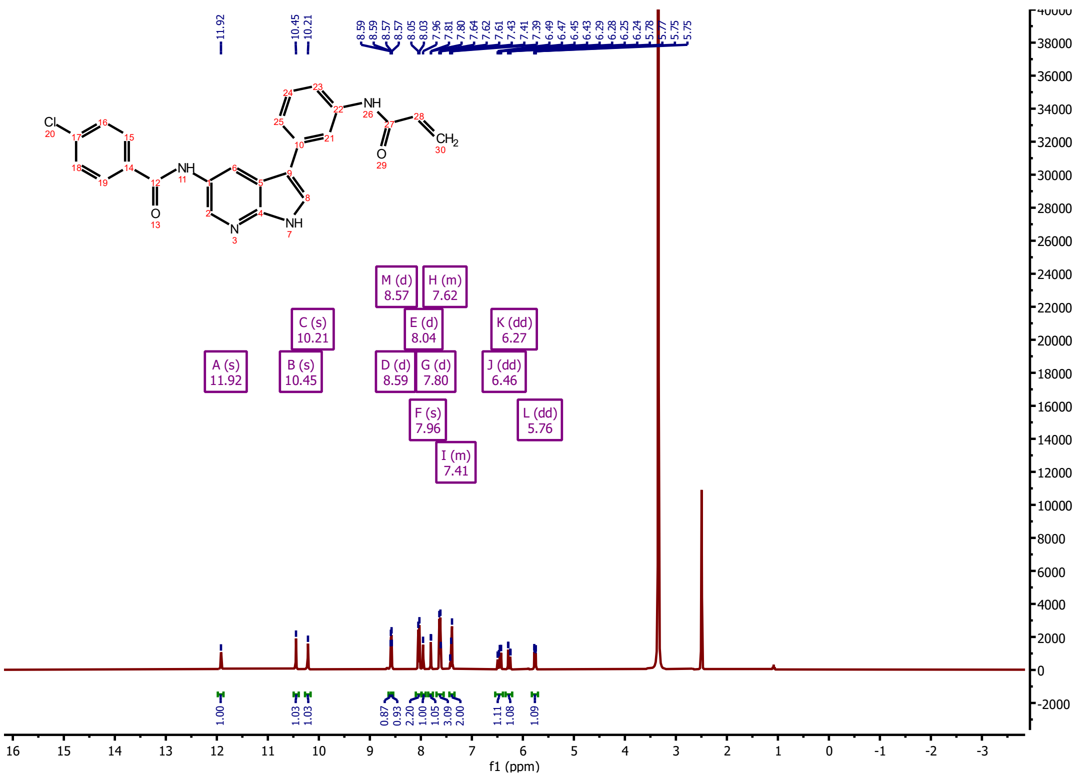

Figure S 26. ^{1}H-NMR spectrum of **11j** in DMSO- d_6.

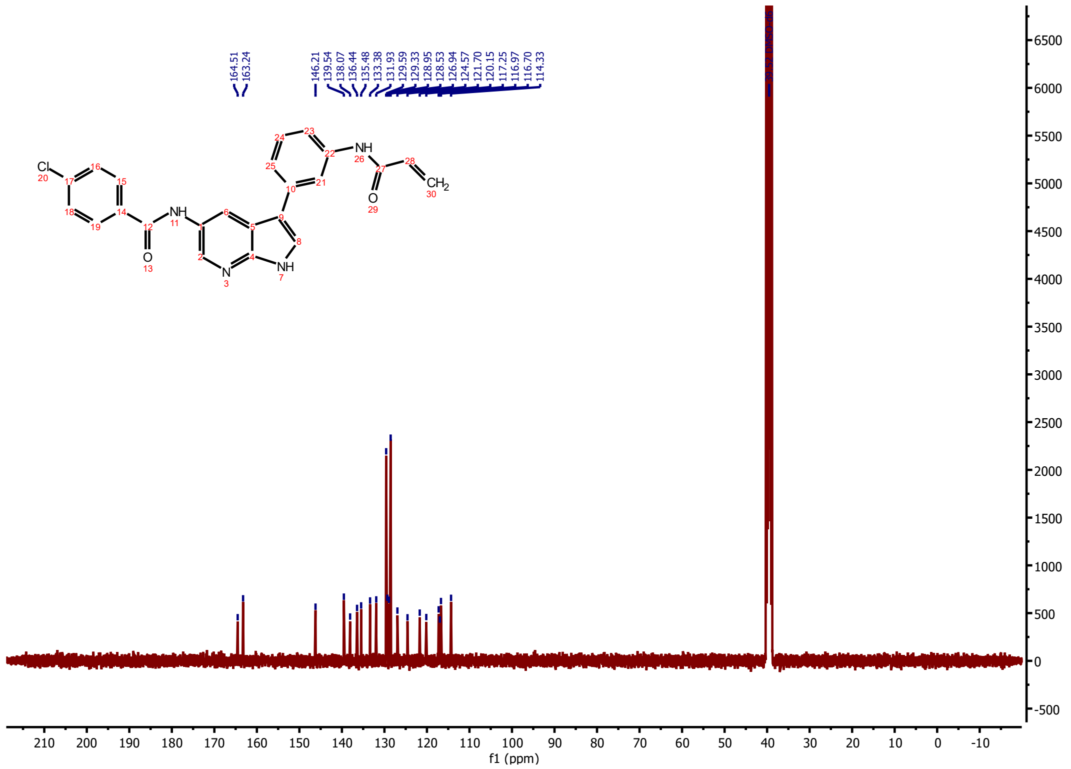

Figure S 27. ^{13}C-NMR spectrum of **11j** in DMSO- d_6.

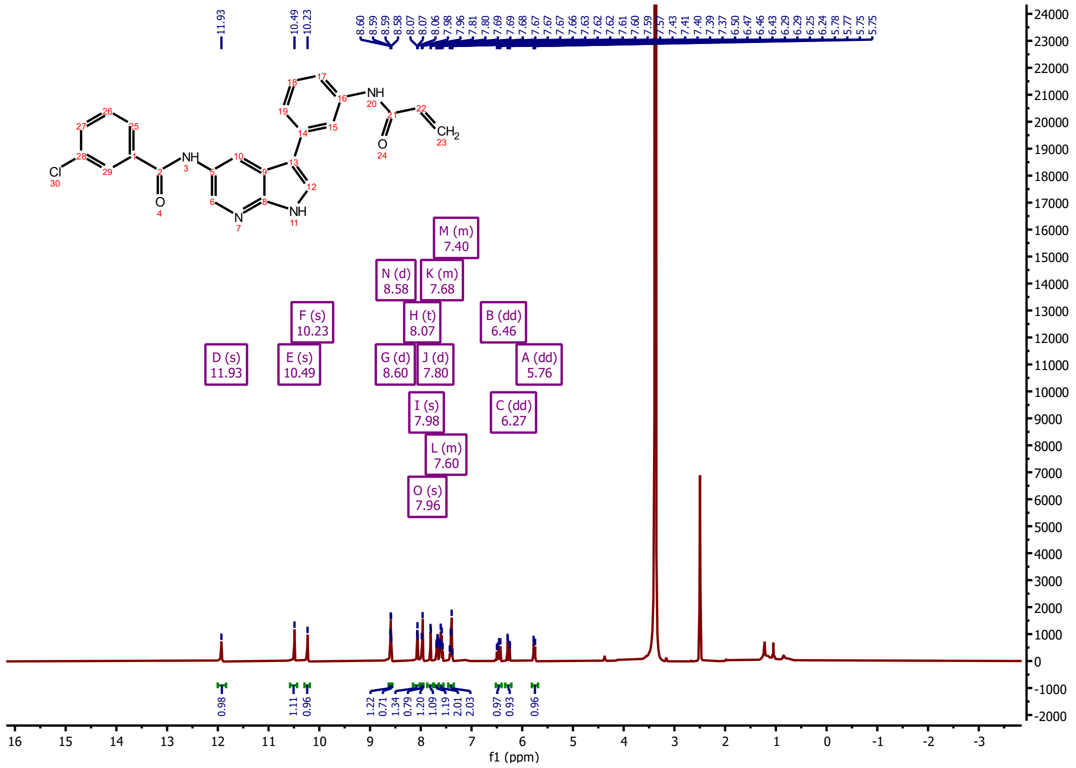

Figure S 28. ^{1}H-NMR spectrum of **11k** in DMSO- d_6.

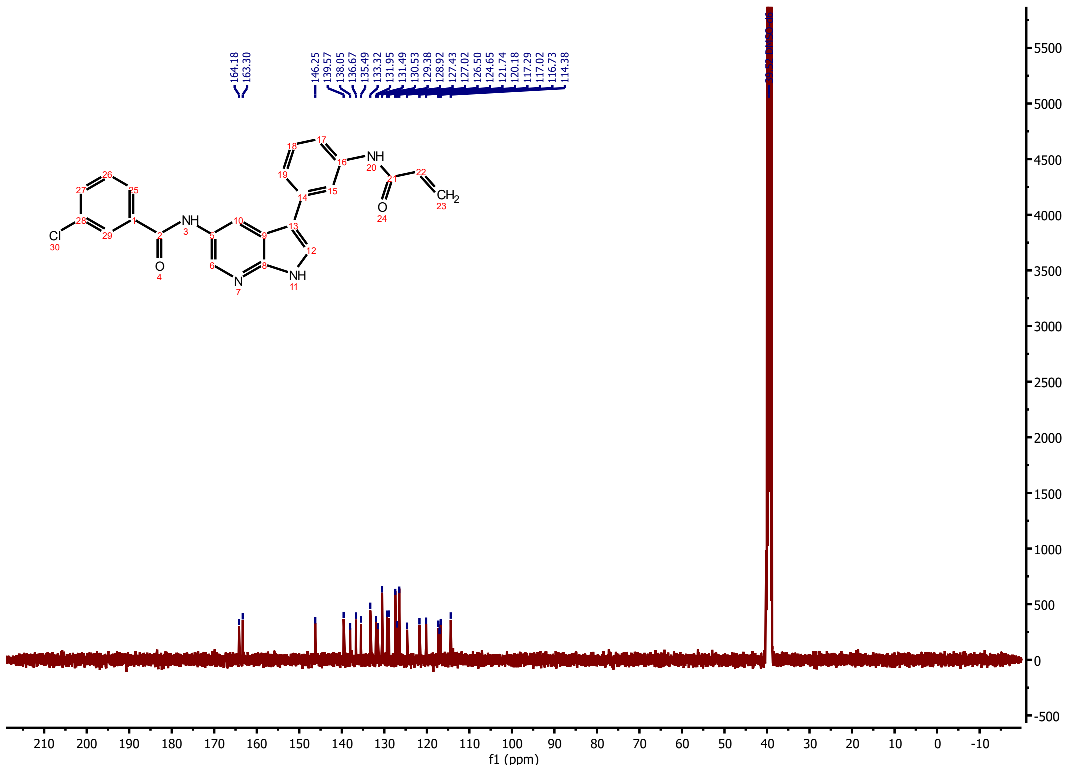

Figure S 29. ^{13}C-NMR spectrum of **11k** in DMSO- d_6.

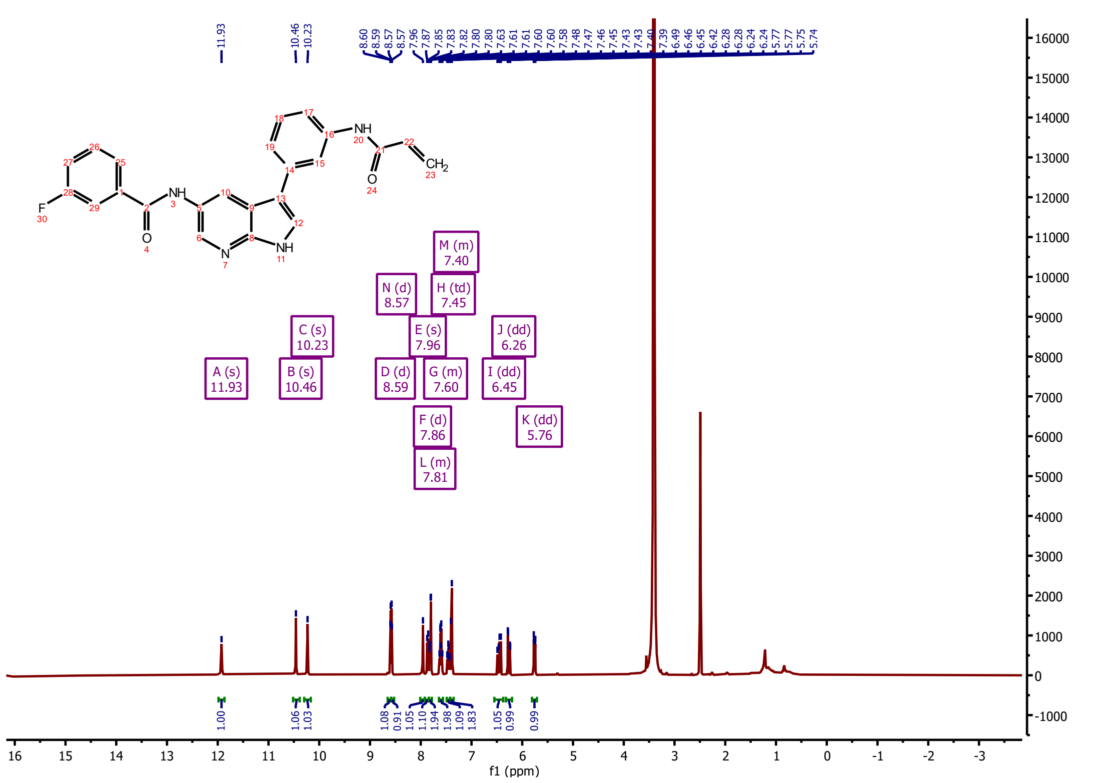

Figure S 30. ^{1}H-NMR spectrum of **11l** in DMSO- d_6.

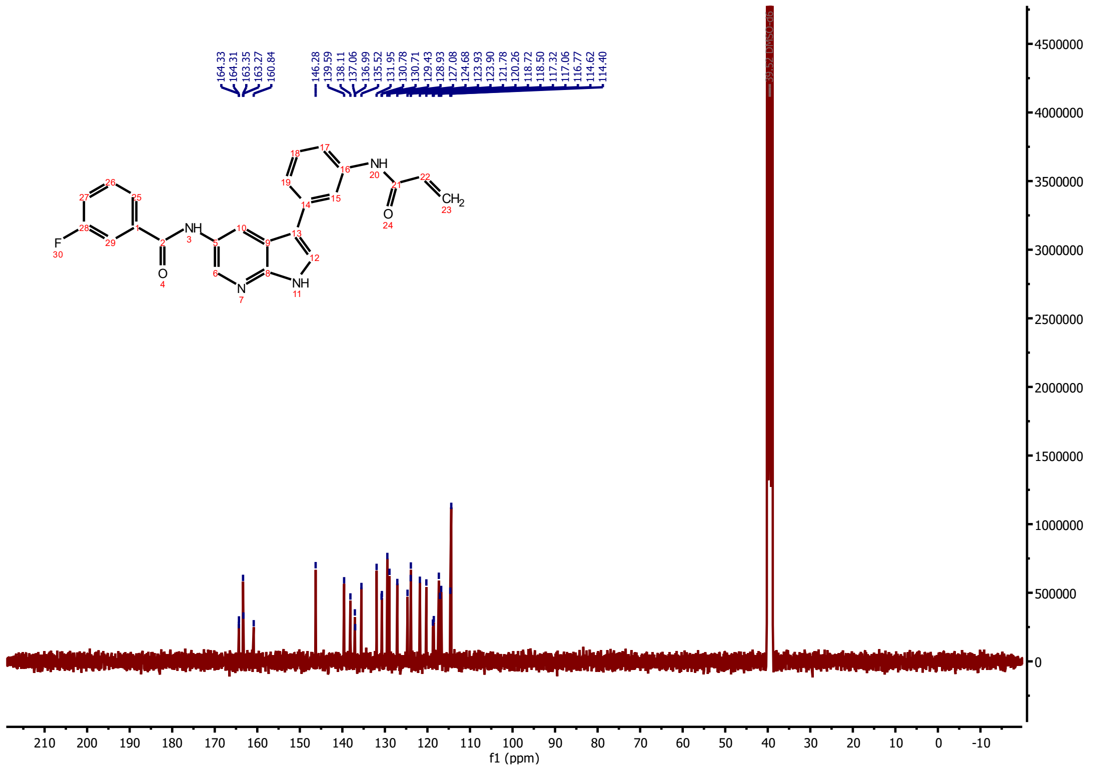

Figure S 31. ^{13}C-NMR spectrum of **11l** in DMSO- d_6.

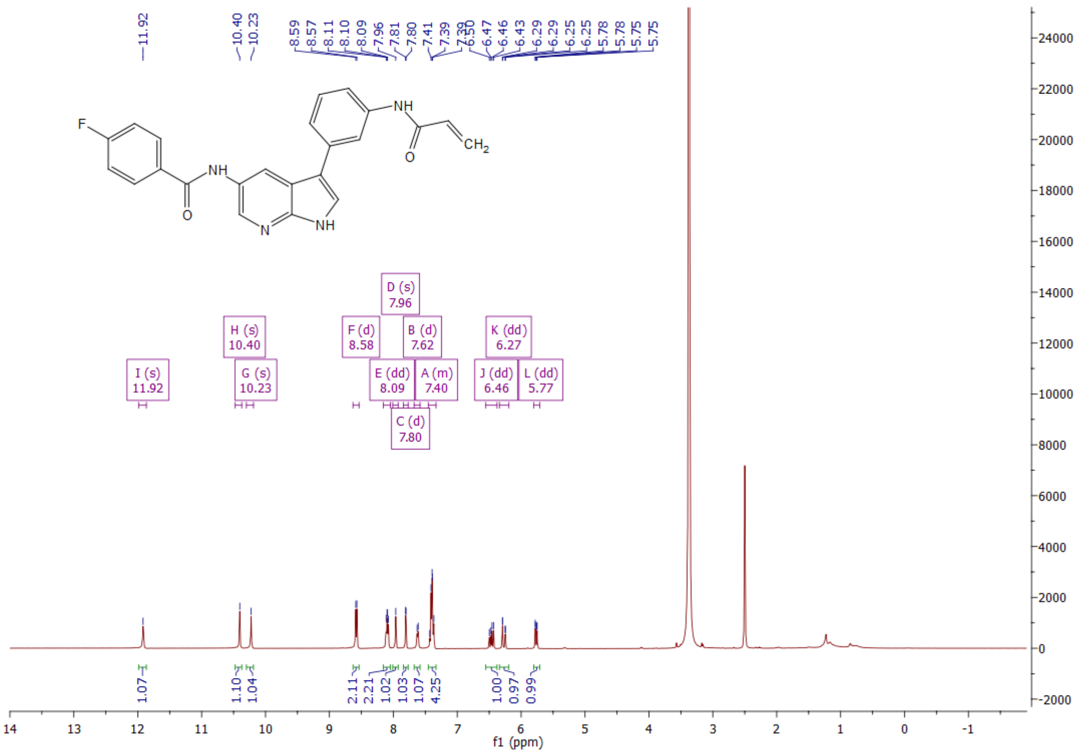

Figure S 32. ^{1}H-NMR spectrum of **11m** in DMSO- d_6.

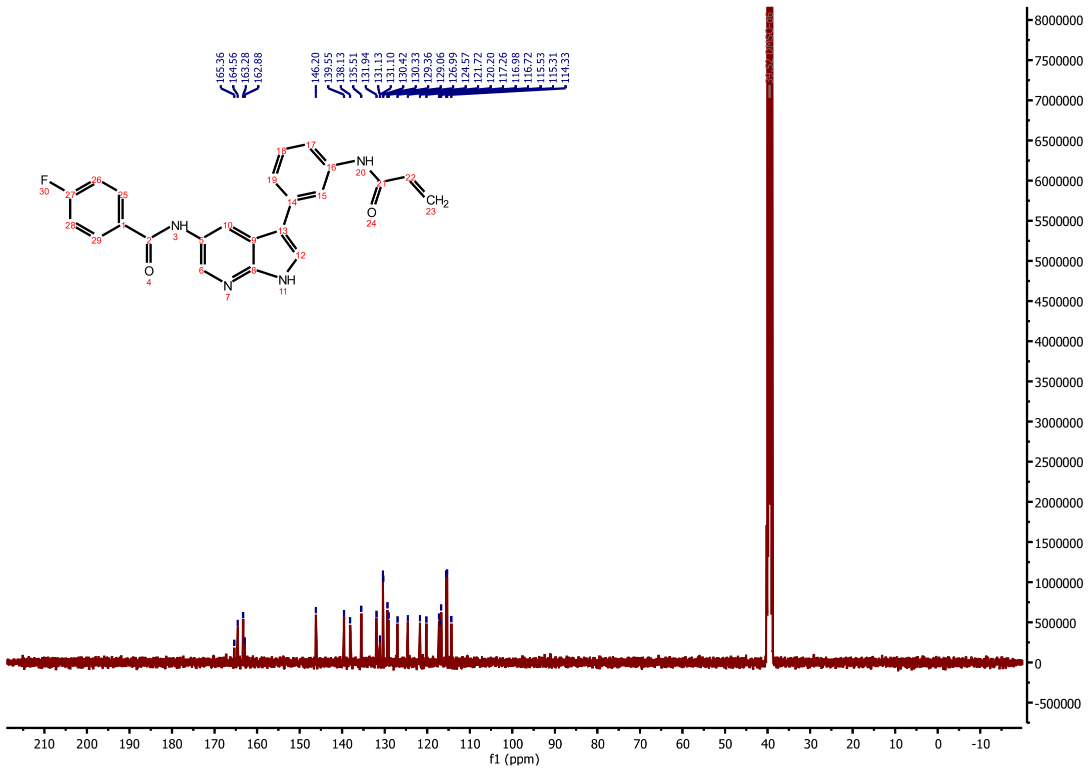

Figure S 33. ^{13}C-NMR spectrum of **11m** in DMSO- d_6.

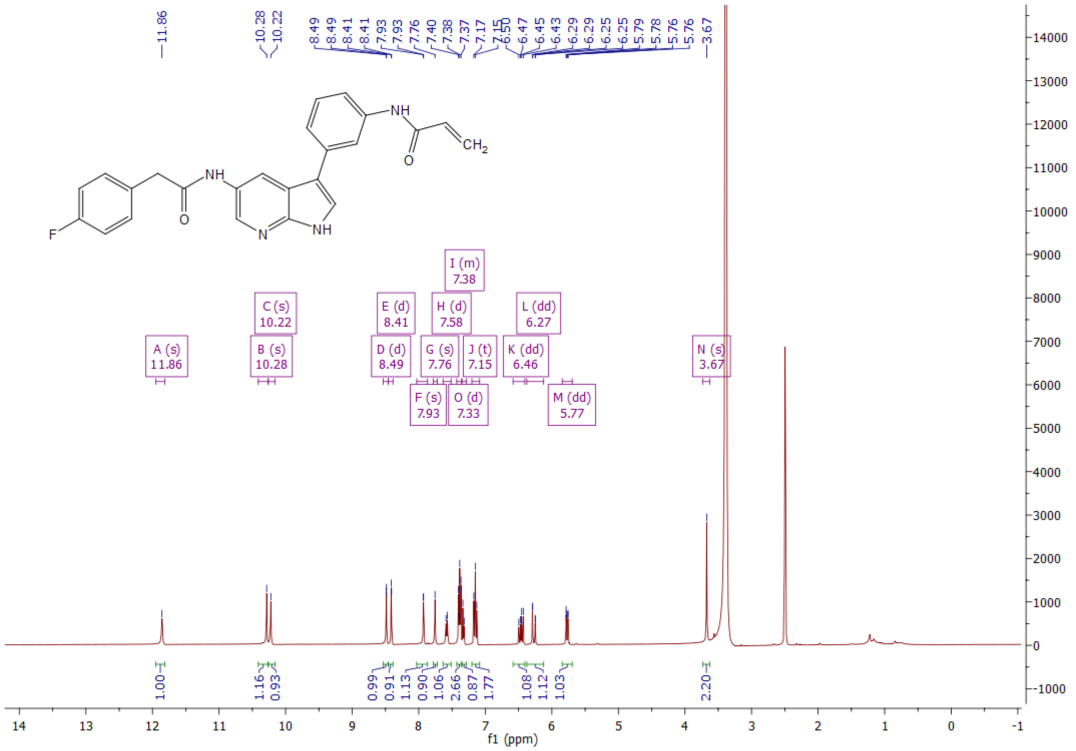

Figure S 34. ^{1}H-NMR spectrum of **11n** in DMSO- d_6.

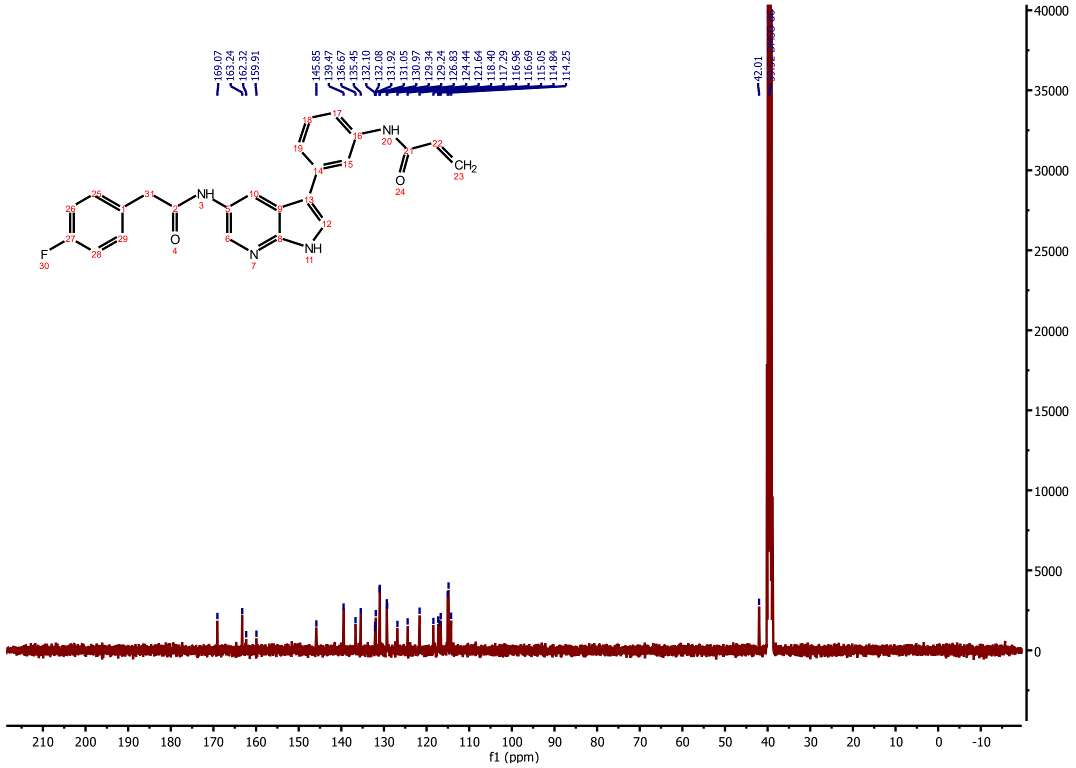

Figure S 35. ^{13}C-NMR spectrum of **11n** in DMSO- d_6.

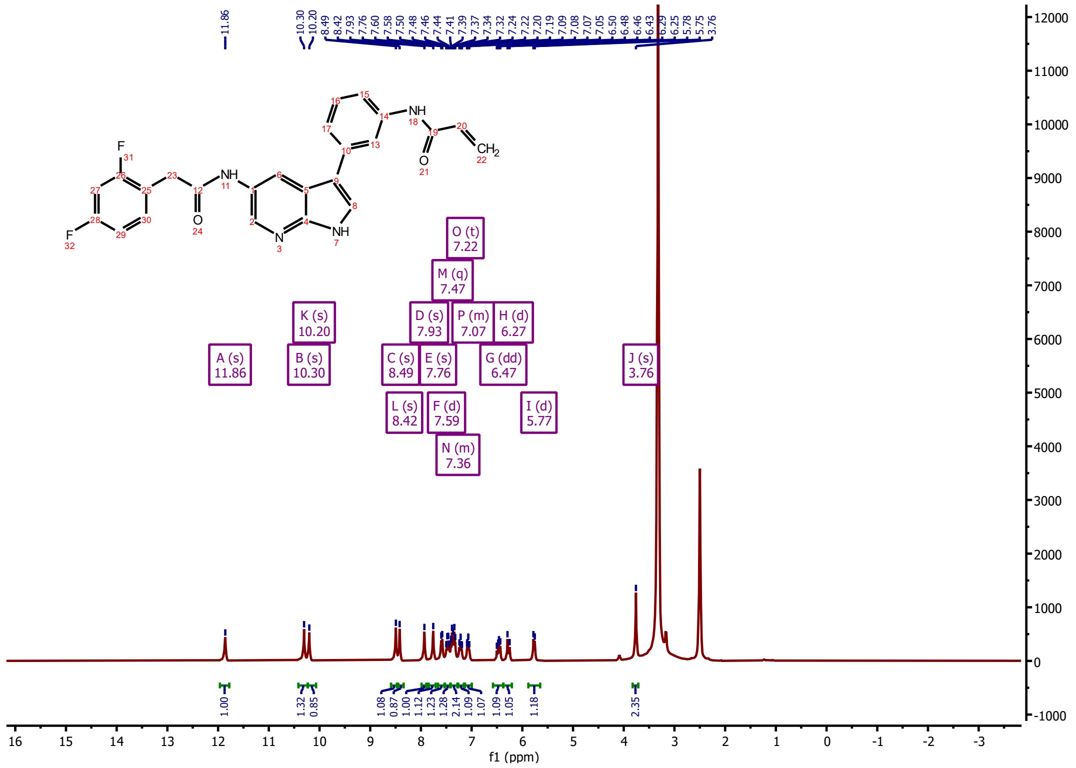

Figure S 36. ^{1}H-NMR spectrum of **11o** in DMSO- d_6.

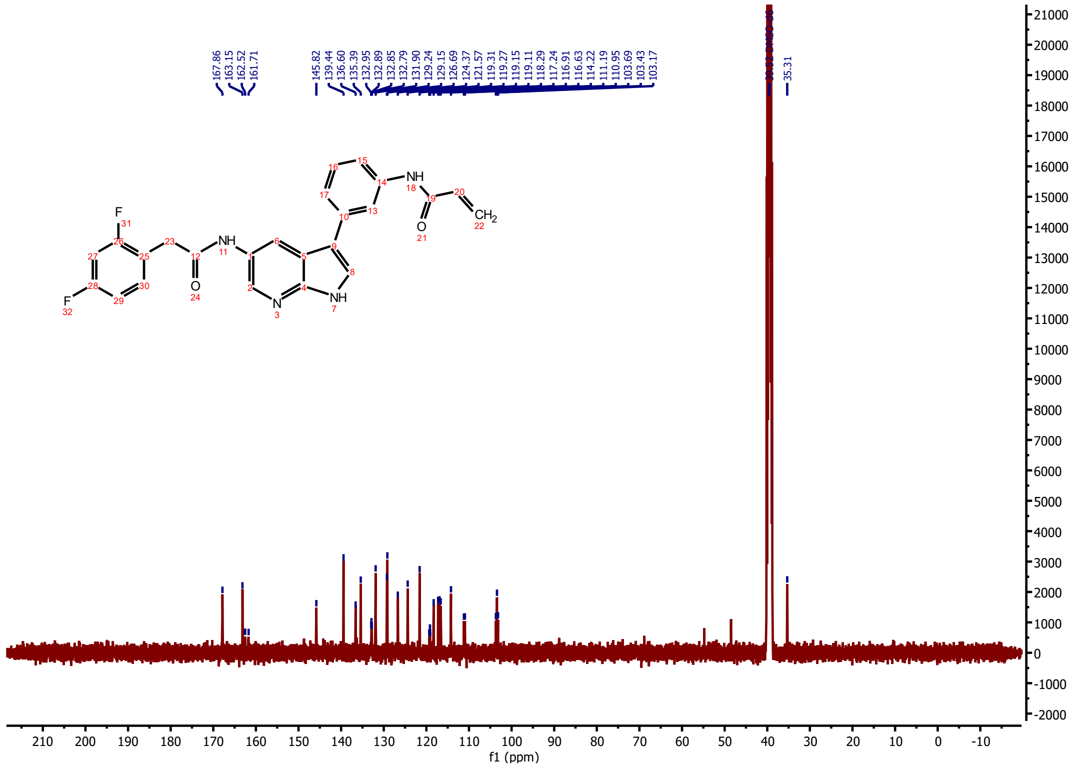

Figure S 37. ^{13}C-NMR spectrum of **11o** in DMSO- d_6.

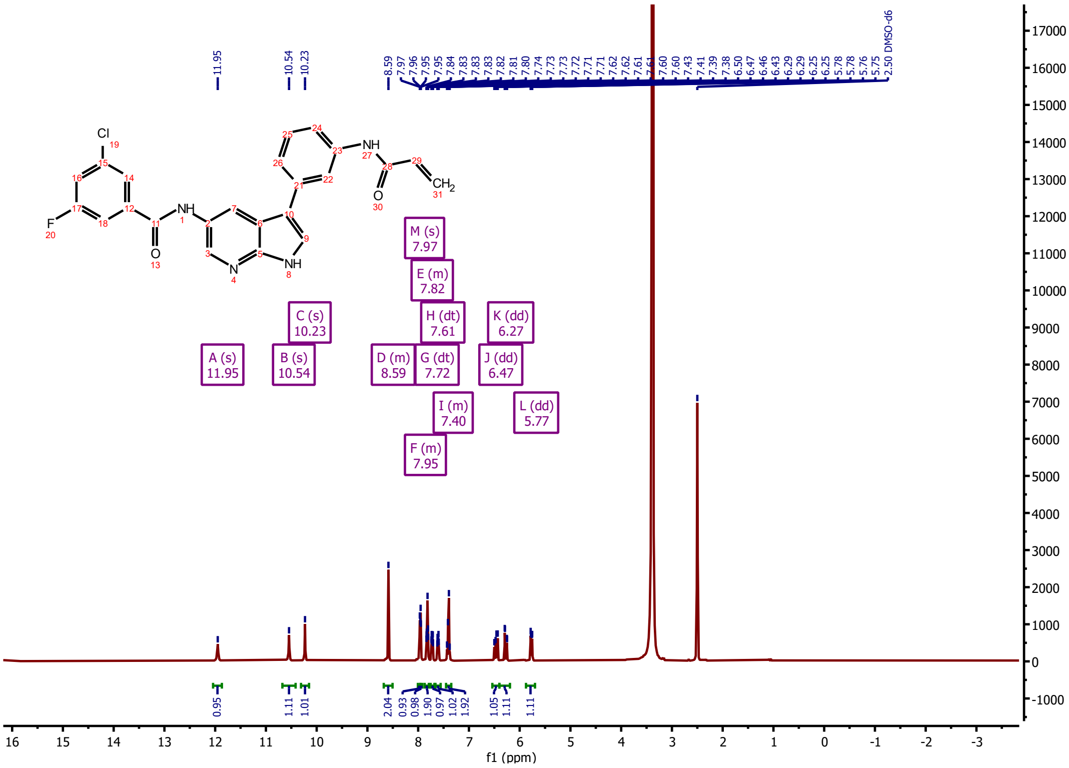

Figure S 38. ^{1}H-NMR spectrum of **11p** in DMSO- d_6.

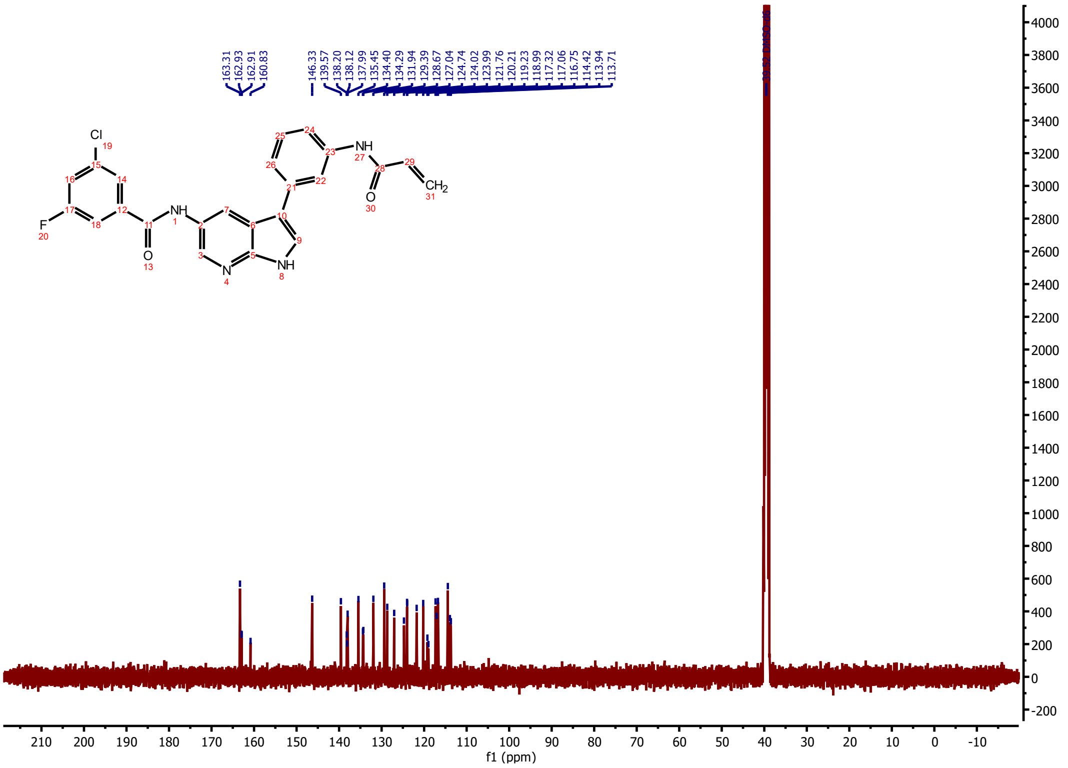

Figure S 39. ^{13}C-NMR spectrum of **11p** in DMSO- d_6.

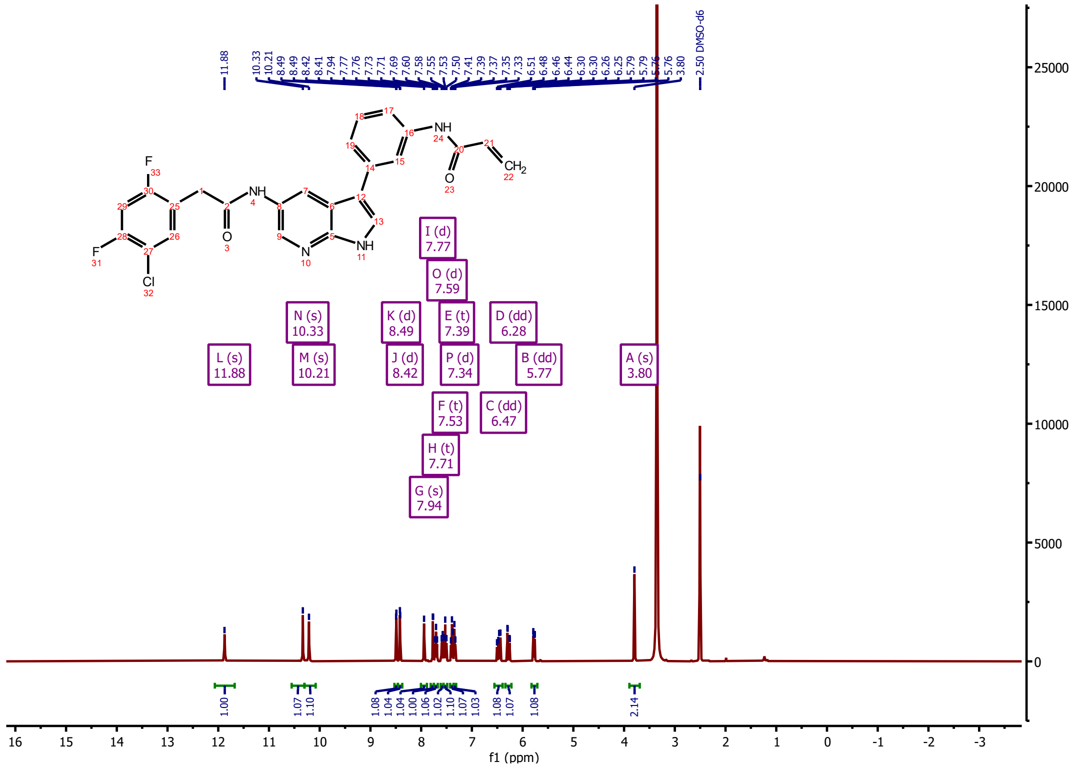

Figure S 40. ^{1}H-NMR spectrum of **11q** in DMSO- d_6.

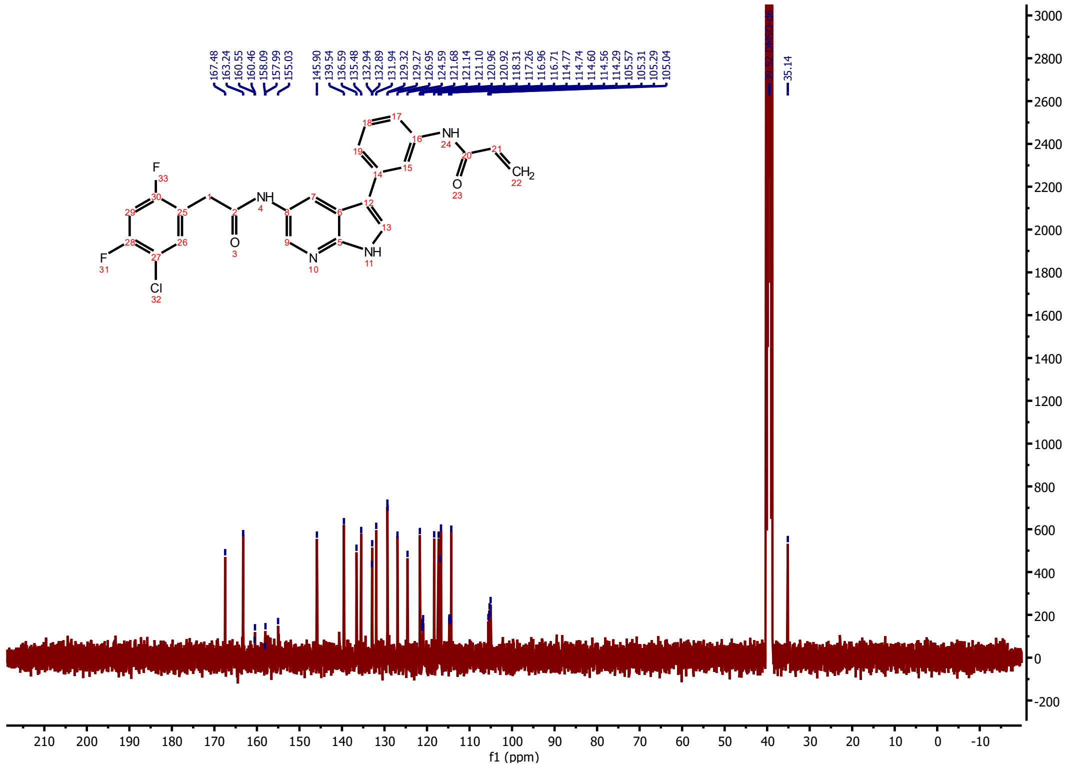

Figure S 41. ^{13}C-NMR spectrum of **11q** in DMSO- d_6.

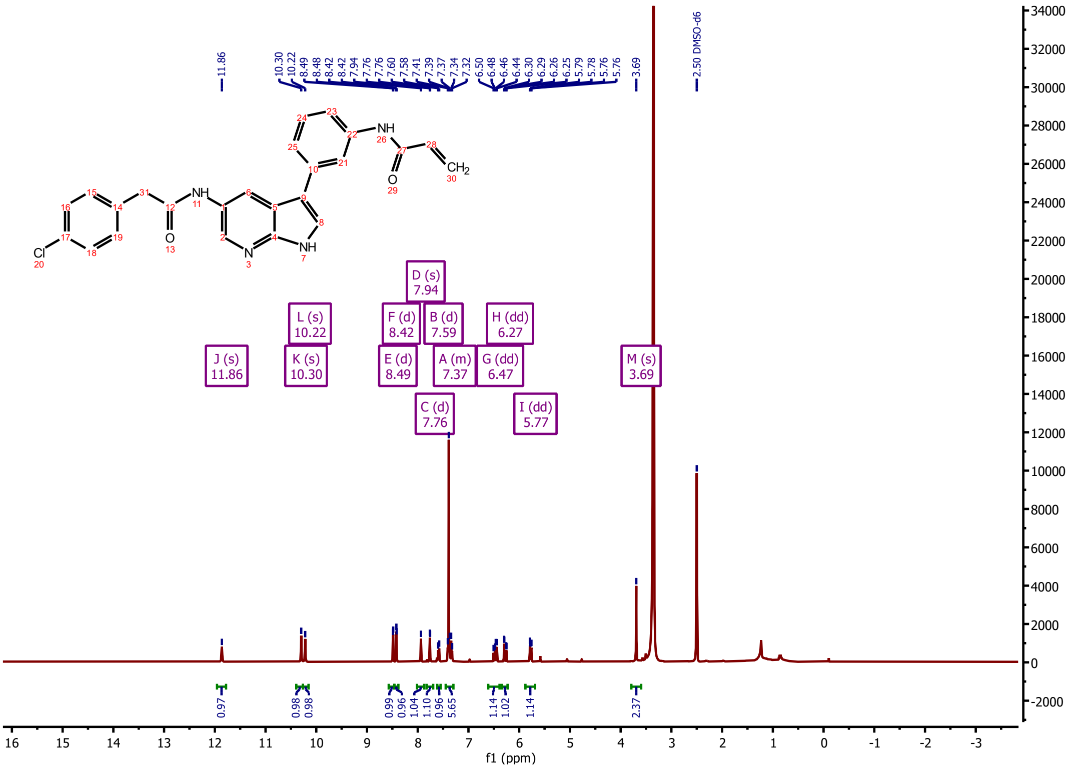

Figure S 42. ^{1}H-NMR spectrum of **11r** in DMSO- d_6.

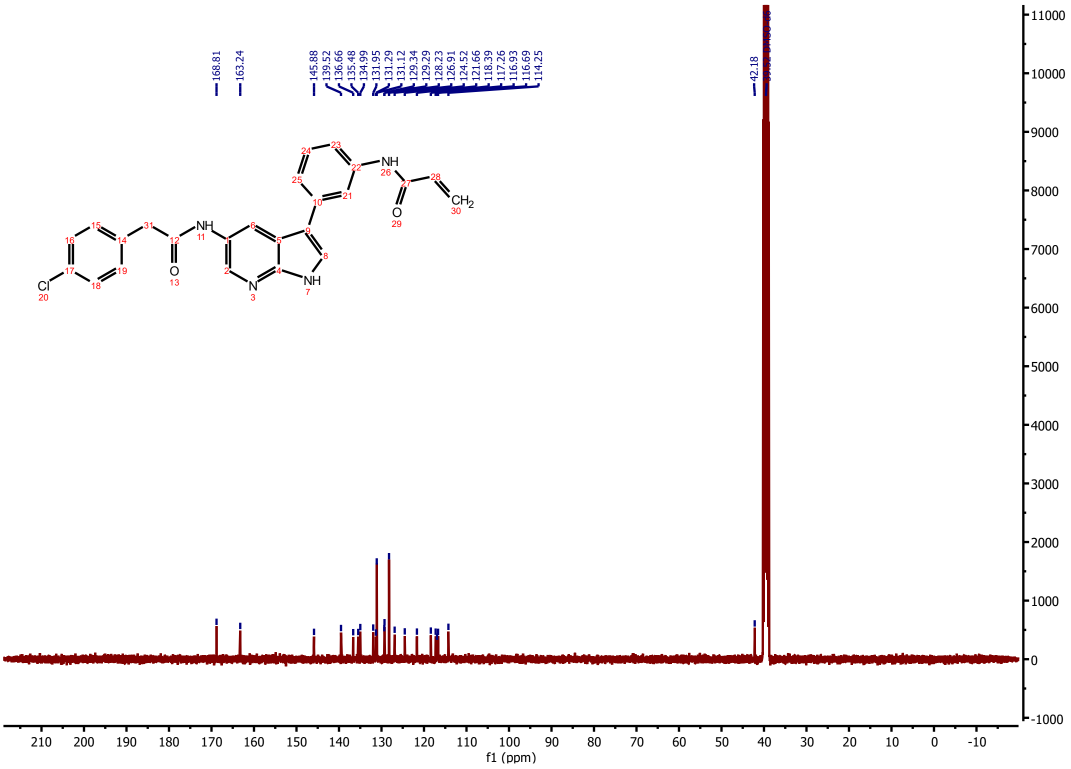

Figure S 43. ^{13}C-NMR spectrum of **11r** in DMSO- d_6.

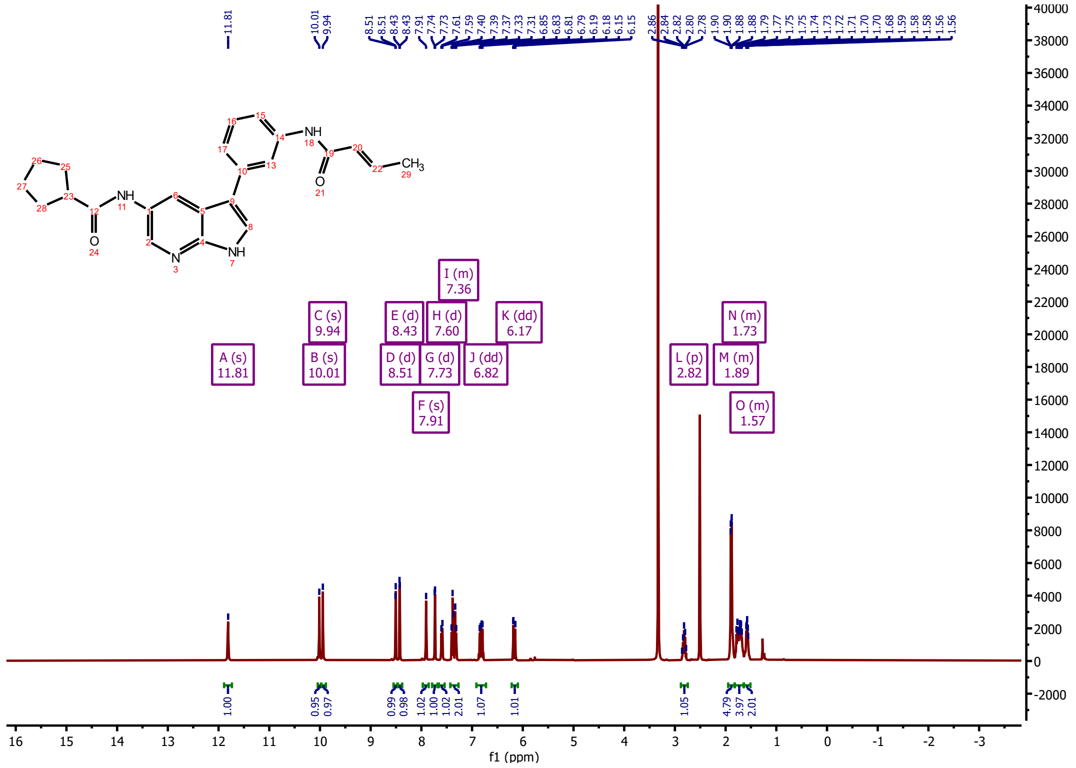

Figure S 44. ^{1}H-NMR spectrum of **12a** in DMSO- d_6.

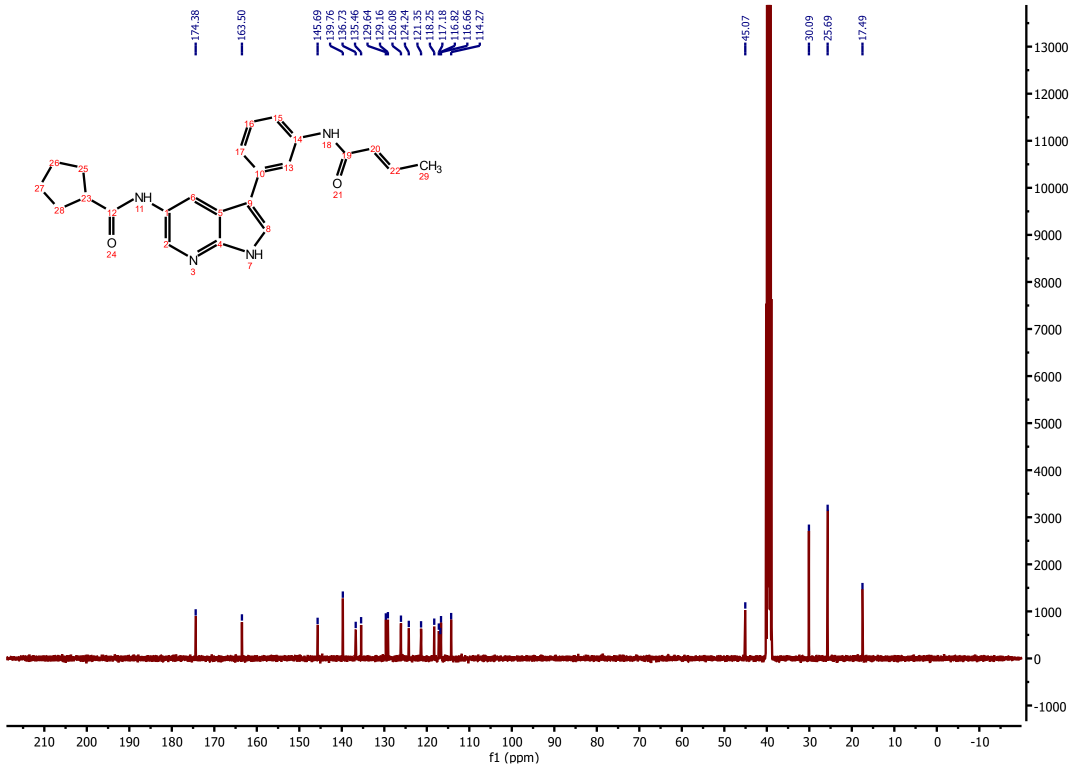

Figure S 45. ^{13}C-NMR spectrum of **12a** in DMSO- d_6.

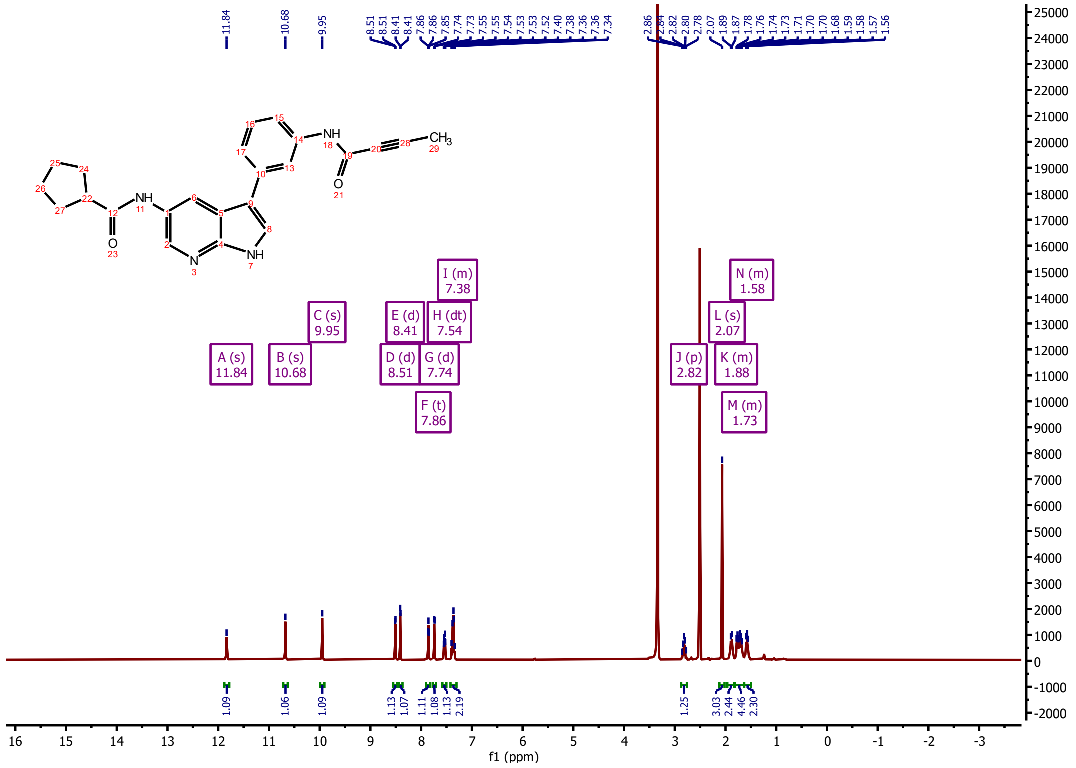

Figure S 46. ^{1}H-NMR spectrum of **12b** in DMSO- d_6.

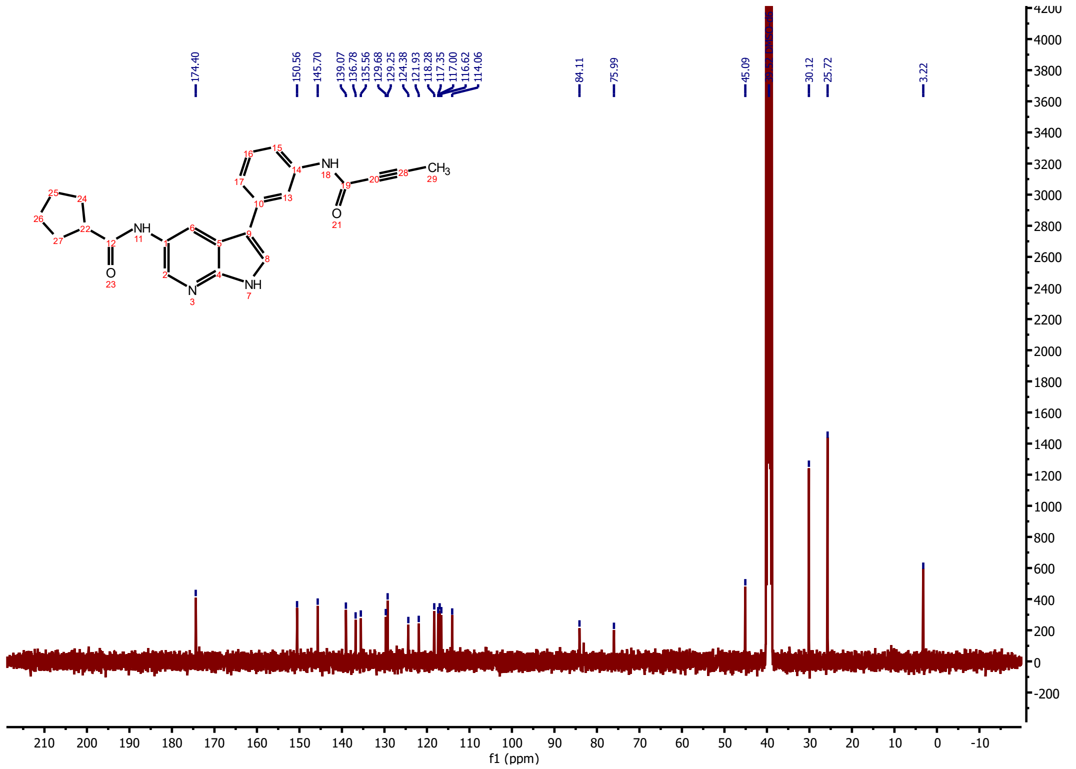

Figure S 47. ^{13}C-NMR spectrum of **12b** in DMSO- d_6.

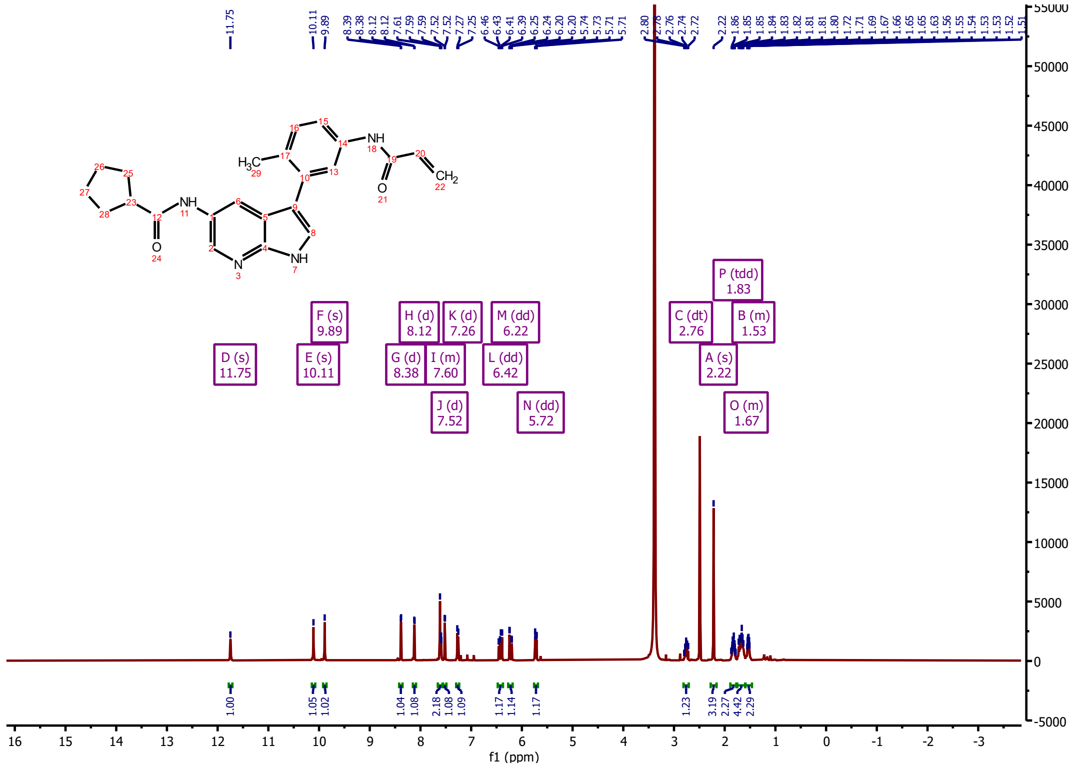

Figure S 48. ^{1}H-NMR spectrum of **12c** in DMSO- d_6.

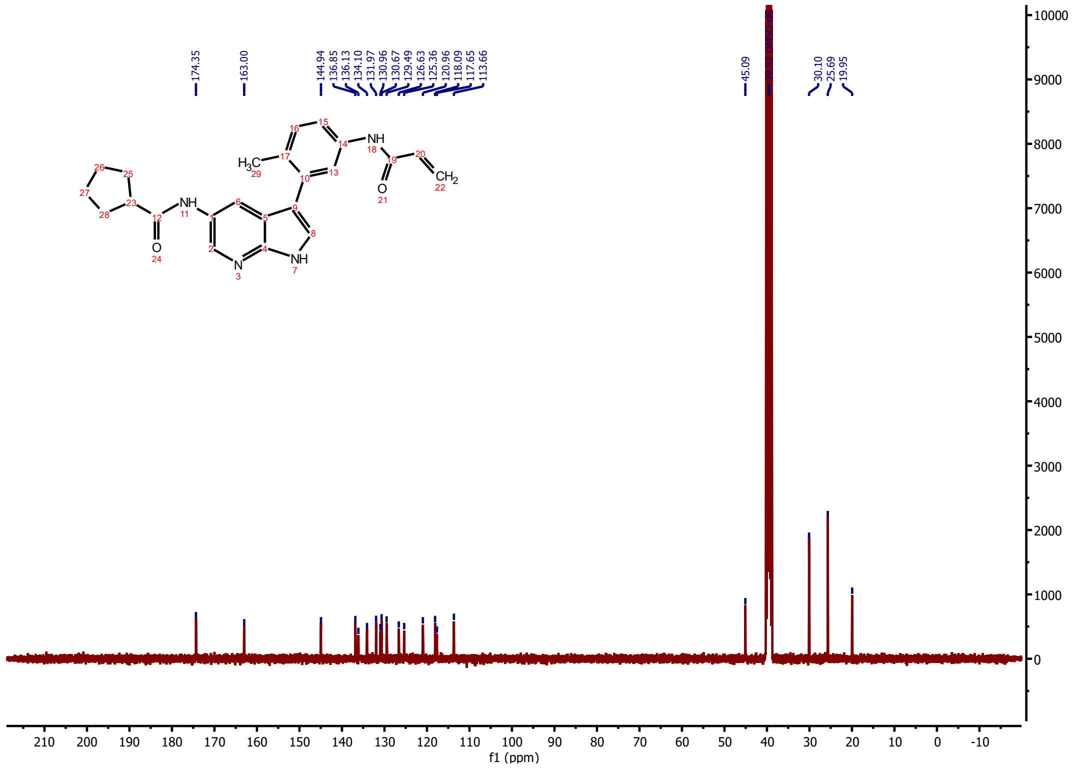

Figure S 49. ^{13}C-NMR spectrum of **12c** in DMSO- d_6.

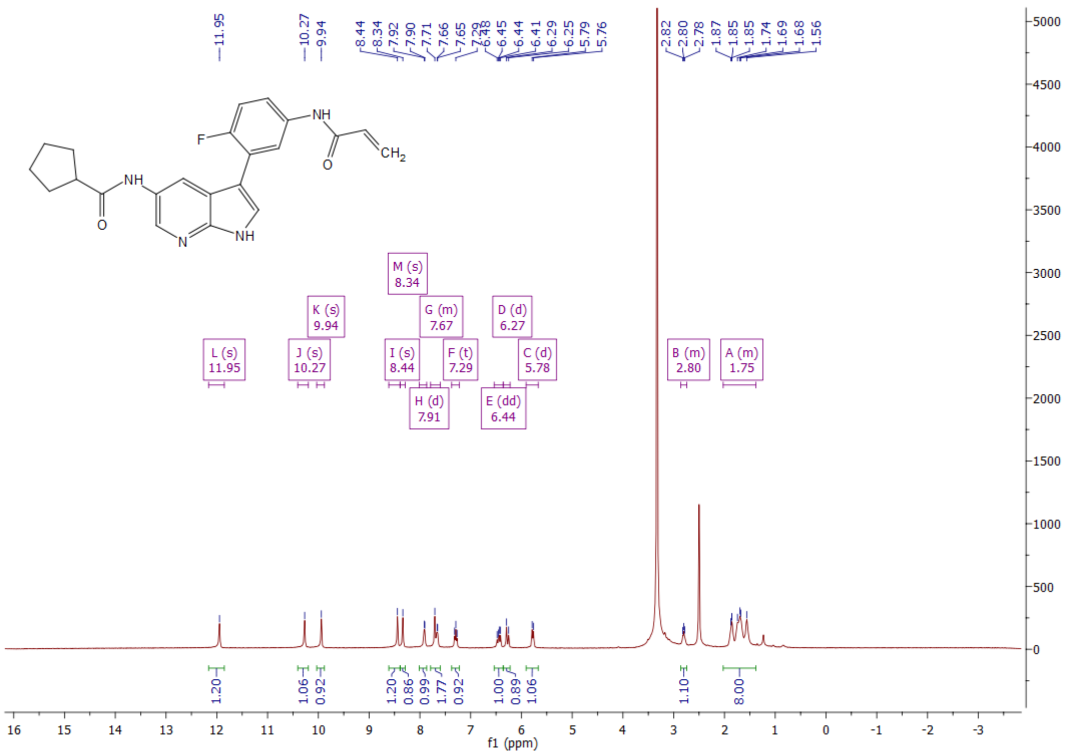

Figure S 50. ^{1}H-NMR spectrum of **12d** in DMSO- d_6.

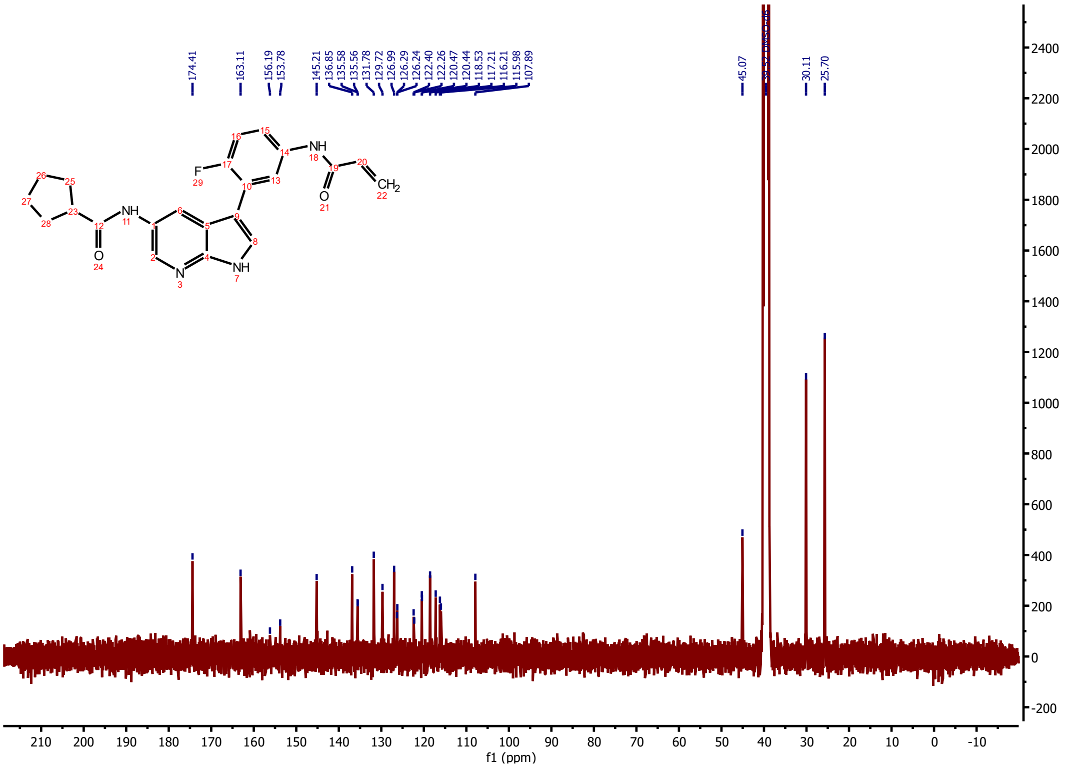

Figure S 51. ^{13}C-NMR spectrum of **12d** in DMSO- d_6.

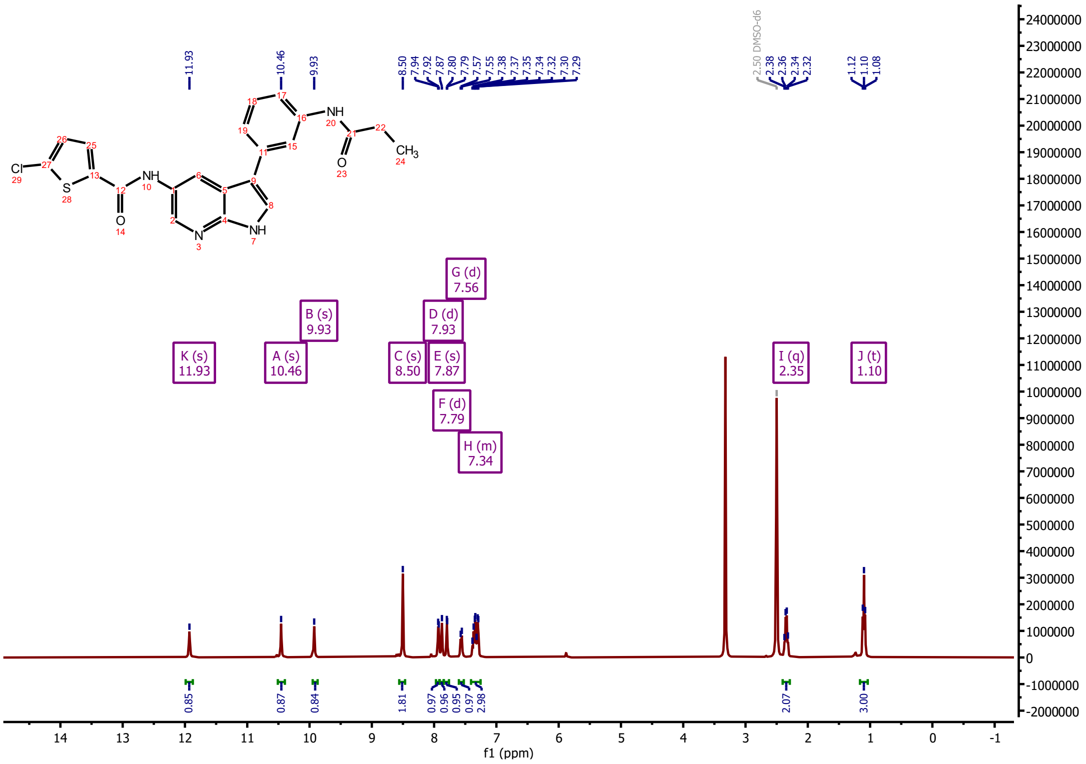

Figure S 52. ^{1}H-NMR spectrum of **33** in DMSO- d_6.

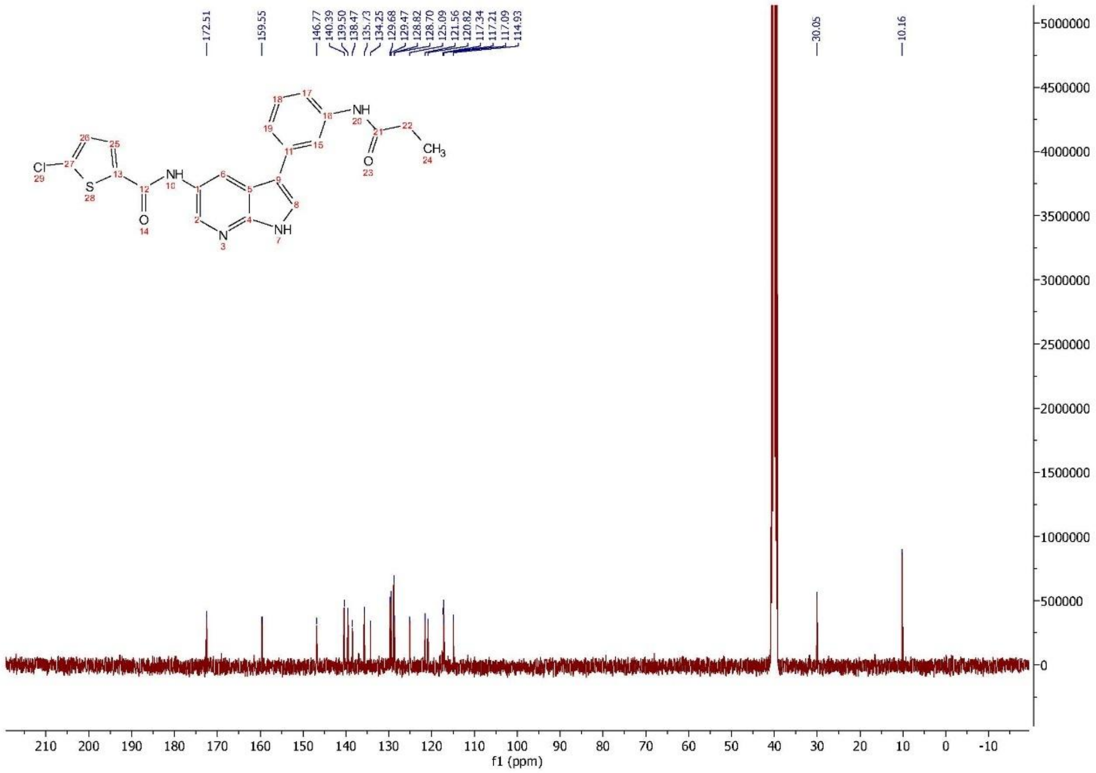

Figure S 53. ^{13}C-NMR spectrum of **33** in DMSO- d_6.

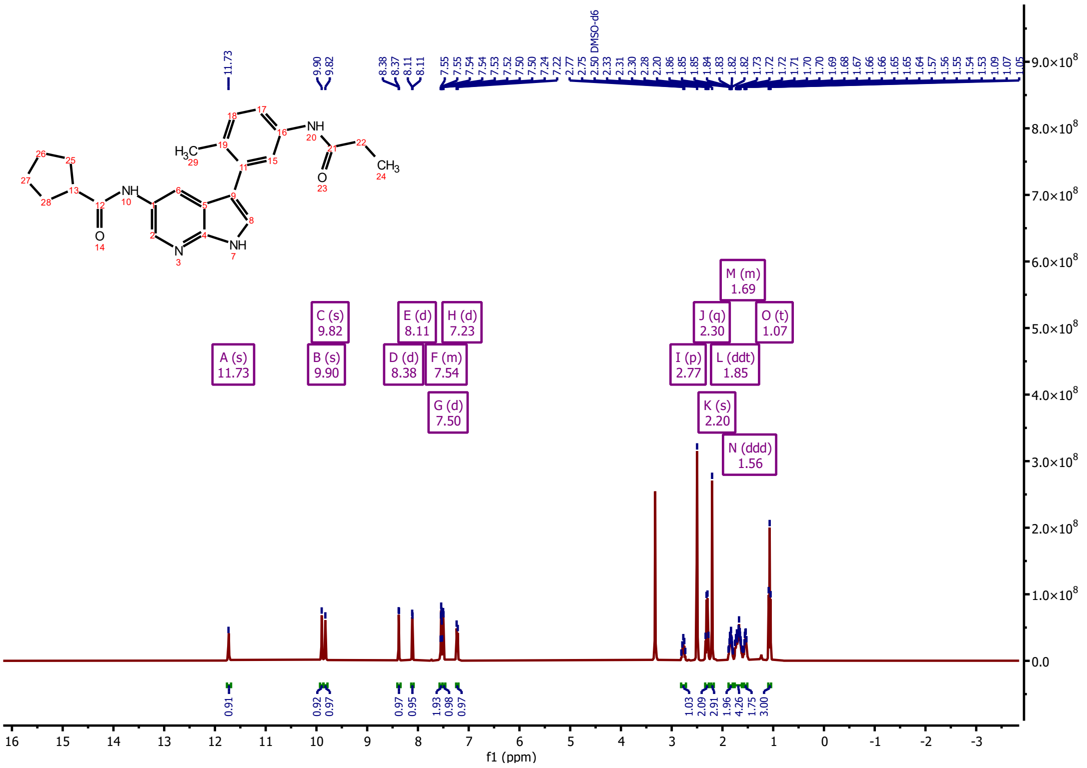

Figure S 54. ^{1}H-NMR spectrum of **34** in DMSO- d_6.

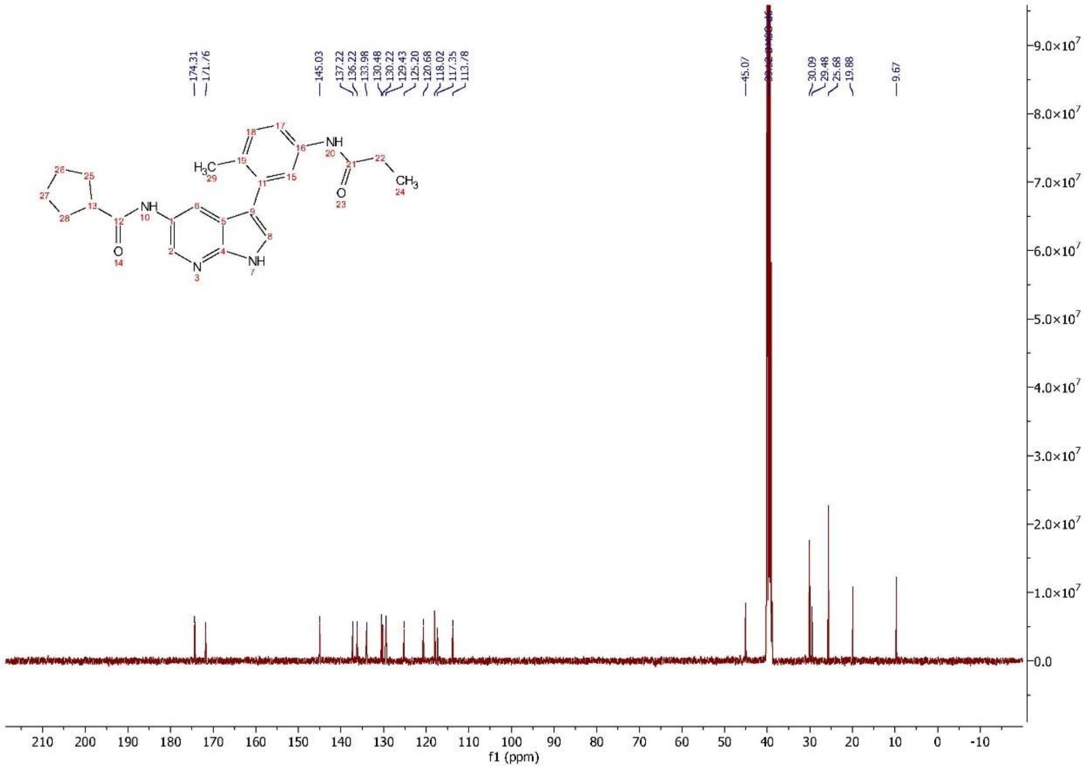

Figure S 55. ^{13}C-NMR spectrum of **34** in DMSO- d_6.
